# Analysis of BAC-end sequences in rainbow trout: Content characterization and assessment of synteny between trout and other fish genomes

**DOI:** 10.1186/1471-2164-12-314

**Published:** 2011-06-14

**Authors:** Carine Genet, Patrice Dehais, Yniv Palti, Guangtu Gao, Frederick Gavory, Patrick Wincker, Edwige Quillet, Mekki Boussaha

**Affiliations:** 1 INRA, UMR 1313 GABI, Génétique Animale et Biologie Intégrative, 78350 Jouy-en-Josas, France; 2 INRA, UMR 444 ENVT Génétique Cellulaire, 31326 Castanet-Tolosan, France; 3 INRA, Sigenae, 31326 Castanet-Tolosan, France; 4 National Center for Cool and Cold Water Aquaculture, ARS-USDA, 11861 Leetown Road, Kearneysville, WV 25430, USA; 5 CEA Genoscope, 2 rue Gaston Crémieux, 91057 Evry Cedex, France

## Abstract

**Background:**

Rainbow trout (*Oncorhynchus mykiss*) are cultivated worldwide for aquaculture production and are widely used as a model species to gain knowledge of many aspects of fish biology. The common ancestor of the salmonids experienced a whole genome duplication event, making extant salmonids such as the rainbow trout an excellent model for studying the evolution of tetraploidization and re-diploidization in vertebrates. However, the lack of a reference genome sequence hampers research progress for both academic and applied purposes. In order to enrich the genomic tools already available in this species and provide further insight on the complexity of its genome, we sequenced a large number of rainbow trout BAC-end sequences (BES) and characterized their contents.

**Results:**

A total of 176,485 high quality BES, were generated, representing approximately 4% of the trout genome. BES analyses identified 6,848 simple sequence repeats (SSRs), of which 3,854 had high quality flanking sequences for PCR primers design. The first rainbow trout repeat elements database (INRA RT rep1.0) containing 735 putative repeat elements was developed, and identified almost 59.5% of the BES database in base-pairs as repetitive sequence. Approximately 55% of the BES reads (97,846) had more than 100 base pairs of contiguous non-repetitive sequences. The fractions of the 97,846 non-repetitive trout BES reads that had significant BLASTN hits against the zebrafish, medaka and stickleback genome databases were 15%, 16.2% and 17.9%, respectively, while the fractions of the non-repetitive BES reads that had significant BLASTX hits against the zebrafish, medaka, and stickleback protein databases were 10.7%, 9.5% and 9.5%, respectively. Comparative genomics using paired BAC-ends revealed several regions of conserved synteny across all the fish species analyzed in this study.

**Conclusions:**

The characterization of BES provided insights on the rainbow trout genome. The discovery of specific repeat elements will facilitate analyses of sequence content (e.g. for SNPs discovery and for transcriptome characterization) and future genome sequence assemblies. The numerous microsatellites will facilitate integration of the linkage and physical maps and serve as valuable resource for fine mapping QTL and positional cloning of genes affecting aquaculture production traits. Furthermore, comparative genomics through BES can be used for identifying positional candidate genes from QTL mapping studies, aid in future assembly of a reference genome sequence and elucidating sequence content and complexity in the rainbow trout genome.

## Background

Rainbow trout (*Oncorhynchus mykiss*) are cultivated worldwide for aquaculture production. Trout farming has been successful in North America, the species native area, as well as in many other regions, including Chile and a number of European countries where rainbow trout had been introduced since the 19^th ^century. In 2008, total world production was about 576,000 metric tons with a total export value estimated around 2.4 billions USD (http://www.fao.org/fishery/statistics/en).

The rainbow trout is one of the most intensively studied fish species. Several features such as *in vitro *fertilization, ease of rearing and gamete handling and a large body size with large and clearly defined tissues, make it a particularly suited model to carry out a range of investigations. Hence, considerable amount of basic knowledge has been accumulated in many areas such as physiology, nutrition, behaviour, ecology, genetics, pathology, comparative immunology, carcinogenesis and toxicology (reviewed in [1]).

Combining biological and phenotypic data with genomic information can be used to increase our basic knowledge of the regulation of biological functions, and ultimately used in applied research to improve the environmental and genetic management of aquaculture production systems with focus on complex traits such as meat and carcass quality, stress tolerance or resistance to specific pathogens.

The rainbow trout genome size was estimated to be between 2.4 and 3.0 × 10^9 ^base pairs (bp) [2]. A whole genome duplication event occurred 25 to 100 million years ago in the common ancestor of the salmonids. Since that time, re-diploidization has resulted in a semi-tetraploid state [3]. Consequently, presence of duplicated genetic markers was reported [4] and many homeologous regions have been identified in the rainbow trout genome [5]. Although the tetraploidization event increased the genome complexity, it also makes the salmonids a very pertinent group to study the differential evolution and loss of duplicated genes in the process of re-diploidization.

Several genomic resources have been developed in rainbow trout in the last decade. Seven linkage maps based on either AFLP markers [2,6] or microsatellite markers and few SNPs [7-11] have been constructed. These maps are used for comparative mapping across salmonid species [12], for QTL mapping studies for various traits [13-20] or for linkage disequilibrium studies [21]. Attempts for high throughput discovery of SNP markers are emerging but only a limited number of true SNP have been validated up to now [22]. Large EST databases ([23,24]; http://compbio.dfci.harvard.edu/cgi-bin/tgi/gimain.pl?gudb=r_trout and http://www.sigenae.org) are available, as well as high content DNA microarrays [25,26]. Several bacterial artificial chromosome (BAC) libraries have also been established [4,27,28].

BAC libraries are a valuable genomic resource for many purposes, including clone-based sequencing, positional cloning and physical mapping. The first physical map in rainbow trout was recently built using the 10X HindIII BAC library [28]. The map contained 4,173 contigs and 9,379 singletons. The physical length of the map contigs was estimated to be approximately 2.0 Gb, which represents almost 83% of rainbow trout genome.

BAC-end sequencing has been initially proposed to be an efficient approach for whole genome sequencing projects [29], for comparative physical mapping [30,31], and for the development of molecular markers, mainly microsatellites [32]. In the absence of whole genome sequences, BES analysis can elucidate sequence content and complexity, including gene density, potential transposable elements, and microsatellite markers [33]. Furthermore, paired BAC-end sequences can be very useful for scaffolding in whole-genome sequencing assembly projects.

Here we report on the sequencing and characterization of BAC-end sequences (BES) from more than half of the clones from the rainbow trout 10X HindIII BAC library. The sequence content was analysed for putative genes, repetitive elements and simple sequence repeats (SSR). BES gene content was then used to establish regions of conserved synteny with other fish genomes.

## Results and Discussion

### BAC-end sequencing statistics

Sequencing of rainbow trout BAC ends generated 177,857 raw data reads of more than 100 bp from 92,593 BAC clones, of which 85,120 (~92%) had both ends sequenced and 7,473 had only one end sequenced. An additional 1,372 BES were filtered-out because of high similarity to bacterial and vector sequences or because of low quality sequence. The total of high quality rainbow trout sequence reads was 176,485 including one hundred forty eight BES sequenced twice for quality control purposes.

The PHRED Q20 read length ranged from 101 to 832 bp with a mean of 546 bp. The trout BES Q20 average length was similar to catfish [34] but lower than Atlantic Salmon [35]. Overall, BAC-end sequencing generated a total 96,298,179 bp of genomic sequence representing approximately 4% of the trout genome (assuming genome size of 2.4 × 10^9 ^bp). The GC content was estimated to be around 42%, which is lower than channel catfish [34] and stickleback (44%), but higher than zebrafish (36%) and medaka (40%) (http://genome.ucsc.edu).

### Preliminary survey of repeat content from rainbow trout genome

Few studies have reported the identification and characterization of repeat elements in salmonids, resulting in the absence of sufficient repeat masking data set for rainbow trout. Repbase update release 13.05 contains only 145 ancestral shared repeats and one lineage-specific repeat for rainbow trout, and for salmon, 141 ancestral shared repeats and five lineage-specific repeats [36]. Consequently, masking of rainbow trout BES using RepBase 13.05 generated only 1.66% of masked sequences. Therefore, we used the BES data to generate a new rainbow trout repeat library. This database contains 735 putative elements and was named INRA RT rep1.0 (available as Additional file 1). Repeat element analysis using the new INRA RT rep1.0 library masked almost 59.5% of the BES database in base-pairs (data not shown). The most abundant repeat elements were DNA transposons, and the most common transposon type was the TcMar-Tc1 transposon-related sequence (24.5%) (Table 1). Unknown elements were also abundant and accounted for 19.2% of BES (Table 1).

**Table 1:** Characteristics of the INRA RT rep1.0 database.

	Number of elements	Percentage of sequence		Number of elements	Percentage of sequence
**Class I elements**			**Class II elements**		

**LTR retrotransposons**			DNA	1	0,05
LTR/DIRS1	2	0,02	DNA/Harbinger	2	0,02
LTR/Gypsy	21	1,68	DNA/hAT	9	0,27
**Non-LTR retrotransposons**			DNA/hAT-Charlie	5	0,26
LINE	1	0,29	DNA/hAT-Tag1	1	0,05
LINE/I	1	0,01	DNA/hAT-Tip100	1	0,01
LINE/Jockey	3	0,05	DNA/Helitron	1	0,01
LINE/L1	6	0,14	DNA/Maverick	1	0,01
LINE/L2	19	2,18	DNA/Tc1	11	4,26
LINE/Penelope	1	0,05	DNA/TcMar-Tc1	46	24,51
LINE/Rex1	6	3,03	DNA/TcMar-Tc2	1	0,01
SINE	5	1,09	**Others elements**		
SINE/Deu	5	0,23	Simple_repeat	22	1,19
SINE/tRNA-Lys	1	0,01	rRNA	3	0,11
SINE/5S	1	0,01	Satellite	2	0,06
SINE?	8	0,37	Unknown elements	549	19,19

Columns 1 and 4 show the name of the repeat elements. Columns 2 and 5 provide the number of repeats elements in the INRA RT rep1.0 generated in this work. Columns 3 and 6 correspond to the percentage of total base pairs masked by the corresponding repeat element.

For comparison we masked the rainbow trout BES dataset with the Atlantic salmon repeat database (http://web.uvic.ca/grasp and [37]) and found that only 52.3% of the BES dataset was masked compared to 59.5% with the rainbow trout repeat database. We also estimated the redundancy of each species specific database by repeatmasking the INRA rep1.0 by Salmon Raw 1.6 database and *vice versa*. We observed that respectively 35.3% and 52.8% of the databases were masked suggesting differences in the repeats content between the two salmonid species. However, it is also possible that the restriction enzyme selection for the BAC libraries preparation (EcoRI for Atlantic salmon and HindIII for rainbow trout) might have imposed some bias on the repeats content of each database.

**Figure 1:**
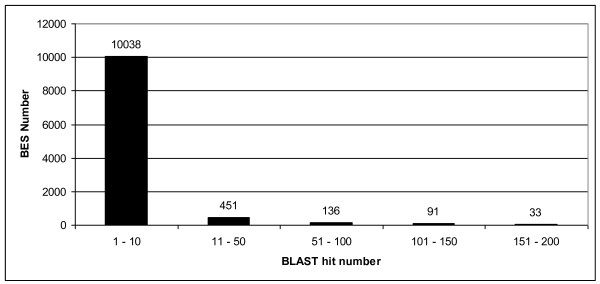
**Low complexity sequences identified through BLAST search of masked BES against themselves**. × axis represents the distribution of BLAST hits. Y axis represents the number of BES.

In addition to the automated detection of repeats using RepeatModeler, we checked for undetected repetitive elements by aligning the masked BES reads to each other. The alignment results were classified according to the number of significant hits and are summarized in Figure 1. Most of the BES reads (93.4%) had less than 10 hits, indicating that masking with the INRA RT rep1.0 library was effective. However, 711 reads (6.6%) produced more than 10 hits, suggesting that these BES reads may contain repetitive DNA sequences. Moreover, almost 263 reads (2.4%) produced more than 50 hits, suggesting that these BES reads contain interspersed repeats not yet in INRA RT rep1.0 database.

Some classes of repeat elements in salmonids can be as long as 1,500 bp [37]. Progress is currently being made to further characterise these long interspersed repeats in rainbow trout by using full length BAC-insert sequencing which will enable characterizing full-length copies of repeat elements and identifying new repeats (Jean-Nicolas Volff, personal communication).

#### Development of microsatellite markers

A total of 6,848 microsatellites were identified in 6,196 BES reads (Table 2). Approximately 56% of the microsatellites (3,854) were suitable for PCR primers design as they were flanked by sequences of at least 50 bp. We were able to define 2,061 primer-pairs (~30%) from 1,923 distinct BES as more than one microsatellite can be detected in a single BES.

**Table 2:** Distribution of Simple Sequence Repeats in trout BAC end sequences.

Repeat	Type	Number	Repeat	Type	Number
**Monomer**	A/T	71	**Tetramer**	CACA/TGTG	38
	C/G	1		TCTG/CAGA	37
**Dimer**	TG/CA	1346		TATG/CATA	26
	AC/GT	1238		AGAC/GTCT	38
	GA/TC	607		GAGA/TCTC	24
	TA/TA	505		ACAT/ATGT	22
	AG/CT	498		AGAA/TTCT	17
	AT/AT	311		Other	370
**Trimer**	AAC/GTT	5	**Pentamer**	TCAAA/TTTGA	19
	CAT/ATG	13		ATTTG/CAAAT	15
	AAT/ATT	36		Other	92
	ATA/TAT	36	**Hexamer**		356
	TTA/TAA	24	**Heptamer**		80
	Other	55	**Octomer**		278
**Tetramer**	ACAC/GTGT	26	**Nanomer**		144
	TGTA/TACA	66	**Decamer**		478
	ATAC/GTAT	23			
	CTCA/TGAG	25	Total		6,920
					

Di-nucleotides were the most abundant repeat motif (65.1%) followed by tetra-nucleotides (10.3%) (Table 2). The most abundant di-nucleotide repeats were TG/CA accounting for 19.5%. AC/GT and GA/TC repeats accounted for 17.9% and 8.8%, respectively. Polymorphism and usefulness of the BES microsatellites for linkage analysis and genetic mapping was assessed in the USDA-NCCCWA panel of five families [9] using 193 markers (Additional file 2). Out of the 193 loci tested, 126 (65.3%) were polymorphic with allele numbers ranging from 2 to 12; 57 (29.5%) failed to produce a specific PCR product and 10 (5.2%) were monomorphics. Fifteen microsatellite loci were duplicated, of which nine were useful for linkage mapping. These results show that most of the microsatellite markers developed from BES are polymorphic in the NCCCWA reference families and thus can be used for direct integration between the genetic and physical maps of the rainbow trout genome [38].

### Homology with other fish genomes

We investigated the BES sequence homology and gene content by computational and manual annotations. Of the 176,485 high quality BES reads, 97,846 BES reads (55%) had more than 100 base pairs of contiguous non repetitive sequences and were used for assessing genome syntenies by BLASTN and BLASTX similarity searches against the ENSEMBL genome and peptide databases of zebrafish, medaka and stickleback. The fractions of the 97,846 non-repetitive trout BES reads that had significant BLASTN hits against the zebrafish, medaka and stickleback genome databases were 15%, 16.2% and 17.9%, respectively, while the fractions of the non-repetitive BES reads that had significant BLASTX hits against the zebrafish, medaka, and stickleback protein databases were 10.7%, 9.5% and 9.5%, respectively (Tables 3 and 4). Compared to similar analyses that used BES from non-model fish species to construct "*In-silico*" comparative genome maps [39-41], the sequence homologies between rainbow trout and the model species were low, most likely due to the large evolutionary distance between the salmonids and the model species.

**Table 3:** Rainbow trout BAC end sequences BlastN statistics

	Medaka	Stickleback	Zebrafish
**Total BES with hits**	15,896	17,499	14,716
**BES with unique hits^a^**	15,379	15,612	13,868
**BACs**			
**BAC with one end**	14,206	15,467	13,304
**Paired BAC-ends**	845	1,016	706
**Paired BAC-ends with unique hits**	792	812	632
**Unique hits paired BAC-ends matched on the same chromosome**	418	566	360
**Also identified by BlastX**	85	105	105
**Microsynteny identified**	307	421	176

^a ^The bioinformatic filtering criteria used for identifying 'unique' hits are described in the methods section.

**Table 4:** Rainbow trout BAC end sequences BlastX statistics

	Medaka	Stickleback	Zebrafish
**Total BES with hits**	9,312	9,308	10,487
**BES with unique hits^a^**	9,125	9,058	9,983
**BACs**			
**BAC with one end**	8,700	8,674	9,717
**Paired BAC-ends**	306	317	385
**Paired BAC-ends with unique hits**	293	305	355
**Unique hits paired BAC-ends matched on the same chromosome**	132	148	155
**Also identified by BlastN**	85	105	105
**Microsynteny identified**	40	45	30

^a ^The bioinformatic filtering criteria used for identifying 'unique' hits are described in the methods section.

Multiple gene hits may be caused by gene duplications or by the presence of conserved sequences among members of gene families. BLASTX searches revealed hits with some genes existing in large copy numbers as exemplified by *protocadherin *members families (53 hits) and *odorant receptor *members families (48 hits) (Additional file 3). The others gene hits showed identity to transposable elements such as piggybac transposable element 4 or LINE-1 type transposase domain containing 1 or transposase (data not shown) which revealed that these transposable elements were not masked by the INRA RT rep1.0 database.

### Identification of regions of microsynteny

The sequence homology searches were conducted using both BlastN and BlastX alignment tools.

For BlastN searches, we identified 792, 812, and 632 BACs with both ends having significant unique hits to the medaka, stickleback, and zebrafish genomes, respectively (Table 3). Out of those, 418 (53%), 566 (70%), and 360 (57%) unique paired BAC-end hits matched on the same chromosome (macro-synteny) in medaka, stickleback and zebrafish, respectively. Of those, we identified 307 (73%), 421 (74%) and 176 (49%) regions of microsynteny between rainbow trout and medaka, stickleback, and zebrafish, respectively (additional file 4).

For BLASTX analysis, we identified 293, 305, and 355 BACs with both ends having significant gene hits to the medaka, stickleback, and zebrafish genomes, respectively (Table 4). Further analysis revealed that 132 (45%; medaka), 148 (48%; stickleback), and 155 (43%; zebrafish) paired BAC-end gene hits were in macro-synteny. Of those, we identified 40 (30%), 45 (30%) and 30 (30%) regions of microsynteny between rainbow trout and medaka, stickleback, and zebrafish, respectively (additional file 5). Finally, our BlastN analyses identified 85 (medaka), 105 (stickleback), and 105 (zebrafish) unique paired BAC end hits in macrosynteny that were also detected using BlastX analyses (Table 3).

As expected, our analysis revealed moderate macro-synteny between rainbow trout and the three model species and even lower level of microsynteny likely due to chromosomal rearrangements that have occurred since the divergent of those species from a common teleost ancestor. The strongest decline in number of microsynteny regions was observed for zebrafish, which is indeed more distant from rainbow trout than the medaka and stickleback [42]. While the number of significant BES hits with BlastN was between 1.78 (zebrafish) to 2.7 (stickleback) times greater than those with BlastX hits, the difference in identifying microsynteny was even greater. The number of microsynteny regions identified with BlastN was 5.8 (zebrafish) to 9.3 (stickleback) times greater than BlastX. This can be explained by several factors including incomplete annotations of the model fish genomes and the presence of pseudogenes and conserved non-coding sequences that were not included in the peptide databases. BlastN also appears to be more accurate for estimating microsynteny as it provides the exact points of sequence matches on the chromosomes of the reference genomes (instead of the ORF boundaries for BlastX). However, it is also important to note that 17% (stickleback) to 33% (zebrafish) of the macro-synteny BAC paired ends identified by BlastX were not revealed by BlastN. This may be caused by non- or less-conserved peptides whose coding sequences are not under strong selection pressure and have evolved enough to escape detection as significant unique hits by BlastN.

The comparative genome analyses reported here provide a survey of conserved synteny between rainbow trout and three model fish species. The results of our analyses suggest that for many regions in the rainbow trout genome comparative mapping might serve as a useful genomic resource for identifying candidate genes in QTL detection studies. Nevertheless, further assessment of regions of conserved synteny by direct sequencing of full-length BAC clones and by evaluating gene content and orthology revealed that gene order, orientations, and gene length are highly variable across fish species (manuscript in preparation).

## Conclusions

In the present study, we sequenced and subsequently characterized more than half of BAC ends from the rainbow trout Swanson YY double haploid male 10X HindIII BAC library. These genomic sequences were used to generate the first rainbow trout specific repeat database containing 735 putative repeat elements. This database is useful for repeat masking in salmonid genomes. Approximately 59.5% of the BES database in base pairs was masked by this repeat database, providing for the first time an estimate of how much of the rainbow trout genome is composed of repetitive sequences. We detected 6,848 microsatellites in the BES dataset, of which 3,854 presented high quality flanking sequences with more than 50 bp in length. A subset of those were validated and used to construct the first rainbow trout integrated genetic-physical map [38]. The development of those new microsatellite markers will also serve to increase marker densities on current rainbow trout genetic maps and initiate *in silico *comparative mapping with species whose genomes have been fully sequenced. Paired BAC-ends were used to establish regions of microsynteny between trout and model fish species (zebrafish, medaka, and stickleback). The microsynteny analyses revealed low to intermediate genome homology between rainbow trout and the other fish species. Our findings suggest that due to chromosomal breakage and rearrangements that have occurred during fish genomes evolution, only closely related species like other salmonids will be useful for chromosome-wide and genome-wide comparative analyses with rainbow trout.

## Methods

### BAC culture and BAC-end sequencing

A 10X HindIII bacterial artificial chromosome (BAC) library from a Swanson YY male doubled haploid homozygous line was previously constructed [4]. More than half of this library (99,840 BAC clones) was used for BAC-end sequencing. BAC culture and sequencing reactions were conducted, as described previously [43]. Briefly, BAC DNA was extracted using a standard alkaline lysis protocol developed by Genoscope (Evry, France). Sequencing reactions were carried out in the Genoscope facility with T7 or Sp6 universal primers, using ABI kit version 3.1. Generated raw sequence files were subsequently processed using the PHRED software [44], vector and bacterial sequences were removed. Q20 values were achieved by setting the sequence quality PHRED score cut-off value to 20. All processed BES were submitted to the EMBL/EBI database with consecutive accession numbers of FQ482162-FQ658498 and are available through the web site of the INRA bioinformatics group (http://www.sigenae.org/troutBES).

### Identification of repetitive DNA elements

#### Complex DNA repetitive elements

RepeatModeler software was used for identifying repeat elements boundaries and for classifying the newly reconstructed repetitive sequence from the rainbow trout BES data (http://www.repeatmasker.org/RepeatModeler.html). A specific rainbow trout repeat database was constructed and was named INRA RT Rep1.0. This database was subsequently used as a custom file for masking BES sequences using RepeatMasker (http://www.repeatmasker.org/).

The Atlantic salmon repeat database was used (file Salmon Raw Repeat DB V1_6 available at http://web.uvic.ca/grasp) for comparison purposes.

#### Identification of microsatellites and simple sequence repeats

Microsatellites and other SSR motifs were identified using Tandem Repeat Finder (TRF) software [45]. We examined ten classes of SSRs by using a maximum period size of 10 with default settings. BES containing microsatellites were subsequently masked using RepeatMasker with INRA RT rep1.0 custom library file: BES harbouring SSRs with at least 50 bp flanking sequences were then selected and forward and reverse primers were designed using Primer3 software [46]. Microsatellites and corresponding flanking sequences were submitted to the GenBank STS database with consecutive accession numbers of GF100674-GF100698; GF107484-GF107651; GF107921-GF109647; GF110457-GF110594 and GF110820-GF110822.

### Assessment of microsatellites polymorphism

The polymorphism of 193 microsatellites markers was assessed by genotyping 10 parents from the National Center for Cool and Cold Water Aquaculture (NCCCWA) reference mapping panel [9]. Primers were optimized for amplification by varying annealing temperatures and MgCl_2 _concentrations. PCR amplifications were conducted in an MJ Research DNA Engine thermal cycler model PTC 200 (MJ Research, Waltham, MA) as previously described [47]. Three microliters of each PCR product were added to 20 μL of water, 1 μL of the diluted sample was added to 12.5 μL of loading mixture made up with 12 μL of HiDi formamide and 0.5 μL of Genscan 400 ROX internal size standard. Samples were denatured at 95 °C for 5 min and kept on ice until loading on an ABI 3730 DNA Analyzer (ABI, Foster City, CA). Output files were analyzed using GeneMapper version 3.7 (ABI, Foster City, CA),

### Assessment of regions of synteny with other fish genomes

#### Sequence homology searches and results filtration

Masked BES reads with more than 100 base pairs of contiguous non-repetitive sequences were analysed for sequence homology by BLASTN using ENSEMBL DNA databases for zebrafish (Danio_rerio.Zv9.61.dna_rm.toplevel.fa), stickleback (Gasterosteus_aculeatus.BROADS1.61.dna_rm.toplevel.fa), and medaka (Oryzias_latipes.MEDAKA1.61.dna_rm.toplevel.fa) and for gene content by BLASTX using the ENSEMBL non redundant protein databases for zebrafish (Danio_rerio.Zv9.61.pep.all.fa), stickleback (Gasterosteus_aculeatus.BROADS1.61.pep.all.fa), and medaka (Oryzias_latipes.MEDAKA1.61.pep.all.fa). BLASTN and BLASTX searches were carried out using an e-value cut off of 1e^-5 ^with following parameters (-m9 -r1 -q-1 -G4 -E2 -W9 -F "m D" -U for BLASTN). The BLAST search results were filtered to remove non specific sequences using the following filtration steps: (1) For each BES read with BLAST hit, results were filtered to keep only the hits with the minimal e-value score; (2) BES reads with at least two hits having the same minimal e-value were filtered to keep the hits with the highest HSPs (high-scoring segment pairs; calculated as the product of % identity multiplied by alignment length); and 3) keep only BES reads with single hits following filtration steps 1 and 2. For BLASTX the Ensembl protein IDs were renamed by their corresponding Ensembl gene IDs as each gene may encode several peptides due to alternative splicing.

#### Comparative synteny analysis

Identification of regions of conserved synteny between rainbow trout and model fish species were investigated using paired BAC-ends with unique hits. A region of microsynteny with the target genome was established if both BAC ends were mapped to the same chromosome with a space of 10 to 300 Kb between both ends and if they were properly oriented (tail-to-tail; the two ends in opposing orientation with 3' ends internal) [41,48,49]. In addition, we defined regions of macro-synteny as those in which the two paired BAC-end hits were mapped to the same chromosome of the model species.

## Authors' contributions

CG conceived the project. MB and CG supervised the project, and wrote the manuscript. MB and PD carried out bioinformatics analysis. YP performed the microsatellites genotyping analyses, provided 7,296 BAC clones for BAC end Sequencing and revised the manuscript draft. GG participated in and contributed ideas to the bioinformatics analysis. FG and PW carried out DNA extraction and DNA sequencing. EQ helped with interpretation of data analysis and revised the manuscript draft. All authors read, commented on and approved the final manuscript.

## Supplementary Material

Additional file 1 **INRA RT rep1.0**. The first Rainbow trout repeat database elements contains 735 putative elements in fasta format. Click here for file

Additional file 2 **PCR conditions, allele size range, number of alleles and GenBank accession numbers for tested microsatellites**. Duplicated markers are in bold. ND indicates that for these duplicated microsatellite it was not possible to determine the allele numbers. * indicates monomorphic or non informative microsatellites in the mapping panel. Abbreviations: Atp: Annealing temperature. Click here for file

Additional file 3 **An example of multiple gene hits corresponding to odorant receptor gene family (sheet 1) or protocadherin gene family (sheet 2) in zebrafish**. Click here for file

Additional file 4 **Summary of conserved microsyntenies identified by Blastn analysis with medaka (sheet 1), stickleback (sheet 2) and zebrafish (sheet 3)**. BAC, Forward and reverse hits indicate the orientation of the BES using the forward T7 and the reverse Sp6 sequencing primers, respectively. Span indicates the average distance between the two genes in the corresponding species. Click here for file

Additional file 5 **Summary of conserved microsyntenies identified by BlastX analysis with medaka (sheet 1), stickleback (sheet 2) and zebrafish (sheet 3) gene hits**. Putative identities of mate paired genes were provided as ENSEMBL protein ID. Forward and reverse hits indicate the orientation of the BES using the forward T7 and the reverse Sp6 sequencing primers, respectively. Span indicates the average distance between the two genes in the corresponding species. Click here for file
