# Supplementary material for: Analysis of BAC-end sequences in rainbow trout: Content characterization and assessment of synteny between trout and other fish genomes
Source: BMC Genomics. 2011 Jun 14;12:314. doi: 10.1186/1471-2164-12-314 (PMC3125269; doi:10.1186/1471-2164-12-314)
Supplement: Additional file 1 — INRA RT rep1.0. The first Rainbow trout repeat database elements contains 735 putative elements in fasta format. [file 1471-2164-12-314-S1.DOC]

>rnd-2_family-1#DNA/TcMar-Tc1 ( Recon Family Size = 1811 Final Multiple Alignment Size = 1054 )

AACAGGTACAAAAGTATCTATATCCACAGTAAAACGAGTCCTATATCGAC

ATAACCTGAAAGGCCGCTCAGCAAGGAAGAAGCCACTGCTCCAAAACCGC

CATAAAAAAGCCAGACTACGGTTTGCAACTGCACATGGGGACAAAGATCG

TACTTTTTGGAGAAACGTCCTCTGGTCTGATGAAACNAAAATNGAACTNT

TTGGCCATAATGNCNANCGTTACGTTTGGAGGAAAAAGGGGGAGGCTCGC

AAGCCGAAGAACACCATCCCNACCGTGAAGCACGGTGGTGGCAGCATCAT

GCTGTGGGGGTGCTTTTCTGCGGCAGGGACTGGTGCACTTCACAAAATNG

ATGGNANGATGAGGAGGAAAATTACGTGGATATATTGAAGCAACATCTCA

AGACATCAGTCAGGAAGTTAAAGCTGGGTCGCAAATGGGTCTTCCAACNG

GACAACGACCCAAGCACACNNCCAAGGCNGCGNCGGAGTGGCTTCGGGAC

AACAAAGTCTCAANGTNCTGGAGTGGCCATCACAGAGCCCGGACCTCAAC

CCNATCGAACATCTGTGGGNAGANCTGAAAATAGCGTGTGCGAGCAAGGA

GGCCTACAAACCTGACTCAGTTACACCGGCTCTGTCAGGAGGAATGGGCC

AAAATTCACCC

>rnd-2_family-84#DNA/TcMar-Tc1 ( Recon Family Size = 283 Final Multiple Alignment Size = 263 )

GAAGGCTACCCGAAACGTTTGACCCAAGTTAAACAATTTAAAGGCAATGC

TACCAAATACTAATTGAGTGTATGTAAACTTCTGACCCACTGGGAATGTG

ATGAAAGAAATAAAAGCTGAAATAAATCATTCTCTCTACTATTATTCTGA

CATTTCACATTCTTAAAATAAAGTGGTGATCCTAACTGACCTAAGACAGG

GAATTTTTACTAGGATTAAATGTCAGGAATTGTGAAAAACTGAGTTTAAA

TGTATTTGGCTAAGGTGTATGTAAACTTCCGACTTCAACTGT

>rnd-2_family-119#DNA/Tc1 ( Recon Family Size = 264 Final Multiple Alignment Size = 237 )

TCATACCCAAGAAGACTCGAGGCTGTAATCGCTGCCAAAGGTGCTTCAAC

AAAGTACTGAGTAAAGGGTCTGAATACTTATGTAAATGTGATATTTCAGT

TTTTTATTTTTAATACATTTGCAAAAATTTCTAAAAACCTGTTTTTGCTT

TGTCATTATGGGGTATTGTGTGTAGATTGATGAGGGGGAAAAAACGATTT

AATCNATTTTAGAATAAGGCTGTAACGTAACAAAATGTGGAAAAAGTCAA

GGGGTCTGAATACTTTCCGAATGC

>rnd-2_family-15#LINE/Rex1 ( Recon Family Size = 430 Final Multiple Alignment Size = 169 )

ACCCCCAGGTGGTGAGGGTAGGNAACAACACGTCCGCCACGCTGATCCTC

AACACNGGGGCCCCNCAGGGGTGCGTGCTCAGTCCCCTCCTGTACTCCCT

GTTCACCCACGACTGCGTGGCCANGCACGACTCCAACACCATCATCAAGT

TTGCNGACGACACAACAGTGGTAGGCCTGATCACCGACAACGACGAGACA

GCCTATAGGGAGGAGGTCAGAGACCTGGCAGTGTGGTGCCAGGACAACAA

CCTCTCCCTCAACGTGANCAAGACAAAGGAGATGATCGTGGACTACAGGA

AAAGGAGGACCGAGCACGCCCCCATTCNCATCGACGGGGCTGTAGTGGAG

CGGGTCGAGAGCTTCAAGTTCCTCGGCGTCCACATCACCGACAAACTAAC

ATGGTCCAANCACACCAAGACAGTCGTGAAGAGGGCACGACAACGCCTNT

TCCCCCTCAGGAGACTGAAAAGATTTGGCATGGGTCCTCAGATCCTCAAA

AGGTTCTACAGCTGCACCATCGAGAGCATCCTGACCGGTTGCATCACCGC

CTGGTACGGCAACTGCTCGGCCTCCGACCGCAAGGCGCTACAGAGGGTAG

TGCGTACGGCCCAGTACATC

>rnd-2_family-26#DNA/TcMar-Tc1 ( Recon Family Size = 173 Final Multiple Alignment Size = 168 )

CACAGTGCCTTGCGAAAGTATTCGGCCCCCTTGAACTTTGCGACCTTTTG

CCACATTTCAGGCTTCAAACATAAAGATATAAAACTGTATTTTTTTGTGA

AGAATCAACAACAAGTGGGACACAATCATGAAGTGGAACGACATTTATTG

GATATTTCAAACTTTTTTAACAAATCAAAAACTGAAAAATTGGGCGTGCA

AAATTATTCAGCCCCTTTACTTTCAGTGCAGCAAACTCTCTCCAGAAGTT

CAGTGAGGATCTCTGAATGATCCAATGTTGACCTAAATGACTAATGATGA

TAAATACAATCCACCTGTGTGTAATCAAGTCTCCGTATAAATGCACCTGC

ACTGTGATAGTCTCAGAGGTCCGTTAAAAGCGCAGAGAGCATCATGAAGA

ACAAGGAACACACCAGGCAGGTCCGAGATACTGTTGTGAAGAAGTTTAAA

GCCGGATTTGGATACAAAAAGATTTC

>rnd-2_family-2#Unknown ( Recon Family Size = 409 Final Multiple Alignment Size = 164 )

ATACACAAGGAATCTTTTTGGAAAAACTGAGCATCTGCTATCTAACTGAG

AGTATCCTCATTGAAAACATCAGAAGTTCTTCAAAGGTAAATGATTTTAT

TTGAAGGCTTTTATGTTTTTGTTAATGTTGCGTGCTGGATGCTAACGCTA

ATGCTAACGCTAAATGCTAACGCGAAATGCTAACGCTAGCTAGCATACTT

TTACACAAATGATTGTTTTCCTATGGTTGAGAAGCATATTTTGAAAATCT

GAGATGACAGTGTTGTTNACAAAAGGCTAAGCTTGAGAGCTGGCATATTT

ATTTCATTTCATTTGCGATTTTCATGAATAGTTAACGTTGCGTTATGGTA

ATGAGCTTGAGTCTGATAGATTTACACGATCCCGGATCCGGGGGTTTTTT

CGTAGCCAAACGTGACGCAGAAAACGGAGCGATTTGTCCTANACAAATAA

TCTTTCNGGAAAAACTGAACATTTGCTATCTAACTGAGAGTCTCCTCATT

GAAAACATCCGAAGTTCTTCAAAGGTAAATGATTTTATTTGAATGCTTTT

CTGTTTTTTTGTGTAAATGTTGCCNGCTGAATGCTAACGCTAAATGCTAC

GCTAGCCATCGATACTGTTACACAAATGCTTGTTTTGCNATGGTTGAGAA

GCATATTTTGAAAATCTGAGATGACAGTGTTGTTAACAAAAGGC

>rnd-2_family-11#Unknown ( Recon Family Size = 162 Final Multiple Alignment Size = 142 )

GCCATCCAGGACCTCTATACCAGGCGGTGTCAGAGGAAGGCCCTAAAAAT

TGTCAAAGACTCCAGCCACCCTAGTCATAGACTGTTCTCTCTGCTACCGC

ACGGCAAGCGGTACCGGAGCGCCAAGTCTAGGTCCAAGAGGCTTCTNAAC

AGCTTCTACCCCCAAGCCATAAGACTCCTGAACAGCTAATCAAATGGCTA

CCCGGACTATTTGCACCCCCCCCCCCCCTTTTACGCCGCTGCTACTCTCT

GTTTATTATCTATGCATAGTCACTTTAATANCTCTACCTACATGTACATA

TTACCTCGACTAACCGGTGCCCCCGCACATTGACTCTGTACCGGTACCCC

CTGTATATAGCCTCGCTATTGTTATTTTACTGCTGCTCTTTAATTATTTN

TTACTTTTATTTCTTAAACTGCATTGTTGGTTAAGGGCTCGTAAGTAAGC

ATTTCACNGTAAGGTCTACACCTGTTGTATTCGGCGCATGTGACAAATAA

AATTTGATTTGAT

>rnd-2_family-217#DNA/Tc1 ( Recon Family Size = 155 Final Multiple Alignment Size = 135 )

GTCATACCCAAGAAGACTCGAGGCTGTAATCGCTGCCAAAGGTGCTTCAA

CAAAGTACTGAGTAAAGGGTCTGAATACTTATGTAAATGTGATATTTCAT

TTTTTATTTTTNATAAATTTGCAAANATTTCTAAAAACCTGTTTTCGCTT

CGTCATTATGGGGTATTGTGTGTAGATTGATGAGAATATATATATTTTTA

TACATTTGCAAAAATTCCNAAAACCTGTTTTTGCTTTGTCATTATGGGGT

ATTGTGTGTAGATTGATGAGGGGGAAAAAACGATTTAATCCATTTTAGAA

TAAGGCTGTAACGTAACAAAATGTGGAAAAAGTCAAGGGGTCTGAATACT

TTCCGAATGCACATCTGTGTG

>rnd-2_family-240#DNA/TcMar-Tc1 ( Recon Family Size = 85 Final Multiple Alignment Size = 77 )

TTGGACTCTGGAGCAGTGGAAACGCGTTCTCTGGAGTGATGAATCACGCT

TCACCATCTGGCAGTCCGACGGACGAATCTGGGTTTGGCGGATGCCAGGA

GAACGCTACCTGCCCNAATGCATAGTGCCAACTGTAAAGTTTGGTGGAGG

AGGAATAATGGTCTGGGGCTGTTTTTCATGGTTCGGGCTAGGCCCCTTAG

TTCCAGTGAAGGGAAATCTTAACGCTACAGCATACAATGACATTCTAGAC

GATTCTGTGCTTCCAACTTTGTGGCAACAGTTTGGGGAAGGCCCTTTCCT

GTTTCAGCATGACAATGCCCCCGTGCACAAAGCGAGGTCCATACAGAAAT

GGTTTGTCGAGATCGGTGTGGAAGAACTTGACTGGCCTGCACAGAGCCCT

GACCTCAACCCCATCGAACACCTTTGGGATGAATTGGAACGCCGACTGCG

AGCCAGGCCTAATCGCCCAACATCAGTGCCCGACCTCACTAATGCTCTTG

TGGCTGAATGGAAGCAAGTCCCCGCAGCAATGTTCCAACATCTAGTGGAA

AGCCTTCCCAGAAGAGTGGAGGCTGTTATAGCAGCAAAGGGGGACCAACT

CCATATT

>rnd-2_family-85#DNA/TcMar-Tc1 ( Recon Family Size = 84 Final Multiple Alignment Size = 72 )

AAAGTATGTGGACACCCCTTCAAATTAGTGGATTCGGCTATTTCAGCCAC

ACCCGTTGCTGACAGGTGTATAAAATCGAGCACACAGCCATGCAATCTCC

ATAGACAAACATTGGCAGTAGAATGGCCTTACTGAAGAGCTCAGTGACTT

TCAACGTGGCACCGTCATAGGATGCCACCTTTCCAACAAGTCAGTTCGTC

AAATTTCTGCCCTGCTAGAGCTGCCCCGGTCAACTGTAAGTGCTGTTATT

GTGAAGTGGAAACGTCTAGGAGCAACAACGGCTCAGCCGCGAAGTGGTAG

GCCACACAAGCTCACAGAACGGGACCGCCGAGTGCTGAAGCGCGTAGCGC

GTAAAAATCGTCTGTCCTCGGTTGCAACACTCACTACCGAGTTCCAAACT

GCCTCTGGAAGCAACGTCAGCACAANAACTGTTCGTCGGGAGCTTCATGA

AATGGGTTTCCATGGCCGAGCAGCCGCACACAAGCCTAAGATCACCATGC

GCAATGCCAAGCGTCGGCTGGAGTGGTGT

>rnd-2_family-39#Unknown ( Recon Family Size = 147 Final Multiple Alignment Size = 61 )

CCATACAACTCTCCTTCCGTGGCCTCCAACTGCTCTTAAACGCAAGTAAA

ACTAAATGCATGCTCTTCAACCGATCGCTGCCCGCACCCGCCCGCCCGTC

CAGCATCACTACTCTGGACGGCTCTGACTTAGAATACGTGGACAACTACA

AATACTTAGGTGTCTGGTTAGACTGTAAACTCTCCTTCCAGACNCATATC

AAACATCTCCAATCCAAAGTTAAATCTAGAATCGGCTTCCTATTTCGCAA

CAAAGCNTCCTTCACTCACGCTGCCAAACATACCCTCGTAAAACTGACCA

TCCTACCGATCCTCGACTTCGGCGACGTCATTTACAAAATAGCCTCCAAT

ACCCTACTCAGCAAACTGGATGCAGTCTATCACAGTGCCATCCGTTTTGT

CACCAAAGCCCCATATACTACCCACCACTGCGACCTGTACGCTCTCGTCG

GCTGGCCCTCGCTNCATACTCGTCGCCAGACCCACTGGCTCCATGTCATC

TACAAGACCCTGCTAGGTAAAGTCCCCCCTTATCTCAGCTCGCTGGTCAC

CGTAGCATCNCCCACCCGTAGCACGCGCTCCGGCAGGTATATCTCNCTGG

TCATCCCCAAAGCCAACACCTCCTTTGGCCGCCTTTCNTTCCAGTTCTCT

GCTGCCAGTGA

>rnd-2_family-16#LINE/L2 ( Recon Family Size = 81 Final Multiple Alignment Size = 52 )

TGGACCCTCTCTTTCTAAAATTATCCGCCGAAATTGTCGCAACCCCTATT

ACTAGCCTGTTCAACCTCTCTTTCGTGTCGTCTGAGATTCCCAAAGATTG

GAAAGCNGCCGCGGTCATCCCCCTCTTCAAAGGGGGGGACACTCTTGACC

CAAACTGCTACAGACCTATATCTATCCTACCCTGCCTTTCTAAGGTCTTC

GAAAGCCAAGTCAACAAACAGATTACCGACCATTTCGAATCCCACCGTAC

CTTCTCCGCTATGCAATCTGGTTTCNGAGCTGGTCATGGGTGCACCTCAG

CCACGCTCAAGGTCCTAAACGATATCNTAACCGCCATCGATAAGAGACAN

TACTGTGCAGCCGTATTCATCGACCTGGCCAAGGCTTTCGACTCTGTCAA

TCACCGCATCCTCATCGGCAGACTCGACAGCCTTGGTTTCTCAAATGATT

GCCTCGCCTGGTTCACCAACTACTTCTCTGATAGAGTTCAGTGTGTCAAA

TCGGAGGGCCTGCTGTCCGGACCTCTGGCAGTCTCTATGGGGGTGCCACA

GGGTTCAATTCTCGGGCCGACTCTCTTCTCTGTATACATCAATGACGTCG

CTCTTGCTGCTGGTGAGTCTCTGATCCACCTCTACGCAGACGACACCATT

CTGTATACTTCCGGCCCTTCTTTGGACACTGTGTTAACAACCCTCC

>rnd-2_family-238#Unknown ( Recon Family Size = 61 Final Multiple Alignment Size = 52 )

GTCACGCCCTGACCTTAGTTCCTTTTTTATGTCTCTATTTTGGTTTGGTC

AGGGCGTGAGTTGTATTTCTATGTTTTGGCCTGGTATGGTTCTCAATCAG

AGGCAGCTGTCTATCGTTGTCTCTGATTGGGAACCATACTTAGGTAGCCT

GTTTTCCCACTGTGNGTTGTGGGTGGTTGTTTTCTGTNTCTGTGTTGTCG

CCAGACGGNACTGTTTCGGTTGTTCTTTCGTTGATTTTGTTTTGTAGTGT

TCAGTTNAGTTTAATAAAATGACGAACACTTACCACGCTGCGTTTTGGTC

CGATCCTTCCTACTCCTCCTCAGAAGAGGAGGAAAACCGTTAC

>rnd-2_family-65#LTR/Gypsy ( Recon Family Size = 52 Final Multiple Alignment Size = 49 )

CGACCGTTCCTTTTGTCGTTTGGACTGACCATAGGAACCTTGAGTACATC

CGTTCTGCCAAACGACTTAATGCGCGTCAGGCGCGTTGGGCGCTGTTTTT

CGCTCGTTTCGAGTTCGTGATTTCTTATCGTCCGGGCTCTAAGAACACCA

AGCCTGATGCTTTGTCTCGTCTCTTCAGTTCTTCAGTAGCCTCCACTGAC

CCCGAGGGGATTCTCCCTGAGGGGCGTGTTGTCGGGTTGACTGTCTGGGG

AATTGAGAGGCAGGTAAAGCAAGCGCTCACTCAAACTCCGTCGCCGCGCG

CTTGTCCTAGGAACCTTCTTTTCGTTCCCGTTCCTACTCGTCTGGCCGTT

CTTCAGTGGGCTCACTCTGCCAAGTTAGCCGGCCACCCTGGCGTTCGGGG

TACGCTTGCTTCCATTCGCCAGCGTTTTTGGTGGCCCACCCGGGAGCATG

ACACGCGTCGTTTCGTGGCTGCTTGTTCGGTCTGCGCGCAGACTAAGTCC

GGTAACTCTCCTCCTGCCGGCCGTCTCAGGCCGCTTCCTATTCCCTCTCG

ACCGTGGTCTCACATCGCCTTAGATTTTGTCACCGGACTGCCTTCGTCAG

CGGGGAAGACTGTTATTCTTACGGTTGTCGANAGGTTCTCTAAGGCGGCT

CATTTCATTCCCCTTG

>rnd-2_family-181#Unknown ( Recon Family Size = 55 Final Multiple Alignment Size = 47 )

TTTATATTTTTTTTATTACCCCTTTTTCTCCCCAATTTCGTGGTATCCAA

TTGTTAGTAGTTACTATCTTGTCTCATCGCTACAACTCCCGTACGGGCTC

GGGAGAGACGAAGGTCGAAAGCCATGCGTCCTCCGAAACACGACCCAACC

AAGCCGCACTGCTTCTTAACACAGCGCGCATCCAACCCGGAAGCCAGCCG

CACCAATGTGTCGGAGGAAACACCGTGCACCTGGCGACCTNGGTCAGCGC

GCACTGCGCCCGGCCCGCCACAGGAGTCGCTAGTGCGCGATGAGACAAGG

ACATCCCTACCGGCCAAACCCTCCCTAACCCGGACGACGCTAGGCCAATT

GTGCGTCGCCCCACGGACCTCCCGGTCGCGGCCGGCTGCGACAGAGCCTG

GGCGCGAACCCAGGGTCTCTGGTGGCACAGCA

>rnd-2_family-233#Unknown ( Recon Family Size = 46 Final Multiple Alignment Size = 42 )

GATTATTTGAATCAGCTGTGTAGTGCTAGGGCAAAAACCAAAACGTGCAC

CCCNTGGGGGCCCCAGGACCGAGTTTGGGAAAA

>rnd-2_family-57#LTR/Gypsy ( Recon Family Size = 42 Final Multiple Alignment Size = 41 )

CTTCTGCTAAAGAGACGGCACAAATCATCATCGAGAATGTTTTCAGAATT

CATGGCCTTCCGTCAGACGTCGTTTCGGACAGAGGTCCGCAATTCACGTC

TCAATTTTGGAGGGAGTTTTGCCGTTTGATTGGGGCTTCCGTCAGTCTCT

CTTCCGGCTTTCACCCCCAGTCTAACGGTCAAGCAGAACGGGCCAATCAG

ACTATTGGTCGCATCTTACGCAGTCTTTCTTTTCGCAACCCTGCGTCTTG

GTCAGAACAGCTCCCCTGGGCAGAATACGCCCACAACTCGCTTCCTTCGT

CTGCGACCGGGCTATCTCCTTTTCAGAGTAGCCTCGGGTACCAGCCTCCG

CTGTTCTCATCTCAGTTCGCCGAGTCCAGCGTCCCCTCCGCTCAGGCTTT

TGTCCAACGTTGCGAGCGCACCTGGAAGAGGGTCAGGTCTGCACTTTGCC

GTTATAGGGCGCAGACTGTGAGGGCTGCTAATAAGCGTAGAACTAAGAGT

CCTAGATATTGTCGCGGTCAGAGAGTTTGGCTCTCCACTCAGAACCTTCC

CCTTAAGACGGCTTCTCGCAAGTTGACCCCGCGGTTCATTGGTCCGTTCC

GTATTTCTCGGGTCATTAATCCTGTCGCAGTTCGACTTCTTCT

>rnd-2_family-25#DNA/TcMar-Tc1 ( Recon Family Size = 48 Final Multiple Alignment Size = 37 )

AATATGGGACCAAATACTAAACTTTTGACTACTTTAATACACATATAAGT

GAATTTGTCCCAATACTTTTGGTCCCCTAAAATGGGGGGACTATGTACAA

AAAGTGCTGTAATTTCTAAACGGTTCACCCGATATGGATGAAAATACCCT

CAAATTAAAGCTGACAGTCTGCACTTTAACCTCATAGTCATTGTATCATT

TCAAATCCAAAGTGCTGGAGTACAGAGCCAAAACAACAAAAAAATGTGTC

ACTGTCCCAATACT

>rnd-2_family-6#LINE/L2 ( Recon Family Size = 81 Final Multiple Alignment Size = 32 )

CATAATGAAAGAAAAGCANTGCAAGTTCGAATTTTGTAAAGTTAGTGTGG

GAGAGGTGGAAAAATTGTTGTCGATCAATAATGACAAACCTCCCGGCATT

GACAACTTAGATGGAAAGCTACTGAGGATGGTAGCTGACTCTATAGCCAC

TCCTATCTGTCATATTTTTAATCTGAGCCTAGAGGAAAGTNTTTGTCCTC

AGGCCTGGAGGGAAGCCAAAGTAATTCCGCTACCCAAGAGTGGTAAAGCG

GCCTTTACTGGTTCTAACAGCAGACCTATAANCTGGCTGCCGGCCCTTAG

CAAACTGTTGGAAAAAATTGTGTTTGACCAAATACAATGCTATTTCTCTG

TAAACAAATTAACAACAGACTTTCAGCATGCTTATAGGGAAGGGCACTCA

ACATGCACNGCACTGACACAAATGACTGATGATTGGCTGAAAGAAATTGA

TAATAAGAAGATTGTGGGAGCTGTNTTGTTAGACTTCAGTGCAGCTTTTG

ACATTATCGATCATAACCTGCTGTTGGAAAAACGTATGTGTTATGGCTTT

NCANCCTCTGCCATANCGTGGATTNAGAGCTACCTGTCTAACAGAACACA

GAGGGTTTT

>rnd-2_family-225#Unknown ( Recon Family Size = 31 Final Multiple Alignment Size = 30 )

TCCCCGGATGCCCCAAACAATCGGAAAGGGGATTCATCAGAGAAAATGAC

TTTACCCCAGTCCTCAGCAGTCCAATCCCTGTACCTTTTGCAGAATATCA

GTCTGTCCCTGATGTTTTTCCTGGAGAGAAGTGGCTTCTTTGCTGCCCTT

CTTGACACCAGGCCATCCTCCAAAAGTCTTCGCCTCACTGTGCGTGCAGA

TGCACTCACACCTGCCTGCTGCCATTCCTGAGCAAGCTCTGTACTGGTGG

TGCCCCGATCCCGCAGCTGAATCAACTTTAGGAGACGGTCCTGGCGCTTG

CTGGACTTTCTTGGGCGCCCTGAAGCCTTCTTCACAACAATTGAACCGCT

CTCCTTGAAGTTCTTGATGATCCGATAAATGGTTGATTTAGGTGCAATCT

TACTGGCAGCAATATCCTTGCCTGTGAAGCCCTTTTTGTGCAAAGCAATG

ATGACGGCACGTGTTTCCTTGCAGGTAACCATGGTTGACAGAGGAAGAAC

AATGATTCCAAGCACCACCCTCCTTTT

>rnd-2_family-9#Unknown ( Recon Family Size = 29 Final Multiple Alignment Size = 28 )

GTACATTATGCCTTGAATCTATTCTATCGCGCCCAGAAACCTCCTTTTAC

TCTCTGTTCCGAACGTACTAGACGACCAGTTCTTATAGCCTTTAGCCGTA

CCCTTATCCTACTCCTCCTCTGTTCCTCTGGTGATGTAGAGGTTAATCCA

GGCCCTGCAGCGCCTAGCTCCACTCCTATTCCCCAGGCGCTCTCATTTGT

TGACTTCTGTAACCGTAAAAGCCTTGGTTTCATGCATGTTAACATTAGAA

GCCTCCTCCCTAAGTTTGTTTTATTCACTGCTTTAGCACACTCTGCCAAC

CCGGATGTCCTAGCCGTGTCTGAATCCTGGCTTAGGAAGACCACCAAAAA

TCCTGAAATCTCCATCCCTAACTACAACATTTTCCGNCAAGATAGAACTG

CCAAAGGGGGCGGTGTTGCAATCTACTGCAGAGATAGCCTGCAGAGTTCT

GTCTTACTATCCAGGTCTGTACCCAAACAATTCGAGCTTCTACTTTTAAA

AATCCACCTTTCCAGAAACAAGTCTCTCACCGTTGCCGCTTGCTATAGAC

CACCCTCTGCCCCCAGCTGTGCCCTGGACACCATATGTGAACTGATTGCC

CCCCATCTATCTTCAGAGCTCGTGCTGCTAGGCGACCTAAACTGGGACAT

GCTTAACACCCCGGCCATCCTACAATC

>rnd-2_family-83#Unknown ( Recon Family Size = 25 Final Multiple Alignment Size = 24 )

ACCTTAGGAACATTCCTGTGGTGGAATTGTCCTTCTAAACCTAACGGTTC

CGCCGCTGTCACCCAAAAGCAAATGACGTTGTGGTGCAGGCTTCATTTTG

GGCCTACTTTTCTAATGGTCGCTGCGCCTAGACCGAGCGAGCTACGGTCA

AGCGGGATATCTCGTTGAACTCGGCACGGCCTAGGGATTATGGTAATGCC

ATTTCCTGCTCTCTGTGTCTTTAAGCACCGCACTTTTTCACTCCATCCTT

GCTGTGTGTGTGTGAGAGAGCTTTTCTTTGACATCTGTTGGGAGAAATGA

CTGATTTACAGTTCAGGAGGGTTACCTAGTCACACATATGAAGTTTTGGA

GAGATCTGACTTTTTTAACCCTTCGAAACAGCCCCTGNGACACCAATTAT

GGCACTTCCGGTTGGCACAGGAAGCTATAAGTAAACACATGTCTTCATTG

AGGTAGCCNCTTACAGAATCCTGAGTTTTAAGTCTTTANGTTAAGAATTG

ACTGATCTACACAGGGTTGAATGCAGTGATTTTCATAATGACAGGTTATG

CGTTTCAAAACACTTTTAGGGTGATTTTAACCACTTCCGGTTGGTCCAG

>rnd-2_family-404#DNA/TcMar-Tc1 ( Recon Family Size = 25 Final Multiple Alignment Size = 21 )

CCGGAGAAGACAAGGTGAGCGCTACCATCAGTCCTGTGTCATGCCAACAG

TAAAGCATCCTGAGACCATTCATGTGTGGGGTTGCTTCTCAGCCAAGGGA

GTGGGCTCACTCACAATTTTGCCTAAGAACACAGCCATGAATAAAGAATG

GTACCAACACATCCTCCGAGAGCAACTTCTCCCAACCATCCAGGAACAGT

TTGGTGACGAACAATGCCTTTTCCAGCATGATGGAGCACCTTGCCATAAG

GCAAAAGTGATAACTAAGTGGCTCGGGGAACAAAACATCGATATTTTGGG

TCCATGGCCAGGAAACTCCCCAGACCTTAATCCCATTGAGAACTTGTGGT

CAATCCTCAAGAGGCGGGTGGACAAACAAAANCCCACAAATTCTGACAAA

CTCCAAGCATTGATTATGCAAGAATGGGCTGCCATCAGTCAGGATGTGGC

CCAGAAGTTAATTGACAGCATGCCAGGGCGGATTGCAGAGGTCTTGAAAA

AGAAGGGTCAACACTGCAAATATTGACTCTTTGCATCAACTTCATGTAAT

TGTCAATAAAAGCCTTTGACACTTATGAAATGCTTGTAATTATACTTCAG

TATTCCATAGTAACATCTGACAAAAATATCTAAAGAC

>rnd-2_family-75#Unknown ( Recon Family Size = 24 Final Multiple Alignment Size = 21 )

GACTACAAACTTGTTGGATGCATTTGCCGTTTGTTTTTGGTTGTGTTTCA

GATTATTTTGTGCCCAATAGAAATGAATGGTAAATAATGTATTGTGTCAT

TTTGGAGTCACTTTTATTGTAAATAAGAATAGAATATGTTTCTAAACACT

TCTACATTAATGTGGATGCTACCATGATTACGGATAATCNTGAATGAATC

GTGAATAATGATGAGCGAGAAAGTTAGACCACAAATATCATACCCCCCAA

AACATGCTAACCTCCCCTGTTATTGTAAATGGTGAGAGGTTAGCATGTCT

TGGGGGTATGATATTTGTGCGTCTGTAACTTTCTCACTCATCATTATTCA

CGATTCATTCAGGATTATCCGTAATCATGGTAGCATCCACATTAATGTAG

AAGTGTTTAGAAACATATTCTATTCTTATTTACAATAAAAGTGACTCCAA

AATGACA

>rnd-2_family-123#DNA/TcMar-Tc1 ( Recon Family Size = 23 Final Multiple Alignment Size = 20 )

ATACAGTGGGGCAAAAAAGTATTTAGTCAGCCACCAATTGTGCAAGTTCT

CCCACTTAAAAAGATGAGAGAGGCCTGTAATTTTCATCATAGGTACACTT

CAACTATGACAGACAAAATGAGAAAAAAAAATCCAGAAAATCACATTGTA

GGATTTTTAATGAATTTATTTGCAAATTATGGTGGAAAATAAGTATTTGG

TCACCTACAAACAAGCAAGATTTCTGGCTCTCACAGACCTGTAACTTCTT

CTTTAAGAGGCTCCTCTGTCCTCCACTCGTTACCTGTATTAATGGCACCT

GTTTGAACTTGTTATCAGTATAAAAGACACCTGTCCACAACCTCAAACAG

TCACACTCCAAACTCCACTATGGCCAAGACCAAAGAGCTGTCAAAGGACA

CCAGAAACAAAATTGTAGACCTGCACCGGGCTGGGAAGGCTGAATCTGCA

ATAGGTAAGCAGCTTGGTTTGAAGAAATCAACTGTGGGAGCAATTATTAG

GAAATGGAAGACGTACAAGACCACTGATAATCTCCCTCGATCTGGGGCTC

CACGCAAGATCTCACCCCGTGGGGTC

>rnd-2_family-62#LINE/L2 ( Recon Family Size = 21 Final Multiple Alignment Size = 19 )

GAAAATTGGAACGGAAATGGCGCCACACCAAACTGGAAGTCTTCCGACTA

GCTTGGAAAGACAGTACCGTGCAGTATCGAAGAGCCCTTACTGCTGCTCG

ATCATCCTATTTTTCCAACTTAATTGAGGAAAATAAGAACAATCCGAAAT

TCCTTTTTGATACTGTCGCAAAGCTAACTAAAAAGCAGCATTCCCCAAGA

GAGGATGGCTTTCACTTCAGCAGTGATAAATTCATGAACTTCTTTGAGGA

AAAGATCATGATTATTAGAAAGCAAATTACGGACTCCTCTTTAAATCTGC

GTATTCCTTCAAAGCTCAGTTGTCCTGAGTCTGCACAACTCTGCCAGGAC

CTAGGATCAAGAGAGACGCTCAAGTGTTTTAGTACTATATCTCTTGACAC

AATGATGAAAATAATCATGGCCTCTAAACCTTCAAGCTGCATACTGGACC

CTATTCCAACTAAACTACTGAAAGAGCTGCTTCCTGTGCTTGGCCCTCCT

ATGTTGAACATAATAAACGGCTCTCTATCCACCGGATGTGTACCAAACTC

ACTAAAAGTGGCAGTAATAAAGCCTCTCTTGAAAAAGCCAAACCTTGACC

CAGAAAATATAAAAAAAC

>rnd-2_family-251#Unknown ( Recon Family Size = 20 Final Multiple Alignment Size = 19 )

TAGAGGTCGACCGATTATGATTTTTCAACGCCGATACCGATTATTGGAGG

ACCAAAAAAGCCGATACCGATTAATCGGCCGATTTTAAAAAANTAAATAT

TTTTTTTTATTTGTAATAATGACAATTACAACAATACTGAATGAACACTT

ATTTTAACTTAATATAATACATCAATAAAATCAATTTAGCCTCAAATAAA

TAATGAAACATGTTCAATTTGGTTTAAATAATGCAAAAACAAAGTGTTGG

AGAAGAAAGTAAAAGTGCAATATGTGCCATGTAAGAAAGCTAACGTTTAA

GTTCCTTGCTCAGAACATGAGAACATATGAAAGCTGGTGGTTCCTTTTAA

CATGAGTCTTCAATATTCCCAGGTAAGAAGTTTTAGGTTGTAGTTATTAT

AGGACTATTTCTCTCTATACCATTTGTATTTCATNNACCTTTGACTATTG

GATGTTCTTATAGGCACTTTAGTATTGCCAGCGTAACAGTATAGCTTCCG

TCCCTCTCCTCGCTCCTCCCTGGGCTCGAACCAGCAACACAACGACAATT

AGCGCGCGCCACCACTCGCTAGCCGTTTCACGTCGGTTACACAAACC

>rnd-2_family-314#SINE ( Recon Family Size = 23 Final Multiple Alignment Size = 18 )

TAAAATAACAGGGCTATATACAGGGGGTACCGGTACCGAGTCAATGTGCG

GGGGTACAGGTTAGTCGAGGTAATNTGTACATGTAGGTAGGGGTGAAGTG

ACTATGCAAGTTGGATAATAAACAGTGAGTAGCTGCAGTGTAAAAACAAA

TGGAGGGGGTCAATGTAAATAGTCCGGATGGCCATTTGATGAATTGTTCA

GTGGCAGGTAGCCTAGTGGTTAGAGCGTTGGGCCAGTAACCGAAAGGTTG

CNAGATCGAATCCCCGAGCTGACAAGGTAAAAATCTGTCGTTCTGCCCCT

GAACAAGGCAGTTAACCCACCGTTCCTAGGCCGTCATTGAAAATAAGAAT

NTGTTCTTAACTGACTTGCCTAGTTAAATAAAGGTTAAATAA

>rnd-2_family-290#Unknown ( Recon Family Size = 19 Final Multiple Alignment Size = 18 )

CAGTCTGTTATTCGAACTCAATCAGCATGACAGAGTGATCTCCAGCCTTG

TCCTCGTCAACACTCACACCTGTGTTAACGAGAGAATCACTGACATGATG

TCAGCTGGTCCTTTTGTGGCAGGGCTGAAATGCAGTGGAAATGTTTTTTG

GGGGATTCAGTTCATTTGCATGGCAAAGAGGGACTTTGCAATTCATCTGA

TCACTCTTCATAACATTCTGGAGTATATGCAAATTGCCATCATACAAACT

GAGGCAGCAGACTTTGTGAAAATTAATATGTGTCATTCTCAAAACTTTTG

GCCACGACTGTA

>rnd-2_family-77#LINE/L2 ( Recon Family Size = 20 Final Multiple Alignment Size = 17 )

TTTTCGTCTTTTGAGCTTCTAGTCATGAAATCTATGCAGCCTACTCAATC

ACTTTTTATAGCTACTGTTTACAGGCCTCCTGGGCCATATACAGCGTTCC

TCACTGAGTTCCCTGAATTCCTATCGGACCTTGTAGTCATAGCAGATAAT

ATTCTAATCTTTGGTGACTTTAATATTCACATGGAAAAGTCCACAGACCC

ACTCCAAAAGGCTTTCGGAGCCATCATCGACTCAGTGGGTTTTGTCCAAC

ATGTCTCCGGACCTACTCACTGTCACAGTCATACTCTGGACCTAGTTTTG

TCCCGTGGAATAAATGTTGTGGATCTTAATGTTTTTCCTCATAATCCTGG

ACTATCGGACCACCATTTTATTACGTTTGCAATCGCAACAAATAATCTGC

TCAGACCCCAACCAAGGANCATCAAAAGTCGTGCTATAAATTCNCAGACA

ACNCAAAGATTCCTTGATGCCCTTCCAGACTCCCTCTGCCTACCCAAGGA

CGTCAGAGGACAAAAATCAGTTAACCACCTAACTGAGGAACTCAATTTAA

CCTTGCGCAATACCCTAGATGCAGTTGCACCCCTAAAAACTAAAAACATT

TCTCATAAGAAACTAGCTCCCTGGTATACAGAAAATACCCGAGCTCTGAA

>rnd-2_family-199#Unknown ( Recon Family Size = 18 Final Multiple Alignment Size = 17 )

AAAATGTTAAAACATTTCTAGCCTAACAGGGTTGACGTGTTATGCTCGAC

CCGCTCAGTTTTCCACCACAAAACGCCAGAAAAAGAGTAGAACCAGCTCA

CCTGCTTTTACACTATGATTTGACTATTAGATGTTCAATGTTTCTTTTGA

AAAAAATATTTAAAAAGGAATAGTTTCACCATATTAAAACGAGAGTTCAG

TTCACGTAACAGGGTTGACCTTAAATGAGGGACAGACGTAAATGAATCAC

TAATCACATGAAATAAATAATAATCTTCAGAAATGACTTCGTCAAAGCAA

CAAAAAGGCAACAAATTTGTCAAAGCAACAAAATAACTAGGGCTTTACAA

TGATGGTGAAAACTTGGAGAAATGTTGGGGTTAAGTGGGTTAAAATCTTC

CTAGAAGTCACAGAGGATGCACGGAGGGACATGTCAAAATGCTGAATTTT

GGCACTTTAGCAAGTCTTTATTCATATAAAAAAATCTGACGTATTGAATT

TTCCATGTGGTCTATATTAAAGGGCACTTCATTAATATAACAGGCTTTTA

AAATTCAATATTGGTGCACAATTTCTACTTAAAATATCAAAGGGACGCNA

AAGGCACTCATTTCGTGGAACGACC

>rnd-2_family-154#LINE/L2 ( Recon Family Size = 20 Final Multiple Alignment Size = 17 )

TGCTNCTCCAGTTTCAACTGTTCTGCCTGCGGCTATGGAATCCTGACCTG

TTCACCNGACGTGCTACCNNTCCCAGACCTGCTGTTTTCAACTCTCTAGA

GACAGCAGGAGCGGTAGAGATACTCTNAATGATCGGCTATGAAAAGCCAA

CTGACATTTACTCCTGAGGTGCTGACCTGTTGCACCCTCTACAACCACTG

TGATTATTATTATTTGACCCTGCTGGTCATCTATGAACATTTGAACATCT

TGGCCATGTTCTGTTATAATCTCCACCCGGCACAGCCAGAAGAGGACTGG

CCACCCCTCATAGCCTGGTTCCTCTCTAGGTTTCTTCCTAGGTTCCGGCC

TTTCTAGGGAGTTTTTCCTAGCCACCGTGCTTCTACACCTGCATTGCTTG

CTGTTTGGGGTTTTAGGCTGGGTTTCTGTACAGCACTTTGAGATATCAGC

TGATGTAAGAAGGGCTTTATAAATACATTTGATTTGATT

>rnd-2_family-20#Unknown ( Recon Family Size = 17 Final Multiple Alignment Size = 16 )

AGCTAGCTCAAAGGACATTCAAAGTTCCTCCATAGAAGCCGCTCCTCCGT

AGGTATAATTCTGTGGGCCTAATTCAGATAATGCATGTCATAACAAGATG

CCCAGCGCTTCAAGCCTCCTCCACCCTCTCTCGCTCTCTCCTCCACCCGC

AAAATTTCAGTCGCATCTCGCGCCCTGCACTTGTCCGTCCACACGCATGT

ANACAACTACCTTGCCCGCTCTATCCGCACTGATTGGTGAAGTAATTTAA

TGTCAGCTAAATGTAAAAAACTATCGATTTCAGAGGTTTAAAAAGGAACA

GAAAGGAACGATATAAACCGGTACTTTTTGNGGGTTCGAACCGGTTCAGA

ACTTTATTTTGCTGGTCGGAACAGTGGAACGGAACGAAAAAAATATGGTT

CCGTTCAGAACGAAACGATTGGAAAATAATTTCGGTTCCAACCCCTGGT

>rnd-2_family-86#Unknown ( Recon Family Size = 19 Final Multiple Alignment Size = 16 )

AGTAAAGCAATGAAAACAAGCAAGCATCCCAGAAAAGTGCTCAAAATAGC

CCACGTTAACATATGTAGCTTAAGAAACAAGGTTCATGAAATCAATAATT

TGCTAGTAACAGATGACATTCATATTCTGACTATCTCTGAAACTCACTTA

GATAATACCTTTGATGATACAGTGGTAGCAATACANGGTTATAACATCTA

CAGAAAAGACAGAAATGCCAACGGNGGAGGTGTTGCTGTNTATATTCAGA

ACCACATTCCTGTAAAGCTTAGAGAGGATCTCATGTTAAATGCTGTTGAA

GTAATATGGCTACAGGTTCATCTGCCTCACCTAAAGCCCGTTCTTGCGGG

AAGCTGCTATAGACCACCAAGTGCTAACAGTCAGTATCTGGATAACTGCG

TGAAATGCTTGATAATGTGTGCGATATCAACAGAGAGGTATATTTTCTGG

ATGATTTNAACATTGACTGGCTTTGAACAAGCTGCCCACTCAAGAGGAAG

CTTCAAACTGTAACCAGCGCCTGTAATCTGGTTCAGGTTATCAGTCAACC

TACCAGGGTGTTTACAAACAGCACAGGAACGAGATCATCNACATGTATTG

ATCACATCTTTACTAATGCTGCAGAACTTTGCTCTAAAGCNGTATCCGNA

CCCATCGGATGCAGTGATCACAATATAGTGGCTATATCT

>rnd-2_family-438#Unknown ( Recon Family Size = 16 Final Multiple Alignment Size = 15 )

CTACAAACATGCANTGCTAGCTAGCTGTAGCTTATGCTTTCAGTACTAGA

TTCATTTCTCTGATCCTTTGATTGGGTGGACAACATGTCAGTTCATGCTG

CAAGAGCTCTGATAGGTTGGAGGACGTCCTCCGGAAGTTGTCATAATTAC

TGTGTAAGTCTATGGAAGGGGGTGAGAACCACGAGCCTCCTAGGTTTTGT

ATTGAAGTCAATGTACCCAGAGGAGGACGGAAGCTAGCTGTCCTCCGGCT

ACACCACGGTGCTACCCTACAGAGTGCTGCTGAGGCTACTGTAGACCTTC

ATTGCAAAACAGTGTGTTTTAATCAATTATTTGGTGACGTGAAT

>rnd-2_family-110#Unknown ( Recon Family Size = 18 Final Multiple Alignment Size = 15 )

TGGGTGCGTTCGTAAATTCAATCTGGAGTGCCAGAGTGCGCTCGTAAATT

CTGAGCGTTGCCAGATTGTCCGTTCGTAAATTCAGAGCGTTTCGCTCTCG

GAGCGCACACCGGACGCTCTGGCCGAGGAGTAGGGTTGATCCGAGCGTTC

CGACCTCACAACAGCAGTCAAGCACCCAAGCTAACGTTGGCTAGCTTGCT

AGCTACTTCCAGACACAAATGAGAGAACAGCTCACTCTGACCATTTTACT

CACCCTAGCAGAGCTGGTTAGGCTGTTTTCATGTTATCCAGAGCGTTGGT

GACTGCAACTGTGCTGCTGGCAACAATTTAATTACGCTTTTTTGCAGACG

TTTACTGACACCGGCCATATTCAACGGGTGTTGAGCGTTCGTAAATTCGT

CAGTTATTCTGCGCTCTGGCACACTCAGACGAGAGTGCTCTGAAATCGGA

GTAGATAGCCAGAGCGAATTTACGAACGCGCCCGAAT

>rnd-2_family-131#Simple_repeat ( Recon Family Size = 22 Final Multiple Alignment Size = 15 )

TTTATTNCTGACAGAGGACATACCTCTGCGTCCCAAACGGCACCCTATTC

CCTTTGGGCCCTGGCCAAAAGTAGCGCACTATATAGGGAATAGGGCCCTG

GTCAAAAGTAGTGCACTATATAGGGAATAGGGCCCTGGTCAAAAGTAGTG

CACTATANAGGGAATAGGGCCCTGGTCAAAAGTAGTGCACTATATAGGGA

ATAGGGTGCCATTTGGGACGC

>rnd-2_family-450#SINE/Deu ( Recon Family Size = 16 Final Multiple Alignment Size = 15 )

AATTTCAACTCTTTGTCCGTTTTTTCCCACAAGGCTGAAATATGTGCCCT

CGCTTTGAAATAAGTAAGCAAGGTTAGTTTGTATTTCATCCAACAATCTG

TGCTACAAATTATGGCTACAGTATATGCAATTTCATCCACTTTATGAGAT

TGCCTTTCGCTTACGGCCACACCGGCCTGAGTACGCCTGATCTCGTCCGA

TCTCGGAAGCTAAGCAGGGTCGGGCCTGGTTAGTACTTGGATGGGAGACC

GCCTGGGAATACCAGGTGCTGTAAGCTTTTTGTCACTGCCTTGTGGAATT

TCAACTCTTTGTCCGTTTTTTCCCACAAGGCTGAAATATGTGCCCTCGCT

TTGAAATAAGTAAGCAAGGTTAGTTTGTATTTCATCCAACAATCTGTGCT

ACAAATTATGGCTACAGTATATGCAATTTCNTCCACTTTTTGAGATTGCC

TTTCGCTTACGGCCACACCGGCCTGAGTACGCCTGATCTCGTCCGATCTC

GGAAGCTAAGCAGGGTCGGGCCTGGTTAGTACTTGGATGGGAGACCGCCT

GGGAATACCAGGTGCT

>rnd-3_family-4#DNA/TcMar-Tc1 ( Recon Family Size = 4709 Final Multiple Alignment Size = 1243 )

AAACATCCCAAGGAGCACTGTGCAAGCGATAATATTGAAATGGAAGGAGT

ATCAGACCACTGCAAATCTACCAAGACCTGGCCGTCCCTCTAAACTTTCA

GCTCATACAAGGAGAAGACTGATCAGAGATGCGGCCAAGAGGCCCATGAT

CACTCTGGATGAACTGCAGAGATCTACAGCTGAGGTGGGAGAACCTTCCA

GAAGGACAACCATCTCTGCAGCACTCCACCAATCAGGCCTTTATGGTAGA

GTGGCCAGACGGAAGCCACTCCTCAGTAAAAGGCACATGACAGCCCGCTT

GGAGTTTGCCAAAAGGCACCTAAAGGACTCTCAGACCATGAGAAACAAGA

TTCTCTGGTCTGATGAAACCAAGATTGAACTCTTTGGCCTGAATGCCAAG

CGTCACGTCTGGAGGAAAANCAACACATCACTGAGTACCACTCTCCATAT

TTTCAAGCATGGTGGTGGCTGCATCATGTTATGGGTATGCTTGTCATCGG

CAGGGACTGGGAGATNGTCAGGATCGAGGGAAAGATGAACGGAGCNAAGC

ACAGGACCATTCTGGAAGAAAACCTGATGGAGTCTGCAAAAGACCTGAGA

CTGGGACGGAGATTCGCCTTCCAACAGGACAATGACCCAAAACACAAGGC

CAAATCTACGCTGGAGTNGCTTACCAAGAAT

>rnd-3_family-12#DNA/TcMar-Tc1 ( Recon Family Size = 493 Final Multiple Alignment Size = 488 )

GATAGGCTAATTGACATNATTTGAGTCAATTGGAGGTGTACCTGTGGATG

TATTTCAAGGCCTACCTTCAAACTCAGTGCCTCTTTGCTTGACATCATGG

GAAAATCAAAAGAAATCAGCCAAGACCTCAGAAAAAATTGTAGACCTCCA

CAAGTCTGGTTCATCCTTGGGAGCAATTTCCAAACGCCTGAAGGTACCAC

GTTCATCTGTACAAACAATAGTACGCAAGTATAAACACCATGGGACCACG

CAGCCGTCATACCGCTCAGGAAGGAGACGCGTTCTGTCTCCTAGAGATGA

ACGTACTTTGGTGCGAAAAGTGCAAATCAATCCCAGAACAACAGCAAAGG

ACCTTGTGAAGATGCTGGAG

>rnd-3_family-34#DNA/TcMar-Tc1 ( Recon Family Size = 262 Final Multiple Alignment Size = 249 )

TGATAGGCTAATTGACATNATTTGAGTCAATTGGAGGTGTACCTGTGGAT

GTATTTCAAGGCCTACCTTCAAACTCAGTGCCTCTTTGCTTGACATCATG

GGAAAATCAAAAGAAATCAGCCAAGACCTCAGAAAAAAAATTGTAGACCT

CCACAAGTCTGGTTCATCCTTGGGAGCAATTTCCAAACGCCTGAAGGTAC

CACGTTCATCTGTACAAACAATAGTACGCAAGTATAAACACCATGGGACC

ACGCAGCCGTCATACCGCTCAGGAAGGAGACGCGTTCTGTCTCCTAGAGA

TGAACGTACTTTGGTGCGAAAAGTGCAAATCAATCCCAGAACAACAGCAA

AGGACCTTGTGAAGATGCTGGAG

>rnd-3_family-24#Unknown ( Recon Family Size = 315 Final Multiple Alignment Size = 167 )

TTTTTACATCCCTGTTAGTGAGCATTTCTCCTTTGCCAAGATAATCCATC

CACCTGACAGGTGTGGCATATCAAGAAGCTGATTAAACAGCATGATCATT

ACACAGGTGCACCTTGTGCTGGGGACAATAAAAGGCCACTCTAAAATGTG

CAGTTTTGTCACACAACACAATGCCACAGATGTCTCAAGTTTTGAGGGAG

CGTGCAATTGGCATGCTGACTGCAGGAATGTCCACCAGAGCTGTTGCCAG

AGAATTNAATGTTAATTTCTCTACCATAAGCCGCCTCCAACGTCGTTTTA

GAGAATTTGGCAGTACGTCCAACCGGCCTCACAACCGCAGACCACGTGTA

ACCACGCCAGCCCAGGACCTCCACATCCGGCTTCTTCACCTGCGGGATCG

TCTGAGACCAGCCACCCGGACAGCTGATGAAACTGAGGAGTATTTCTGTC

TGNAAAAACTCATTCTGATTGGCTGGNCTCGTCTGTGCCCACCCACGGAT

GCGCATCTGTATTCCCAGTCATGTGAAATCCATAGATTAGGGCCTAATTT

ATTAAATCCATAGATTAGGGCCTAATGAATTTATTTCAATTGACTGATTT

CCTTATATGAACTGT

>rnd-3_family-651#DNA/TcMar-Tc1 ( Recon Family Size = 139 Final Multiple Alignment Size = 136 )

AGTGTACAAAACATTAGGAACACCTGCTCTTTCCATGACATAGACTGACC

AGGTGAATCCAGGTGAAAGCTATGATCCCTTATTGATGTCACTTGTTAAA

TCCACTTCAATCAGTGTAGATGAAGGGGAGGAGACAGGTTAAAGAAGGAT

TTTTAAGCCTTGAGACAATTGAGACATGGATTGTGTATGTGTGCCATTCA

GAGGGTGAATGGGCAAGACAAAATATTTAAGTGCCTTTGAACGGGGTATG

GTAGTAGGTGCCAGGCGCACCGGTTTGAGTGTGTCAAGAACTGCAACGCT

GCTGGGTTTTTCACGCTCAACAGTTTCCCGTGTGTATCAAGAATGGTCCA

CCACCCAAAGGACATCCAGCCAACTTGACACAACTGTGGGAAGCATTGGA

GTCAACATGGGCCAGCATCCCTGTGGAACGCTTTCGACACCTTGTAGAGT

CCATGCCCCGACGAATTGAGGCTGTTCTGAGGGCAAAAGGGGTGCAACTC

AATATTAGGAAGGTGTTCCTAATGTTTTGTACACTCAGT

>rnd-3_family-78#DNA/TcMar-Tc1 ( Recon Family Size = 217 Final Multiple Alignment Size = 134 )

CGTCCCTACGGTGAAGCATGGTGGTGGAAGCACGGGGGTGGCAGCATCAT

GTTGTGGGGGTGCTTTGCTGCGGGAGGGACTGGTGCACTNGTCAGAATNG

ANGGAATGATGAACGGAGCAAAGTACGGGGAGATCCTTGANGAAAACCTG

CTCCAGNNCGCTCAGGACCTNAGACTGGGNCGGAGGTTCGCCTTCCAAAN

GGACAATGACCCCAAGCATACTTCCAAAGTTGTGGCAAAGTGGCTTAAGG

ACAAGTCAANGTNTTGGAGTGGCCATNNCAAAGCCCGGACCTCAATCCGA

TCGAAAATCTNTGGAGAGACCTGAAAATAGCTGTGCAGCGACGCTCCCCA

TCCAACCTGACGGAGCTTGAGAGGATCTGCAGAGAAGAATGGGAGAAACT

CCCCAAATACAGGTG

>rnd-3_family-128#DNA/TcMar-Tc1 ( Recon Family Size = 160 Final Multiple Alignment Size = 122 )

AAACATCCCAAGGAGCNNNNNNNNNNNNNNNNNNNNNNNNNNNNNNNNNN

NNNNNNNNNNNNNNNNNNNNNNNNNNNNNNNCCGTCCCTCTAAACTTTCA

GCTCATACAAGGAGAAGACTGATCAGAGATGCAGCCAAGAGGCCCATGAT

CACTCTGGATGAACTGCAGAGATCTACAGCTGAGGTGGGAGTATCTGTCC

ATAGGACCACTTTAAGCCGTACACTCCACAGAGCTGGGCTTTACGGAAGA

GTGGCCAGAAAAAAGCCATTGCTTAA

>rnd-3_family-198#LINE ( Recon Family Size = 138 Final Multiple Alignment Size = 111 )

TTGAACGAATTGCAAAAATCGCTGAAGCTGGAGACTTNTATCTCCCTCAC

TAACTTTAAGCATCAGCTGTCAGAGCAGCTNACCGATCGCTGCACCTGTA

CATAGCCCATCTGTAAATAGCCCACCCAACTACCTACCTCATCCCCATAC

TGTTTTTATTTTTCTGCTCTTTTGCACCCCAGTATCTCTACTTGCACATC

ATCATCTGCACATCTATCACTCCAGTGTTNATTTGCTAAATTGTAATTAC

TTCGCCACTATGGCCTATTTATTGCCTTACCTCCCTTATCTCACCTCATT

TGCACACACTGTATATAGATTTTTCTACTGTGTTATTGACTGTACGTTTG

TTTACTCCATGTGTAACTCTGTGTTGTTGTNTGTGTCGCACTGCTTTGCT

TTATCTTGGCCAGGTCGCAGTTGTAAATGAGAACT

>rnd-3_family-72#DNA/TcMar-Tc1 ( Recon Family Size = 139 Final Multiple Alignment Size = 110 )

CATCTCTGCAGCACTCCACCAATCAGGCCTTTATGGTAGAGTGGCCAGAC

GGAAGCCACTCCTCAGTAAAAGGCACATGACAGCCCGCTTGGAGTTTGCC

AAAAGGCACCTAAAGGACTCTCAGACCATGAGAAACAAGATTCTCTGGTC

TGATGAAACCAAGATTGAACTCTTTGGCCTGAATGCCAAGCGTCACGTCT

GGAGGAAACCT

>rnd-3_family-71#Unknown ( Recon Family Size = 116 Final Multiple Alignment Size = 90 )

GCACTGAGATGCAGTGCCTTAGACCGCTGCGCCACTCGGG

>rnd-3_family-193#Unknown ( Recon Family Size = 137 Final Multiple Alignment Size = 87 )

GGGGTCACTTAGAAATGTCCTTGTTTTTGAAAGAAAAGCACATTTTTTGT

CCATTAAAATAACATCAAATTGATCAGAAATACAGTGTAGACATTGTTAA

TGTTGTAAATGACTATTGTAGCTGGAAACGGCTGATTTTTAATGGAATAT

CTACATAGGCGTACAGAGGCCCATTATCAGCAACCATCACTCCTGTGTTC

CAATGGCACGTTGTGTTAGCTAATCCAAGTTTATCATTTTAAAAGGCTAA

TTGATCATTAGAAAACCCTTTTGCAATTATGTTAGCACAGCTGAAAACTG

TTGTNCTGATTAAAGAAGCAATAAAACTGGCCTTCTTTAGACTAGTTGAG

TATCTGGAGCATCAGCATTTGTGGGTTCGATTACAGGCTCAAAATGGCCA

GAAACAAAGAACTTTCTTCTGAAACTCGTCAGTCTATTCTTGTTCTGAGA

AATGAAGGCTATTCCATGCGAGAAATTGCCAAGAAACTGAAGATCTCGTA

CAACGCTGTGTACTACTCCCTTCACAGAACAGCGCAAACTGGC

>rnd-3_family-216#Unknown ( Recon Family Size = 102 Final Multiple Alignment Size = 79 )

TATAAATGCCTCATGAGCTTAGTTCAACTGTCGTACCCCATCAGAACCCA

AAATATAAGCTTGTTTTACTCCAATGTTTGTAAACAANGTAAATGTAAAC

AAACACTGTATAGCCTCAAAACATGGTTAAAACTATAATTTTGATATCAT

GGATGGTCAGTCCTTGCATCCATAGCTCTGTCTATGAATTTGAGAGTGGT

TACATTTCTCCAGCCCCATCCCTCAGCTTTTTACCGAAACAGNGGCGGGG

NGACCGCTTTGTTATTGTTTCAACTGCGGATTTGCCCTTT

>rnd-3_family-64#Unknown ( Recon Family Size = 98 Final Multiple Alignment Size = 75 )

TCGATTTATCGGTGGTGACAGTGTTTCCTAGCCTCAGTGCAGTGGGCAGC

TGGGAGGAGGTGCTCTTATTCTCCATGGACTTTACAGTGTCCCAGAACTT

TTTGGAGTTNGTGCTACAGGATGCGAATTTCTGCTTGAAAAAGCTAGCCT

TGGCTTTCCTAACTGCCTGTGTATATTGGTTCCTAACTTCCCTGAAAAGT

TGCATATCACGGGGGCTATTCGATGCTAATGCAGAACGCCACAGGATGTT

TTTGTGCTGGTCAAGGGCAGTCAGGTCTGGAGTGAACCAAGGGCTATATC

TGTTCCTGGTTCTACATTTTTTGAATGGGGCATGCTTATTTAAGATGGTG

AGGAAGGCACTTTTAAAGAATAACCAGGCATCCTCTACTGACGGGATGAG

GTCAATATCCTTCCAGGATACCCCGGCCAGGTCGATTAGAAAGGCCTGCT

CGCTGAAGTGTTTTAGGGAGCGTTTGACAGTGATGAGGGGTGGTCGTTTG

ACCGCGGACCCATTACGGATGCAGGCAATGAGGCAGTGATCGCTGAGATC

TTGGTTGAAAACAGCAGAGGTGTATTTGGAGGGCGAGTTGGTTAGGATGA

TATCTATGAGGGTGCCCGTGTTTACGGATTTGGGGTTGTACCTGGTAGGT

TCATTGATAATTTGTGTGAGATTGA

>rnd-3_family-145#DNA/Tc1 ( Recon Family Size = 76 Final Multiple Alignment Size = 72 )

ACAGTTGAAGTCGGAAGTTTACATACACTTAGGTTGGAGTCATTAAAACT

CGTTTTTCAACCACTCCACAAATTTCTTGTTAACAAACTATAGTTTTGGC

AAGTCGGTTAGGACATCTACTTTGTGCATGACACAAGTAATTTTTCCAAC

AATTGTTTACAGACAGATTATTTCACTTATAATTCACTGTATCACAATTC

CAGTGGGTCAGAAGTTTACATACACTAAGTTGACTGTGCCTTTAAACAGC

TTGGAAAATTCCAGAAAATGATGTCATGGCTTTA

>rnd-3_family-130#Unknown ( Recon Family Size = 66 Final Multiple Alignment Size = 65 )

GAATCGACACAGGTAGACCTCATAGTGGCCTGATTGGACTGTCACCGAAG

ACAGGTTCATACGGCATTCATAACCCACATAGGCTTCAGGTTGAATTTAG

GGGTGCAGGCAATGTATTCCTATGGGGAGAGATGTCATTGTAATCTGTGA

GATCAAAAAACCCTGTTTTACTGTNAAGGGTTAATGCCACACGGTCAAGG

TTAGGCTTGCACGGATCGGGAGGNNNNNNNNNNNNNNNNNNNNNNNNNNN

NNNNNNNNNNNNNNNNNNNNNNNNNNNNNNNNNNNNNNNNNNNNNNNNNN

NNNNNNNNNNNNNNNNNNNNNNNNNNNNNNNNNNNNNNNNNNNNNNNNNN

NNNNNNNNNNNNNNNNNNNNNNNNNNNNNNNNNNNNNNNNNNNNNNNNNN

NNNNNNNNNNNNNNNNNNNNNNNNNNNTTTTGTTGATTTCAGAATGCCAT

TTTGGTGATGGTCCCTCTCCGTTTAAACATATTGCAAATGTACAAAAGTC

AACTAGCAAGTCAGTGTCCATCAGTCAATTAGATGTCATTATGACACCAA

ACGGATATCAATTGACACCAAACCCACTTTTTCCTACTCATTTAGNAGCC

AACTATCACATATG

>rnd-3_family-46#DNA/TcMar-Tc1 ( Recon Family Size = 78 Final Multiple Alignment Size = 60 )

ATACCCCAAGAGACTTGCAGCTGTAATCGCTGCAAAAGGTGGCTCTACAA

AGTNTTCCGTTTTTTGTCTTATTTCTTGTTTGTTTCACAATAAAAAATAT

TTTGCATCTTCAAAGTGGTAGGCATGTTGTGTAAATCAAATGATACAAAC

CCCCCCAAAATCTATTTTAATTCCAGGTTGTAAGGCAACAAAATAGGAAA

AATGCCAAGGGGGTGAATACTTTCGCAAGCCACTG

>rnd-3_family-200#DNA/TcMar-Tc1 ( Recon Family Size = 61 Final Multiple Alignment Size = 60 )

GCCCATGATTTTGGAATGAGATGTTCGACGAGCAGGTGTCCACATACTTT

TGGTCATGTA

>rnd-3_family-117#SINE? ( Recon Family Size = 60 Final Multiple Alignment Size = 56 )

AGTATGCAAACCCCTGAGGGAGAAATCTGTGCTCGATTGCCAGGCCAACA

GAAATCTCAACTGACTCTTGAAAAATCTGACTTTGCTTTCATTTTCAGTT

TGGACATTTACTCATTCTCAGAAGACTTTGGTGAATCCATTGTAGATGTC

CAGTGTGTGAGGTGATTTACTTTGTGCAAGGCTTAAGTTGTTAAATGGGA

AACGAGTAACCAACTAGTCATCGACAACTGTCTTGAGTTTGGCATTTGTG

ACAATGTCCTGGGACATCCCACAAACGCGTCACCTTCTAACTACGAGTGT

GTGGCCTGTGAGTGGTATAGAGCGAAATCGATTACATGTACGTTTTCGTG

AGAGTCTCAGCTTTCCATAGAGGGGTCACAGTAGTTTCTAGCTCTAACCA

TTCAGTCTATACAGATGTTTTTGTTAGAAGTGTTAAAGCCCAACACTGCA

AACGTATAGTTAGTACCAATGCTCCACGTTTTTTCAAACGTCCACCACAC

CAGCTCCTCGTTAGTNCAGTGGTGAGNATCCCCGCCTGCCACGCGGGAGG

CCCGGGTTCGATTCCCCGACGGGGAGAAAGGACTGCTTTTTTTTTAGGGT

GAACAATGTAGCTGAGCCCACTATGCGAAAAATATGCTTGGAGAT

>rnd-3_family-124#Unknown ( Recon Family Size = 56 Final Multiple Alignment Size = 52 )

TCACGTTTCAGGTAAGACCCAGATGCAGACAGTGTCGAAGTAACAAATGT

TTATTACAACAACAGGGGCAGGCAAANGACAGGTCGAGGGCAGGCAGGGG

TCAGTAATCCAGAGCAGAGTCCAAAAGGTACAGGACGGCAGGCAGGCTCG

GGGTCAGGGCAGGCAGGGGTCGGTAATCCGGAGGCGGGGCAGGGTACAGG

ACGGCAGGCTCAGGGTCAGGGCAGGCAGAACGGTCAAAACCGGGAAGGAC

TAGAAAACAGGAGCAAGGAAAAACAGGAGCACGGGAACAAACGCTGGTAG

GCTTGACAAAACAAGACGAACTGGCAACAGACAGACAGAGAACACAGGTA

TAAATACACAGGGGATAATGGGGAAGATGGGCGACACCTGGAGGGGGGTG

GAGACAAGCACAAAGACAGGTGAAACAGATCAGGGTGTGAC

>rnd-3_family-122#Unknown ( Recon Family Size = 50 Final Multiple Alignment Size = 49 )

GGCTGCTGAGGGGAGGACGGCTCATAATAATGGCTGGAACGGAGCGAATG

GAATGGCATCAAACACATGGAAACCATGTGTTTGATGTGTTTGATACCAT

TCCACTNATTCCGCTCCAGCCATTACCACGAGCCCGTCCTCCCCAATTAA

GGTGCCACCAACCT

>rnd-3_family-55#LINE/L2 ( Recon Family Size = 68 Final Multiple Alignment Size = 48 )

CTCTAATGTCAAACATGTAAAGTGTGGTGTACCGCAGGGCAGCTCTCTAG

GCCCTCTACTCTTTTCTATTTTTACCAATGACCTGCCACTGGCATTGAGC

AAAGCATGTGTGTCCATGTATGCTGATGATTCAACCATATACGCGTCAGC

AACCACAGCTANTGAAGTCACTGAAACNCTTAACAAAGAGTTGCAGTCNG

TTTCGGAATGGGTGGCCAGNAATAAGCTGGTCCTGAACATCTCTAAAACT

AAGAGCATTGTATTTGGTACAAATCATTCCCTAAGTTCTAGACCTCAGCT

GAATCTNGTAATGAATGGTGTGGCTGTTGAGCAAGTTGAGGAGACTAAAC

TGCTTGGCGTNACCCTAGATTGTAAACTGTCATGGTCAAAACATATTGAT

NCAACGGTNGCNAAGATGGGGAGAAGTCTGTCCATAATAAAGCGNTGCTC

TGCTTTCTTGACACCACTNTCAACAAGGCAGGTCCTGCAGGCCCTAGTTT

TGTC

>rnd-3_family-45#Unknown ( Recon Family Size = 49 Final Multiple Alignment Size = 47 )

AATGTGACCGACCGNCTCGATTCGGTCTTATGTAGCAAAATTTGAAATTG

TGTTTTTTACATTGGATAAAAGTAGAGACTCAGAGCTACAAAATGGTATA

TCATACACTGCATTTGAGGAACAATGGGAAAGTAATTCTGCTTTGAAAGT

TGATAAACTTGTAANCTCACTTTTGAGAAAATGGCCTTTGAATGTTTTGG

TACCCTACTGGAGAGCTCTTCTTTGTCTACACCCATTCAGCATCGTTCAC

ACCCTC

>rnd-3_family-70#Unknown ( Recon Family Size = 51 Final Multiple Alignment Size = 45 )

TTTTTTATTTCGATGGCTCACTGTTCGTTGTTGGCATTTCCTGCCTAAGT

TTGCCCACTTTGCCAATGAAATAATCATTAAAATAATTGGCAACATCAAA

TGGTTTTGTGATGAATAAGCCATCTGATTCGATGAANGATGGAGCCGAGT

TTGTCTTTCTGCCCATAATTTCATTTAAGGTGCTCCAAAGTANTTTTTCC

ATCATTCTTTATATCATTGATCTCGGCTTCATAATACAGCCAGTCGGATG

TGCAGCCAGACTTATTTGCCACTCCTTTTCCCCATCCCTCTCAACCATAC

AGTTTTTCAATTCCTCATCAATCCACGGGGATTTAACAGTTTTTACAGTC

ATTTTCTTAACGGGTGCATGCTTATCAGTAACTGGAATAAGCAATTTCAT

AAATGCGTCAAGTGCAGCGTCTGGNTGCTCCTCATTAATCACACCAGACC

AACAAATATTTTTNACATCATCAACATAGGAATCACTGCAAAACNTNTTG

TATGACCTCTTATACACTATNTTAGGCCCAGC

>rnd-3_family-131#Unknown ( Recon Family Size = 55 Final Multiple Alignment Size = 42 )

GGTCGTTCCACCAATTCGGTGCCTTTTGAGNAGTGTAACTTGGTNAAAAA

CACTTTATTTCACCTAATTTTAACATTCTGTCATAAAGAGCACATGTTCA

ACTTCAAAAAAAAACACGTTTTCCCATCTCAAGAGGTTAAATAAAAAAAC

TATAGTAAGTGCCTATTAAGTGCCAAATAAAGTAACAGGGTTGACGATTT

CATCTTAAATCAGCCATAAATCCCCTTGTGACAGGGGGAATGGAAGCTTG

TTGTGTGCAACAGGGAGGGGCAATTGA

>rnd-3_family-210#Unknown ( Recon Family Size = 44 Final Multiple Alignment Size = 41 )

TTTTACTAATTTATTGCCACCGGGGCCCGCCGGTGTAACTGCTAAACTGC

TTACTGACTGTACACTGTAACGTTACTGCATGATTGTAGCGGGTTTACTA

ACGCGTTAGTTCTATTAGCTATGTTGACTATGACGTTACTTTAGCTAATA

TGGTGACAACGATGTAGGCTGTGTGTAGCGGTTATGATATGGTTTGGCTT

GGAAAGNTTTTTTCGCCTGGTCACATACAGCTGATGTGTTGTGCATTGAA

GTCCACAAGCGAAGGGAAAAGGTGAGAGGAGGAGAGCGCGTAGATGCGAG

AAGGAATACAACGTGGCTGCTATGAAAGTGAACTGTGTTTACGCGTGATC

AGGGGTGTATTCATTCCGCCGATTCTGTTGAAAAACGTTTCTTAAACGGA

AGCAAACGGAACGAAACGGGGATAAACATACCTGAATTTGTCCAATAGAA

ACTCTCGTTTGCAACTGTTGGACTAATGATTACACCCTATATCAGCTAGA

TGCAGGAGGCGGTATTGAATGTGTCACTGTCTGTCACCTCGAATTTTCTC

GACCTGTGTGCACCTACGTTGTAAACTTTAAT

>rnd-3_family-280#buffer ( Recon Family Size = 67 Final Multiple Alignment Size = 41 )

ACTAGCAATACAAACCGATTGTCATAGCTAGCTAGCTAACCAACCAGGTT

CAATGTTAGCTAGCTAGCTAGCTAACATTAGGCTATAACTAGCAANGCAA

ATGGCTTCTGAGAAAACATNAATAACGTTACTACACAGATCATACACGTA

ATCGTTAGCTAGCGAGCCAGCCAGCTAACNTCAGCTAAAGTTAGCTAGCT

AACAGCAAGCTTTAACTTGGAGTCTTTTGTTTAGACATGTCACGCTAACG

TTAGCTAGCTAAACAATGAACCATAATCCCAACCCATAGGTAACGTTACT

AGCAATACAAACCGTTTAACCAACCAGGTTCAACGTTAGCTAGCGAGCCA

GCCAGCTAACTTTAGCTAGCTAGCTAACAGTACGCTTTAGCTTGAAATGA

AAACNACTTTCTGACAAAATTAGAAACGTGTAATATCTGAAAATGTAGCT

AGACTATCTTACCCGTATACATGGATGGACGCTTCACGGCGGACTGCAAC

CCCTTTCATAAGANATCCCGTGTTGTTTCCGTTTAGTTTGCACAGCTTGT

TTGGCCCGTTGTGTTCCACTGATTTCAAAACGTGTTATTTTCAGAGTGAG

AGAGNCAGCATTGCGTTGCACGATCGTCGTCCAGAAAGTAGTCCATCGCA

ACTTTTTC

>rnd-3_family-423#SINE ( Recon Family Size = 72 Final Multiple Alignment Size = 39 )

GGCTCAGTTGGTAGAGCATGGCGCTTGCAACGCCAGGGTNGTGGGTTCGA

TTCCCGGGGGGACCCGTACGTAAAATGTATGCACTCACGACTGTAAGTCG

CTCTGGATAAGAGCGTCTGCTAAATGACAAAAAT

>rnd-3_family-613#Unknown ( Recon Family Size = 45 Final Multiple Alignment Size = 38 )

GTGCGTACTGGTGGCGGGGAAGTCAGGCGCAGGAGAGCGAAAANTGAGTT

ACAACGGCGTCATTTAATNATCCGTAAACACCGCAACANAACAATACAAT

AAACGGGTCGAACAAAACCCGGTAAACACCAGCGTACCGCGTATAAGCAC

TACAAAAAACAATCNCGCACAAAGACATGAGGGGGAACAGAGGGTTAAAT

ACACAACATGTAATCGGTGGAATTGAAACCAGGTGTGACGGAAGACAAGA

CAAAACCAACGGAAAATGAAAAATGGATCGGCGGCGGCTAGAAGGCCGGT

GACGTCGACCGCCGAGCGCCGCCCGAACAAGGAGAGGGGCCGACTTCGGC

GGAAGTCGT

>rnd-3_family-98#Unknown ( Recon Family Size = 38 Final Multiple Alignment Size = 37 )

AAAGCTGCAATATGTAACTTTTTGGGCGACCCGACCAAATTCACATAGAA

ATGTGAGTTATAGATCTGTCATTCTCATTGAAAGCAAGTCTAAGAAGCGG

TAGATCTGTTCTATGTGCGCTATTTCTATGCTTCCCGTTCTTAAGTTTCG

TTTTTGCGTCTTTTACTTTCGGTTTTGTACACCAGCTTCAAACAGCTGAA

AATACAATATTTTCGGTTATGGAAAATATATTTCACAGCGGTTTAGATGG

TACAATGATTCTCTACACTATACTTGCTTGTTTTGTCACATAAACTGAAA

TTAGGCGAACTATTAGAATTTTAGCAACCAGGAAATGGCGGAGCGATTTC

TGCATA

>rnd-3_family-237#Unknown ( Recon Family Size = 38 Final Multiple Alignment Size = 37 )

GTAACAGTATAACTTTAGACCGTCCCCTCGCCCATACCCGGGCGCGAACC

AGGGACCCTCTGCACACATCAACAACAGTCACCCACGAAGCATCGTTACC

CATCGCTCCACAAAAGCCGCGGCCCTTGCAGAGCAAGGGGAACNACTACT

TCAAGGTCTCAGAGCGAGTGACGTCACCGATTGAAACGCTATTTAGCGCG

CACCGCTAACTAAGCTAGCCGTTTCACATCCGTT

>rnd-3_family-229#Unknown ( Recon Family Size = 37 Final Multiple Alignment Size = 36 )

ACGGTGCGTGAATGAGGACCCAAAAGCGAATTAACTTAAACAGAGCTTCT

TTAATNACCAAACATAGGTAGGCTCAGATGGACCGGCAGATTCCGACAGG

ACAGGACAAGGTTACAGCAAACATGACGACAGTCTGGTTCAGGCATGAAA

CACAACAAACAAGAATCCGACAAGGACAGGAGCAGAAACAGAGAGAGATA

TAGGGGCCTAATCAGAGGGAAAAAGGGAACAGGTGGGAAAAGGGGTGACG

AGGTAGTTAGGAGGAGACAAGGAACAGCTGGGGGAAAGCGGGGGAGAAAA

GGTAACCTAACAACGACCAGCAGAGGGAGACAGGGTGAAGGGAAAGGACA

GAGACAAGACACAACATGACAGTACATGA

>rnd-3_family-67#Unknown ( Recon Family Size = 36 Final Multiple Alignment Size = 35 )

TACAGATGTAGGATCTTAATTCGATCACCCTTTGTTGCATGAGAACTTTC

CTGCANNGCAGGAAATGCAAAACTGTAGTGTATTCGAGGTTTAAAAAGGC

TTCTGAAGTTTGTAATTTCCACTTCGAAATTTCAGACTTGATTTTCCCTT

ACGAAAAAATGTATCAACCCCTACAAAAATGTCCATTAATTATAATCCAC

ATAATAATTCACATTTCCTGTTGCTGCAGGATTATTTTCCTGCTGTAGCA

AACTGGCTCAAATTAAGATCCTACATCTGT

>rnd-3_family-20#LINE/L2 ( Recon Family Size = 72 Final Multiple Alignment Size = 34 )

TATCGGCCTATATCGAATCTCCCATTCCTCTCAAAAATNTTAGAAAAAGC

TGTTGCGCAGCAACTCACTGCCTTCCTGAAGACAAACAATGTATACGAAA

CGCTTCAGTCTGGTTTTAGACCCCATCATAGCACTGAGACTGCACTTGTG

AAGGTGGTAAATTACCTTTTAATGGCGTCAGACCGAGGCTCTGCATCTGT

CCTCGTGCTCCTAGACCTTAGTGCTGCTTTTGATACCATCGATCACCACA

TTCTTTTGGAGAGATTGGAAACCCAAATTGGTCTACACGGACAAGTTCTG

GCCTGGTTTAGATCTTATCTGTCGGAAAGATATCAGTTTGTCTCTGTGAA

TGGTTTGTCCTCTGACAAATCAACTGTAAATTTCGGTGTTCCTCAAGGTT

CCGTTTTAGGACCACTATTGTTTTCACTATATATTTTACCTCTTGGGGAT

GTCATTCGAAAACATAATGTTAACTTTCACTGCTATGCGGATGACACACA

GCTGTACATTTCGATGAAACATGGTGAAGCCCCAAAATTGCCCTCGCTAG

AAGCCTGTGTTTCAGACATAAGGAAGTGGATGGC

>rnd-3_family-375#DNA/hAT-Charlie ( Recon Family Size = 35 Final Multiple Alignment Size = 33 )

CAAATGTGATATAGCGAAGCACCTGAGTGAGTTGGGTGCGCAATTACGCA

GGTACTTTCCCGAAACGGACGACACAAACAACTGGATTCGTTATCCCTTT

CATGCCCTGCCTCCAGTCCACTTACCGATATCTGAACAAGAGAGCCTCAT

CGAAATTGCAACAAGCGGTTCTGTGAAAATTTAATTTAATCAGAAGCCAC

TGCCAGATTTCTGGATTGGGCTGCGCTCAGAGTATCCTGCCTTGGCAAAT

CGCGCTGTTAAGACACTGATGCCCTTTGCAACCACGTACCTATGTGAGAG

TGGATTCTCGGCCCTCACTAGCATGAAAACTAAATACAGGCACAGACTGT

GTGTGGAAAATGATTTAAGACTGAGACTCTCTCCAATACAACCCAACATT

GCAGAGTTATGTGCATCCTTTCAAGCACACCCTTCTCATTAACCTGTGGT

GAGTTATTCACAATTTTCGATGAACAAATAAGGTTTTATATGTAAGATGG

TTAAATAAAGAGCAAAATTATTGATTATTATNATATTATTATTTGTGCCC

TGGTCCTATAAGAGCTCTTTGTCACTTCCCACGAGCCGGGTCGTGACAAA

AACTCACACTCATTCTTATGTTTAA

>rnd-3_family-76#Unknown ( Recon Family Size = 37 Final Multiple Alignment Size = 33 )

AAGAGACCCTTAAACCCAGACTTGGACCACACACCCACTCCACTGAATAG

CAGGCTAGTGATTGCTTTGCAACGCTTGCAGTTAGCCACTGATTCCTTCC

AAACCACTCATTGTTGAATTTGCGATTTCCAACTTGTTGTGTAATGTTTA

TGTCCAATGGCCGATGAGCACCGATACGTTTTATCTATAATTTCTCTTCA

TNATTTCTCTTCATATGACAAGGATTGAAAAGGATTTGCCAGTAGATTGT

CGACTTGATTCATGATGATGACTGCTAGCTAAGATTTTGAAAGTATGATG

TTGACATGATCAGTCCAATCAAAGCTACTGTAGATATAACGTGATTTGAC

GTCATTTTATCTGTGGCCAATGACCTTGAGCCTTCTTGGATGGGCACTTC

TAATGTAACTCTATGGCAGCACCCAAGGGGCTTGAATTTTCGAGCTCTAC

CCGTAGATTTTGCGGTGACGTAGTGTCCCCATGAGTGACAGAACACTGAG

CCAATCACGGCGCAACTAGAGAACATTACCAACCCCTACGCTCCGTATTT

TCCGCTGGCTGCCCCACCACCACAGAAAGCACTGAGCTAGGCTGAAACAC

CTGCATTTTGGAGCTGCCTTACTCAAGAAAGCAAA

>rnd-3_family-306#DNA/hAT-Charlie ( Recon Family Size = 34 Final Multiple Alignment Size = 33 )

GTTGGTATCTGTACTGATGGCGCAAAAGCCATGACAGGGAGACATAGTGG

AGTGGTAACGCGCGTGCAAGCAGTTGCTCCCGACGCCACTTGGGTACACT

GCAGCATCCACCGAGAGGCTCTTGCTGCCAAGGGAATGCCTGACAGCTTG

AAANACGTTTTGGACACTACAGTGAAAATGGTTAACTTTGTTAAAGCAAG

GCCCCTGAACTCTCGTGTATTTTCTGCACTATGCAATGATATGGGCAGCG

ACCATGTAACGCTTTTACAACATACAGAAGTGCGCTGGTTATCAAGGGGC

AAAGTATTGACACGTTTTTTTAATTGAGAGACGAGCTTAAAGTTTTCTTT

ACTGACCATAATTTTCACTTGTCTGACCGCTTGCATGATGACGAGTTTCT

CACACGACTGGCCTATCTGGGTGATGTTTTTTCTCGCCTGAATGATCTGA

ATCTAGGATTACAGGGACTCTCCGCAACTATATTCAATGTGCGGGACAAA

ATTGAGGCTATGATTAAGAAGTTGGAGCTCTTCTCTGTCTGCATTAACAA

GGACAACACACAGGTCTTTCCATCATTGTATGATTTTTTTGTGTGCAAAT

GAA

>rnd-3_family-312#Unknown ( Recon Family Size = 58 Final Multiple Alignment Size = 32 )

AGCAGTCCCAACACCTTACCACCGCTACACCTGGCTATCAGCGGAGCCTT

GTCTGGCAGCGAAACAGTTCATTCAGCCTCATTTACTGCCTTTTAAAACA

TAGCTGATATGGCTGACTTGCTTAAACAAATGTGGTTTCTACTGACAATT

GAGATGTACAAACTATGGCATAAGGGGACGACGAGCGGATAAGAGGCAAT

CCGTAATTTCGATTAAGACATTAATGAGCGAGCTAGGACGGACGTAGTCA

ATATAACTATTTGTTCAGCACTTTTGAAATGTACAGCGACAGAATTCAGA

ACATGGGCCGTTCTTACAGTATTCTCCCTGTACACCAAGTCAGAACCGTA

GGATAAATAAAGGGGGCATATAAGCAGACAATGAAAGCTCTTACAATATT

CGATGATTACATTTCTCTAAAACAGGCTATAGGCTACATGTGCACCACCA

AGTCAGAACAGTAGGCTAAGTTANGAGGGGGAAANGGACCAAATTATTAG

GGTGAGGCACA

>rnd-3_family-368#DNA/hAT ( Recon Family Size = 39 Final Multiple Alignment Size = 32 )

AATGTACTTTTTACTCCATACATTTTCCCTGACACCCAAAAGTACTCGTT

ACATTTCGAATGCTTAGCAGGACAGGAAAATGGTCCAATTCACGCACTTA

TCAAGAGAACATCCCTGGTCATCCCTACTGCCTCTGATCTGGCGGACTCA

CTAAACACAAATGCTTCGTTTGTAAATTATGTCTGAGTGTTGGAGTGTGC

CCCTGGCTATCCGTAAAAAAAAAACAAGAAAATCGTGCCGTCTGGTTTGC

TTAATATAAGGAATTTGAAATGATTTATACTTTTACTTTTGATACTTAAG

TATATTTTTGAAATTACATTTACTTTTGATACTTAAGTATATTTAAATAG

ACTTTTACTCGAGTAGTATTTTACTGGGTGACTTTCACTTTTACTCGAGT

CATTTTCTATTAAGGTATCTTTACTTTTACTCAAGTATGACAATTGGGTA

CTTTTTCCACCA

>rnd-3_family-1#LTR/Gypsy ( Recon Family Size = 50 Final Multiple Alignment Size = 31 )

GCGCAAGGTCCTCCGGCGCCTGTTGGAGCATGACCTGTNCGTGAAGGCTG

AGAAGTGCGNGTTTTTCCAACGGTCCGTCTCCTTCCTGGGNTATCGCATT

TCCGCNTCAGGGGTGGAGATGGAGAGCGACCGCGTTACGGCCGTGCGTGA

TTGGCCGACTCCAACCACGGTNAAGGAGNTGCAGCGNTTTCTNGGGTTCG

CCAACTNCTACCGGAGNTTCATCCGGGGTTTCGGCCAGGTGGCNGCTCCC

GTTACCTCACTGCTGAAGGGGGGCCCGGTGCGCTTGCAGTGGTCGGCTGA

GGCGGACAGGGCTTTCGGGCACCTGAAGGCTCTGTTCACCNCGGCNCCCG

TNCTCGCCCATCCGGACCCCTCTNCGNCGTTCGTGGTGGAGGTGGACGCG

TCCGANGCTGGGGTAGGGGCCGTCCTNTCCCAGCGCTCGGGTACGCCACC

GAAGCTCCGCCCCTGTGCCTTCTTCTCNAAGAAGCTCAGCCCGGCGGAGC

GAAACTATGATGTGGGGAACCGGGAGCTGTTGGCTGTC

>rnd-3_family-624#Unknown ( Recon Family Size = 56 Final Multiple Alignment Size = 30 )

TGACCAATCAGGACCTGAATATGACTGCACGTCACATAATAATTTAACGC

GTTCATACATTTTTTACGTAGTTATTACACATTGATTACACTATCACTCG

TATTTCATATGTCACAACGATTCGTCGATACGTATGCTATGATGCTGGTA

AAGTTGTCTCGCGCACCTACAGTGCTGGTCATAAAAAAAGCTAGCTAGCT

CATGGATGCAAACAATGTTCTTCCCCAAAAACATAGCAAAACGACAATCT

GTTTCAGTAGCTATAGTTAGCTAGCTAACTATATAGCTAGGTGTCATCAT

CTAAAATAACCCTAATTTATAAGACAGTTCTTATTTGATTAATGGTGGTC

GGACCCATCTATGTGAAGCTAGCCACAATAAGGATTAGCCACAATAGTGG

ACTTTGCGGTTNGCCTTCAAAATAAAAGTACGTCATTGACAGTGATGCAA

ATTAATACAAATAGTGGAATCATGCCATAATTGAATAGATCATGCTAAAC

GAGGTTGGAATGTTATATAAATTCAACAAAAGACAATAATTTGTTAATTT

GACAAAAATCTGTTGAAATCACACTGTGGATGTATTAGACTTTAGAATTG

CATTGGGGGCATACTTATTTCACTGTACAGCCTTACCTATG

>rnd-3_family-1394#Unknown ( Recon Family Size = 32 Final Multiple Alignment Size = 29 )

CTTCTCTGCCCTTAACCTTGTTCTGAACACCTCCAAAACAAAGGTCATGT

GGTTTGGTAAGAAGAATGCCCCTCTCCCCACAGGTGTGATTACTACCTCT

GAGGGTTTAGAGCTTGAGGTAGTCACCTCATACAAGTA

>rnd-3_family-8#Unknown ( Recon Family Size = 30 Final Multiple Alignment Size = 28 )

TACGACGGGTGGGTCTAATCCTGAATGCTGATTGGTTAAAACCGCATTCC

AGCCGGTGTCTATTCCACAAGTTACCACCGGCTAAATCTATGACGTTAAA

ATGCCTATTTACTCTGTTCCATCTGACTGCGCAATCCACTGTCTCATCAG

CCCAGCCAGGCAATTTATAAACTTGATCTCCACTGTAAAAAGCATCTAGA

CATTATCTCACATTTCTTTTAGACTAACATTTCGTTTTCAACAGCGGAGA

TTTGTATAAACCTTGCTGTCTGTCTCTCCGACATTTGCAACATTGTTTCA

ATATTCAAATTCGATCTCCAGCTGTCCCATAGTAATGAACGTGTAGGGGT

CGGGAGTCGGGACGAGACAGGCAGGCAGCTTTTCTCAGCCAGTCGAAATC

GCGAATCAGCTGGCATCATTTGTTATGGATATATACAAANAAATGTCAAT

AGAAAACAGGTCAAACAAACGAAATGCGGCTAGTTTGCAGTCTTTCCGGC

TTCAGTTTGAAGTGATTNTGTTAGCT

>rnd-3_family-184#DNA ( Recon Family Size = 30 Final Multiple Alignment Size = 28 )

AAGCAATAAGGCACGAGGGGGTGTGGTATATGGCCAATATACCACGGCTA

AGGGCTGTTCTTACGCACGACGCAACGCGGAGTGCCTGGATACAGCCCTT

AGCCGTGGTATATTGGCCATATACCACAAACCCCCGAGGTGCCTTATTGC

TATTATAAACTGGTTACCAACGTAATTAGAGCAGTAAATGTTTTGTCATA

CCTCTGATATACCACGGCTNTCAGCCAATCAGCATTCAGGGCTCGAACCA

CCCAGTTTATA

>rnd-3_family-422#Unknown ( Recon Family Size = 34 Final Multiple Alignment Size = 28 )

TGTGTTGTTTATTGTTTTGTTNGNTGGACATTTTTTAATAAAGAGAAAAT

GTACGCTCACCACGCTGCGCCTTGGTCCGCTTCNTTCGACGGCCGTGAC

>rnd-3_family-331#Unknown ( Recon Family Size = 32 Final Multiple Alignment Size = 28 )

ACTCGCGAAAAATTAACACCACCCTACTCCACTACTTAACCCTATCTANT

CCTACCCCAGGCCAACGGCCTGAGAGGACGGGACACCACCACTTAACACA

CCCTGTAACTCTTCTGNNGTCAAATCTCGCACACCCAAGTACCTCTCTGC

AGCTGCCACCACAACCTCTATTTTCTGTGACTTACGTTCCATCCCTGCGG

TACAGTTGATAACCATTGCTATGAACGCTAAAAATCCAACCTTACTGAAA

CATATCACTTGTTGGCCTATCCCTCTACTGGTACAGATCTACTACTCACA

CCACTCCTCTCAGGATCCCTCACCCTTGACCCATCTTCCTCTACTCTCTT

CACTGCCTCAGCATACGACAACTTCTGCACTACTCTGACCCTGGAAACCT

CAACCTGCCTCTCTCGCACCGGACATTTCTGATCCCCAGCCCCATGGGCA

CCCCTACAATTAACACATACCACTACTTTCCCCGATACTACACATTCCTT

TGTCTCATGCCCTCCTGCACACTTCTCACACCTAGGAACCTCCCTCCTAC

ACACTGCTGCCACATGCC

>rnd-3_family-159#Unknown ( Recon Family Size = 32 Final Multiple Alignment Size = 28 )

CAGTCACAGTGGTCAGGTATTCTGCCACTGTGTACTCTCTGTTTAGGGCC

AAATAGCATTCTAGTTTGCTCTGTTTTTTTGTTAATTCTTTCCAATGTGT

CAAGTAATTATCTTTTTGTTTTCTCATGATTTGGTTGGGTCTAATTGTGT

TGCTGTCCTGGGGCTCTGTGGGGCGTGTTTGTGTTTGTGAACAGAGCCCC

AGGACCAGCTTGCTTAGGGGACTCTTCTCCAGGTTCATCTCTCTGTAGGT

GATGGCTTTGTTATGGAAGGTTTGGGAATCGCTTCCTTTTAGGTGGTTGT

AGAATTTAACGGCTCTTTTCTGGATTTTGATAATTAGCGGGTATCGGCCT

AATTCTGCTCTGCATGCATTATTTGGCGTTCTACGTTGTACACGGAGGAT

ATTTTTGCAGAATTCTGCGTGCAGAGTCTCAATTTGGTGTTTGTCCCATT

TTGTGAATTCTTGGTTGGTGAGCGGACCCCAGACCTCACAACCATAAAGG

GCAATGGGTTCTATAACTGATTCAAGTATTTTTAGCCAGATCCTAATTGG

TATGTCGAATTTTATGTTCCTTTTGATGGCGTAGAAGGCCCTTCTTGCCT

TGTCTCTCAGATCGTTCA

>rnd-3_family-356#Unknown ( Recon Family Size = 29 Final Multiple Alignment Size = 27 )

GCAGAGCGGCACCAGAATTAAAAACACGTCTTACCTTTTTGTAGTTAATA

AATCCAACGTGAAACGTGATAACTATAGTATCCTTAACTAGCATTGAAAA

AGTTAATCCATTCTTCTAGCTAATTAAAAATCTCTCCCTATCTTCTGAAT

CACGCTTGTAACGTCAGTAGGCTACAGTAGCCTATGCTTCGGAAGGGGGA

GGGGCAGGTAGCCTACACACGCACAGGCAAAGATTTTCAGCTTGCAGGCA

GACACTGGAATACGTTTCTGAGTGACAGAGTGAGGGCTTTGCATAGGCGC

TTTGTTGCGTTTTTTTGTGGGACTGAAAAAAATGCCCGGAACGTAAAATA

ACGTTATTAACCGGTTCCCATGCTTTTAAAATAACGGTTCTGTTCCGGAA

CAGTATGGATCACTTTCGTTCCNGGTTCCGNTTCTGTTCCTCGAA

>rnd-3_family-104#Unknown ( Recon Family Size = 31 Final Multiple Alignment Size = 27 )

TTTGTTGGTGCGTGCGAGCAGTGTGGGTGCAATAATTGAACAANAAGTAC

GTTGGCATTTTCCCACCCGAGATGTGTTTCCATCAAATTGACTTGTTGCG

GATAAAAGGCTGTGCGTGATGACGTAGTGCACATAAAAATAACTTTTGCG

GTTAAATTCCCATGTACCGAAAAAATACAAGTTAAATGGGTTTCTATCGC

ATTTTCAACTCTACTGATGGTTTTGTCACAAAAAAATGTTGGTNAAATAG

CGAATGTGCCCACTCTGGTCTTGGCACGTGCGCTCTAGCCAACAGCTCGC

AGATACAGTGCGGGTATAGCCTACATGATGAGATTATTATGGACAAAAGT

GCAAGATTATTTTTATTTGTCAAACGGCAGCCAAGCATCGATCATCATGT

CACCAGAATAAGACCCTCGATATTTATTGGAAAGGAGCATCAAGCTCATC

GCCTTGCACTTTCACCACCCTGTGAAGTTCGTCATAACTTATTTCATCTG

TAGCCTAATAAAC

>rnd-3_family-350#Unknown ( Recon Family Size = 32 Final Multiple Alignment Size = 26 )

GCACTCCACGAGGAGNCTGCCTGTTACGCGAATGCAGTAAGCCAAGGTAA

GTTGCTAGCTAGCATTAAACTTATCTTATAAAAAACAATCAATCATAATC

ACTAGTTATAACTACTAATCCAGGTTAGCAGGCAATATTAACCAGGTGAA

ATTGTGTCACTTCTCTTGCGTTCATTGCACGCAGAGTCAGGGTATATGCA

ACAGTTTGGGCCGCCTGGCTCGTTGCGAACTAATTTGCCAGAATTTTACG

TAATTATGACATAACATTGAAGGTTGTGCAATGTAACAGGAATATTTAGA

CTTAGGGATGCCACCCGTTAGATAAAATACGGAACGGTTCCGTATTTCAC

TGAAAGAATAAACGTTTTGTTTTCGAAATGATAGTTTCCGGATTCGACCG

TATTAATGACCAAAGGCTCGTATTTCTGTGTGTTATTATGTTATAATTAA

GTCTGATTTGATAGAGCGGTCTGGCTGGGCAGCAGCAGGCTCGTAAGCAT

TCATTCAAACAGCACTTTCGTGCGTTTTGCCAGCAGCTCTTCGCAAGCAC

NGCGCTGTTTATGACT

>rnd-3_family-51#Unknown ( Recon Family Size = 30 Final Multiple Alignment Size = 26 )

TCCCAAGTGGGTGTGACACCTACTCCCGTTGCCTGCTGCGCTGCTGCTTG

GATTCCGCCCGGCGATCTGTTCACCGTCTGCCCTGGCCCGTCTCCCACGT

CTGGTACAGGTACCCCCACCACACTCCCGAGCCTTCGCTCACCTCCAGCA

CACGCGCACTGTTGGGGCGAGTGACTCTGAACTTACAATGGCCCCCAGGT

GTTATATATNTTTTCTTGTGCATTTTGCTATTTGCCTCATGACTCCTGCG

TGCTTTGTCGACTACGAACTTGCTTGTTTACCCAACCGCGGGACAGACTA

TGTTTGTTCCCACACTCGGGACTCTCTNTTGGCCTCCCATCCTATTCTAA

CCACCTCTCGCCGGCTTTGTATTCATGTTACCTGATGAAATTGCTGTACA

ATATGATCTTGTTGCCATCTGATGCACATTCAAATGTCATAAATCAACAC

TGCAGAGCTCTCCCTGTCCTCTGCCGTGCCCTGATTGTATTACTGCTGAT

GATATCTGGAAATGTGCATGTACACCCTGGCCCATCTACTGTTGCTAGCC

CCAATTCTGACTTGTGCTCTGATA

>rnd-3_family-753#Unknown ( Recon Family Size = 29 Final Multiple Alignment Size = 26 )

CTGAAAAAAGGTGCTATCTAGAACCTAAAAGGGTTCTTCGGCTGTCCCCA

TAGGAGAACCCTTTGAAGAACCCTTTTGGTTCCAGGTAGAACCCTTTTGG

GTTCCACGTAGAACCCTCTNCGGAAAGGGTTCTACCTGGAACCCAAAAGG

GTTCTACCTGGAACCAAAAAGGTCAAAGGGTTCTCCTATGGGGACAGCCG

AAGAACCCTTTTGGAACCCTTTTTTCTATT

>rnd-3_family-206#SINE/Deu ( Recon Family Size = 29 Final Multiple Alignment Size = 26 )

GCTGCTGGAAGTGGTGTTGGAGGGCCAGTAGGAGGCACTCTTTCCTCTGG

TCTAAAAAATATCCCAATGCCCCAGGGCAGTGATTGGGGACACTGCCCTG

TGTAGGGTGCCGTCTTTCGGATGGGACGTTAAACGGGTGTCCTGACTCTC

TGTGGTCACTAAAGATCCCATGGCACTTATCGTAAGAGTAGGGGTGTTAA

CCCCGGTGTCCTGGCTAAATTCCCAATCTGGCCCTCATACCATCATGGCC

ACCTAATCATCCCCAGCTTACAATTGGCTCATTCATCCCCCTCCTCTCCC

CTGTAACTATTCCCCAGGTCGTTGCTGTAAATGAGAATGTGTTCTCAGTC

AACTTACCTGGTAAAATAATGGATAAATA

>rnd-3_family-73#Unknown ( Recon Family Size = 27 Final Multiple Alignment Size = 26 )

CAGAGGTAGACATGCTCATGTTATTTATGTTAGTGCAGGGTGAGCTGCAC

ANAGTGGNCTTCCTACTAGGGCACACCGCCTCAGTGCTAACAGTATAACT

CTGGTTCATAGGCNCATGATTACTGCNTACAATAGCTGTAGGATCAGCAG

AGGCATTCGGGGCAGTTAGAGGGACATAAATTAGGTTACTTACATTGTGT

CTGCCAACGCCCCTGGGATNATGTACATTTGCTGCAGCATTATGACGACT

CAGCGACACAATGGTAGGGATTAACTGAGCTGGGCTTGGGTCATTGATAA

GTCATTGTCTCAACGCAGCCTTGAAATGCGTGGACAGGGTCCAGGAGCCA

AGATGATTTGGATGGACTCCGTCATCCCTGTAGAACANGCTTTGTTTCCA

GAAGGTNTCAAAGTTGTCAATAAACGTGACACCCGCAGAGCTGCAGTAGT

CTCGTAGCCAGTTATGGAGGGCTAGAAGTCTGCTGAATCGTTCAATGCCA

CGATTTAGGNAGGGCAGAGNGNCAGATATTA

>rnd-3_family-211#Unknown ( Recon Family Size = 26 Final Multiple Alignment Size = 25 )

CGAAGACGTGGATGTCGATTAAGGCAGCCCCCCGCACCTCTCTGATTCAG

AGGGGTTGGGTTAAATGCGGAAGACACATTTCAGTTGAATGCATTCAGTT

GTACAACTGACTAGGTATCCCCCTTTCC

>rnd-3_family-466#DNA/TcMar-Tc1 ( Recon Family Size = 26 Final Multiple Alignment Size = 25 )

AGAGGAAGGCATTGATGCTATGGACTGGCCCGCCCGTTCCCCAGACCTGA

ATCCAATTGAGCACATCTGGGACATCATGTCTCGCTCCATCCACCAACGC

CACGTTGCACCACAGACTGTCCAGGAGTTGGCGGATGCTTTAGTCCAGGT

CTGGGAGGAGATCCCTCAGGAGACCATCCGCCACCTCATCAGGAGCATGC

CCAGGCGTTGTAGGGAGGTCATACAGGCACGTGGAGGCCACACACACTAC

TGAGCCTCATTTTGACTTGTTTTAAGGACATTACATCAAAGTTGGATCAG

CCTGTAGTGTGGTTTTCCACTTTAATTTTGAGTGTGACTCCAAATCCAGA

CCTCCATGGGTTGATAAATTTGATTTCCATTGATAATTTTTGTGTGATTT

TGTTGTCAGCACATTCAACTATGTAAAGAAAAAAGTATTTAATAAGAATA

TTTCATTCATTCAGATCTAGGATGTGTTATTTTAGTGTTCCCTTTATTTT

TTTGAGCAGTG

>rnd-3_family-22#Unknown ( Recon Family Size = 27 Final Multiple Alignment Size = 24 )

GTACAACATTAAAACCTATTCAGTCTGTCTACTTACAGGCTCTCAAAGTG

CTTGATAGGAAGCCCAATAGCCATCATCATTGTCACATCCTTAGAAAGCA

TGAGCTCTTGAGTTGGGAAAATCTTGTGCAATACACCGACGCATGTCTTG

TATTCAAGATCCTTAATGGCCTGGCTCCCCCTCCACTCAGTATTTTTGTT

AAACAGAAAACCCAGACATATGGCAGCAGATCCACAAGGTCTGCCATGAG

AGGTGACTGTATAGTTCCCCTAAGGAAAAGCACCTTTAGTAAATCCGCAT

TCTCTGTGAGAGCTTCCCATGTCTGGAATACACTGCCATCAGACACACAT

AACTGCACCACATATCACACTTTCACAAAATGCTTGAAGACATGGCTAAA

GGTCAATCAGATTTGTGAACATGGTCCCTAGCTGTGTGTTGCCGCTTTCC

ATGTTGTCTGTTGTCTGTAGCTTGTGAGGTGTGGAAACACTTTGTTGCTT

TTATGAATTTTGTCTTGCTGCTTTTTGTTTTATGTTGCTCTGTCTGTATG

CTACGTCTTGCTTGTCCTATGTTGCTCTGTCTGTATGCTACGTCTTGCTT

GTCCTATGTTGCTATGTCTTGCTTGTTCTATGCTGCTATTGTCTATATTG

TAATTGTTTT

>rnd-3_family-162#Unknown ( Recon Family Size = 28 Final Multiple Alignment Size = 24 )

GACTATCATTAAATGTGATGACTGTTATTTTATCGAATCAATTAACTACG

TAATTAATTATTACCCGATTAAACTAATCATGTAACAATTAACTCATTAG

GAATCGGGGCACCACGGAAAAAGTTCGTTTAACGAGTTACCGTCTCCCGA

ATTAACTCTTAAAGAATATATCGNTNTNATATCAGTCATCCATTAATCGT

TATTACCTCACGTCAGTCTCATTCCGAACGTCGTAAATTCTTGGTTATCT

GCACGAACCCTGGCCTANACGATGAATCAGCGATACACAAATTGGCTTAA

TTATTTATTTACTAACTAACTAAATAATCACACAGAAATACATAAACACA

CAGNATAGGTTGAATTGATTACTAACATAATGCAAGGAAAAGTCCCTAGC

GGACTAANCCGATATGACGGCTTGTTACACAANGAAAGGGGTGGGGAGAG

AAAGAGCGGGAGAGACAAAGA

>rnd-3_family-29#Unknown ( Recon Family Size = 30 Final Multiple Alignment Size = 23 )

ACAGCGCTGTATGGGTTTCGACTGACAGCCGTTCTGAACTTGAGCACCGC

GCGCGACAAGAAGTTGCCCCCATCCCTCCCGCTAATTGGGCAACTTTNGC

ANATTTTTCGTTGTCTGAGTGAGGGAAATGCTGTAAATCAAGAGGACTCG

ATGTATTCTGAAAGATGAGACGTTGAGGATTATAATAAATACCGCTTTGA

TGTCATACAAGCAAGCCAAACACCCGTGAGTCCAAATGTGCACTTCACAT

TTCCATATCATAAAACTCATGTCATATACAGTGTCAACGTTTATGATCGC

TATGTTTGATGTAGTTACAAGATGACATGGCGTCATACCCTGGGTTGTCC

TGAACAGAATGAGCCTATGTTTGTTTATTGTGAAGAAAATTTGCCGCGGA

ACACGCACCTGAATGCACTCATGACTCCATTCTATGCGCACCAAAATTAC

GATCTGTTTCTCTGAATTGCCTTGTGAAAGCCTAACACTGTAAGATCGTA

TTTTATAACTTAGCTAGTCATGTTGGCAATAGAACGANCTTTCAAATNAT

GCCCACCTGACCCATGGACCGTTTTTTTTTGGATTGCGTAAACAACAACA

GTAATTGTGTGATGGCGGGGAT

>rnd-3_family-390#Unknown ( Recon Family Size = 26 Final Multiple Alignment Size = 23 )

GGCCCGTATTCATAAAGCGTCTCAGAGTAGGAGTGCTGATCTAGGATCAG

GTCCCCCCTGTCCATATAATCTTATTCATTATGATCTAAAAGGCAAAACT

GATCCTAGATCAGCACTCCTACTCTGAGACTTTGTGAA

>rnd-3_family-23#Simple_repeat ( Recon Family Size = 35 Final Multiple Alignment Size = 23 )

TTACATTAATTTGGGGACAGGTCGAAAAGCATTAAACATTTATGGCAATT

TAGCTAGCTAGCTTGCACTTGCTAGCTAATTTGTCCTATTTAGCTAGCTT

GCTGTTGCTAGCTAATTTGTCCTGGGATATAAACATTGAGTTGTTATTTT

ACCTGAAATGCACAAGGTCCTCTACTCCGACAATTAATCCACACATAAAA

CGGTCAACCGAATCGTTTCTAGTCATCTCTCCTCCTTCCAGGCTTTTTCT

TCTCTTGACTTTATATGGCGATTGGCATCTAACTTTCATAAATAGGTGCA

TTACCGCAACCGACCTCAGTTCGTCTTTCAATCACCCACGTGGGTATAAC

CAATGAGGAGATGGCACGTGGGTACCTGCTTCTATAAACCAATGAGGAGA

TGGGAGAGGCAGGACTTGCAGCGCGATCNGCGTCAGAAATAGAACCGAGT

TCTATTTTAGCGCCTGGCNACGCAGACGCTCGTTGGCG

>rnd-3_family-231#Unknown ( Recon Family Size = 53 Final Multiple Alignment Size = 23 )

AGAGAGAGGTTTAGTGCTTTNATATTTAATAATCTCNGCCCNCCCAATTC

ATATTCATTATATAAATAGGCCCGTTTNATTTTGTCTGGTTTAGCGTCCC

AGATAAAGCGAAATATTTTTTGCTCATATGATTTGAAAAACGAATCGTCA

GGAGTAGGCAGCGCCATAAGTAAGTGAGTAAACTGAGATATGACTAAGGA

GTTAATCAGGGTAATTTTTCCATAAATAGACAGGTATTTACCTCTCCGTG

GTTGCAGGATCTTGTCTATTTTTGCNAGTTTTCTATTGAAATTCATTGTG

GTGAGCTTATTTATATATTTTGNGATATGAATACCGAGTATGTCTACTTC

ACCGTCAGNCCATTTTATNGGTAAACTACAGGGTAATGTAAAAGTTGTAT

TTTTTAANGATCCAATACGTAATATNGTACACTTATCATAATTAGGTTTT

AGTCCAGAGAGTNCAGAAAAGTNATCTAGATCCTCAATGAGACNNTGCAG

GGATCCAGATTGCGGACTTAANAGAAAACTTGAGTCATCGGCGTACANTG

ACACCTTTGTTTTTAAGCCCTGGATTTCTAATCCTCTG

>rnd-3_family-395#Unknown ( Recon Family Size = 25 Final Multiple Alignment Size = 22 )

AGAGGTCGACCGATTAATCGGAATGGCCGATTTAATTAGGGCCGATTTCA

AGTTTTCATAACAATCGGTAATCTGCATTTTTGGACGCCGATTATGGCCG

ATTACGTTGCACTCCACGAGGAGACTGCGTGGCAGGCTGACCGCCTGTTA

CGCGAGTGCAGCAAGGAGCCAAGGTAAGTTGCTAGCTAGCATTAAACTTA

TCTTATAAAAAACAATCAATCTACATAATCACTAGTTAACTACACATGGT

TGATGATAT

>rnd-3_family-271#Unknown ( Recon Family Size = 24 Final Multiple Alignment Size = 22 )

CCTGTAACANCGTAATGTATTCGGCACAAAAGCTCGTACGGGATAACTGA

CATATCCTAACATCACTTTGTCGGGCAAAGACTCAACATCAAAACTCAAA

AGAACAGACAATGACTCTTCTGTTTCACCACTCTCGCCACCCTGTCTGCG

TCGCACCAAACGACGAGCGTCACAAACACCGGGAATCTTCCCCTTCAGTT

GGTCAACTTTCACATTTACCGCTACCCCAGNAATCACTCCTTTCAATGGC

GCCCTTTTCTTGAGAGCAAAACAAGTCACAGCTCTTGCCCCCATTCGTTT

AACGCGGAGCGCCTGCTCCCTCTGACCGGCAGAAACACAAACAATTATCA

CAAGACCACTTCCGGTTACCCTCACCGATTCCACAGCACCCAACTCCGTT

TTCACCCACCCTGAAACCGCANATGGATCAGCCAAAAGGCGAGGGTCCAC

TTTTTCCAAAAACTTCACTCCTACTGTCACAGACTCATCTTTATCCTNAC

CCTCGGTGCAAGCCTCGGGCTCCGAGAACTTCACCACACCTACCGCCTCC

GATACTTCGCCCT

>rnd-3_family-43#DNA/TcMar-Tc1 ( Recon Family Size = 25 Final Multiple Alignment Size = 22 )

AAATGATCACGAGAACGGTGAGCGAAAATCCCAGAACCACACGGGGGGAC

CTAGTGAATGACCTGCAGAGAGCTGGGACCAAAGTAACAAAGCCTACCAT

CAGTAACACACTACGCCGCCAGGGACTCAAATCCTGCAGCGCCAGACGTG

TCCCCCTGCTTAAGCCAGTACATGTCCAGGCCCGTCTGAAGTTTGCTAGA

GAGCATTTGGATGATCCAGAAGAAGATTGGGAGAATGTCATATGGTCAGA

TGAAACCAAAATATAACTTTTTGGTAAAAACTCAACTCGTCGTGTTTGGA

GGACAAAGAATGCTGAGTTGCATCCAAAGAACACCATACCTACTGT

>rnd-3_family-847#Unknown ( Recon Family Size = 41 Final Multiple Alignment Size = 22 )

ACTACAAGACCATGGTGCTTGCCTACGGAGCAGCAAGGGGAACTGCCCCT

CCCTACCTTCAGGCTATGCTCAAACCCTACACCCCAACCCGAGCACTCCG

TTCCGCCACCTCTGGTCTCTTGGCCCTCCCACCCCTACGGGAGGGCAGCT

CCCGCTCAGCCCAGTCCAAGCTCTTCTCTGTCCTGGCACCCCAATGGTGG

AACCAGCTTCCCCCTGAAGCTAGGACAGCGGAGTCCCTGCCCATCTTCCG

AAAGCATCTGAAACCCTACCTCTTCGAAGAGTATCTTAAATAATCACCGC

GCCCT

>rnd-3_family-567#Unknown ( Recon Family Size = 26 Final Multiple Alignment Size = 22 )

GTCACGCCCTGACCTTAGAGAGCCGTTTTNTTTCTCTATTTTGGTTAGGT

CAGGGTGTGACGTGGGGTGGGCATTCTATGTTTTGT

>rnd-3_family-207#Unknown ( Recon Family Size = 24 Final Multiple Alignment Size = 21 )

AAAAACGAACATAGACTGCCCACCCAACTCACGCCCTGACCATACTAAAT

AAAGACAAAACAACGGAAATAAAGGTCAGAACGTGACA

>rnd-3_family-15#Unknown ( Recon Family Size = 25 Final Multiple Alignment Size = 21 )

TATTATATATTTTCCTTTATTATTTTCCCCTAACCCTACCACCCCTCCCC

TAATTGGAGTAAACTAATGGACAACAATACTTAGGCTTCTACTTCCAGCT

TATACATACTATATACATTTTACGGACACAGTATATTTTACANTAGTTAT

CTTTTTTTTTTGTTTTTAGTCCCACCCTTCAGCTCCCCTCAACCCCTCCG

AACACCATCCAGTTTTGATTTCTATTTGCCATATACTTTCAACTGTGCTG

TGATGTTTNACA

>rnd-3_family-527#Unknown ( Recon Family Size = 22 Final Multiple Alignment Size = 21 )

CCTTGACTTGGAAGAGTTCCGGTGTTGGATAGCCATAGCCAGCTAGCTAA

CATAGCATCCCTCTCTGTTTGAGCCGGGTGTTTGAGTAGGCTAAACTAGC

TAGCTGCATTCGCTAGCTAAGTGAAAGTGAAAAAAAATACAACGAAATAT

AGCTATCTCTCTCTCTCTCTCTCTCTCTCTCCTTCATTTTNGAAGAAATN

AATTTGTTCAAAACTGTTCAACTATTGTCTTTCTCTCTCTTTGAGTCAAC

TACTCACCACATTTTATGC

>rnd-3_family-340#Unknown ( Recon Family Size = 22 Final Multiple Alignment Size = 20 )

CGTGGGTTCAAATAGTATTCGAAATCTTTCAAATACTTTGAGCGTTTGCT

CGAGCCTGCCCGGAGTGCCAGATGGGCGGGGTTTGCACTTTTGGGACTAT

TCCATTGGTTCATTGCGCCAGGCAAGCTCAATCAAGCGCAGNTAAAGTAT

TTAAATGATTTCAAATAGTATTTGAACCCAGGT

>rnd-3_family-699#Unknown ( Recon Family Size = 22 Final Multiple Alignment Size = 20 )

AACCCACTGGGCACAGACGTCAATCCAACGTCTTTTCCACGTTGATTCAA

CGTCATTNCGTTGAAATGACGTGGAAACAACGTTGATTCAACCAGTGTGT

GCCCAGTGGAAT

>rnd-3_family-86#Unknown ( Recon Family Size = 29 Final Multiple Alignment Size = 20 )

AACCCGGACGACGTTGTGCGCCGCCCTATGGGACTCCCGATCACGGCCGG

NTGTGATGCAGCCTGGATTCGAACCAGGGACTGTAGTGACGAC

>rnd-3_family-318#Unknown ( Recon Family Size = 24 Final Multiple Alignment Size = 20 )

AGTGGCTGCAATAGTTAGCTTTGTTCGAGCTAAAAAAGAACCGTCGGACC

TAGAGTTCCGAAACTTTAGAAACCTGTTCTAGACCTCCGGTCGATGGTGC

GTGGCGAGTTACGTGGCTCTAGAAGGTTCTCGGACCGAGAAACAGCCTCG

TACATTTGCAATAACTTCAATTCATTTTTGCATCACG

>rnd-3_family-954#DNA/Tc1 ( Recon Family Size = 26 Final Multiple Alignment Size = 20 )

GCCTTGCAAAAGTATTCATCCCCCTTGGCGTTTTTCCTATTTTGTTGCAT

TACAACCTGTAATTTAAATNGATTTTTATTTGGATTTCATGTAATGGACA

TACACAAAATAGTCCAAATTGGTGAAGTGAAATGAAAAAAATAACAAAAA

ANTAAAAAAACGGAAAAGTGGTGCGTGCATATGTATTCACCCCCTTTGCT

ATGAAGCCCCTAAATAAGATCTGGTGCAACCAATTACCTTCAGAAGTCAC

ATAATTAGTTAAATAAAGTCCACCTGTGTGCAATCTAAGTGTCACATGAT

CTGTCACATGATCTCAGTATA

>rnd-3_family-364#Unknown ( Recon Family Size = 23 Final Multiple Alignment Size = 20 )

TAGTCATTGCGTGCTAGGAATATGGGACCAAATACTAAACTATGACTATT

TTATTTTATAAGAATCTTTAGGGGCGTCAGTCATTTTGACCCCTACCTTT

TTGAGCGAAAAAAAAACTTTGTTATTAAACGAAATCTCTTTCTCTGAGCA

ATTGTATTAGTATAAAATAATATAATTTCCCTTTTTTTGCATACAATATA

GCTCAGTATTTNTATTATTTATTTTATACAGTCATTTTTGCTCATCTTTA

TCAAGGGTGTCAATCATTTCGGATATTGAGCACCTAANATCAGGTCATAN

CATGTCT

>rnd-3_family-800#Unknown ( Recon Family Size = 22 Final Multiple Alignment Size = 20 )

GATTGAGGGGGAACCAATTATCTCTTGGCTCCACAATGTCTGTGCGCCAG

TCACTCCGTCTCNGTGAGCTTGTCCAGGAGTGGGGNGAAGAGGGGGTTTA

CTTGAGATGGGAGTATCTAGAGTTGACAATTGATATATGCCATTGGATGA

GGTAATGTTTTGGTACTATGTGGTACCAAGAACGAGATTAGAACCTCGTT

TTAGGAGACCAAACTGAACGATAATTTATAGCNAATGCTGTCTGGCTACG

GGATACTCCTTTCTCAAGTAAAAGNCTCCTTTGTAGAGTTCCTAAGATCT

GTGGTTCGTCATGTGAGTTGAGAGGGGTGTATCTTTGCTATAAAAGATCT

CAGTTGCCATTGTGTCGGNACTCTCAGANAATTCATTTATAGACACTGAA

TTGATCTGAGAGTCACAGGGCTGTGGCGGAGCTCATATTATTAAAGATGG

ACTTTANGTATAACTCTGACTTGTGTGTGGTTTGCNCTCTCATGATTTGG

TAATACAGGAAATTNCCACNACA

>rnd-3_family-266#DNA/TcMar-Tc1 ( Recon Family Size = 23 Final Multiple Alignment Size = 20 )

GTTGCCCAGCAACAGCCCCGAAACATCACTGCTCTAGAGGAGATCTGCAT

GGAGGAATGGGCCAAAATACCAGCAACAGTGTGTGAAAACCTTGTGAAGA

CTTACAGAAAACGTTTGACCTCTGTCATTGCCAACAAAGGGTATATAACA

AAGTATTGAGATAAA

>rnd-3_family-97#Unknown ( Recon Family Size = 23 Final Multiple Alignment Size = 20 )

TAAAGGCCCAGTGCAGTCAAAAACGTGATTTTCCTGTGTTTTATATATAT

TTCCACACTATGAGGTTGGAATAATACTGTGAAATTGTGAAAATTATGAT

AATGCCCTTTTAGTGTAAGAGCTGTTTGAAAAGACCGCCTGAAATTTCGG

CCTGTTTTGGTGGGATCGANCTTGGCCGGCTAGATTAGTTAATAGACCAA

TAAGAAAGAGAGTTCCAAACCTCTCTGCCAATAACAGCTAGTTTTCCGTT

TTCCCCTCCCCACTCAGACCACTCCCAGACAGTCCTAGNCAAATTCTTGC

TTGAGAAATTGCATCTTGCTAAGAAGCTATTTAAAAAAAAAAACATTTGA

CCATTTTGATTGAAAACAATCACAGTAAGGTACTTAATTGTTACCCAGAA

ATGATTTGATATTGAGATAAAAACGGCTGCATTGGGCAGTCTAAGCA

>rnd-3_family-112#DNA/TcMar-Tc1 ( Recon Family Size = 21 Final Multiple Alignment Size = 19 )

NNNNNNNNNNNNNNNNNNNNNNNNNNNNNNNNNNNNNNNNNNNNNNNNNN

NNNNNNNNNNNNNNNNNNNNNNNNNNNNNNNNNNNNNNNNNNNNNNNNNN

NNNNNNNNNNNNNNNNNNNNNNNNNNNNNNNNNNNNNNNNNNNNNNNNNN

NNNNNNNNNNNNNNNNNNNNNNNNNNNNNNNNNNNNNNNNNNNNNNNNNN

NNNNNNNNNNNNNNNNNNNNNNNNNNNNNNNNNNNNNNNNNNNNNNNNNN

NNNNNNNNNNNNNNNNNNNNNNNNNNNNNNNNNNNNNNNNNNNNNNNNNN

NNNNNTGTTCACAAATGCTCTCCATCCAACCTCACTGAGCTCGAGCTGTT

TTGCAAGGAGGAATGGGAAAAAATGTCAGTCTCTCGATGTGCAAAACTGA

T

>rnd-3_family-853#Unknown ( Recon Family Size = 22 Final Multiple Alignment Size = 19 )

GCATTTGTAGTTCTGCATGATAGCCACATTAGCCGCTAATTAGCATTTCA

TTTTGGGGGGTAAATACAGGCGAATATATTGATAAAAGTCACCTTGTCCG

AGAGAGATTTACACGGTTATCAAAACGTCACGCCAGGGTAAGCCTACACG

AAACACAGCCCTTATTTTAAGTTTCTAAAATCCCCTATGGGAAAAACGAT

TGGAACCATTTCCCTGTTTGACCGCTAGGTTTTATGGGTATTATGACTCG

TACTGTGGTAC

>rnd-3_family-169#DNA/TcMar-Tc1 ( Recon Family Size = 21 Final Multiple Alignment Size = 19 )

ATACACTGCTCAAAAAAATAAAGGGAACACTTAAACAACACAATGTAACT

CCAAGTCAATCACACTTCTGTGAAATCAAACTGTCCACTTAGGAAGCAAC

ACTGATTGACAATAAATTTCACATGCTGTTGTGCAAATGGAATAGACAAC

AGGTGGAAATTATAGGCAATTAGCAAGACACCCCCAATAAAGGAGTGGTT

CTGCAGGTGGNGACCACAGACCACTTCTCAGTTCCTATGCTTCCTGGCTG

ATGTTTTGGTCACTTTTGAATGCTGGCGGTGCTTTCACTCTAGTGGTAGC

ATGAGACGGAGTCTACAACCCACACAAGTGGCTCAGGTAGTGCAGCTCAT

CCAGGATGGCACATCAATGCGAGCTGTGGCAAGAAGGTTTGCTGTGTCTG

TCAGCGTAGTGTCCAGAGCATGGAGGCGCTACCAGGAGACAGGCCAGTAC

ATCAGGAGA

>rnd-3_family-908#Unknown ( Recon Family Size = 18 Final Multiple Alignment Size = 18 )

TTATCATATTTCTTTTGGTTTTACTTGTGCATAAGGCATATGTTTTCTCC

ATTTGCAATATGATGTCATAGGAAGTCAAAAGTCAAAGTCAAAAGTAAAT

ATTTTCCTCCGTGCACCTGGTGATCTGAAATGTGCACCTGGTGACCTGAA

ATGTGCACCTCGTGATCTAAAATATGCAGCTGGTTACCCAACATTAAAAT

TTTGGGATGTTTTTTTGTTTATGTTTCCTTACTTTTTCAAAGTCACTGTG

CATTTGCAATATGTTTCAATGGGCAGGTACCATCACGAATTTGTCATGCT

CAAAATTCAAACTTTACTTTGCTCAAACTATACCTTNGTCAACTTGTATT

ATCATATTTCTTTTTGTTTTACTTGTGCATAAGGCATATGTTTTCTCCAT

TTGCAATATGATGTCATAGGAAGTCAAAAGTCAAAGTCAAAAGTAAATAT

TTTCCTCCGTGCACCTGGTGATCTGAAATGTGCACCTGGTGACCTGAAAT

GTGCACCTGGTGATCTAAAATATGCAACTGGTAACCCAAAAAAAATTTTG

GGATGTNTTTTTGTTTATGTTTCCTTACTTTTTCAAAGTCACTGTGCATT

TGCAATATGTTTTAATGGGCAGGTACCATCACGAATTTGTCATGCTCA

>rnd-3_family-63#Unknown ( Recon Family Size = 26 Final Multiple Alignment Size = 18 )

GTCACGTGTGCTCCCTCTCTGGCCTCTAGGTCACCAGGCTGCTCGTTATT

GCGCGCACCTGNCACCATCGTTACGCGCACCTGCGCCTCATGACGCTCAC

CTGGACTCCATCACCTCCCTGATTATCATTGCTTCCGTTTCAGTCTCCCG

TCNGTGCGTTGTTCGTGTTTCTTGTTTTGTATTATGTTTCGTTTATTTAT

TAAAACACTCACTCCCTGNACTTGCTTCCCGACTCCCAGCGCACATG

>rnd-3_family-410#Unknown ( Recon Family Size = 24 Final Multiple Alignment Size = 18 )

TTTCTCCCGTTGTGCTATTTACAAACACGTGACTGGCTCAACTGTTCTGG

GGAACTACGGTAAGCTTCATAATGNAAAATAATGTGACAGGTGAAATGAA

GAACGCGATCTGCTTTATCTCCTAACGTATTGCACAAGTTGACTGCAGGT

ATTTANCTACAAATATAAANATGTATTGAAAAACTTAAAATAAATNGTTT

CGACGGTATTGAAAAACCATCCCGTGGCTATTTCCAAATACCCCGGTATA

CGG

>rnd-3_family-295#Unknown ( Recon Family Size = 30 Final Multiple Alignment Size = 18 )

AGGGCCTAACTGTACGCGCGCCAAATAGCCTACACGCCAATCGCCAAATG

CTTTTGGGAACGGGCAGAAAAAGTTTCGATCCACGGAGGCAAAAAGGACA

ACGTCGGAGTTTAATTCAATAAGAGAAAAGCCGCGAAATGGAGAGTTGAA

AATAAATAGAAGGGAGGGCCAGAAAAGTAATGTTTGGGAAAGATTCGGTG

AAGTGGTAAAAGAGGATGATAGCAGTTGTTGTGTGACGATTGTGAGGCGC

TATACAAATTCGACAGTCACAAGACGGGGACTTCAAATAGGCCTATGGCA

CGTCAAGGGAACTGTAGCCTACTGTTCAGATGGGTTAAATGGAAACTGAA

ATCTGGACACTGACTGTAGGTCTATAACCTCTCACATAGCCTTAATATTA

ACTCCTGCAGAATTAAGCATTTCTTGCAGTAAAATGATACACCAAATGTA

GGCTACATTTTGACTCTGGAACAGGAGTGGAGAAATATATGNATTTATTT

CAGCATCTTGAGAGAATGCGAATGCGCAGTTAGATACCGTCTGTAGAGGC

GCTATCTTTATCAGCATCATAAAAGCACAAAATATGCATCCAAGCCGAAN

TGAAATCTTATCAGAAACATGTTGGGTCGTTTTCACAGCTTTCTATTTCC

TTTCAA

>rnd-3_family-126#Unknown ( Recon Family Size = 26 Final Multiple Alignment Size = 18 )

TAAATATGTATAAAAAAANCAAATATATTTACTNAGCNTTCTTATATCTC

TTAGATATAGGACAGACACTTCAAAACCTTGTTCCTTATGATTTATTTTT

GACTGTCTGTTTTGCCATTTATGAATGTGTTATTCAATGCGTTTCTATGG

GCTATAGTAGTAAAGGGCAAAATTCCAAATCAAATAGCTAAATGATCCNT

GGTATGACCNTCTTAAAACAATTCCATATGTTAGCTTAGTAACCCCCCCC

CCAACGGCTTAACTTTGGAGAANTAA

>rnd-3_family-1085#Unknown ( Recon Family Size = 22 Final Multiple Alignment Size = 18 )

GTTAACTGCCAAAATAAAGGAAACACTTGAGTAAATGAGGGATACAAAGT

ATATTGAAAGCAGGTGCTTCCACACAGGTGTGGTTCCTGAGTTAATTAAG

CAATTAACATCCCATCATGCTTAGGGTCATGTATAAAAATGCCCAGTTGC

CCATTATTTTGGCTACCATGGCTAGAAGAAGAGATCTCAGTGACTTTGAA

AGAGGGGTCTCAAAGGAGCATAGGGGGTTTAAAGAGTGTGTGTGT

>rnd-3_family-525#DNA/TcMar-Tc1 ( Recon Family Size = 18 Final Multiple Alignment Size = 17 )

TAACCATTCCTTTACCTGCAGTGCATTCAAAAGTATTTAGACCCCTTCAT

CCTGTTTCCATTGACATAATTTGGAAAGGCACACACATGTCTATATAAAG

GTCCCACAACTGACAGTGCATTTCAGAGCAAAAAAAGATGAACGGAGCAA

AGTACAGAGAGATCCTTGATGAAAACCTGCTCCAGAGCGCTCAGGACCTC

AGACTGGGGACAAGGACCTTAGCACACAGCCAAGACGACGCAGGAGTGGC

TTCGGGNCAAGTCTCTGAATGTCCNTGAGTGGCCCAGCCAGAGCCCGGAC

TTNAACCCGATCGAACATCTCTGGAGAGACCTGGAAATAGCTGTGCAGCG

ACGCTCCCCATCCAACCTGACAGAGCTTGAGAGGATCTGCGGAGAAGAAT

GGGAGAAACTCCCCAAATACAGGTGT

>rnd-3_family-260#Unknown ( Recon Family Size = 18 Final Multiple Alignment Size = 17 )

TTCCCGTGGTTCATTTTCATGCCAGCCAGGTAGGCTATACTCCTGTTGTA

AAGATAAACAATGTGCTTAATATTAGGAAAGTTGAGAAATAAATATAGTA

GGCCTAGCCTATAGAAAGCTGATGGGATCCTCCTCTTTTTAGTAGAGGCC

ATCACTCTGTTTTCTCGCGCAATTGCATAGCCTATAGAAATGTTGCGCAA

CATGAGCTCATGGGCTCTCATGAAGTGTTTGATTAGATTTTCGATTACAT

TTGCATTGATGTCAGAGTGATTAGAGGGACAATAGAGTGCTGAGTACCAG

GCAGTTAGCAAGTTTGGTAGGCTACTAATGACCATCAGCAGCATCAGAGC

TTGGAGAAGCCTAATTACCGTGACTAAACGGTCACGTGGAATTTGACTGC

CTTCATGACTCGTGACCGCCGGTGTGGCGGTAATACGGTCACCGCAACAG

C

>rnd-3_family-358#Unknown ( Recon Family Size = 19 Final Multiple Alignment Size = 17 )

TTGCCCCTAGACGCTGATCTNGGGTCAGTTTTGCGTTTCCCCCACTAATG

GTTAAGGTTAGGATTGGGGGAGGGGAAGCTGATCCTAGATCTGTACCTAG

GGGAAA

>rnd-3_family-220#Unknown ( Recon Family Size = 20 Final Multiple Alignment Size = 17 )

GGTCGTGTTCATTAGGGCACGCAACGGAAAACGTTTCAAAACGTTTTGCA

ACGGAAAACGAAAATGAGTGTTTCTTATTGGACAAGTCCAGGTAGTCCCT

CCCTGTTTCAGTCCGTTTTCTTCCGTTTGGTGCCTAATGAACACGACCT

>rnd-3_family-290#Unknown ( Recon Family Size = 17 Final Multiple Alignment Size = 16 )

TGCGTTGTTTGCTCTATAACCTGTTAGTTCATATGCCTTGCGACCGTGAT

ATATAGGCCTAAAGGCCGAGACAATAAGAAGACACAGTGGCAGAATAAAT

TCAACCACACCTTTGTTTCATCACAAAACCGGAGAGCAACATCTGTCCGG

TGAAGTCCACAAAGCATATTGCATGTAACAAACAGTTACATGACCTACAG

CATGGTCAAGCAAGTTAATGTTTCCGACATTTTCGGACCACTAAACAACT

ATTGATTTAGAACCACGGAGAGTTACGCAAGTCGCAAAGAAAACAGGAGC

TGCCTCCACTATTCCAGCACCATTTCAACATCATCAAATCACCTATGCTT

AGTCTAATACAGTGACAACTAAAAGATACCAAAAACNATTTAGTCCAATC

AACGTAAGCTAAATAATGTGGCTGTCCATGGTNCTGATTTAT

>rnd-3_family-351#Unknown ( Recon Family Size = 17 Final Multiple Alignment Size = 16 )

ATTTGTGGTCCTGGCAACTGGACCTTTTTTGGAACACCATTATTTTAGTC

TTACAGATCTGACAGAATCTGTGCAGAAGATCTAGGTGCCGCTGTAGGCC

CTCCTTGGTTGGGGACAGAAGCACCAGATCATCAGCAAACAGTAGACATT

TGACTTCAGATTCTAGTAGGGTGAGGCCGGGTGCTGCAGACTGTTCTAGT

GCCCTCGCCAATTCGTTGATATATATGTTGAAGAGGGTGGGGCTTAAGCT

GCATCCCTGTCTCACCCCACGGCCCTGTGGGAAGAAATGTGTGTTTTTTG

CCNATTTTAACCGCACACTTGTTGTTTGTGTACATGGATTTTAAATGTCG

TATGTTTTTCCCCCAAACACCACTTTCCGTCAATTTGTATAGCAGACCCT

CATGCCAAATTGAG

>rnd-3_family-69#SINE/Deu ( Recon Family Size = 23 Final Multiple Alignment Size = 16 )

TTCACAGGGTTGGTTAACTCTTGAGGTCCCTTCGGTCTCCACCGAGCTAG

GTAAATCNGCCTTTAGTTTTNNTGCACCGTATTTCTGGAATAATCTNCAA

AATGCGTTTCGTNTTGATGTTCTGGTGCCGCTCGGGCAATTTAGAGTGTT

GATTGGGGACCTTTTTGCGGAGGAATGTTTTTCTGGGTGATTTGGTATGT

TTTATTTGTGCTTCCCGTNANTGTGTTTTTGTATTTTAATGTGTATATAT

AGTTTGGGTATNATGTGTACATTTATGTTGTATNATNCAGGGCTCGTCTG

TAAAAGAGACCTTGGTCTCAGTATGACTTCCCTGTTAAAATAAAGGTTAA

ATAAAATAACAAATTATG

>rnd-3_family-397#Unknown ( Recon Family Size = 18 Final Multiple Alignment Size = 16 )

TATGCTTCCCAGCCGAAACAGTTTGCGCTTTATGACTANATTATGACTTT

TTGTTCGTTTTGGTGTTCGGCAGTTTTTTTTTCGGCTGTTCGNGCGCACA

ATNTTTTTTNTGAGGCTAGCCGAAGTCTACGCCCCTTCGTCGGTGATTGG

TCGACAGTAGGGGTTCTTCAATGAAGTGTTTGTCATTCGATGAGAGACTA

GTTTTCATGCACATTTTTTCACTTGAGAAATACTGCACCAAACATCCTAG

TTAGATGTAAAATTGCGCGACTAAGACCTCCTCTGCAAAAACGTCAAAAT

TAATTACAGATTTCTTGATTTATCTTAGATTNATTCNGACTATTTTGAGG

AAGTGTATNCTGGCTATGTTGTTGCTACGGCGTCTCAAGAGGGACAAACA

GTAATATTGCCGCATTTTTTCTCGTTTTTCAAGCGAAGGTCTTTTAAGGG

AGTATGCGAGGCACAGACGTTCGCTTCGCCTAGCCGAGTTCTGCTAGGAG

CAAACCGAACCTGGTATGCGGACGCCT

>rnd-3_family-116#Unknown ( Recon Family Size = 19 Final Multiple Alignment Size = 15 )

ATAATGCCTTGCATTACGNCTGGGAAGAAAACACTTACATTTGCTCAACT

TGCCATTGGCTCAACTTACCCCAAGGCAAACATTTTGACTATATTAGCCC

ACACAGCTACAAGGATGCACTTTCATGCTAGGTTTAGGACCTCATATTGA

AGCTTATAGAGACCCCAACTGATGTATAGAACAATCTTAAAATGATCTAC

TTTGGTTTAGATACAAGCATCATGAAACCTCTAACACAATAAATTNATTT

GACTTGGTGAAAATCAGTTTTTTGGACCTAACTTGCTTACCATTTTTCCA

TGTGGTTTCTTCCTTCACAGACTCCATGAAATGATGACCTCTTCCTAAAT

ATTTGGTCAAATTATNAATTTTGTGTATGGTTTCCTAGAAACAAGGGTGG

CTCAACTTACCCCTTTGGCTCAACTTACCCCACTCTCCCCTA

>rnd-3_family-92#DNA/hAT ( Recon Family Size = 17 Final Multiple Alignment Size = 15 )

CAGTAGTGTAAAGTACTTAAGTGAAAATACTTTNAAGTACTACTTAAGAC

ATTTTTTGGGGATATCTGTACTTTACTTTACTTTTCGNTATCTTTACTTT

ACTTTGGGTTCGTCTTTTGACAACCTTTACTTTTACTTCACTACATTCNT

AAAGAAAATANTGTACTTTTTANTCCATACATTTTCCCTGNCACCCAAAA

GTACTCGTTACATTTTGAATGGCTAGCAGGACAGAAAAATGGTCCAATTC

ACGCACTTATCAATATAACGTCCCTGGTCATCCCTACTGCCTCTGATCTG

GCGGACTCACTAAACACAAGTGCTTCGTTTGTAAATTATGTCTGAGTGTT

GGAGTGTGCCCCTGGCTGTCCGTAATTGAAAAAAATAAAAAAATAGTGCG

GTCTGGTTTGCTTAATATAAGGAATTTGAATATTTATAGCATTTACTTTT

AGTATTCTTAAGTATATTTTAGCAATTACATTTACTTTTGATACTTAAGT

ATATTTAAAACCAAATACTTTTAGACTTTTACTCAAGTAGTATTTTACTG

GGTGACTTTCACTTTTACTTGAGTCATTTTCTATTAAGGTATCTTTACTT

TTACTCAAGTATGACAATTGGGTACTTTTTCCACCACT

>rnd-3_family-139#Unknown ( Recon Family Size = 19 Final Multiple Alignment Size = 15 )

AACAAACACTCAAACTGGACAGTTTTATCTCAATCTCTTCATTCAAAGAC

TCAATCATGGACACTCTTACTGACAGTTGTGGCTGCTTCGCGTGATGCAT

TGTTGTCTCTACCTTCTTGCCCTTTGTGCTGTTGTCTGTGCCCAATAATG

TTTGTACCATGTTTTGTGCTGCTACCATGTTGTTTTNCTACCGTGTTGTT

GTTGTGTTGCTACCATGCTGTGTTGTCATGTGTTGCTGCCTTGCTATGTT

GTTGTCTTAGGTCTCTCTTTATGTAGTGTTGTGTTGTCTCTCTTGTCGTG

ATGTGTGTTTTGTCCTATATTTATTTATTTATTTAAATGTTTAATCCCAG

CCCCCGTCCCCGCAGGAGGCCTTTTGCC

>rnd-3_family-248#Unknown ( Recon Family Size = 17 Final Multiple Alignment Size = 15 )

GTACTTCCATTCGTTTTTTCAAACTGGTACCGGGGGACCTTCAGACGAGT

CTTGTGAGGCCTGTGGGCGTCCTGGAGCAAAACAACCAAGACGTGTACGC

GTTCGGCCCGAAACTNTTCGGACGTTACGGACAGAAGTTGGCAGATCGGC

TGTACCGACTTCAGACGAGTCCCGAGACGCTTGTGGGGGTCGTAGAGCAA

AACGGAGAACACCATCGTGTTCGTGAGAGGCTCAAACNGTTCGGACGCTA

CACGATTTTCGGGATGTCTCATGGTCTGACAAACACCGCTCTAGCTCTGC

CACCTTTCACCGCAGATGCGGAAGCGCGACATAGGCGGATGCGGTGGATT

GAGACGCATCCAATGCAAANAACANATCTCTAGCTTAAACTGACA

>rnd-3_family-1091#LINE/L2 ( Recon Family Size = 16 Final Multiple Alignment Size = 15 )

AAAAAATAGCCTTTCGTGAACTAACACTCGCACTTGACTCTTCCCCCCTT

ANTAGCTCTGACTTTGCTGATAGCTACTTTATTGAGGAAAAATGTACTTA

CTATGACTGTGATATGTGGTTGTCTCACCTAGCTATCTTAAGATGAATGC

ACTAACTGTAAGTCGCTCTGGATAAGAGCGTCTGCTAAATGACTAAAATG

TAA

>rnd-3_family-618#Unknown ( Recon Family Size = 17 Final Multiple Alignment Size = 15 )

TAGGGCTCTATTCAATCCGTATCGCNGAAGTTCAGCGTTACAGCGCGATT

GAAATTTAAAGGCAATGTTCCCGCGTTNGCGGAGACTGCGTTCGCGGTAA

GACTGCATTCACGGTAAACGCTGCATATGTCGGCTCAATCGGAAATGACC

TTCACATTTCAACGCAGATCTTCCGCGATACTTCGGCGATACGGATTGAA

TCCAG

>rnd-4_family-1#LINE/Rex1 ( Recon Family Size = 7552 Final Multiple Alignment Size = 913 )

AGAAATCCCGCTATGCCCTCCGACGAACCATCAAACAGGCAAAGCGTCAA

TACAGGACTAAGATCGAATCGTACTACACCGGCTCCGACGCTCGTCGGAT

GTGGCAGGGCTTGCAAACTATTACGGACTACAAAGGGAAGCACAGCCGCG

AGCTGCCCAGTGACGCGAGCCTACCAGACGAGCTAAACGACTTCTATGCT

CGCTTCGAGGCAAGTAACACTGAAACATGCATGAGAGCACCAGCTGTTCC

GGACGACTGTGTGATCACGCTCTCCGCGGCCGACGTGAGTAAGACCTTTA

AACGGGTCAACACTCGCAAGGCCGCNGGNCCAGACGGATTACCAGGACGC

GTNCTCAGAGCATGCGCNGACCAGCTGGCNGGTGTNTTCACNGACATNTT

CAACCTCTCCCTGTCCCAGTCTGTNGTNCCCACATGCTTCAAGNNGACCA

CCATNGTCCCTGTGCCCAAGAANGCNAAGGTAACCGGNCTAAATGACTAC

CGCCCCGTAGCACTCACNTCTGTCGCCATGAAGTGCTTTGAGAGGCTGGT

CANGGNTCACATCANCNCCACCNTNCCNGACACCCTAGACCCACTCCAAT

TTGCATACCGCCCCAACAGATCCACAGACGACGCAATCTCCATCGCACTC

CACACTGCCCTNTCCCACCT

>rnd-4_family-7#DNA/TcMar-Tc1 ( Recon Family Size = 1437 Final Multiple Alignment Size = 694 )

AGTATTCAGACCCTTTGCTATGAGACTCGAAATTGAGCTCAGGTGCATCC

TGTTTCCATTGATCATCCTTGAGATGTTTCTACAACTTGATTGGAGTCCA

CCTGTGGTAAATTCAATTGANNNNNNNNNNNNNNNNNNNNNNNNNNNNNN

NNNNNNNNNNNNNNNNNNNNNNNNNNNNNNNNNNNNNNNNNNNNCCCAAG

CCATGAGGTCGAAGGAATTGTCCGTAGAGCTCCGAGACAGGATTGTGTCG

AGGCACAGATCTGGGGAAGGGTACCAAAACATTTCTGCAGCATTGAAGGT

CCCCAAGAACACAGTGGCCTCCATCATTCTTAAATGGAAGAAGTTTGGAA

CCACCAAGACTCTTCCTAGAGCTGGCCGCCCGGCCAAACTGAGCAATCGG

GGGAGAAGGGCCTTGGTCAGGGAGGTGACCAAGAACCCGATGGTCACTCT

GACAGAG

>rnd-4_family-92#Unknown ( Recon Family Size = 259 Final Multiple Alignment Size = 252 )

ACTGAACAAAAATATAAACGCAACATGTAAAGTGTTGGTCCCATGTTTCA

TGAGCTGAAATAAAAGATCCCAGAAATGTTCCATACGCACAAAAAGCTTA

TTTCTCTCAAATGTTGTGCAC

>rnd-4_family-134#Unknown ( Recon Family Size = 260 Final Multiple Alignment Size = 154 )

TGACTTCATCACTACTTGTNTTTGTGAGAGGTATTGACGTGTTGAANGCA

CCGAGCTGTCTGTTTAAACGACTGGCACACAGCTCGGACACCCACGCATA

CCCCACAAGACATGCCACCAGGGGTCTCTTCACAGTCCCCAAGTCCAGAA

CAGACTATGGGAGGCGCACAGTACTACATAGAGCCATGACTACATGGAAC

TCTATTCCACATCAGGTAACTGACGCAAGCAGTAAAATCGGATTTAAAAA

ACAGATAAAANAACACCTTACGGAACAGCGGGGACTGTGAAGCGACACAA

ACATAGGCACAGACACACGCACACACACACGATAACACACGCACTATACA

TACACACGGATTTTGTATTGTAGATATGTGGTAGTGGTGGAGTAGGGGCC

TGAGGGCACACAGTGTGTTGTGAAATCTGTGAATGTATTGTAATGTTTAA

AAATGTTTTAATGCTTTAAAATTGTATAAACTGCCTTAATTTTGCTGGAC

CCCAGGAAGAGTAGCTGCTGCTTTGATCCATAATAAATACAAA

>rnd-4_family-556#Unknown ( Recon Family Size = 156 Final Multiple Alignment Size = 127 )

TGACTATGTACAGACTCAGTGAGCATAGCCTTGCTATTGAGAAAGGCCGC

CGTAGGCAGACCTGGCTCTCAAGAGAAGACAGGCTATGTGCACACTGCCC

ACAAAATGAGGTGGAAACTGAGCTGCACTTCCTAACCTCCTGCCNAATGT

ATGACCATATTAGAGACACATATTTCCCTCAGATTACACAGATCCACAAA

GAATTCGAAAACAAATCCAATTTTGATAAACTCCCATATCTACTGGGTGA

AATACCACAGTGTGCCATCACAGCAGCAAGATTTGTGACCTGTTGCCACG

AGAAAAGGGCAACCAGTGAAGAACAAACACCATTGTAAATACAACCCATA

TTTATGTTTATTTATTTTCCCTTTTGTACTTTAACCATTTGCACATCGTT

ACAACACTGTATATANACATAATATGACATTTGAAATGTCTTTATTCTTT

TGGAACTTCTGTGAGTGTAATGTTTACTGTTNATTTTTATTGTTTATTTC

ACTTTTGTTTATTATCTACTTCACTTGCTTTGGCAATGTNAACATATGTT

TCCCATGCCAATAAAGCCCCTTGAATTATA

>rnd-4_family-248#Unknown ( Recon Family Size = 128 Final Multiple Alignment Size = 108 )

ACACTGAACAAAAATATAAACGCAACATGCAACAATTTCAAAGATTTTAC

TG

>rnd-4_family-847#Unknown ( Recon Family Size = 140 Final Multiple Alignment Size = 106 )

GACCCGTTTCAGGAAACTAGGCGTATGTCGCACGTCACTACTTCACAGGA

GAGCCGTTTGAACGTAACATTTTTATCAAAATGCGTTTTTTGGCAGAAAT

GCCTTCTNGAACATGTGAACTTTCATGTGCCTTAATAACAAACTTGTATG

CCATCTGTAAATACGAATAAAATTGTTAAATTACGAGCCTAGTTGGTTTA

GCCACGGAAAAAGNCAGCAACCTTCCCGCTAGCCATGATTGGCTGAGATA

ATGAGTGGGCTGGACATGCCGAGAGATGAGTTCGGATTGGTCTGCCATGT

AGCACGCTTCTGTCTATAAATGAGCTGGTCAGTATGTGTAGGTAATCCTN

TCTAACGCGGCTTTTTTTAAAGATATCGAAGTGTTGCTCTCCACTTTCTG

GAGGACCGAGTTTTGAAATCAGTGGAATTAGAGTATGATAGCTAAGGAGA

TGGAGAAATTCTGGCGTTTGATTGCAAATATGCAGAGGGAGTCGAAAAGA

GAACACACAGAAGGCTGTTGTATAAAACACCTGTCTCCGGATTAC

>rnd-4_family-196#Unknown ( Recon Family Size = 91 Final Multiple Alignment Size = 89 )

ATTGTCTAGCCCGGCTGATTTGTAGGGGTCCAGATTTTGCAGCTCTTTCA

GAACATCAGCTGTCTGGATTTGGGTGAAGGAGAAACGGGGGGGGCTTGGG

CGAGTTGCTGCGGGGGGTGCGGGGCTGTTGACCGGGGTAGGGGTAGCCAG

GTGGAAAGCATGGCCAGCCGTAGAGAAATGCTTATTGAAATTCTCGATTA

TCG

>rnd-4_family-138#LINE/L2 ( Recon Family Size = 214 Final Multiple Alignment Size = 89 )

AGAGATGCTTGTTCTAGGTCCCAAGAAACAAAGAGATCTTCTGTTGAATC

TGACAATTAATCTTGATGGTTGTACAGTCGTCTCAAATAAAACTGTGAAG

GACCTCGGCGTTACTCTGGACCCTGATCTCTCTTTTGACGAACATATCAA

GACTGTTTCAAGGACAGCTTTTTTCCATCTACGTAACATTGCAAAAATCA

GAAACTTTCTGTCCAAAAATGATGCAGAAAAATGNATCCATGCTTTTGTT

ACTTCTAGGTTAGACTACTGCAATGCTCTACTTTCCGGCTACCCGGATAA

AGCACTAAATAAACTTCAGTTAGTGCTAAATACGGCTGCTAGAATCCTGA

CTAGAACCAAAAAATNTGATCATATTACTCCAGTGCTAGCCTCCCTACAC

TGGCTTCCTGTTAAGGCAAGGGCTGATTTCAAGGTTTTACTGCTAACCTA

CAAAGCATTACATGGGCTTGCTCCTACCTATCTCTCCGATTTGGTCCTGC

CGTACATACCTACACGTACGCTACGGTCACAAGACGCAGGCCTCCTAATT

GTCCCTAGAATTTCTAAGCAAACAGCTGGAGGCAGGGCTTTCTCCTATAG

AGCTCCATTTTTATGGAATGGTCTGCCTACCCATGTGAGAGACGCAGACT

>rnd-4_family-778#Unknown ( Recon Family Size = 96 Final Multiple Alignment Size = 88 )

TATATTCTTCAAGAATCAATGGGTACATATCATTAATTTATAAGTCCAAA

AATGGATGTAGCAACTGCAGATTGCCCC

>rnd-4_family-190#Unknown ( Recon Family Size = 95 Final Multiple Alignment Size = 88 )

AAAGGATAAACATTCAACATTGGCCATGCTGTCAATCCAGCATGACTTCT

GCCGCGCTCAAAACAACTGGAAACTCGGAACTGGGAAATCTCAGACTTCA

GTGAGTTCAAGACAACTGGGAACTCGGAAAAAAACGAGCTCCGACTGGGA

AAATACGTTTTGAACGGTCATCCAACTCGGAATTNCAAGTCGGGAACTCG

GGCCTCTTTCTAGAGCTCCGACCTGAAGATCACTGACGTCATCAGATTCG

ACCTTGTTTTTTTTCCGAGTTCCCAGTTGTCTTGAAAGCACCATAAATCC

AGAGAATGCCAGACTTTGATGACAAAGTTTGCCCACGAAGGACCGCCGCG

CCACCTTCCTGTTCAAGTGAGCACAAGGTGAGTCCAAAAATGTCTTGTAT

GCTGCTGCATAAATGATGTAATATGCCAGGGAGATATGTATACTGTAGCT

AAGAAAGTAATACTAAGTGTATGTTGTGTAGTAAGCTGTTA

>rnd-4_family-350#Unknown ( Recon Family Size = 98 Final Multiple Alignment Size = 87 )

TCTCAACGTCAACAGTGAAGAGGCGACTCCGGGATGCTGGCCTTCTAGGC

AGAGTTGCAAAGAAAAAGCCATATCTCAGACTGGCCAATAAAAAGAAAAG

ATTAAGATGGGCAAAAGAACACAGACACTGGACAGAGGAACTCTGCCTAG

AAGGCCAGCATCCCGGAGTCGCCTCTTCACTGTTGACGTTGAGACTGGTG

TTTTGCGGGTACTATTTAATGAAGCTGCCAGTTGAGGACTTGTGAGGCGT

CTGTTTCTCAAACTAGACACTCTAATGTACTTGTCCTCTTGCTCAGTTGT

GCACCGGGGCCTCCCACTCCTCTTTCTATTCTGGT

>rnd-4_family-926#Unknown ( Recon Family Size = 113 Final Multiple Alignment Size = 80 )

GCTTTGGAATGTGTTGGGTGGACGGGAGGAGAGCACCGTGTTGATACAGG

GGAGACGGATGGACATCCGTGGATTAGGTGAAGGAGGGGTTGAGGTCAGG

AGGTGAGGTCAGGTCACCTTAAGGGAGNAATAAGGGTTTGGTATCCTGTT

ACCCTCTTCCTGCCTAGCAATAAGATGTAATGNTTGGAGAGAAGGAGACT

CGGCCTAAGTGGGATGTANATANCTGTGATGGTGNGAAATGTTTTGTGTC

TGAATACAGCTGTATCGANCCTTTGGGAAGAATTAAACTTGGTTTAAGCT

TTTCTAGTGTCCGTGAGTTATTTACTCTGAAAAATAAGAACCT

>rnd-4_family-334#DNA/TcMar-Tc1 ( Recon Family Size = 80 Final Multiple Alignment Size = 78 )

ACCGAAGAATTTCTGCACAAACTGTCAGAAACCGTCTCAGGGAAGCTCAT

CTGCGTGCTCGTCGTCCTCACCAGGGTCTTGACCTGACTGCAGTTCGGCG

TCGTAACCGACTTCAGTGGGCAAATGCTCACCTTCGATGGCCACTGGCAC

GCTGGAGAAGTGTGCTCTTCACGGATGAATCCCGGTTTCAACTGTACCGG

GCAGATGGCAGACAGCGTAGTGT

>rnd-4_family-176#LINE/Rex1 ( Recon Family Size = 116 Final Multiple Alignment Size = 72 )

CGCCCCCAGGTGGTGAGGGTAGGNAACAACACCTCCGCCNCGCTGATCCT

CAACACGGGGGCCCCTCAGGGGTGCGTGCTCAGCCCCCTCCTGTACTCCC

TGTTCACCCACGACTGCGTGGCCAGGCACGACTCCAACACCATCATCAAG

TTTGCCGACGACACAACAGTGGTAGGCCTGATCACCGACGACGACGAGAC

AGCCTACAGGGAGGAGGTCAGAGACCTGGNAGTGTGGTGCCAGGACAACA

ACCTCTCCCTCAACGTGAGCAAGACAAAGGAGCTGATCGTGGACTACAGG

AAAAGGAGGACCGAGCACGCCCCCATTCNCATCGACGGGGCTGTAGTGGA

GCGGGTTGAGAGCTTCAAGTTCCTCGGCGTGCACATCACCGACAAACTGA

AATGGTCCANNCACACAGACAGTCGTGAAGAAGGCGCGACAGCGCCTCTT

CAACCTCAGGAGGCTGAAGAAATTTGGCTTGGGTCCTCAGACCCTCAAAA

GGTTCTACAGCTGCACCATCGAGAGCATCCTGACTGGTTGCATCACCGCC

TGGTATGGCAACTGCTCGGCCTCCGACCGCAAGGCGCTACAGAGGGTAGT

GCGTACGGCCCAGTACATCACTG

>rnd-4_family-44#LINE/L2 ( Recon Family Size = 72 Final Multiple Alignment Size = 69 )

TTCAACTTTTCCCCCGATGCCTTGAGATGGATGAAATCATACCTTGAAGG

CAGAACTCAGTGTGTCAGAGTGAGCAATGAGCTGTCGCCCACTCTTAGCT

ATGATGTGGGCGTGCCCCAAGGGTCAATACTGGGGCCCCTCCTGTTCAGC

CTGTACATTAATGATCTGCCTTCTGTCTGTACTGGGTCTGAAGTTCAAAT

GTATGCAGATGATACAGTGATATATGTGCATGCAAAGAGCAAACAACAAG

CTGCACAAGAACTCACTACTGTAATGGTCCAGGTTACAAAGTGGCTCAGT

GACTCGTGTTTGCATCTCAATGTGAAAAAAACTGTTTGCATGTTCTTCAC

AAAGAGGGCAACAGATGCTACTGAGCCAGATGTCTATGTGTCAGGGGAGA

AGCTCCAGGTGGTATCCGATTTTAAGTACCTTGGCATCATACTTGATTCC

AACCTCTCTTTTAAAAAGCATGTGAAAAAGGTAATTCAAATAACCAAATT

CAACCTAGCTAATTTCCGATTTATACGAAATTGTTTGACTACAGAGGTAG

CAAAACTGTACTTCAAATCTATGATACTCCCCCACTTAACATACTGCTTG

ACTAGTTGGGC

>rnd-4_family-142#Unknown ( Recon Family Size = 76 Final Multiple Alignment Size = 65 )

TGACACGTCAAATAGTGTTATTTGACGTGTATCTTTTTTGACACGCAAAG

ACCCAAACGGCGTTCCATAGAAATCCTGGTTGAGAATGAAACGACTGAAC

AAATGAACAACGAAACAGCACAGCAAGTAAGTGAAAGAAATAGGTTTTGA

TTATGTTTTACTGGTAATGGGGACATACGTAAATGCCAACAAAATAACTT

TTTGGTCAGTGTGTGTGTGTGTGCTTTATTTAACTAGGCAAGTCTTATTT

ACAATGATGNCCTACTCCNGNNNNNNNNNNNNNNCNNNNNNNNNNNNNNN

NNNNNNNNNNNNNNNNNNNNNNNNNNNNNNNNNNNNNNNNNNNNNNNNNN

NNNNNNNNNNNNNNNCNCTCNNNNCTCTTGCACTGAGATCTTCCNNAANA

CAGACCGTGTGCGTGCGTGTGTGTTAACTATTTAACTGTACTAGAATGCT

TAAAAGGCCGCTAAAATTTTAAATATCGGTTATCGGTTTTTTTTGTTGGC

AAGGAAAATATCGGATATCGGTATCGGCCAAAAATGTCATATCA

>rnd-4_family-4#Unknown ( Recon Family Size = 93 Final Multiple Alignment Size = 65 )

ACAGTCACTCAATTAGCCCATGTCAGCTAACAATTTTAGATTGGTAGNTT

AGTCTAGCCAGCTATCTAACTTGTAGTAATCGTGGCCGAATTACCGACCG

GGCACGCAGGGCACGTGCCCAGGGGCCCTGACCTCCAGGGGGCCCCATTG

ATTTTGTTAGTCACTCTCACTCAGATATCATATTAACATGGCATTAAAAC

TGCAAANATTTCTCTCCGCCCCATGGCAAAATGTGTAGAATTGCAGGAAA

TTAGCTTTAAAACTGCAACATTTTCTCTCCGCCCCATGGCAAAATGTGTA

GAATTGCAGGAAATTAGCTTTAAAATGCAAAATTTGTCTCTACGCCGCCA

AGTGGGGGA

>rnd-4_family-78#Unknown ( Recon Family Size = 171 Final Multiple Alignment Size = 63 )

GTTCACCAACCAAGGCATTCCTAGTCGATCACCAAACATTTCTGTAAAAA

ACCACGATAAAGCCTATTTTTTTTGTATTCGTTTCGCGCTGTTGGCGGTA

GGTGCACTTGATTCAGCAGCCCTAGCGCCGGGAAGGCAAAGTGTTCCCAT

TTTGAACCATTTCATGTGTCTGAAGGTAGAACTCCGCCTACCCGGCGGGC

CCAGAGAGCAAATCAAGTGCACCTATAGGCCTACCGCTGGCCAATCAGAT

AGCTCAGATCACCGTGTCTGCACAGTTTCCTCGAGCCATAGACTGTAAAA

AGAAGCCTCGAACGCACAGCAAAGTTGATACTGTGAGATTTCAAAACTTT

TAAAACCATGACTAGAGAGAGACTCAACGAATACAGCAAAGAGCTGCTGT

TTTTATGAGTAAGTTCAAGTTNTTATTCAGCACTGTCAACACTTTGTTCA

ACACTTTTATAAGCCATAAAATGCGCGTTCTCCCTACTTCCACTCACGCT

ACAACCAGCACTGCAGCTGCAATGAATGAGTANAGCAAAGTGTTCCG

>rnd-4_family-74#Unknown ( Recon Family Size = 66 Final Multiple Alignment Size = 63 )

CCAAGTCAATAAAAATAGCAGCACAACATTGCTTAGAATCAAGGGCAATG

GTGACATCATTGAGGACCTTTAAGGTTGCAGTGACACATCCATAACCTGA

GCGGAAACCAGATTGCATACCAGAGAGAATACTATAGACATCGAGAAAGC

CGGTCAGTTGATTATTGACANGTTTTTCCAACACTTTTGATAAACAGGGC

AAAATAGAAATTGGCCTATAACAGTTAGGATCAGCCCCTTTTAATAAAGG

ACGNACCGCGGCTGCCTTCCAAGCAATGGGAACCTCCCCAGAGAGGAGAG

ACAGGCTTGGCGATGATAGGGGCAGCAACCTTAAAGAAGAAAGGGTCTAA

ACCATCTGACCCAGATGTTTTTTGGGGGTCAAGTTTAAGGAGCTCCTTTA

GCACCTCGGACTCAGTGACCGCCTGCAGGGAGAAACTTTGTAGCGGGGCA

GGGGAAAAAGAGGGAGGAGCATCGGGGNTAGTCGCATTAGAAGGGGTGGG

AGATGAGGAAATGTTGGACGGGCAAGGAGGCATGGCTGAGTCAAATAGGA

ATCCTGACTTAATGAAGTGGTGATTAAAGAGCTCAGCCATGTGCTTCTTG

TCAGTAACAACCACATCATCAACATTAAGG

>rnd-4_family-93#Simple_repeat ( Recon Family Size = 104 Final Multiple Alignment Size = 59 )

TCCAAGATGGCGTAGCAGTCGGACGTCTTTGTCCTTCGTCTTGTCGTGTC

CCGTGTATATATCTTTTTACATATTTTTCTTCGCATATATTTTTTTATAT

TTTTCTAAACCTCAACTTCAAAATACTCTCCTGCAACCCGCCTCACCCAA

TGTGGTACGGATCTGCTATTTTAATATTTTTATTTACCTCGGAACCGGAA

CCCCCCAACAGAAGCTAGCCAGCTAACTAGCTACTAGCTAGTAGTCAGCT

AACCACCGCTAGCGGTCATCAGCTAACCTTTAGCTCGGAAAGCTCTCGCC

AGTTCGNACAACGCGACTCGAACCAGAGCATACCGGACCTATTTTCTCTC

CATATCCCCGGATTCCTACCGCAAGCTCTGGACNTTTNCACCTGGATCNT

CGCAGCTAGCTAGCTGCTATCCGAGTGACTACTCCTGGCTAACGTCTGTC

CCGGAGCAAGCACCAATTAGCCCGGAGCTAGCCCGGCTAGGCCCATCTCC

CGGCT

>rnd-4_family-418#DNA/TcMar-Tc1 ( Recon Family Size = 72 Final Multiple Alignment Size = 58 )

ATTCATTTTTTTTGTCCTGAATACAAAGCGTTATGTTTGGGGCAAATCCA

ATACAACACATTACGTAAGGACAAACCTAAAACNTAAGACCAAATCTACA

CTGTAGTTGCTTACCAATAAGACAGGGAATGTTCCTGAGTGGCTTTAAGT

CGACGCTTGGAAACGGTTGTCTAGCAATGATCAACAACCAATTTGACAGA

GCTTGAAGAATTTTGAAAATAATAAATGGGCAAATGTTGCACAATCCAGG

TGTGGAAAGCTCTTAGA

>rnd-4_family-435#Unknown ( Recon Family Size = 57 Final Multiple Alignment Size = 54 )

GTTGGACACTAAGAAAGCTTTGTTGTAGAGCGTTTAACACAAAATCCGGG

GAGGGGCCAGCTGAGTATAAGACTGTATCATCTGCATATAAAT

>rnd-4_family-302#Simple_repeat ( Recon Family Size = 54 Final Multiple Alignment Size = 53 )

ATGATACGATTTCCAGCATTATCTCAGTATTCCAAGGCATTGNNNNNNNN

NNNNNNNNNNNNNNNNNNNNNNNNNNNNNNNNNNNNNNNNNNNNNNNGGC

CAATGCAGGATGTTTTGAACATGTAATTTCGAG

>rnd-4_family-349#Unknown ( Recon Family Size = 66 Final Multiple Alignment Size = 52 )

GTGTATATAGGACTTGCATCCTTGGCACAATAAAGTTATTATATTCTACT

CAATCCATAGGCTTCATTAATGCCATTCAGACTTGGTTCATGGATTGGTA

TGAATATTAGTTCAGAACCNAACGTTTTAGGGTCCATACACAGATCGGCT

ACAGTTATTTTTAATCCGACACTAAGTAAATAGCTAGCTACGTTACTTCA

GCTGCAAGGCAAGCTTACATTCAATTGTGCAGTAAATGCTTTAGCTAGCT

AGATTCTTTACATCCACTGTTTCACAAGACGACAGCCTGGTTTGCTGGTT

CTCAGGAGTCCCTAAAAGACAAATTCATTTTCGTAANTTAACTTTGACAA

AAAAAAAGCCGCACAAACGTCTCTACATTAACACATTCCTCAAATTGTAC

ATTTGCTTGACTCGTTATGACAACTTCCATCTATTTTGTCAGCAATCTTC

GTTGAGCGCCACAAGGCAAGGGTGGCTCGCGTCAAAATTCGTCATTGGTC

ATATAAAGCCTTGGGACCCAAATGCATAATGAGTGCTCTAACTCCCCCTT

GTGGTGGTCTGGAGCAATGAAGCCGTCCCGCGGTGCAGGCTCGCAACTTT

TAAAGGAGTGTGTANATTCAGTCCCCGCTGTTCCTTACGGTGTGTGTTTA

>rnd-4_family-411#LINE/L2 ( Recon Family Size = 72 Final Multiple Alignment Size = 51 )

TCACTACTGGTGTCCCCCAGGGCTCGGTTCTAGGCCCTCTCCTCTTCTCT

CTATACACCAAGTCACTCGGCTCCGTCATATCCTCACATGGTCTCTCCTA

TCATTGCTATGCGGATGACACTCAACTACGCATCTCTGCGTGCCTGGCAG

ATATCTCAGCTTGGATGTCGGCCCACCACCTCAAGCTCAACCTCGACAAG

ACGGAGCTGCTCTTCCTCCCGGGGAAGGCCTGCCCGCTCCAAGACCTCTC

CATCACGGTTGACAACTCCACGGTGTCCCCCTCCCAGAGTGCAAAGAACC

TTGGCGTGACCCTGGACAACACCCTGTCGTTCTCTGCAAACATCAAAGCA

GTGACTCGCTCCTGCAGGTTCATGCTCTACAACATCCGTAGAGTACGACC

TTNCCTCACACAGGAAGCGGCGCAGGTCCTAATCCAGGCACTTGTCATCT

CCCGTCTGGACTACTGCAACTCGCTGTTGGCTGGGCTCCCCGCTTGTGCC

ATCAAACCCCTGCAACTTATCCAGAACGCCGCAGCCCGCCTGGTGTTCAA

CCTTCCCAAGTTCTCCCATGTCACCCCGCTCCTCCGCACACTCCACTGGC

TTCCAGT

>rnd-4_family-153#Unknown ( Recon Family Size = 100 Final Multiple Alignment Size = 50 )

AACTTGCAACATTGTATAAAATATTCTGGGCCCTCAGTTTCCCGCGCCAG

TGAGCTCNGGACAGACACAGCTGTAGGCTATTTGCGCAAGGGATAAGAAG

CAGTGCTTGACTTGGGCAGGAGCTCACCGGAGCCGAGTACCGGCACCTCA

AATGTTCTACTGCTTGAGCTCCTGTTCTTATAGAATATTAGCTCAAAAGT

ATTGTGGAGCTCCTGCACCTAAATATAAACAGTACCGGCACCCAAAATGA

GTACCGGAACCTATTTCAGTCCAAGTCAAGCACTGATAAGAAGTAATCAG

GTAGGCCTATTTTATGACGTTTCCACTGGATCAGAGCATGACATTTTTCC

CTTTCACGCCGAGTGGTTATCGAAAGGGAGAGAGCTGGAAAGATTTTTCA

AATACATTGAGGAACTATTGTCATTCTCAATGGATGTAAAAACAGACTTT

GTTTGCTTGCTGTTTGAGGTGAAGAAAACATTACTTTGAGAAGCTCCACA

GCTCATTAGTGGTGGTGCGTTAAGCCAATCAGAAATACTATCAGATCCCC

AAATGGGCACATTTATAGGCCTACATTTGCGCGCAGGCCAGGTAGCCTAT

AGGCCTACTTCTATGCGCGATCAGGTGCGCGTCCT

>rnd-4_family-487#DNA/TcMar-Tc1 ( Recon Family Size = 52 Final Multiple Alignment Size = 50 )

CATAGGATGACAATGCCCCCATCCACAGGGCACGAGTGGTCACTGAATGG

TTTGATGAGCATGAAAACGATGTAAACCATATGCCATGGCCGTCTCAGTC

ACCAGATCTCAACCCAATTGAACACTTATGGGAGATTCTGGAGCGGCGCC

TGAGACAGCGTTTTCCACCACCATCAACAAAACACCAAATNATGGAATTT

CTCGTGGAAGAATGGTGTCGCATCCCTCCAATAGAGTTCCAGACACTTGT

AGAATCTATGCCAAGGCGCATTGAAGCTGTTCTGGCTCGTGGTGGCCCAA

CGCCCTATTAAGACACTTTATGTTGGTGTTTCCTTTATTTTGGCAC

>rnd-4_family-479#DNA/TcMar-Tc1 ( Recon Family Size = 57 Final Multiple Alignment Size = 49 )

GCTATGGTGATATGAGCTGAAGATAGAGGTCTATGGCCACGCACAAAAGC

GATGGGTTTGGCGTCGAAATGAGAATGCATNAGCAGAAAATAACCCCATA

CCTACTGTAAAATATGGTGGTGGATCTTTGATGTTATGGGGCTATTTTGC

TTCCACTGGTCCTGGGGCCCTTGTTAAGGTCAACGGCATCATGAACTTTA

CCCAGTACCAGGACATTTTAGCCAAAAACCTGGTTGCCTCTGCCAGGAGG

CTGAAACTTGGCCGCAAGTGGATCTTCCAGCAAGACAATGACCACAAAGA

AATGGTTAATTGGCCGCAAAATCAACGGTCTCCGGACTTGAACCCCATTG

AAAACCTGTGGTTTGAATTGAAGAGGGCAGTCCATAAGCGCAGACGAAGG

ATATCAAGGATCTGGAAAGATTCTGTATGGAGGAATGGTCTAAGATCCCT

CCCAATGTGTTCTCCAATCTCATAAAACATTNTAGAAAAAGGCTCAGTGC

CGTTATCCTCGCAAGGGGAGGGTGCACGAAGTATTGAAAA

>rnd-4_family-974#Unknown ( Recon Family Size = 51 Final Multiple Alignment Size = 48 )

GGTTCAGAGTTTGTTTTGATATTTCAACCTGCGTGTCGTGATCGCGTTTG

GTGTGGGGGGACAAAATNAACGTGCGCACGATGGCGCACGCGCGCAGCCG

GTTTGGGTTCCG

>rnd-4_family-1226#Unknown ( Recon Family Size = 50 Final Multiple Alignment Size = 48 )

TATGCAGGTTTAAAAATATATACTTCTGTGTATTGATTTTAAGAAAGGCA

TTGATGTTTATGGTTAGGTACACGTTGGTGCAACGACNGTGCTTTTTTCG

CGAATGCGCTTTTGTTAAATCATCCCCGTTTGGCGAAGTAGGCTGTGATT

CGATGATAAATTAACAGGCACCGCATCGATTATATGCAACGCAGGACACG

CTAGATAAA

>rnd-4_family-43#Unknown ( Recon Family Size = 86 Final Multiple Alignment Size = 47 )

ACCTCACTCCGGGGCGTGGCTACTCACTGTGCAAAGNTATATGANAACAA

GTTATCTCTTATAANTCCTAAAATCACATTCNTATCTTAACAAAAATACT

TTCATCATTNTTCATATTATATGCACAACGTATCGGATGNAAACTTGACA

NATAAAGTGGGTATACTTTCCAAGTTACAGTATTTCGTTCATACCGTTTT

TAATGACATCACAAAATGANAAACAATATGACATTAGTNTTCTATANCTC

CCCACTGACCATTCCCCACGTTCTTACGTTAGAAATATTGTTCCATTATT

CACTTTTTGAACGTTAGAGTTTTGGGCGGCAAAAGTCTCTCTAGACAAAA

GCATTCCTTTGGTCGATACTAAAGGGNAGAGAGAGAGTCCTCTCCTGCTT

AAATTTACGACCGGGCGTGGAGGTGTCAAAACCCCCACAGTTCCCTCCTC

CCCTCTTCTGCGGGTGAGAGAGGGTCTGGTTATTGTCANCCATTGCCAAG

CTGATCTGACCCATTNTGATCCTCACAGGA

>rnd-4_family-230#Unknown ( Recon Family Size = 50 Final Multiple Alignment Size = 47 )

TAGGGGGTAGATCAGCTTTAATATTGCAGATAGATTGTGGCTTCCATCAA

TGTAATTGTCTGCATCATTTCCAATCCCCCATATATTTTTTTGCAAAT

>rnd-4_family-270#Unknown ( Recon Family Size = 47 Final Multiple Alignment Size = 46 )

TGTCGGGACGGAAGGCGTCTTCCTCCACCTCCCCTCTTCCATACATTTGG

GTTACCAGGTGCATATGTAAGCGACAGGAGCCAGAGTCCAGAAGCCCATA

GAGAGAGTGGGTAATCGGACTAAGGAAGGTGAGTACTAGGCTGAAAAGTA

CCACCGGTACCGGTTAACCCAAAGGCCCAAGAGAGAGTGGGCAACCCAGA

GTCCGATATCCCAAAAAGAGAGTGGGTAATCGGACTAAGGGAGGTGAGTA

CTAGGCTGAAAAGTACCACCGGTAACCCAGTGTGGACTTAGGCTCTGGGC

GGAAAGCGTCCNNNNNNNNNNNNNNNNNNNNNNNNNNNNNNNNNNNNNNN

NNNNNNNNNNNNNNNNNNNNNNNNNNNNNNNNNNNNNNNNNNNNNNNNNG

TGATTAAGGGCCTGTACTCTCATGCCAGAGAGGGAGGCCTGTTACTGGAT

AGGTAGTGAGGGCCTTTAGGCCTGAAGGTCCATAGTTCCACTGTCCTTAA

GGGGGGCA

>rnd-4_family-315#Unknown ( Recon Family Size = 109 Final Multiple Alignment Size = 46 )

AAAACAGCAAATTAAAAACATTGACAGGTCAGGGAATCAGCCTCAAGATC

ATTCATCAGTGATTTAAAAACACCAATCGGGACAAGTTCTTCCAGTTTAA

AAGTATTTTGTTAGCAGGATAAAGTCCAGCGAACGAAGAGAGTACCCACC

ACATTTCTGAACAATAAAAATGCCCAAATAAAAAGGTAGTAAACCCAAAA

TGGCTTTGTAAATAAAAGTATACCAGTGACTGAGCCTACGAGTGACTAGA

GAAGGCCAGCCAACCCTGGTATACAAAGTGCAGTGGTGCGTAAGGGTTTT

GCAGTTTAAAATAAATCTCAAAGCGCCATGGTAAAGGGCGTCAATTGATC

TCAAACACTGAGCGGAAGCATTCATATATAAAATATCCCCATAGTCTAGT

AAAGGCATAAATGTAGCTGATACTAGCCTCCTTCTGGCTTCAAAAGAAAA

ACAGGCCTTATTCCTAAAATAAAATCCCAATTTCAGCTTCAATTTTTTTG

TAAGTTGTTGAATATGCAATTTAAAAGAGAGGCCGTCATCAATTAAAATT

CCAAGATATTTATATGAGGTTACAGCCTCAATCTCNTTGCCCTGACAGGT

AGTAATAGGTGAAAGGTTCAGAGGTCTATTTCTTGCTTTAGAAAACACCA

TTAGTTTAGTTTTGTCAGTATTGAGGA

>rnd-4_family-533#Unknown ( Recon Family Size = 46 Final Multiple Alignment Size = 45 )

ACGGTCGGTGCCGTTTAAGATGAGGGAGGACGATTTTTTTTCATGAGCAT

GGCCTTATTTCTATTACAGCATATTGGATGACTGTCATTCATATTCCATT

CACCCAGCTCAATGTAACATCGATAGGTTTAGGCTACTACATGATACTCG

AATTTTCCCTATACCCATCATGAGGTTGCTACAACCTA

>rnd-4_family-156#Unknown ( Recon Family Size = 59 Final Multiple Alignment Size = 45 )

TCGTCCCAGATCTGTTTGTGCTGTCTTGCCAACTCCTATGGTCATTGTCA

TGCCAAACGACCATAGGAGTTGGCAANACAGCACAAACAGATCTGGGACC

AGGC

>rnd-4_family-60#DNA/TcMar-Tc1 ( Recon Family Size = 46 Final Multiple Alignment Size = 43 )

GTACACCTGTTCTGAAAGGCCCCAGAGTCTGCAACACCACTAAGCAAGGG

GCACCACCAAGCAAGCGGCACCATGAAGACCAAGGAGCTCTCCAAACAGG

TCAGGGACAAAGTTGTGGAGAAGTACAGATCAGGGTTGGGTTATAAAAAA

ATATCNGAAACTTTGAACATCCCACGGAGCACCATTAAATCCGTTATAAA

AAATGAAAGAATATGGCACCACAACAAACCTGCCAAGAGAGGGCCGCCCA

GGCAAGGAGGGCATTAATCAGAGAGGAAACAAAGAGACCAAAGATAACCC

TGAAGGAGCTGCA

>rnd-4_family-488#Unknown ( Recon Family Size = 44 Final Multiple Alignment Size = 43 )

AGGTCACCCGTGATACAAGTCAGTATTCGACGTCCATCCATGTCTGAGGA

CGTCGGGAGATGACGTGGAAACCGGCCACTAGGGGCAACAGTGAGCGCTG

TTACCTTCAAGTAGGTTTCGGTTTTGCTAGGGCGTTGTGGACGGGGATGG

CGGATGGGCGTAAGCATCTGCCTCTGATTGCGTGTTCGAATCCAGCGATA

GAAAGTTGTTTTTGATGTTTTTGTTTTAAGCCTATCCCAAACCTTAACCC

TTACCTTAACCATTCGGAGTTAATGCCTAAACTTAACCCTAACCTTAAAA

ATTCGGAGTTAATGCCTAAACTTAACCTTAAAAACTTCGAAATTTGACGT

TTGAGAAACGTGGATGAACGTCTAATTCTGACGTGAGACTGTGAGAGCTN

GTT

>rnd-4_family-182#Unknown ( Recon Family Size = 67 Final Multiple Alignment Size = 43 )

AACTTATAGCCTCTGTCTCTTGCGCAATACGCACGCACCTGCGCTCCGCT

GGCCTCTCTCCTTAAAAAACGCTCCTTAGCTCTTGGAGTCCCATGCAGGA

AAAGCTAGAATAGCTTGCTGGACTTTATTTTTTTTNTTAGACTATTCGAA

GAGGGACGCAAGCTCTTGTTTGCTGAATGTTAAATACAGCAGCCAATAGA

ACTCAGGGTAGTTTGAAAGAAGAGCGAACGCGCGATGGCGGTAGGCTATA

GCAGTTATTTATTCAGACCCATAACCATTCAATCTTCGTGAAGAGAAGTG

AAAGCCTTCTGCATCTAATTCTAGTCCTATATTATTCAAGGATGTCATTA

GAATGATGAAGATAAGGACAACGAAACTTATTTCATTTAACTGTTGAAAG

CGAGGAAGGAATGAAGCAACAATAGAGAGAGAAGGAGGTAGGCTAATAAC

ATTAGGGGAAATATTATGAAAGGTATATATGCGTCAGCCTACTAATTATA

CAAATAGGCCCTATTATATTTCAAAATTAAAATCGAATTCGCTAGCCTAT

ACTTGTAACTTCGTAGGCCGCATGTGCTGCACCAGAATCGCATGTTCTCT

CCCTGCTTTATCATGGTTTGAACGATGTGCGTAATTCC

>rnd-4_family-1495#Simple_repeat ( Recon Family Size = 71 Final Multiple Alignment Size = 43 )

TAAACGTAAATCCGGGACACTCAAATTAGTATGATATGTTACGTTTGGTA

TGGTTACATAAGACAGAAGGTTACTTAAAGGCGAAAAGAAAGTAGGGTGG

TTGGGTGGGCGTATAACGCGAACGTCTAGCAACCCAAAGGTTGCGNGTTC

GAATCTCATCACGGACAACTTTAGCAAATTAGCAACTACTTACTACTTTT

TAGCTACTTTGCAACTACTTAGCATGTTAGCTAACCCTTCCCCTAACCCT

AACCCTAACCTTTTAACCTAACTCCTAACCCTAACCCTAACCTTAACCCC

TAGCCTAGCTAACGTTAGCCACCTAGCTAACCGTTAGCCACCTAGCCACC

TAGCCACCTAGCTAACGTTAGCCACAACAAATTGGAAT

>rnd-4_family-1815#Unknown ( Recon Family Size = 46 Final Multiple Alignment Size = 42 )

GTGGCGATTTTAGCATGTAAATCTTGGTGGGGCAAAAAAATAATTGTGGG

ATGCATGCCAGCAAAGCCACTACACAACACAACACTAAACAATACATTAA

TTGCACTATAACGGTGACAAACGGTGCCCACAAACTGTTAGGGCCTACAT

AAAGCT

>rnd-4_family-301#Unknown ( Recon Family Size = 47 Final Multiple Alignment Size = 42 )

AACCAGGTTTCCATCCAACCTTTTTATGCGAGTAAAGTACGTCGGATAAA

TGTCACGACAGGCCTGATGGAAACAGNAAATTTGTCGGTAAACTTTCCAA

ATGTCGACAAANCAAAATACGCTAGACANGGTGGGATCTTTTTGTGTCGG

TGAAATGGCGGTGGAAACGCCTTTATGCGCAAATATTGATATAATAACCA

TCATATCGAAGTAAACTTGGAGTCACGCGATGATATGTTGTGTGGTCCTC

CCACTACGACTCGGGAAAG

>rnd-4_family-419#Unknown ( Recon Family Size = 45 Final Multiple Alignment Size = 41 )

TATCTACTTCCCCAGAGTCAGATGAACTCGTGGATACCATTTTTATGTCT

CTGCGTCCAGTATGAAGGAAGTTAGAGGTAGTTTCGCGAGCCAATTGCTA

ACTAGCGTTAGCGCAATGACTGGAAGTCTACGGGTAACTGCTAGCATGCT

AGCGATACCCATAGACTTCCAGTCATTGCGCTAACGCTAGTTAGCATTGG

CTCGCGAAACTACCTCTAACTTCCTTCATACTGGACGCAGAGACATAAAA

ATGGTATCCACGAGTTCATCTGACTCTGGGGAAGTAGATAAAGGGCCTCA

TTGCCAAAATCCCGAAGTATCCCTTT

>rnd-4_family-730#Unknown ( Recon Family Size = 42 Final Multiple Alignment Size = 41 )

TTTTACTGCAGTGGGCTAAATCAGGGTCACACAGAGTGTTTCTTGGTAGT

CTTAAACAAATCTACTTTGAAACAAAAGTATACACCTCACACACATGGTT

ATGGGCTTAAAAAAAGAAGACACCTGTACCATGTCAGATATAGAGTTGAA

ATGTATTCAATTTTGAGTTTGCATCCCAATATTACACTTTATATACATCA

CAGAAGACTGAAATATAACAAAACCGTTTGACATAGAAACACCGGATTTT

CGGCGTTNTTTAAAAATAAATTAAAATGTTTATTAATTATGAAATTATGA

AAAATATGAATAACATTCCACCCATGAGGCCACTAGAGGGCGNTTTGGTC

ATTTGACTGCAGGAAAG

>rnd-4_family-321#Unknown ( Recon Family Size = 46 Final Multiple Alignment Size = 41 )

TTACACAGGCAGCCCAATTCTGATNTTTTTTCCACTAATTGGTCTTTTGA

CCAATCAGATCAGCTCTGAAAAAGATCTGATGTGAAAAGATCTGATGTGA

TTGGTCAAAAGACCAATTAGTGGAAAAAATATCAGAATTGGGCTGCCTGT

GTAAACGCA

>rnd-4_family-818#Unknown ( Recon Family Size = 49 Final Multiple Alignment Size = 41 )

GAAAATATATATAAGAATTTATCAACTCTACAAGCAACACTAGACTCTAT

TATTATGGTGGGAGATTNTAATACGGTTTTAAATACCTCTATGGACCGTA

AAGGAAATCACACTACAAACTATCACCCTCAGGCACTTAAGGAAATCACG

AATGTCATGGATATATTGGAATTAGTGGATATATGGAGGCTTAAATACCC

CGACCTAGTGAGATATACATGGCGGAGGCTTAATCAAGCTAGTCGTCTTG

ACTACTTTCTTATGCCATTCTCTCTGGCACCAAAAGTTTNAAAAGTGTTG

ATAGGGGACAGAATGCGGTCGGATCATCACATAATTGGCATATATATTAC

TCTTACAGAATTTCCACGTGGGCGAGGATATTGGAAATTTAATCAAAGCC

TACTGGATGATAANTTGTTTAGAACTAGGACAGAANAATTTATAACTGAC

TTTTTCAGACATAACATAGGTACAGCAGATCCCCTTACTGTATGGGACAC

TTTTAAATGTGCCTTTAGAGGCCATGCAATTCAGTACTCATCTATAAAAC

AAAAGCAATTTAGATCAAAAGAGTCCATATTAACAAAGGAAATTGAAGGA

CTAACAGTACAGTTAGATAGCAATAAAAGGG

>rnd-4_family-113#Unknown ( Recon Family Size = 44 Final Multiple Alignment Size = 40 )

ATTTAGAGTGATAGAAAGTGGCTTTAGCAGCGGATACAGAGGAAGAGAAG

GTAGAGAGGAGGGTGAAAGGATGATAGGTCCTCCGGAAGTTTAGTTTTCC

TCCATTTTCGCTCAGCTGCCCGCAGCCCTGTTCTGTAAGCTCGCAATGAG

TCACTCAGCCACGGAGCGGGAGGGGAGGGCCGAGCCGGCCGGGAGGAAAG

GGGACAGTGCGAGTCATAGGATGCGGAAAGGGAGGAGAGTAGGGTCGAAG

AGGCAGAATCAGGAGACAGGAGGGAGAAGGATTTAGCAGAAGGGAGAGAT

GATAGGATAGAAGAGGAGAGAGTAGTGGGAGAGAGAGAGAGAAGATTGCG

ACGGCGCATGACCATCTGGGTAGGGGCTGAGTGGNTAGGGTTGGAGGAAA

GGGAGACAGAAAAGGAAACAAAGTAGTGATCAGAGACCTGGAGGGGGGTT

GCAGTGAGATTAGTAGGCGAACAGCCTCTAGTAAAGATGAGGTCAAGCGT

ATTGCCTGCCTTGTGAGTGGGAGGGGACTGGGAAAGGGCGAGGTCAAAAG

AGAGAGNTGGAAAGAAATGAATCGAAGGCAGACGTCGGGAGGTTGAAGTC

GCCAAGTACGAAGAGCGGTGAGCCATCGTCAGGAAATNAGCTTATCAAGG

TGTCAAGCTCA

>rnd-4_family-94#Unknown ( Recon Family Size = 52 Final Multiple Alignment Size = 40 )

GCACATATCGGAATCGGCCGATATTAGCTAAAAATGCCAACATCGGTATC

GGCCCGATGTCTAGTTTAACGCCGATGTGCAAAACCGATGTCAAAGCTGA

CGTGCATACCTATATAAACGTAGGTACATGACGTAATGACGCCACGTAAA

ATTTTGCGCTACACGTGCAACACAGCATTCCTAACCTAGCCCACAATGTC

TGCTGTGTGGATCGAGCAGTCAACAAGTCGAGCAGTCATTTGAAAGAGTA

AGAACATTTCAGCGAGACAACTCAAAGGCGAAATCCATTAANGCCAAGAT

AATGGAATTCATTGCCCTTGACAATCAACCGTTCTCTGTCGTGGGTGATG

TTGGCTTTCGCCGACTGGTCGAGCACCGGTACACACTACCAAGTGCGCTA

TTTTTCAGATGTTGCCCTACCGGAGTTACACAGTAATAGCGTCACTGCTA

TTAGCTTCACGACATACATACTATGGAACGCCGTTTGGGTCTTTGCGTGT

CAAAAATACAGTAGCACTGTCAAAGCTGTACAAAAAAGTCTGCAAACACC

GGCCACGAACGATGTGTTTACAATACCGCGTTGGTAAT

>rnd-4_family-2051#DNA/hAT-Tag1 ( Recon Family Size = 45 Final Multiple Alignment Size = 40 )

GAAATACATCAAAAAGCGTGAAGACTTCTGCCTGAAGCCCATACACGCCG

CAGCGTACATGTTGGACCCCAAGTATGCTGGCAAGAGCATCCTGTCTGGT

GCAGAGATCAACAAGGCCTATGGTGTCATCACTACCGTGTCTCGCCACCT

TGGCCTGGATGAGGGCAAGGTTCTTGGCAGTCTGGCGAAGTACACTTCCA

AGCAAGGGCTTTGGGATGGAGATGCAATATGGCAGTCGTGCCAACATATC

TCATCAGCCACCTGGTGGAAGGGACTTTGTGGATCTGAGGCTCTTTCCCC

TGTTGCCTCCATCATCCTCCAAATCCCACCAACATCAGCCGCCTCAGAGC

GCAACTGGTCCTTGTTTGGGAACACACACACCAAAGCACGCAACAGGCTG

ACCAATACAAGGGTTGAAAAATTGGTGGCCATCCGGGCAAATTTGAGGCT

TTTTGAGCCTGACAACGAGCCATCCTCAACAAGGTTGGAAAGTGACAGTG

AAGATGAGGCCTCAGAGTCTGATGTTCAAGAGGTGGACATTGAGGAGGTC

CAGGGAGAAGACATGGAAGCCTGAGAGGAAGACAACC

>rnd-4_family-246#DNA/Harbinger ( Recon Family Size = 40 Final Multiple Alignment Size = 39 )

GTTTCCCGGACACAGATTAAGCCTAGTCCTGGACTAAAAAGCATGCTCAA

TGGAGAATCTCCATTGAAANTGCTTTTTAGTCCAGGACTAGGCTTAATCT

GTGTCCGGGAAACC

>rnd-4_family-13#rRNA ( Recon Family Size = 40 Final Multiple Alignment Size = 39 )

CCCTAGGCCTATACTATGTATTGTCTATTTTTAGTTGAACCCTGGGTTGA

ATTGAAACAATAGCTGTTGATGACTTTGCAAATGCTATATAGGCCTAAAT

AGCATCATTGATGATATATGANTGATAAAGTATGGTTACATTTCATTTGC

TCTGTTAAACCTACCCTTTGGAATGACTTCGATAGCAACAGTGAATCTAT

TTAGTTNTTAAGTGGAGATCTCTCAACAATCGTTCTCACGATAGCACATT

GGTAATAGTCAGTGACAAATATCAAAGCTAAGCAGGGCCGGGCTTGGTTA

AAACCCTGGATGGGAGACCGAAGGGATAGCTGTAGATAGATCAACTCTCC

AGTAGGAGGTGCTGCCCAGCCTATTGTTTTCTTCTGATAGTGGATATAAC

GTTGAAGATCTGACGTTGTTTCAAAGGTACAAATTCAACATATTTTATAC

AAGGTTTGTCTATGTTGAAATNTGGTTACCATGATGACATAATCCTGTGG

TTGAAATTTCACCCTCAAAACAACAGTTNATGACC

>rnd-4_family-1032#Unknown ( Recon Family Size = 39 Final Multiple Alignment Size = 38 )

TGGTGCACTATGTAAAGTCAAAGTGAGCCTGATTAACAGCTGTTGCATTT

CCACCATTTAGATAAGCATCTTTGTGTC

>rnd-4_family-933#Unknown ( Recon Family Size = 43 Final Multiple Alignment Size = 38 )

GGGTTGTGTTCCAAACGAAACAACTACAAGTGTGCTTGCTCTAGTTCCTC

AACGGCACAGCTAGGAGAGCTCAAAAAGCACCTTATAGTTGAAGACTCTT

CTTTGAGTCATAAAAGTGCATTGAAATTACTTAGGAACTGTGCACACTTT

GGAGAGGTGTGTGGCCACTTGGAGACACCAGTTAGTCCTCTCACTCAAAC

CCTTGTCATTTTTTTGTCTTATGTTGCACCTACCCCACATTTTCCAGGAA

TATTCTTATCATGTTACTGAATGTATCCAGAGTATTTTCAGATTTCGTTA

TCAACAAATGCGGCGAAAAGTACAGTAAATGTAAAATGCACATAAAATCA

ACAGTGTAATGTTTGGATTCAGTCTTGTGTCAGGTGAACTGTTGTGTCCT

CACCTTTGGTCTAATAATTTTCCCATAATCTCCAAACTGTTCCCTTTCAA

TTGCTACCATGGTTATGCGTATGCTTTCCATATTTCTTCTGTAAGAAA

>rnd-4_family-3356#DNA/hAT ( Recon Family Size = 39 Final Multiple Alignment Size = 37 )

CAGGGGTGTCAAACTCATTCCACGGAGGGCCTAGTGTCTGCNGGTTTTTG

TTTTTTCCTTTCAATTAAGACCTAGACAACCAGGTGAGGGGAGTTCCTTA

CTAATTAGTGACCTTAATTCATCAATCAAGTACAAGGGAGGAGCGAAAAC

CCGCAGACACTCGGCCCTCCGTGGAATGAGTTTGACAC

>rnd-4_family-51#SINE ( Recon Family Size = 39 Final Multiple Alignment Size = 37 )

TTGTTCTCAACTGGCCTACCTGGTTAAATAAAGGTGAAATAAAAATA

>rnd-4_family-323#DNA/TcMar-Tc1 ( Recon Family Size = 63 Final Multiple Alignment Size = 37 )

ACACCAGACATCCCAAGAATATTGCTGAACTGAAACAGTTTTGTAAAGAG

GAATGGTCCAAAATTCCTCCTGACCGTTGTGCGGGTCTGATCCGCAACTA

CAGGAAACGTTTGGTTGAGGTTATTGCTGCCAAAGGAGGGTCAACCAGTT

ATTAAATCCAAGGGTTCACATACTTTTTCCACCCTGCACTGTGAATGTTT

ACACGGTGTGTTCAATAAAGACATGAAAACGTATAATTGTTTGTGTTATT

AGTTTAAGCAGACTGTGTTTGTCTATTGTTGTGACCTAGATGAAGATCAG

ATCAAATTTTATGACCAATTTATGCAGAAATCCAGGTANTTCCAAAGGGT

TCACATACTTTTTCTTGCCACT

>rnd-4_family-386#Satellite ( Recon Family Size = 46 Final Multiple Alignment Size = 37 )

TCCCACATTGATCACAGCTANANGGTTTCTCTCCTGTGTGTGTTCGCTNG

TGCGTANTCAGGTGTCCTGANTGNGTGAAGCTCTTCCCACACTGATCGCA

GCTATAAGGCTTCTCTCCTGTGTGTGTTCGCTCGTGTNTATTCAGGTTNC

CCGACTGAGCGAAACTCTTCCCACACTGANCGCAGCCGTAAGGCTTCTCT

CCTGTGTGTATTCGCTGGTGTGTTNTCAGGTCTCCTGACCGAGTGAAACT

CTTCCCACANTCGGAGCAGCGGTAAGGCTTCTCTCCTGTGTGTATTCTCT

GGTGNNCTTTCAGGCTTCTAGATGCGGCGAAGCTCTTCCCACANTGAGAG

CAGCGGTAAGGCTTCTCTCCTGTGTGTGTTCTCTGGTGTNCTTTCAGGTC

CTTCGTTGAGCGAAACTCTTCCCACAGTCAGAGCAGCGGTGAGGTTTCTC

CCCCGTGCGTCTCCGCTGGTGTTTCTTGAGGTGTTCTGATCTGGAGAGAC

TCTTCTCTGCCTCGTCAGCATCATGATGTTGTTGAGGCTCCCCAGAGGAT

AAGNCCCTCCCACTGAGAGAGCGA

>rnd-4_family-233#Unknown ( Recon Family Size = 48 Final Multiple Alignment Size = 36 )

CCTGCATTCCCGCCTTTGGGACAACGACTCCCATTGTTAGGGCGGAGACA

TGAGCATCTCGTCATTATATACAGATCTCTGGTTTCAGCCNCTTGCGAAT

TGGAAAGGAAAGTTGGCGGGTGCACAGTGCACTGTTCGGATGCTGGCTTC

CCCTAAAGTAAATTGATGTTTTGCCAAAATTATATAATTACTCCTGTCTA

AATAAAATNATTCAAAATACTTCACCAGAGAGCATGACTTAGCCACAGAG

GATCATTAGCTTCTTTAAAAAATAGCTGTGGATTGTTTCAAATCACAAGC

GCGTAGGCCTATGCCTATTTGGGAAGCCCGCAATTTGGGCAGCGCGCGTG

GCAATAGGCCTAGCTGATTATTGAGTTGCGGCTGTCAGTGAAAAGCATCT

AAAATATGTGTGAAGATAATGTGTCTTGAGTATAATTTAAAATGTTGAGA

CAAGAAGGGGAGGGGTGTGTGGCGAAGATATGGGCGTCTCTCTCCAGAAT

GCATGCACTCAGCTCTCCAGAATGCGCTCAGCTGTCTCCCGCCAATTCTT

GCGCATTCATTGTTTCATTGTGTGAATTGTTTGCCCTTTGATTGTTTATT

TTTATCAATTCCCCATTACGTATGTCACCAGTAGGCCTAGTA

>rnd-4_family-18#LTR/Gypsy ( Recon Family Size = 37 Final Multiple Alignment Size = 36 )

GACTGACCCAGCAGACCCGGGCCAGCTGCGCAACGCCGTCTCCTCCCAAG

GAGCCACCATCGGGAGGCACGAGGAGTTGCTTCGCGGCCTTATGGAGGGG

TTCCAGACCTTGGCCGAACGCCATGACCGNGCGTTGGACACNTTGCTGGA

GCAATTCCGCGGGTTGCCTGNTAGGCAGCCTACCACGACGGTAACCTCCC

AGCCCCTCAGTAACCCGGCTGTTAGCAGCGCCGCCTCCCCGGCCACCCCG

GCTTCCCGGGAACCCCGCTTACCTCCCCCGGAACGCTTCGATGGAGAGTC

GGGCACCTGTCGGGCGTTTCTAGCTCAGTGTNCCCTCATCTTCGAGCTGC

AGCCCTCCTCCTTCCCCTCGGACCGCTCNAAGATAGCNTACCTCATCACG

CTGATGTCCGGGAGGGCTCTCGCCTGGGCTACGGCNGTATGGGAACAACA

GTCGGCCGTATGCNTCAGTCTGGAGGGGTTCGTGGCGGAGGTGAGGAANG

TTTTGATGCTCCGTTGTCCGGGAGAGAGGCTGCCCGGAAGCTACTCCAGC

TTCGGCAGGACTCCCGCAGTGTGGCAGACTATGCGGTGGATTTCCGCACG

TTGGCAGCTGAGAGTGCCTGGAACCCGGAAGCACTGTTCGACATGTTCCT

GCACGGCGTCTCGGAGGAAGTAAA

>rnd-4_family-460#Unknown ( Recon Family Size = 37 Final Multiple Alignment Size = 36 )

CAGGTATTCCCAAACTGGGGTACGCGCAATGCCGTCGGGGTACGCCAAAT

AAAAATGTGATTATATTTTCCAACGGGGCTATACATTTGGGTGAGGTTTT

TTTCTCGCCTGAGTAGCCTCGTTTCACTGCCAAAAATAAAATTAAACCAT

CTAGTGTTCAGCGAAATAACAACACAATGTCAAATACAGGTAGCCTAGTC

AAATAATTAACATCCAATCACATTAACCGTTACTCTCTCGCGGGAATTCC

ACTAACGGTCCGTATGTAGCCAAACGTAGCTGCTGCTCA

>rnd-4_family-599#Unknown ( Recon Family Size = 42 Final Multiple Alignment Size = 36 )

AATGATTGAATAACATGTATGTGTACATTTATTTTGCAACGCTCGCGCAC

GCGACGCGAGCGGTGTGGTCAGCA

>rnd-4_family-1085#Unknown ( Recon Family Size = 42 Final Multiple Alignment Size = 36 )

GCAGGGATCGTCAACTAGATTCAGCCGCGGGACGATTTTTTTCTTGAGCG

GATGGTCGGGGGCCCGGAACATAATTACAAATAATTTGTANACTGCAAAT

TGACCGCAAGAAGCCCAAACAGATATNATATTTGACTAAAACATAATNAT

TTCGAACCTTGCTTACATTTGTATACGATCACGTACTTCTCTCTATTATG

CGTGGGAATACTTGGGAACAGATTTCCAAAATTAAAATCACTTGGAGCTG

ATTTCCTGGTGTTTTTACAGTCTTTTATGTCCAACAATGAAAAANTCAAA

ANAGGTATTTNTTTTTTTGCTCAGAAAACTTGGGGGGCCAAATAAAACCA

CCCGCGCGCCGCCAGTTGGGGAAAGCTGCCCTA

>rnd-4_family-145#Unknown ( Recon Family Size = 55 Final Multiple Alignment Size = 35 )

TACTTTTTTCAATTTCCGCCTGAAGACATACCCAAATCTAACTGCCTGTA

GCTCAGGCCCAGAAGCAAGGATATGCATATTCTTGGTACCATTTGAAAGG

AAACACTCTGAAGTTTGTGNAAATGTGAATTGAATGTAGGAGAATATAAC

ACAATAGATCTGGTTTAGATAATACAATGAAAAAAACCATACGTTTTTTT

TTTGTTGCATCATCTTTAAAATGAACAAGANAAAACAAACATTCAGATAG

GATGATGGGGACAATTTCGGTGAAAAACATAAGAGGGCAACAGTACTTGT

GCGAAGTTTCAGAATGATAACTTCCAAAATGAGTGTGCTACATGACATTT

ATCATGAAGTCACCCAGGTGTCCCACACAAGTAGCCCAAATGTACCCAAG

TGGCCGAATTGGTGAAGTTATACATTTTGAAGCANATAACTATATACAAC

ATACAAAAATGGTATTCTAACACACC

>rnd-4_family-591#Unknown ( Recon Family Size = 49 Final Multiple Alignment Size = 34 )

TCATTTTAGTCTATTCAAACGGACCCCATTTCAAAGGAAACAAGCACTCA

TTAAGATCAGGTGTGGCCAATTAGTGGGCGCGGCCAACACACCTGAACAC

ACTTAACAAGATAGAGGATAGAGAGAGTTTTGTTGATGCTGAGAACGGAA

CGTATATGTTTTTAAATAACATTCTTANAACGTTCTTTGAACGTTACTAA

TGTTTTCTTGTGGTTTTTATGGAACGTTTTCTTAACGTTCTGAGAACATG

ACTTTAAATAGAACCATGAGGAAACCTGTAGGAAACGTTATGCTGAAGTA

CTGAAATTCCCACAGAAGAACGTTGTTTCTTAACGTTCTCTGAACTATTT

GAGAACGTTCCCAATGTCAAACCAGTTGGAGAACGTTCCTAGAACATTAC

CAAAATTGNAATTAAATGTAACCATGTTTGAACTTTTAGGAAACGTTCTG

TTAAAGTAATGAAATACCAAGANNATTTTTTTGTCAAGTTCCTTAAATGT

GCTGAGAATGTTCCAAAGCCAAGCAACTATCCTGCACCATTCCCAGAAAG

TTGTGGGAAGGTTGTACGCAAAATAACCATAGGACAACCACGCTC

>rnd-4_family-760#LINE/L1 ( Recon Family Size = 36 Final Multiple Alignment Size = 34 )

TGATATACTAAAAGCTCCATTGTTAGATCGTTTTAACTACTCCTATAGAA

ATGGTAGTCTGTCAGGTACTCAGCAGGAAGGTCTGATTTCTCTATTACTA

AACAAGACCCAGTCGGCAAATNTAAAAAACTGGAGGCCCCTTACACTTCA

GTGTTGTGATGCAAAAATNCTAGCAAAATGCATAGCGCATAGAATTAAAA

AGGTNTTGCCGGATATTATTCATCCTGATCAGACAGGTTTTTTACATGGA

CGATACATTGGAGATAATATANGACAAGTACTGGAAACAATAGAACACTA

TGAAANATCCGGGAAACCAGGCCTGGTNTTCATAGCGGATTTTGAAAAGG

CNTTTGATAAAGTACGACTGGAATTTATATATAAATGCCTGGANTATTTC

AATTTTGGAGAATCTCTTATAAAATGGGTTAAAGTTATGTACAGCAACCC

TAGGTGTAAAATAGTAAATAACGGCTACTTCTCAGAAAGTNTTGAACTGT

CAAGAGGAGTAAAACAAGGTTGTCCGCTGTCNCCATATCTATTTATTATG

GCCATTGAAATGCTAGCTATTAAAATCAGATAC

>rnd-4_family-299#DNA/TcMar-Tc1 ( Recon Family Size = 35 Final Multiple Alignment Size = 34 )

TGGTATGACTTAAAGATTGCTGTACACCAGCGGAACCCATCCAACTTGAA

GGAGCTGGAGCAGTTTTGCCTTGAAGAATGGGCAAAAATCCCAGTGGCTA

GATGTG

>rnd-4_family-1160#Unknown ( Recon Family Size = 48 Final Multiple Alignment Size = 34 )

TGTCATGACGTTGGCCTGGGGGTAGGTTTATGACAGTCATAAATACCTCT

TCCCCCCTTTTTCCTCTCTCTACCCTACTGANGTTACATTTGCAAAACCC

TTGGTTAACATAGAGATTCTGGGAACATCAGAAGGTGGGGGGAAATGAAC

TATATTCTGGTAATCCGACCAATTGAACATATGCGGTGGTACTTAATGAA

TATGATGTCAGTTCGGTTGTCATCTGAGACATTCTCATCAATGATAAGAT

GACATAAACTCTACAGTGGAAAGTCTACACATCAGAGTTATCGGATTCAC

ATGGAATTGTTGTTCAATTTAAATGTTTGAATATGAAATTATTCGTGATG

GGATGAAATGTGATTTTAGCTTCTAAAATGTGAGATTTGGGTTTTCATAA

GGTAGGGCTCTGCTCAATCAGTGGCCCGCCCCTGTGAAGGGACATGGGCT

ATAAAACTTTTCAAACACGCCCTCCTCTCCCTTCCTATATAAGCCCTTGA

CGACAATATAACCTCCTGTTCCGAGGACGTGAGGACGACGGTCCGATGTC

A

>rnd-4_family-327#rRNA ( Recon Family Size = 35 Final Multiple Alignment Size = 34 )

TTTTAAGCAGGAGGTGTCAGAAAAGTTACCACAGGGATAACTGGCTTGTG

GCGGCCAAGCGTTCATAGCGACGTCGCTTTTTGATCCTTCGATGTCGGCT

CTTCCTATCATTGTGAAGCAGAATTCACCAAGCGTTGGATTGTTCACCCA

CTAATAGGGAACGTGAGCTGGGTTTAGACCGTCGTGAGACAGGTTAGTTT

TACCCTACTGATGATGTGTTGTTGCAATAGTAATCCTGCTCAGTACGAGA

GGAACCGCAGGTTCAGACATTTGGTGTATGTGCTTGGCTGAGGAGCCAAT

GGTGCGAAGCTACCATCTGTGGGATTATGACTGAACGCCTCTAAGTCAGA

ATCCCCCCTAAACGTAATGATACCGTAGCGCCGTGGAGCTTCGGTTGGCC

CGGGATAGCCTGCCTCTTTTGGCAGGTGAGTAGAGCCGTTCGTGACAGGG

TCGGGGTGCGGCCGAATGAGTTGCCGCCCCTCTCCTGATGCGCACCGCAT

GTTTGTGGAGAACCTGGTGCTAAATCACTCGTAGACGACCTGATTCTGGG

TCAGGGTTTCGTGCGTAGCAGAGCAGCTCACTCGCTGCGATCTATTGAAA

GTCAGCCCTCGAT

>rnd-4_family-48#Unknown ( Recon Family Size = 36 Final Multiple Alignment Size = 33 )

TCATTCTTTATATAATTTATTTCTTTGTTTCATAGTATAGTTTCTTCTTT

TTATTCAGTTTAGTCACATGATTTCTCAATTTGCAGTACGTTTGCCAATC

AGTTGTGCTGCCAGACTTA

>rnd-4_family-990#Unknown ( Recon Family Size = 37 Final Multiple Alignment Size = 33 )

GTCATGTCTGTCAATTATTTGGACACGTTGGTAACCAGGTTGCGTATNTA

AGTGATAATGCCCGAGAAGCCGGTGTTTGGAGGATATATTGGCACGGGTG

TTGTTGGCAAACCGTGCCAATATATCCTCCAAACACCGGCTTCGAGGGCA

TTATCACTTTTATACAACGGGTTACCAACATATTCAAATAATGATTGACA

TATTTTCATTAAAAACGTTATTTTGATGAATTTATTCATACTATTTCATC

CTTCCACGAGATATAGTCCCGACACAAATCTAGGGTTGCTACCCAAGCCG

GCTGGTCGTTCGTTCTATCGGTTCGGTTGCCAGAGACGCGACCCAGTCGT

TCAGTCTTTTTGTTCTGTATCTATGGACGCGACCCAGTCGTTCGTTCTAA

ATGTTCCATTGCCATGCTGGCTGGCTGGCAACGTTCTTGTCCCTTGCTTG

CTAGCTAGCCAACTA

>rnd-4_family-1028#SINE? ( Recon Family Size = 37 Final Multiple Alignment Size = 33 )

TTCTTCTGTCAACGTTGTCGTGGCCGAGTGGTTAAGGCGATGGACTAGAA

ATCCATTAGGATCTTCCTGCGCAGGTTCAAATCCTGCCGACAACGAAAAT

GCATTTTTTTGACCCCTCCGGAGGTTGAAGTTTAGTCGTGTTTCTTATAC

CAATCGCATCCTCTCAGATCTCCGCAAGTACATAGGGATTAGTTGTCCAT

TTGGGATAGGACTGCAGGCCATCTGTCCCACTTCACACCTTTACACACCT

GGTTATCCAGCCTTCTAGCTCTATAGGCCACAGTAGCATAGGTTACAGGT

TTGTAGTCAAGCCACAGATTTCAGGTGAGATCCCATGATACACTCAACAA

TACAACAACACGATTCTCATGAATAACCATGAAGCCATTTGCTAAGATTT

CCCCCATTTTGACATGTGGAAACCTGTTGTCACTCTTACCCTGACGGGAA

GCTCCAGACACTTTTTTGGTCCTATCTGAGGCATTTTTAAATTCATGTCA

AATTGGCCATATTCTGAAGACACTCTTAGGCTGCAGGGTAATCGAACTGG

CCACATCTAAAGTCAAGCCACAGGTAACAGGTGAGAAACCTTGTTAAACC

CTAAAAGAAAAAGAAAAA

>rnd-4_family-45#Unknown ( Recon Family Size = 34 Final Multiple Alignment Size = 33 )

GAGACCATGTTTGTATGCGGCTTTATTAACTCAATGATATATATATGTAT

ATATATATATTATTTTACATTGTTTGCAAACTGATATGTGACACGTATTA

ATGCCAAAATAACATGCAAAACAGGCAAGCCCCACAAAAAATGTGGGGCC

CCACCTGCCCTGAATGACGGGTCGCCACTGGT

>rnd-4_family-423#Unknown ( Recon Family Size = 47 Final Multiple Alignment Size = 33 )

TTTTGTTGTCCTAGGCTACCTGGCTAAAATGCTTGCTCGCTAGCCTAACT

TCCTTTCATGGGCAACGATCCAGGCCAGCTAGTTAACATTAGCCTACTAC

TACATCTAGCTACATGTTGAACTTCCATCCTCTCAGGCCAGGGGCACGAT

GTATGAATTTACGGTTGGATCAGAATCGCCGTTATAATCATTGGCCAGTA

CGGAGAATTAAGTAAAACCACAAGTCCAAATCCCTATCTCCATCCATGGC

TAATTTAGGAAAGGGACGATTTTAGCTAGCTAGCTAGCCACCGGAGGACA

ACGACACAACGAGATGCAACAATTCAAGTTTTCTGTCAATGACGTTTGGC

TTCTGATGTGATGTGATTGGTGTGAAGCCAAATCCAAACTGGCTTCCCTT

GACACTTTTTTTTGGTGCGCCAGGACCATTCACAGTTGAGCTCACTCAGT

TTAGCTCAACGCTGATTGGCTATTATTNTTTTATCAAGGGAGGCCAAATG

CTCGCTGGCTTCCCTTGCATTCAATGCTACGGGCGGCAACAATGTCATAC

TCTTTTTGACCAGACAG

>rnd-4_family-67#Unknown ( Recon Family Size = 36 Final Multiple Alignment Size = 33 )

AGAGAGCAGAGGTCANGATCAGATGAGCTCCCGCATTTTTCAGCATGTTC

TTAAAAAAAGATAGATTTAGAGTTAGATAAACTTGGATTTTTCGTCAGGG

ACATATACAGGATGCTACCCTCCACAACATAACCTTCACTCCAAATCAAA

ACTCCAAACCTACAACCTGATTTTGGACGGAATTACCTGGAAGGATTACT

ACTACCAAGGATTNTTATTTCCAAGCTATACGGCGAATGCTCTGGAAAAC

AACAGAAACCTGAAAACACCATCCAACCTGGAACTGAGTGAGTAATGTTT

AGTCTGAAGCTACTTTATTAAAGCCAAGAAGAAAAATACATTACATAACT

TTATAAGGACTGAATGCTAANATAAG

>rnd-4_family-457#LINE/L2 ( Recon Family Size = 39 Final Multiple Alignment Size = 32 )

GGTTTCGTCGGCGAAACTAAGACCGTCGCCGAAACAAAGACGGTTCTGCC

GAGTTGTTCGAGGAAGGCTCCACACCACTCTCTCACTTGAGTATCTTACC

TAGTTATTTTCAGATCTCATTTTGCTCTTTTGTAGCTTTGGCAACTATGT

GTGTGTTTTCTATACCTGTTCGCTCCTCTCCCTGTAGGCTTTACAGACGC

TCCCCACCCCTTGGCTGCAACCCTTCTAATTTAGTGTACCCTGCGCGCAC

AACCCATGGGTTTGAACTGCCGATCTGCGGTCAAGAACGCCGAGTTCATC

TCAGCCTATGCTGCCCTTCAGTCCCTTGACTTTTTGGCCCTGACGGAGAC

ATGGATCACCCCAGAGAACACTGCTACTCCAGCTGCTCTCTCTTCATCTG

ACTACGTTTTCTCTCATAGTCCGAGAGCATCTGGTCGTCGCGGTGGTGGC

ACAGGGCTACTCATTTCTCCTAAGTGGAGATTTTCTTTTCTCCCTCTCTC

ACCTGTCCATCTCCTCATTTGAATTCCATGCTGTCACTGTCACTTGTCCA

C

>rnd-4_family-204#DNA/hAT-Charlie ( Recon Family Size = 36 Final Multiple Alignment Size = 32 )

TATATGGTGAGCTACCGAGTGGCTAGGACAGGCAAGCCCCATACTATTGT

GAAGGACTTAATTCTTCCTGCTGNCGTGGATATGGCTGGGACAATGCTGA

GGGAAAAGGCCCAAAAAACTATACAGACAATGNCTTCATCAAACAACACT

GTTTCACGACGCATCAGTGACATGGCAGGAGATGTTTTGAAACAATTACT

GCTTCGCATACAAGCCAGTGAATTCTATGCGTTACAGCTGGATGAGTCAA

CAGACGTGGCGGGCCTGGCACAGCTCCTGGTATATGTCCGTTACGTTTAT

GGGGGGTCAATTAAGGAAGACATCCTCTTCTGCAAACCACTGGAAACCAG

GACAACAGGAGAGGATATTTTTAAAGTACTGGACAGCTTTGTGACATCAA

ATGGACTTTGGTGGTCAAGA

>rnd-4_family-1288#DNA/Tc1 ( Recon Family Size = 50 Final Multiple Alignment Size = 32 )

ATACCCAAGAAGACTCGAGGCTGTAATCGCTGCCAAAGGTGCTTCAACAA

AGTACTGAGTAAAGGGTCTGAATACTTATGTAAATGTGATATTTCAGTTT

TGAATTTTTAATATAAAACAATGTCTAAACCTGTTTTTGCTTTGTCATTA

TGGGGTATTGTGTGTAGATTGATGAGAAANAACAATTTAATAAGGCTGTA

ACGTAACAAAATGTGGAAAAAGTCAAGGGGTCTGAATACTTTCCGAATGC

A

>rnd-4_family-265#Unknown ( Recon Family Size = 60 Final Multiple Alignment Size = 32 )

ACAAGCGGTTTCAACTCGCTATTTGCGTTTGAGGTTTGGTCCAACAGAAT

GGTCATATGGACACCGAAACACATTGAGACATTATTTTACTGTAATAGAG

AAGTTAACCTTTCTAACGATACCCTTTTTATGTCTCAACTACTCAAATTG

CGCGCAGAGCAGACACTACTAAAACGAGAGTATCAATGAACATTGATTCT

GGAAAATGAATGCTCAGTTTGTCGTGCGCGGCATTGGGGTACCACGTACC

GCTAGCAGCATTTTAGCCAAAGACTGTTATACTAGCTGTCTAGTCTGTGT

TTTATAAATGTGCAATAAGCATAAAACACAATTATATAAGGAATTGGCAA

CTCGTTCTCATTCTGAGAAATAAGGTAGGCCACTTGATTTCAACATCTGA

ACAAAGTGGACAGGCTAGCATGCTGTTCAAACAGTTGGAGACAGACAGAA

GGGTGTGTTCATAACAATTCAACTGTTCTACCTTGTTAGCTAGCAAATAG

ATCCAAGTTGGCTAAACTTGAAATATAAAGATTAGCTGGCTACTCACTAG

CACGTTGGGCTTGAGAGATTGTTTATGAGACCTGTGTTAATTTT

>rnd-4_family-734#Unknown ( Recon Family Size = 42 Final Multiple Alignment Size = 32 )

ATAGTCCCCAGTCACGTGGTGTTTGTTTACAAGCACACAACGACGAGAGA

CCGGAGCCTTGTGAGTCACTCACTGTTGTGCAGCACGCGCCAGGTGATCT

AGTTAACAGTATGGAATTCACAACTAAATGTTTGCCAGCTAGATATCTTA

TAACTATTAAGTTAACTGTCTAAAATGTGCTAAATGCTCTGCAGTTGTGC

ATTTGGTTTGCTAATTTAGTAGCTAGTTAGCTATCTAGCTAAGCGGTTAG

CTTCTTCCAAAATCAAGCTTCGCTTGGTAACAGCAGAGAATCCCCTCCTG

GATCAAGAGCCTTGCTGTCTGTTTTGTGCGTGCGGCAAACTGTGAGTAGC

ATTTTTTGTTACTTGTATAGTTTAGAGACTGTATAAAACGTTTGCAATGC

GCTCGTTAGCATTTAGTTAGCATTCTCTATGGGATTTTACATGTACTTGT

TAGCATTGCTAACCTTCGGATTACAGAGGCTCAGTGGGGTTTGAAAATAG

CGCCCCTTGTGTTCAGTGCCGGTATTACCGAATATCCCGGTATGGCACAA

GGTCGGTATGAAGGTATGACAATC

>rnd-4_family-910#Unknown ( Recon Family Size = 53 Final Multiple Alignment Size = 32 )

GTTTGAGCATGTGTCCATTAGGCCTATGGATTTATTTTTTTATCAGCATG

AATTAGATTGAGCAATAAAAGCCCCACTTTTATTCCATAGGCTGGGATCC

GCACTATGCAGCTGTTGCAAGAGCGCATTTTTCACTGGCTGTCCACTGGT

TTCAAAAACAATGATTGATAGGCAGCTTAAACTTCTTGAATTCAACCATT

ATTGGGTTCAAATACACATTTAGATTTGTGAACAGCCATCCACAACAACC

ACAATCCGTAAGGCGCAAATAGCTAAATGAGAGAGCAGCAGTGTGATTCA

CATCAATGCGCTATGTAGATATCAATAATAAGTGATATCCGTATCGCCGT

AGACTACACCACTGCTGTCATCCTTACCTCCAAGCGTTTATTCAAGTTGG

ATAATCTTTGGATGCCGACAGCAGTCGCACCATTGGAAGACATAGCTTGG

ACTGTAGCCTACAAAAGCCTATTCCTGCTCTTTTCCCGCGATCCATCAAA

CACATTTGGCGTGTCATCAT

>rnd-4_family-281#LTR/Gypsy ( Recon Family Size = 37 Final Multiple Alignment Size = 31 )

GAAGGCGTGGAGGCATTGGCTTGAGGGGGCTAAACACNNNNNNNNNNNNN

NNNNNNNNNNNNNNNNNNNNNNNNNNNNNNNNNNNNNNNNNNNNNNNNNN

NNNNNNNNNNNNNNNNNNNNNNNNNNNNNNNNNNNNNNNNNNNNNNNNNN

NNNNNNNNNNNNNNNNNNNNNNNNNNNNNNNNNNNNNNNNNNNNNNNNNN

TGTATGACACAGAGGAGCGGTCCATGGATCCCACTCCCATACTCCCGGCT

TCGTGTCTGGTGGCGCCGGTGGTGTGGGAGCTGGACGCGGACATTGAGCG

GGCGTCACGTGCGGAGCCCGCTCCCCCCGAGTGTCCGGCTGGTCGTCTGT

ACGTTCCGTCTGCTGTCCGCGACCGATTGATCTATTGGGCCCACACGTCA

CCCTCCTCTGGTCATCCTGGGATAGGTCGGACGGTGCGCTGTCTTGACGG

GAGGTACTGGTGGCCCACTTTGGCTAAGGACGTGAGGGTTTACGTTTCCT

CCTGCTCGGTGTGCGCCCAGTGCAAGGCTCCTAGACACCTGCCCGGNGGN

AAGCTACNNCCCCTGCCCGTTCCACAACGGCCCTGGTCNCACCTGTCGGT

GGACTTTGTNACTGACCTTCCCCCCTCNCAGGGNAACAC

>rnd-4_family-336#Unknown ( Recon Family Size = 32 Final Multiple Alignment Size = 31 )

TTAGTATAGTTTTATCTAAAAAGGATAACTTTTTAAATGTTTCACTATTT

TATTTTTATGAAATTCACTGAGGAGGATGGTCCTCCCCTTCCTCCTCTGA

GGAGCCTCC

>rnd-4_family-1706#Unknown ( Recon Family Size = 33 Final Multiple Alignment Size = 31 )

AAATGTATCGTATAGTGTGTGTGTGGCAGGCTTACAATGATGGCAAAAAA

CAACATTTGAGAGTGCGCTGACCCTGGTGCTAGAGGGGGTACGCAGCTGG

AGGTTGAATGTTTGAAGGGGTACGGGACTATAAAAAGTTTGGGAACCACT

G

>rnd-4_family-1119#Unknown ( Recon Family Size = 32 Final Multiple Alignment Size = 31 )

GTTGGCGTTTTTTCGTCATGAATCTTGTTCTGGAGGCAGCTCTGCAGAGT

GGTCACTAGCTGGCACAGCCACAAAGTCATAAAATCTGATTTTAAACCTT

AACCCTAACCTTAACCACACTGCTAACCTTATGCCTAACCTTAAATTAAG

ACCAAAAANCATATTTGTTTTCATGAATTTTTACGATATAGCCAATTTTG

ACTTTGAAATCGCTCAGTTCTGCCTCCAGGACAAGACTCATGACAATAAA

CGTCAACCTG

>rnd-4_family-643#Unknown ( Recon Family Size = 40 Final Multiple Alignment Size = 31 )

TTTACATGCACACTAATAATTCGATATTAAACTGATTATGGCAGTAGGCN

GATTATGGCAATAGTCATGTAAACACCTTACTCTGCTTATCTTAATCGGC

GTAAGGTCATAATCGAAGTAAGCATACGCCGATTAAAACACCTGGTTTTC

TGAGCAATCTTTCNAATNATTAGGACATGTAAACACCTTAATCGGCGTTC

CAGCGGTGTATTTGATCTGCGCGTGTGCCAGCACCGGCAGCGCGAGCCTA

TGTNATGTATGCGTCTTAGAAATAGTTTTCACATACAAACTTTATGTCCG

AACTCAGAATCAAATANGCTTCCCAAAAATAACATGGTCGCTGTGGTAGA

ACGTTTATTTTGATTGGCGATTCTGCGTTTATCAAAATCCCATCAGGTAG

CCTGATTTCAGATGTGTCCATGTAAACAGGATTATTAGGGAAATCGTTCT

TCTTGC

>rnd-4_family-653#LTR/Gypsy ( Recon Family Size = 47 Final Multiple Alignment Size = 31 )

TTGTCTGAAGTCGGCGCAGCCTGCCCCGGGATNTCTTCATGCGGGCTTGA

GAGAAGCCTCGGATCTCTCCCTTNCCGTCGGGGAGNACCAGGACCTCCGG

GAGGTTTTCAACAAGGCCCGCGCTACATCTCTCCCTCCGCATCGACCCTA

CGACTGCGCCATCGACCTTCTCCCAGGAACTACACCGCTTCAGGTCCGGA

GACCAAGGCCATGGAGGNGTACATCGAGGACTCTCTNGCTGCAGGGAGTA

TTCGTCCNTCTGCCTCCCCCGCCGGCGCAGGGTTCTTCTTTGTGGAGAAG

AAGGACGAAACCCTGCGCCCGTGCATCGACTACCGGGGCCTNAACGACAT

CACGGTTAAGAAGTTACCCNCTACCNCTCATCGCCNCGGCNTTCGAGCCT

CTCCAGGGNGCGACNATCTTCACNAAGCTGGACCTTCGGAACGCNTACCA

TCTGGTGCGNATCCGGGAGGGAGACGAGTGGAAGACCGCNTTTAACACGN

CTACGGNCACTACGAGTACCTGGTNATGCCNTTCGGNCTGACCAACGCTC

CNGCNGTNTTCCAGGCCCTGGTNAACGACGTGCTTAGGGACATGCTGAAT

CCG

>rnd-4_family-238#Unknown ( Recon Family Size = 79 Final Multiple Alignment Size = 31 )

TACACCTCCATCACTCCAGTATCCCTGCACATTGTAAATATGGTATTGGA

ACTGACCCTGTATATAGTATGCTTACTTACTTTNTCGTGTTCTTCTTATT

TCTATTTCTCGTGTGTTTTTGTTCTACCTTAGTTATTTTTAGTACTACAT

TGNTATTGATTACTGCATTGTTGGGTTTAGAGCTTGCAAGAAAGGCATTT

CACTGTACTTGTGCACGTGACATTAAAACTTGAAACT

>rnd-4_family-170#Unknown ( Recon Family Size = 43 Final Multiple Alignment Size = 30 )

ATAAATCAATGAGATTTTTATTTTCACTGAATCTCCGTTTGGGTATTGGT

TAGNCTACAATTAGGGCGTGGAAATGTTAGGATTAATTTGCTCTTCACTC

GCAGTCAGTAGCCTACTCCCGACCGTCACGTTGTACAGCGCCATATTTTC

CATTCCATCCTAATGGAAACCCTGAGGGTTTCGTTTTTAGTTTTTCTCGG

AATAGAAACACCATAATATTAATCAAATTAATTAAGCCAAATTTCTTAAA

ATCAATCCCATATACTATGTTCTTACAAAAAGGTTTTAAATTCTCTAGTA

CAGCCAATATTGAAGACTATCAAATGCTTCTCAAAGATGCCCTCTGGTGG

TCAAACTAGCACTAACTNGCATTAACGGNAACAATGGCTGACACTTAAAT

AACGTGCCATAGAATTCTGCGGCAGCCCGCAAGNTGTGCCGCAGTACGAC

GCAACT

>rnd-4_family-359#Unknown ( Recon Family Size = 38 Final Multiple Alignment Size = 30 )

TTATGCATAACCCATCATTNATTAGCTAAATATAAGGCTAATACTGCATT

GAATGTATTACCTGAAAGAGGTAGGCTAGGACGTCTATATATAGGGCTAT

ATATCAATCTAGAACAGGATTATCTATTTTGGATGCAATTTGAATGGAGT

TGATGGAGAGAGAGAAAGACTGCGAGAGAGTGCTCGCGCGTGCACTGATT

TAAAGCAACGATATTCGAGTGATAGGCTGTTTTAAAACTCTGCAGCTGCA

ATTAAAAACAAGGTGACCCCCGAAAGCCAGATGGAGATGGGTAAATTAGA

CGCGCATTCGGTCCTTATATTACTGTAGCATAGGCTATGCTGCAGCAAAT

GTAGGCCTACCTGTCACAAGAAAAAAAGTTACCATGATGAGATGATAGGT

CTACATGCATTGTGAACTGCGCTCCATACTGAGATGGGCCGTCTGTCCCC

ACCCTGAGCGTTGATGATTCATCATGCAGCGGCCGTAGAGAAGCCAGATT

TAGACATGCCGTTCATATAAATAATTTATTATAATTTACCGGTTTCTCGC

AGTTATTTATCCCGGGAAAAGGGAGTGGTTTTGGGTGG

>rnd-4_family-345#Unknown ( Recon Family Size = 47 Final Multiple Alignment Size = 30 )

CGTATCGGCCTCACGGCCTTCGTCAGAGCTTTTGTGAGTTTAAAAAAATT

AGCACCCTTATGTAGACCTAGCCCCACCCACATCCGTTCCACGCATCGAA

NGGGGTTGGAGGCGAAGGAAAAAACAAATAAGTGCTACCAAATATAACAA

TGTGCATTTCATAAATATTCGAATAAAGTGTGTACATAAAACGTATTAAA

CACGTCTGTAAAACCACAATGAGGACATCGACCTACCGAGCAAGTTTTCA

TAAATCATTGGGTACAGCGTAAGAAAAACGTCAGAGTAATCTGAAATAGG

GGCATAATTCATGTTCACATTTACAACATAACATACATAGGCATTTCATC

ATTAAGNCCTTTAGGAAATAATGTCTGGAGGGTGAAAATCCCAAAACATT

CTCTTTTGCTCAGAGTATTATTNATATCACCTCCCCTGTCTGATATCTTA

ACTTTCTCTATGCCACAAAATCTAAAGGTAGAAATGTCATGTTTTAGGTC

ATTAAAATGTACTGCGACTGGATAATCCCTGTCGTTTCTCCTGATTNAAC

TTTTATGTTCACTAATTCTCTGTTTGAGAGAACGAGAGGTTTTACCTACA

T

>rnd-4_family-561#Unknown ( Recon Family Size = 34 Final Multiple Alignment Size = 30 )

TCTCCGGGGTTCGCCGTCGAATGGCTGACCCTGGCCGTGACCCCACTCTC

CGAGGGTGTCTCAGGGGGAGTGGGATATGCAAAAACACATTTCCAATTCA

CACGTGTATTAATACACACTTGTACATGCGTGAAATAGGACAAATATAAG

CACCCACCNAATTATTATTATTATTAT

>rnd-4_family-21#Unknown ( Recon Family Size = 37 Final Multiple Alignment Size = 29 )

TATACTTTTTTAGCCAGTAGTTCTGAAAGTAGCGCTCACGAGCCAAAAGT

GGTCCCCGNACTGCGTCACGTAATGTGCAGATATGTGCACCACGTCATTG

CTCTCTCGCTCTCTCTCTCGCTCTGCCGTGCGACCTCGTTGGATAACGCT

GGGCAGGCCTCTTGCTAGCTGTCACTCAAATGGCGAGGGGCTGAAGCTCA

TTGGCTAGAACTCGAATTGCTAGGGGGCTGGCCCACGTGGGGGGAAATGT

AGGGAAAATGGCGCGGCACAGCTTCAGGAAAACAGTCGCTTTCAAACTAG

GGATTTCGTGGCTAATTGAGGTAAGACAGTAATTCTGCTCATAGATTATG

CATGTATGAACTACACATTGCGGGAGGTTTAAAAAAAANACTTTCTTAGT

CGCCAAAGTNCCGGAGCATGTCTTTAAG

>rnd-4_family-351#Unknown ( Recon Family Size = 35 Final Multiple Alignment Size = 29 )

AGATCCAGCCCGAACTGTGCGATGTAGTAGGGAGTTGTAGTTTCCAACAG

GCCAATATTCTACATAGTTTAGCGCANAAAACGTGGTAATTAACTACAAT

GACCATAATCCATTGCGCGCCTACTTGTCCGGTCTGTGTTTCTTTTACGC

CTGCTACGGAAGAGACAGAAGAATGCGCGATCGTGAAGGGATATAGAGAG

CAGCTGTTGCTTCGCGAGGTATCTCTACCTGAAAATACATGATCTAAGTG

ATTGATAGTTGGTATTCAGCAGTCATAAAAGTATGCCTTATTTACTTTGA

AGAACTACTAAAATAGTGATTTTGTCAGACAGCATAGGCAGCAGCTCTAT

AGAGATGAGATGATGACTTGGAATTAAGTAATAAAGTCATCAAATAAAAC

AAATGTAATATACACAACAATNAAATATTTTATTAAAGTAATGTGAATAA

ATGATGGTCAATAAGTGATAAGCAGTAATGGCAGTCACTACCATCATGGG

ACTTTTATTAATTGTTTTATTCTGCGTTGTTACAGCGTTCAACCCAAATA

ATGAAAACCGTGATAATTTTGTTTTTAAATGAACCGAAACCGAACCGACC

TCAAAAAGCACTAATCGCTCAGCACTATTGA

>rnd-4_family-628#Unknown ( Recon Family Size = 30 Final Multiple Alignment Size = 29 )

CAGTGTTAACGTGCAGAAAACCCAGGCTTTTACGAGAGCAGAAATCAGTG

AAGCA

>rnd-4_family-546#Unknown ( Recon Family Size = 30 Final Multiple Alignment Size = 29 )

TTACCTAAAATGGATCAATTGAAAGTGTGGGTTCACAGCTCCAATCCAGA

TGTGTTGGTCATTACTGAGACGTGGTTAAGGAAGAGTGTTTTGAATACTG

ATGTTA

>rnd-4_family-2704#Unknown ( Recon Family Size = 30 Final Multiple Alignment Size = 29 )

GCCCTTTACACTCGTACCCGTATACGGGTTGAAAATGGCAGATTTGGGCA

CTAATAAGCGCCTAAATTGGAACGCAGTGGCTGTACAGTAACATGCTATA

CATGACGTCAGAGCTCTGGCTCTG

>rnd-4_family-1016#DNA/TcMar-Tc1 ( Recon Family Size = 30 Final Multiple Alignment Size = 29 )

AGTGTCTAGGGTTTATCGAAAAAAATGGTGCGACAAACGAAAAACATCCA

GTCAGCGGCAGTCCTGTGTGACGAAAACGGCTCGTCGATGAGAGAGGTCG

AAGGAGAATGGCAAGAATCGTGCAAGCTAACAGGCGGGCCACAAACAGAC

AAATAACGGCGCAGTACAACAGTGGTGTGCAGAACGGCATCTCGGAACGC

ACAACTCGTCGGTCCTTGTCACGGATGGGCTATTGCAGCAGACGACCACA

CCGGGTTCCACTCCTATCAGCTAAAAACAAGAAGAAGCGGCTCCAGTGGG

CACGCGATCACCAACACTGGACAATTGAGGAGTGGAAAAACATTGCCTGG

TCCGACGAATCCCGGTTCCTGTTGCGTCACGCTGATGGCAGAGTCAGGAT

TTGGCGTAAGCAGCATGAGTCCATGGCCCCATCCTGCCTGGTGTCAACGG

TACAGGCTGGTGGCGGTGGTGTAATGGTGTGGGGAATGTTTTCCTGGCAC

ACGTTAGGTCC

>rnd-4_family-427#Unknown ( Recon Family Size = 36 Final Multiple Alignment Size = 28 )

TCAAATGTTGATATTTGGTTGCGTTGTCAACCAAACACAATTCAATATTA

CTTTTGTAATACAGTAAATAGCCTAAAGTTAAGGCTATTTTACAAACTAA

TGTAACAGTATTTATTCAACCTTAAAATGAGTAACACATCCACGGCCACA

TTTTGAGGTTACTGTAACTATACATTTCTTCTTACGTAATCATATAAAGC

GTGCGCGTTCAAGATAGCATGCATAGGTTATCGCAGGTCTGTGGAGATCT

TCACAATTGCTGTAATAATCTGCGATAACATTGAATCTGTTGCATAAATA

AAAAANAAATCTGAACATTGCACTTTCAACCATTTTAAAAAGCGCAACAA

GCACAGTGATAANACGTTTGNATGACAACTAAACCAAANATCTGACGTTG

TNTTCCCATTCGAACTTGGTTGTGCTTTTAGATGGTTGAAAGCATAGTGA

TAACACATTGGGAATTCAACAAACTTCTGGCTGTCTTTTTGAGTGGGTGA

ATA

>rnd-4_family-1173#Unknown ( Recon Family Size = 30 Final Multiple Alignment Size = 28 )

ACTGGGCACAGACGTCAGTTCAACGTCTAGTTTTGATTTACATTTGGTTG

AGTTGTCAACTAACGTGAATTCAACGTGAAATCAACAAAAAATGTCACCA

TGTCATTGGATTTAGGTTAAAAGTTGGGTGAAAAAAAGACGAAATTCCCT

TACGTTGATGACTTTTTGCAAATCCAATCAGTTTCCCACGTTGATTCAAC

GTCATCGCATCGAATTTTTTGTTTGAAATTACTTAGTTTTTGCTCAGTGG

GA

>rnd-4_family-294#Simple_repeat ( Recon Family Size = 28 Final Multiple Alignment Size = 27 )

ACACACACGCACACACAGTTACGCTAACCTTGTGGGGACANACAATTNAG

TCCCATTCAAAATCCTATTTTCCCTAACCCTAACCCTAACCCTAACCCCT

AAACCTAACCCTAACCCTAAACCTAACCCTAACTCCTAACCCCTAACCCT

AACCCTAATTCTAACCCTAACTCTAACCCTAACCCCTAAAATAGCCTTTG

TCCTCGTGGGGACCGGCGAAATGTCCCCACTTGGCCGAATTTTCCTTGTT

TTACTATCCTTGTGGGGACTTCTGGTCCCCACAAGNATAGTNAAACACGT

CCAACCACACACCAC

>rnd-4_family-711#buffer ( Recon Family Size = 53 Final Multiple Alignment Size = 27 )

TTGAATTTTTTTCNCCGAGTTGTAAAAGCAAAGCAGAAACTGGCTACTCT

TTAGTTGACTGATGTAGCTGGCAAGGTAACTGCTGTTTGACTTAACAGAA

AAAAAAGTGTTTATTCCCAGTGCTGAGCTAGTAGTGTAACTGACATGTAA

CGTTAGCTAATGTTTCATCCCCTCTCGACTGAGGTGAAGTGAATGAACTA

GCTAGCTAGCTAGCTAGCTACCACAGCCAGTTCAGCTCTAGCCTCTAGCT

ATCTGTCAGGTAGCAGTATCTAACAGCTGTTGTGTGTATGGAAATGTTTT

GTAGCCTTTGTTTTGTCCCGTTTCAACAGAAGTAAATGAACTTGGCTAGC

TTTCGCCAGTTGATGTACCGTTAGAGCTAGCCAAAAGTTGGCTAACGTTA

GCTAGCTAGCTCACTAACTTTAGCTATTATTTTTGCCTCAATCACACTAG

ACCTGAATCAAACGATGAAACCAGCGGCCAAACGATTGAAGACCGATATT

CTGACGTTTTTTCAGCGCAATGTGAGTTTTTATTAGTCAGTATGGCAATG

TAACGTTATTGATCTGTCAGTTTCCCTAGCTAATTCCCTACTTTTCACAC

TGACACGGTATAAACAGCTC

>rnd-4_family-365#Simple_repeat ( Recon Family Size = 32 Final Multiple Alignment Size = 27 )

AGATGATAATAATAAGATGGAGAGTGATGGAGTCTGTAGTTAGAGAGGAG

TTGAGGCAGTTCACTTCAGATACTTAGCTTGCACTTGTATGTGCTAGAGA

ACTGTGCTACATACCAATTATTTTCAATATGTATAAATATAGAGATAGAA

ATATATGTATGACTCCTGCTCTTTTTGTTTTACCTTTTAATAAATCACTT

AAATGGGGATCCCTCTTAATGGTTCATGAGTAATTTGGTATTGTTCGGCA

TGTTTAACGTTACCTGCCTAAGTACCTTAGATCGCAAGCCCATAATNAGT

CAATAGGGCTAACTGGCTAGCTACTAGTACTGTTAGCTAGCCAGATAGCC

ACGAAGNATTGTTAGCTAGCTAACGTAACCACGAAGAATTGACATCTATC

TTGCATATTATTCGTTTTAGGTCATTTTTGTCCTGTTTAGCTATCTGGCT

AGCTAGATTGTTACGATTCACATATCTAGCTGATAATTGTGAAGTNAAAC

GTTTGCTTTGTGTATATCTTGTTTCGTCTATTGTTGCCATTGTCCTGGCT

GGAAGAAGGTTCAGTGAATGGGGCTGTGNAGCGTGAGGTAATACAGTAGG

GGGAGTGCGAGTGCTTCTCAAATGTANTGCTTTATGCAT

>rnd-4_family-260#Unknown ( Recon Family Size = 30 Final Multiple Alignment Size = 27 )

AGGACACATCACTGCACTCTATACTCCTCTGTAAACTGGTCATCTCTGTA

TACCCGTCGCAAGACCCACTGGTTGATGCTTATTTATAAAACCCTCTTAG

GCCTCACTCCCCCCTATCTGAGATATCTACTGCAGCCCTCATCCTCCACA

TACAACACCCGTTCTGCCAGTCACATTCTGTTAAAGGTCCCCAAAGCACA

CACATCCCTGGGTCGCTCTCTTTTCAGTTCGCTGCAGCTAGCGACTGGAA

CGACC

>rnd-4_family-226#Unknown ( Recon Family Size = 33 Final Multiple Alignment Size = 27 )

GAACAAATGAGTCATAGTGGGCAGAACAAGCAAGGAGGTGGGCAGAGCCA

AGCACGAGCTAGCGAGATCCTATTGGCGCGTTCTAGCATNCATTTGCATA

TTTCCGTTAGGGAACGCCTACTCTGTGAAGTGTGCAATAACTCAATTCGC

CTTTGCACTCCTTCTAAACAACGCGATTTTTNAAAACTTTGGCAAAGGGT

NAAGTCTACAAAACTTAGTCCACTCTGTTCGTAACAGATTCTAGTTTTGG

GAACAGAAAACTGTATTGAGATCAAATGTTTCATCGATGAGAAAATTTGC

AGAATGTCGGCCAAAATCCATCTTCTCCCACTGCCGGCCACTGGGCTTCC

TCTCATCACCATATTTGGTAGTGAGTGGAAACGCCAACCGGATGCTTCAC

ATTTATACATCCGGTGAAATATCTGGCTCATTGTTCTATTGGT

>rnd-4_family-339#LINE/Rex1 ( Recon Family Size = 29 Final Multiple Alignment Size = 27 )

TACAATACCTGTGTAACGGCTCTCGTTGTTGNNNNNNNNNNNNNNNNNNN

NNNNNNNNNNNNNNNNNNNNNNNNNNNNNNNNNNNNNNNNNNNNNNNNNN

NNNNNNNNNNNNNNNNNNNNNNNNNNNNNNNNNNNNNNNNNNNNNNNNNN

NNNNNNNNNNNNNNNNNNNNNNNNNNNNNNNNNNNNNNNNNNNNNNNNNN

NNNNNNNNNNNNNNNNNNNNNNNNNNNNNNNNNNNNNNNNNNNNNNNNNN

NNNNNNNNNNNNNNNNNNNNNNNNNNNNNNNNNNGACATAGAATTTCCCA

CCCGAGTCACACCCTGACCACTCCAATTTGCTTACCGCCCAAATAGGTCC

ACAGACGATGCAATCTCAACCACACTGCACACTGCCCTAACCCATCTGGA

CAAGAGGAATACCTATGTGAGAATGCTGTTCATCGACTACAGCTCGGCAT

TCAACACCATAGTACCCTCCAAGCTCGTCATCAAGCTCGAGACCCTGGGT

CTCGACCCCGCCCTGTGCAACTGGGTACTGGACTTCCTGA

>rnd-4_family-551#Unknown ( Recon Family Size = 30 Final Multiple Alignment Size = 27 )

TATAGACGGGTTGGCTGACAACGTCACGAAAACGATGCGCGCGCTTCGAT

GNGGCCGGAGGCCGTGCGTTGTTGTGATTCTGAACGGCCAGATAGCTAGC

AACAATGACAAGAAGCTGCCATGTGGGGAATCGTAGGTGGCTCGTTTCAG

CTAGTTGTATCTTGTTCTTGATACCATGTCTTGTTTTGACTGATTTCACG

TCAATGCTAATATGGCAAAAATTCGCTAGCTAACCAACAACTGTAACGAT

GTATTTGAGAGACAAGTGCTCATTGTGCAAATGTATTTATGTTTTCAATA

AACATTTGGAGACGAAATATAGTTTACATGTTGTCAACAATCTAAGCCAA

CCCCGTCTGTTTTGCCCCATAGTTGCGCACGCGTCGGTTTTGTTGCTAAA

CAACCAACCCGTC

>rnd-4_family-165#Unknown ( Recon Family Size = 30 Final Multiple Alignment Size = 26 )

GTTGCAGGAATTNAATTCCTCTATGTTTTAACACCCAATTAAANTNAAAC

ACTCTGTCAATTCATAAGAATTTGTAAGACCCTTATTTGCATAAAATGGA

CAGAGACCAGTCTTAAAGTCAANCAGCAGCGTTTATTCNCGAGAGTACTG

ANCATTACACATTTTACCACAGGTTATAAACTGAAAATGACGTCATTAGT

TTTAGTACTAGCCCGTGTCATCTCCGCTCCGGTACAAAGGCCGTATAGTT

CTCGAGCCTTCCCTCCCTATTAAATAATTTACGACCGAGCCAAGGCCNGC

CTGTGTAGATAAGCATTCTAGCCAGTCTGGCGATAAGTTCATTCATTTCT

ACCAAGGAACAGACAGTCATTGTTCTAACTCTTGATTATATTTACACACA

TTATATTCGGTACTGGGATCAAAAAGAAAATTCATACATATACAGTAACA

TAATAGTATTCTGATTAGTACATATACAGTAACATAATAGTATTCTGATT

AGTCAGTCCTGATTGAAATGTATACATAATTAGTCATTATTGATAAAAAT

TCCCT

>rnd-4_family-670#Unknown ( Recon Family Size = 44 Final Multiple Alignment Size = 26 )

GCGTCATCTCTCAACGCTCCNATACCTGATAACGCACCCTCTAACTGGAG

TGAGTGCTCCCGGTCAAACTTCAAAACCGAGCGGTCAGGGCTCTTAGCGT

TGCGAGCTTTTGATGCCTCAAAAGCTGTGCTCGCAAGCTCTCTGAGTTGT

AAGATAGGCAAGCCAACGTGGGCTGTAGGGCCCAAGTAGGTAATGAAGGT

GGGATACATGTTCGACAGAAACATTTGTTTGAATGGTAACAGCTCTTCCA

TTCCTGTTTCAGTGAGTAGGCCAAAGTAAGCTGAACGAAGCCTATGATAG

TAAGCTTGTGGGTGTTCGTTCCGAGCTTGTTTGACGGTGTTAGCCAGTGA

GCTATCGTGTTTGCGAGTCGCAGAACCACTGAATTCTAATTTCAAAGCTG

TGGCAAGTTTAGCGTAGTCGTTTAGCACGTGTTGCTGTTGTAGACGAATG

AACCTCGTCACGTGTCTATTCGACGTTCGCTTCAACAGGTAAACCCTGTC

AGAACCCGTAGCGTTCGGGTAGCCATCCAACGCGTCCTCTATGTCAGCTA

GGAACGTCTCAGTATCGTTTGGCTGACCTGGAACGGGGTCAAAGGTGGGG

AAATTCTTGACGAGTTTGTCAAGGTATTCCGCGCCAAGCGGGCGGAGAGG

GTTGGCTTCATCCTGTGGGGAAATTGGGGCCGAAAGGCCAAGAGGAGAT

>rnd-4_family-276#LINE/L2 ( Recon Family Size = 27 Final Multiple Alignment Size = 26 )

GAAATCAGCATTTGTCCTACCACTTTTAAAAGGGGGAGATCCAACTCTTT

TAAATAATTATAGGCCAATCTCAAAGCTGTCACCCCTGGTGAAAATACTT

GAAACCCTTGTGAGTGAACAGCTAAAATAGTTTTTATTTACTAACTCTAT

TTTATCAATGTACCAATCGGGCTTCAGGAAGAAGCATAGCACAATTACAG

NNNNNNNNNNNNNNNNNNNNNNNNNNNNNNNNNNNNNNNNNNNNNNNNNN

NNNNNNNNNNNNNNNNNNNNNNNNNNNNNNNNNNNNNNNNNNNNNNNNNN

NNNNNNNNNNNNNNNNNNNNNNNNNNNNNNNNNNNNNNNNNNNNNNNNNN

NNNNNNNNNNNNNNNNNNNNNNNNNNNNNNNNNNNNNNNNNNNNNNNNNN

NNNNNNNNNNTCATGTCTGTTAAATTGTCTGTGNGTAATGGTGTGNCCCA

GGGCTCTGTACTTGGTCCCCTCTNATTCACTATTTATATAAATNATTTAG

ACAAAAATGTCCAAAATGCGCAACTTCATTTTTATGCTGATGATACTGTT

ATTTACTGTTGTGCCTCGTCTCTTACAAAAGCTTTCCAGAACTTGCAAAC

TGCTTTTTATACTGTTCAACATACCTTGTGTCAATT

>rnd-4_family-459#Unknown ( Recon Family Size = 36 Final Multiple Alignment Size = 26 )

GATCCGGGGGTCGGGGGTGGTCTGCCGGGCACTGGGATGACAACCCAACT

CGGGATGAGGAGGGCTTTTAGCGGGTGGAATAGTGGGGACCAATTGGAGG

TTTACTTAGTAGCAATGCAAATATCATGGTACAGCTATATAGACACTCAA

TCATCATGTTACTTTTTAATGGTANGTTTACAAACATATATTTTGATAAA

TAGAATTGAAACTTGTGTCTCATTATGGTAAGTGGTGAAATAAGTATAGC

CAGTTATAATTGTAATGGCTTAGCAGATAATAAGAAAAGACGATCAGTAT

TTACCTGGCTAAAAGAGAAGGAATATAATATCTATTGTTTACAGGAAACT

CATTCAACAATTTTAGATGAAGTTTTGTGGAAAAGGACTGGGGGGCGAAA

TATATTTCTCCCATGGGCAAAGAAATTCAAAAGGGGTGATGATATTAATT

AACAGTAATTTTGATCCAAATGTGCAAATTGTCCAAACAGATCNTCAAGG

TAGATGGATTATTTTAAATATGTTATTGGACAATAAACAGATATGGCTTA

TTAACCTATACGGTCCGAATAATGATGAT

>rnd-4_family-162#Unknown ( Recon Family Size = 37 Final Multiple Alignment Size = 26 )

TTGGTCACGTGCATGCGATGCATACATGTGTCACGTAAAGAGAGCAAGGG

TTGAGGGAATAGGGAATGTTTTTCCTAAACAAATTAGGGATTTCGGTAAC

AGAACTTTTCAGTCAGAAATGGCTGTAATTATGTTGCAGCTTCAGCAACA

CGGACAGCGCGATAAACACTTTGATGTTCTGTGGTGGTCGCTGCAGCAGG

GAGGAGAGAGAGACAACGGGTGCTAGTCACAGTCACTCGCCGTCTTTTTT

TAACACAGCGCAGCAAGTCTGAGCCCGGCACAGCACAATCAAATCAATTG

CTGGTCGGACTCCCTCTAGTCATTTGTGTGTCTTAATTATTTAATCAAAC

AGTGCGCTTAAAGCATCAGACAAGCTCAGTGCATATAGTTGATTTGATTA

AAACGCATAGGATGTGTCTATATATGGAAAAATACACGTTAAAACATTTC

GACCAATCGATTGGTCGAAAGAACAGACGACTCTCGGTCGACCAAGATTT

TTTTTA

>rnd-4_family-1898#LINE/Penelope ( Recon Family Size = 37 Final Multiple Alignment Size = 25 )

GCCTGGTCTCATAGACTAGACGTAACATAGTAAATGTAAATCCGGGACAC

TCAAATTAGTATGATATGTTACGTTTGGTATGGTTACATAAGACAGAAGG

TTACTTAAGGCAAAAACGAAAGTAGGGTGGACGTCTAGCAACCCAAAGGT

CGCGTGTTCGAATCTCATCACGGGCAACTTTAGCATCTTNGCAACTACTA

CTTTTTAGCTACTTCGCAACTACTTAGCATGTTAGCTAACCCTTTAGCTA

ACCCTAACCCTAACCCTTTAACCTAACCCTAACCCTAACCTTAACCCCTA

ACCCTAACCCCTAGCTAACGTTAGCCACACAGATAACGTTAGCCACAACA

AATTGNAATTCGTAACATATCATACGTATTGCAAATTCGTAACATATCGT

ACGAATTGTAATTCGTAACATATCATACGAAATGGATGATGGACATCCAC

AAATTAATACATACCATACGAAACGTAACATATCATACTAATTTGAGTGT

CCCGGATTTACGTTTACTATGTTACGTCTANNCCCGAGTC

>rnd-4_family-732#Unknown ( Recon Family Size = 35 Final Multiple Alignment Size = 25 )

TTTGACCTGCTGTGTTTCCTGTTGTAACATGTNATTGGTGTGTTGTTGAA

CATGAAAGATGACAAGTTACTATACCTACTGGTAAAATGGCTGTCAATGG

TCTCTGATCTTCTGTTACTCAATAGCTTGATCATTCTGTGTTCTCCAAGG

AACATATAATTTATTCTTATTAATCTGGCTAGTATGACAACGTAAATGAT

GTCAATCTGGTATGAAAAAACGTATTATTTATATTTATCTCGCGAGAAGG

ACAATGTTGGTCCAGTGTGTTTGTGTGTAAATGTACGCCTATACGCCATG

AAAGTGACTAGCCACTTCATCATTCTTCCGGAGTCAGTCCCCTAATTATT

TTTGAATGGGCTTGGCGATTATATATTACATTTGTGAAAATGCAGTTGTT

TAAAATATATATTTATCATAATATATTTGTGATTGTGGCTGAATTTACTC

CTGCCAGATGCCTTTTAATATAATGTTGCGCTGACGAACATTGCGCTACA

TCTTTGCCCTTTTAAGGCCATTCAAAAAGCTAACAAACATTAGAAAACTA

CAGGGCTACTTCCTTGAAAATGTTGCTGCACTTTCGATGCACGTTAACAC

AACCATTGGAACAAAGTGTTGTTGTTTCGTAAACTCCATGTT

>rnd-4_family-575#Unknown ( Recon Family Size = 29 Final Multiple Alignment Size = 25 )

TCCCAATAATCTCTCCTTTCTCCCGAAGTGTGCACTCGTTCACTNCTCCC

CACGAATNTAAAAGCATTGGATTGGTGTAGGCATGGGCTAGAGAGATTTC

CATATACCAGTCATTTCCTTTCAAATCCATGAAGGGAAGTGAACAAGTGC

ACACTTCGGGAGAAAGGAGAGA

>rnd-4_family-923#SINE? ( Recon Family Size = 28 Final Multiple Alignment Size = 25 )

TAGCGCTCATTCAATGACGATTTACTACTCTTTCCCATTGAAGGCCATAT

TGAGGTTAAATCAATGACTGGACTTTTTAATTTATAAAAACAAATGACTA

TTGAAAGTGCAAGTCAGTATCGTGCATAGTTGTTCTGGTCTCTGTGGTGA

TTTTAGAGCGTTGTTCATATTTCTACTTTTACCGCATACACTTTAACCCG

ATACCCAGTTACTGTGCTGTAGTCAGGATGGCCGAGCGGTCTAAGGCGCT

GCGTTCAGGTCGCAGTCTCCCATGGAGGCGTGGGTTCAAATCCCACTTCT

GACAGTTTTTTAGTGCCTCTCAGGAGTCATGCTGCTCAGCAGTAATAATA

AGAATGATGAAATAACAACTTCACACAGTGAAATAACAATTCATAATAAA

CACTCTCAAATGCAGACCTTCTGGAGCTGAGCAAAGATCAAGGTTAAATA

AGAAAACAGCTGGACTTAATAACAAATGAATGAGGGGAACATTTTGTGTT

ATTGCATTATAATGAGTTACTGCATTATCTTTCAGTTGTTAAATTTCACT

TTCTGTAGGCTATGCCTGTTGGCCCATTCACTCCTA

>rnd-4_family-1199#DNA/TcMar-Tc1 ( Recon Family Size = 28 Final Multiple Alignment Size = 25 )

CGAATAACACACAAATTATTGTATTTTTCTTGTCTATATTGAATACATAA

TTTAAACATTCACGTGTAGGTTGGAAAAAGTATGTGAACCCCTAGGCTAA

TGACTTCTCCAAAAGCTAATTGGAGTCAGGAGTCAGCTAACCTGGAGTCC

AATCAATGAGACGAGATTGGAGATGTTGGTTAGAGCTGCCTTGCCCTATA

AAAAAACACTCACAAAATTTGAGTTTGCTATTCACAAGAAGCATTGCCTG

ATGTGAACCATGCCTCGAACAAAAGAGATCTCAGAAGACCTAAGATTAAG

AATTGTTGACTTGCATAAAGCTGGAAAGGGTTACAAAAGTATCTCTAAAA

GCCTTGATGTTCATCAGTCCACGGTAAGACAAATTGTCTATAAATGGAGA

AAGTTCGGCACTGTTGCTACTCTCCCTAGGAGTGGCCGTCCTGCAAAGAT

GACTGCAAGAGCACAGCGCAGAATGCTCAATGAGGTTAAGAAGAATCCTA

GAGTGTCAGCTAAAGACTTACAGAAATCTCTGGAACATGCTAACATCTCT

GTTGACGAGTCTACGATACGTAAAACACTAAACAAGAATGGTGTTCATGG

GAGGACACCGCGGAAGAAGCCACTGCTGTCCAAAAAAAAACATTGCTGCA

CGTCTGAAGTTTGCAAAAGAGCACCT

>rnd-4_family-515#Unknown ( Recon Family Size = 27 Final Multiple Alignment Size = 25 )

CCACCCTGCATTCAAGAGCATGCTGTGTCTCAACCGATGGCGGGCACAAC

ACAAAAACCTCAGATGATGTAAAATAGGGTCTAAGCTACAACATATGCAA

AAAAATTCGAGTTTACGCGTTTTTAGATAATTGACGTTAAAAATAGTTTA

AAAATGACAATTTTTATTTAAAANATTTTTCAAGAAATGATAAATAGTTT

GACAACCCTGTTTGTAAGNTTTTAAATNATATCAANCTCAACCGTTTATC

TTTTCCAGTGATGAAGACATGGATGTCTCATGGTATGGTGGGGTATGCAA

AACGGGTCAACTTTGAGCACCTTTATCTCCTGAATGTTTTGGCATTCAGG

TCCAAAAAGTCACTTTCTGANCACTTCTACCATGGGCAAATATGTATGGA

AAGTTTCGTTCAAATCAAAAGGGGTGCTGTCAAAAAGTGATTGAANTCAA

>rnd-4_family-1634#Unknown ( Recon Family Size = 27 Final Multiple Alignment Size = 25 )

GCCTTCCCGCCTTTGGGACAACGACTCCCATTGTTAGGGCGGAGACATGA

GCATCTCGTCATTATATACAGATCTCTGGTGTAAATGGGAAGGTGCGCAC

AGCACAGAAGGAGCGAAGGAGACGAGACCAAAAAGACAACATGAGAACAA

GCGGACATAAACGCTCATAAAAAAAACAAAATNGATTCTTGCGATACAGG

CATTTGGAATATCGCGCAAAAACATACATTCGAATTAATCGAATTAATTC

GATATATCGCCCAGCCC

>rnd-4_family-358#DNA/hAT ( Recon Family Size = 33 Final Multiple Alignment Size = 25 )

TATAAGCTTCTGTCTTAAGCATCCTAAATAATGCCATAGATTCAGTTTTT

GAAAAATAGTCAACTTGAAGGCGAAAAAGGGAAGCATGATGGNAATCCAA

CTGATTGTAGGCTACTAAAGCCAACGACTTACCGCCGCGCCACGGAGTTT

TGATGAAATCAGCGGAGAAATAAAAACGTATATCTGATAATCGCACGCTG

CTATTTATTGCTGACCAGCAGATGTCACTAATGTGCATTGTGAACGGATA

ATGAAGCTCCGAGTAATGAACCGTTTTTCGGACACAATTTGNTGGAAAGC

CCCAAGGCTTCAGGGTAGCTTCACTTCACAAACAATTCAATAGAGTCCAC

ATATCAAACAATTGGAACTTTTAAATCACCCACAACCTGCATTTAGCACG

ACTGCCAAAGCAGGGAGAATTTAACAAAGAAGACTATCTAAACAATTTAA

ACTGAAACAACCATCTCAGTAATGGGTGCAACAACCTGCAATAAAGCAGG

CTGGGAAATATGATAATGATGGGTGTGGTTTTGAAGNGTCAGTGTTTTAC

TCTACACAGAGTTATAAACACTCACAAAAGCTCTGACGAAGGCCTTGAGG

CCGACACGT

>rnd-4_family-34#Unknown ( Recon Family Size = 34 Final Multiple Alignment Size = 25 )

GAAGCCTACCATTGACTATGTACANACCCAGCGTGCGACAGAGCTGCAGG

AGTTGTGACCCGTTTTTGTTGGTTATGTTGTCGTAGTTGTGTCTAGGGGG

GCATATGGGGAGGGAATGCTGTCACCTCCAGGTAGGTGTTTGTCCCCCTG

TGTGCTGAGGGTGTCAGGTTCTTGTCCAGTTCTGGCATTTAGGTCGCCAC

AGACTAGTACATGTCCCTGGGCCTGGAAATGGTTGATCTCCCCCTCTAGG

ATGGAGAAGCTGTCGTCGTTAAAGTATGGGGATTCTATCGGGGGATATAG

GTAGCACACANGAGGACATTTTTCTCTGTTGAGATAATTTCCTTATTAAT

TTCTAGCCAGATGTAAAATGTTCCTGTTTTGATTAATTTAATAGAGTGGG

TTAGGTCTGCTCTATACCAAATTAGCATACCCCCTGAGTCCCTTCCCTGT

TTCACACCTGGTAGTTTGGTGGATGGGACTACCAGCTCTCTGTAACCTAG

AGGGCAACCAGTGGGTCCGTCTCCTCTATACCATGTTTCTTGTAGGATGA

CAATGTCTGTATTTCCGATTTNTTTGATGAAGTCTGGGTTCCTGCTCTTT

AGGCCAAAGGCAGATGACCTCAGACCTTGTATATTCCAGGATGA

>rnd-4_family-262#Unknown ( Recon Family Size = 38 Final Multiple Alignment Size = 24 )

TAGTGATTCCTCACGAAACCAGGACAAAAAAANACCATGATTTTGGGGAT

TTTCACGAAATGTAATCCATAGAATGGTCCTTTGGAGGAAGGATTGTTGA

CATATATTTGACATTTGTATCTAAAAGCATAATTGAGAAATAATTNATCA

AAGTTGGCATATTTATGACATTTTGGCCTTACCCAGGCCTCCTTTAAGAC

TCCATAATGTTTCATCTGTAGGTGCTACAAAGCAAAAATACA

>rnd-4_family-525#Unknown ( Recon Family Size = 33 Final Multiple Alignment Size = 24 )

TGTTCGGCCATATTGGCACTCCCCAGNAGGAGCAGTCCTCCATAGGAATG

AATGGAATTCTACAGTATTTCAATTAAATGTTTCAAGGACAAAATTACAT

GTATTTAAGTATTTTTGTTGTTGTAGTGGGGACAGTAACATTAGAACTTT

CNAAAAATTATACTTTAAGGAAAATGTTTTATATATTTTTTCATTTTNAT

TTTTATGTTTAGCTCACATAATATAATTTAAAAGTATGCATTAAGGCGTC

TGTAATAGAATAAACGTGGCAAAAACAAATGTAGACGTTAATAAATGCAT

TTCTATAGCTTCCNAAATATTTTTTACAACGGTGGGGGAGTGCCAAGATG

GAGGCGCGGCGGCTTCAAAACAGCGCCCCCTGTNAGTCATCTAGCGTATA

TATAAATCAT

>rnd-4_family-701#LTR/Gypsy ( Recon Family Size = 56 Final Multiple Alignment Size = 24 )

CTCCTTCCTCTGCCTGGGCTCCCGTCGGCCNTGCAAACCGCGGAGGCTCT

TTTTACGCACGTCTTCCGGCACTACGGGATCCCGGAGGACATTGTGTCAG

ATCGTGGCCCTCAGTTCACCTCACGCGTATGGAGAGCGTTCATGGAACGN

CTGGGGGTCACGGTCAGCCTCACCTCCGGGTACCGNCCTCAANCTAATGG

GCAGGTGGAGAGGGTNAACCAGGAGGTGGGCAGGTTCCTGNGGTGTTACT

GTCAGGACCGGCCGGGGGAGTGGGCGGATTTTCTACCTTGGGCGGAGTAC

GCCCAGAATTCGCTCCGCCACTCCTCCACTAACCTCACTCCTTTCCAGTG

CGTNCTGGGNTATCAGCCGGCCCTGGCACCGTGGCATCCGAGCCAGACCG

AGGCTCCTGCGGTGGACGAGTGGTTTCGGCGCGCTGAGGAGGTCTGGAAC

GCTGCGCACGCGCGNCTCCAGCGCGCCGTCCGTCGNCANAAGGAGCAGGC

NGACCGCCACCGCAGTGAGGCCCCCGTGTTCCTCCCCGGGNGACCTGGTC

TGGCTCTCTACCAGGAACCTCC

>rnd-4_family-1127#Unknown ( Recon Family Size = 26 Final Multiple Alignment Size = 24 )

GATACGGTAGACCATTCCATTCTTGTGGGCCGGCTAAGGAGTATTGGTGT

CTCTGAGGGGTCTTTGGCCA

>rnd-4_family-1058#Unknown ( Recon Family Size = 32 Final Multiple Alignment Size = 23 )

TGCTAGANAACCCCACTAGCCACAATCCCAATACCTACACACCAAAACCC

CAAGACAAAACACACCACATACAAAAACCCATGCCACACCCTGGCCTGAC

CAAATAAATGAAGATAAACACAAAATACTTNGACCAGGGCGT

>rnd-4_family-131#Unknown ( Recon Family Size = 25 Final Multiple Alignment Size = 23 )

ATCACACCGACAGCGTCGTTGCGCAAAATGGTACGCAGCATCATCTGGAT

ATGTGTGCAACAAAAGTTNAACATTCACCTTCTGCTACCATTTCTGTCAA

GCCGTCTACGCATACAGTTTGACGCATACGTTCGATAAATCCAACGTATG

CACCACACCGAACGCACTGCAACTGCCTCTGCAACGCAATGCTGCAAGGC

AAACGCAGCGTTTCATTGGAAATGAATGTACTTCTGGTGTACCAAAATGC

AATGACGCTG

>rnd-4_family-49#Unknown ( Recon Family Size = 28 Final Multiple Alignment Size = 23 )

TTTGTGGGGAAAAACTCATTCTGATTGGCTGGGCCTGGCTCCCCAGTGGG

TGGGCCTGGCTCCCAAGTGGGTGGGCCTATGCCCTCCCAGGCCCACCCAT

GGCTGCGCCCCNGCCAGTCATGTGAAATCCATAGATTA

>rnd-4_family-2019#Unknown ( Recon Family Size = 32 Final Multiple Alignment Size = 23 )

ATAGGTTTCCATCCAATTGGCGACAGATTTTCATGCGAATATTCTAAAAT

CTNCATGAAAACAATATGCCATTTCAGAGTTTCCTTTAGGAAAATTTGGC

GCCGGACAACNGACCGGGAAGATTTTAATTTACCGGACATTTGAGAAATT

TACTGGACATCCATATGCATTCAGATCCGACCCGGCGCAGCCAGCGCCAT

CAATAAACGAGGGATGTTGGCCAAATGAGCCACATTTACGTGTCCTTAAA

AACAGCCTATAACAAATTACAAAAACACTATTCCTTGTGTTTCGACGTGT

AGCGTATGCAAAACTTTATAGCCTATTTTTATTGNTTTTGGTACTTTCCT

AAAACAACAATTGTCCACCTCACGGTTCAGCTGTCGGAGATTTGAGCACT

AAATGCATCAGGGCATTGCATGCATAGGCCTATAGGCTTAGGTGTCCACT

GTATGATATTAANATTTAAAAAAATATAACTATATAAAGGAAGAG

>rnd-4_family-242#DNA/TcMar-Tc1 ( Recon Family Size = 24 Final Multiple Alignment Size = 23 )

GAGACATACCCCAAGAGACTTGCAGCTGTAATTNCTGCAAAAGGTGGCTC

TACAAAGTATTGACTTTGGGGGGGTGAATAGTTATGCACGCTC

>rnd-4_family-75#Unknown ( Recon Family Size = 38 Final Multiple Alignment Size = 23 )

ACGGAGCAGCCATTACGAACACAGGCTGTAAAACAGTGATCACTAAGGTC

ATTACAGAAAACACCAGACTGATACCTATCAGGATTATTTGTGAGGATAA

CATCGAGGAGAGTAGCCTTTTCTGGGTGTTTGGAGTCATACCTTGTGGGA

TTGGTAATAATCTGAGAAAGATTTAGGGAGTCCCATTGCTTTAGGACTTG

GCCGGGTGGTTTAATTCAGANTTAGTGTAAGGGCCAGGAGAGAGCTTAGG

GCAGGTNNNNNNNNNNNNNNNNNNNNNNNNNNNNNNNNNNNNNNNNNNNN

NNNNNNNNNNNNNNNNNNNNNNNNNNNNNNNNNNNNNNNNNNNNNNNNNN

NNNNNNNNNNNNNNNNNNNNNNNNNNNNNNNNNNNNNNNNNNNNNNNNNN

NNNNNNNNNNNNNNNNNNNNNNNNNNNNNNNNNNNNNNNNNNNNNNNNNN

NNNNNN

>rnd-4_family-1603#Unknown ( Recon Family Size = 26 Final Multiple Alignment Size = 23 )

TAGCCCAATAACGGACTAAAGGAGCCTAACGTTACTCCTTAGTCCAAGGA

CCTTACATGTTCTNTTTAGAGGCGAATTGGCTCTGGCAGCCAAAACAGCC

AAATACTCCATCTAAACGTGAATCGATTCTCAATTGCGGTACGGTTCTAG

AAACATAAAGCCCTCTACTTTCATATCACATCAAAATAATTTCACAAATG

CAAAATATACGCTTACTGTACACTGTTTTAACCGGCTTTAACACAGTTGC

AGCCGGCAGTATTTTTCAGTGACGACACGTTTGTGGCGTCCGTCTTCCGT

TACACACAGGTGTTCGGAGCACGCTAGATAGCTAATTAGCACAGGTGGTC

GCCTCTGTCTTCCGTAAACAAGCGCTGTAACATGGAGATATGGCCGTGTG

GGAACACTAACCCTATAAAGGTGTGTATCAATTTAACCTTTTAGATTTCC

TAAAAGATATGAAAGATAAGGTCCTTACGTTTCCAAAACCGTACCGCAAG

CGATGCGTGTTAACGTTCA

>rnd-4_family-1294#Unknown ( Recon Family Size = 28 Final Multiple Alignment Size = 22 )

ATCGAGCCAATGTCTTAAGTAGTTGAACATGTTATTACTCCAACCTCGTG

AAAGTGACAAACTGACACGTTTTCATTTTCGTCAAAAACAACTTTATATC

GAAGGAGTGCCTTTGATTTGACGGCCTGCACATGCGCAGTTCGGCGCGAG

ACGACCGTTAGACCCGATGACGTGTATCTAGCCAACGTCGCCATGACATC

GCCTACAAGCGTGATCGGGGATTTCTATTGGAGAAGCAGTTTCTGCCTAT

CTTCATACTGTACTGTCTTTG

>rnd-4_family-1355#Unknown ( Recon Family Size = 23 Final Multiple Alignment Size = 22 )

AGCCCCACCCATCTCGTTTCGCTCTCGGAGCGCACACTTGACGCTCTGGC

CGATGATTTGTTT

>rnd-4_family-1206#LINE/L1 ( Recon Family Size = 22 Final Multiple Alignment Size = 22 )

TGGATTTCGACTATAAAATAATTACCAAATTGCTCGCCAAAAGACTAAAC

ACTCTTCTTCCCAAAATAATAAAAGCGGACCAAACTGGATTTATTAGAGA

CAGATACTCTTCTGATAACATTCGCCGTCTTTTTGATATTATTGATCAAG

TAAACGCACAGAAGACCCCTGTCCTGCTGGCTTCACTGGATGCTGAGAAG

GCGTTTGACAGGATGGAGTGGAGCTTTCTGTTTTCAGTCTTAGAAAAGTT

CAATATGGGCCCAAATTTTATTAAATGGATCAAATCACTATACTCTCATC

CAAATGCCATGGTGACTACTAATGGACTGAACTCTGACAGATTCCCTCTG

GAACGGGGCACAAGACAAGGGTGCTCGCTGTCCCCGCTGCTCTACTTGTT

GGGGGCGGAGCCTCTGGCAGAGCTGATAAGGAGCAATCCAAGTATTATGG

GTGTTTCTGCAGGCGGCCTGCAGCACAAGATTTCGCTTTACGCGGATGAT

GTCTTGCTCTACATATCCAACCCTGAGAAATCCCTCCCTCTCATTTTAGA

CACAATTGCTCAGTACGGCAAGTTTTCAGGNTATAAGATNAATTTTAACA

AATCCACTGTCTGCCCTCTCAATATTACACTCACCAGCTCTATG

>rnd-4_family-706#DNA/TcMar-Tc1 ( Recon Family Size = 23 Final Multiple Alignment Size = 21 )

TTAAGATCAAAATAAATATGAAAGGAGCAAAGGNCAGGTAAAAAATTATA

GGAAAACCCGCCATCGACTTCTGAAAACCTAACCCTGNGATAGTTTTATT

TTTCAGCGGGACAATTACACAAATTTTTATGCCAAAGACACACCAGAATG

GCTTTCCAAGAGGTGTTGAGTGTTCCTGAGTGGTCCAGTCTCAGTCCTGA

CTTAAATCTGCTTNAAAATCTGAGACAAGNTTTGAATATTGCTGTCCATC

AATGATTCCCAACCAAATTTACTGAGCTTGAGNAATTTTGACAAAAACAA

TGNATATATGTATGCTCCGTAAGAGGTTGGTAGAATCTTATTCAAAATNA

TTCACAGCCGTAATGGCTGCCAAAGGTGCTTCCACCAAGTATTAACTCTG

GGTGTGTGAAGACATACGCAATCAAGACATCTTCGTTTTNTATTTATTTG

GAAAATGTTCTATATTTTTCTTTCACTTTGAAAATGTGGAGTAGGTTGTG

TAGATCGGTAGGAAAAAAATCGAATGTAATCCTTTTTAGATTGAATTTTA

AGGCAGCAAAACGTGAAGACTGTGCAA

>rnd-4_family-186#Unknown ( Recon Family Size = 22 Final Multiple Alignment Size = 21 )

GCTAGCTAGCTAGCTCTGGTCCAACGTTAAGCCAACTCTC

>rnd-4_family-1042#Unknown ( Recon Family Size = 24 Final Multiple Alignment Size = 21 )

TGGGCATGGTCTAATTGACTCAACAACCAAAAACATCTAAGTTTAGCTTT

CTTAATGAACCAGAAAATCAATAGTTATTTCTGGTTGTTTATCAAAGTTA

GCTGGCTAACTCATTGATCCTGCTTTGTAGTATACC

>rnd-4_family-16#Unknown ( Recon Family Size = 25 Final Multiple Alignment Size = 21 )

CCATCGATTCCCTTTCTGTTGGTTTTGTCTTATTGGTTTCACCTGTTTCG

TGTTGGGGTTTGTTTNAGGGCTATTTAAGGCCGCTAGGCCCGCCGGGTGT

TCGCGTTTATTTTTCGTGTATTTGTTCTCCGGACTGTTTTGGTCCTGTTG

TTTGGGCTGGTCATTTTATNCGCCCTGTGTGTTGGCGTGACCGTTTTCGC

TGCGCCGGGGAATAAATTTGNCGATCCACCGAACCCTGCTCTCTGCGCCT

GACTCCACCCACCACTCCTAGTCGATCGTAACATA

>rnd-4_family-1480#Unknown ( Recon Family Size = 26 Final Multiple Alignment Size = 21 )

TGCAACAGTATCTTCTAAATCAAAGAGGAATACGCGAAGCATGAATATGT

TAGCTACATGAAGTAGCTAAGAGAAAACATTTAATGTAGCCAAAGATTAT

AGGGTCCCCTAGGAAACACTTATCAACACTTTGGTTCCTACCCTGTCACA

ATAACTCCTCCCTGGCATTTTAATTCGTTGTCATGTCAAACAACACTGTA

TTCAAAGTGCCCACTATTATATTCTAACTATAGAATTAGAATAATCATTC

TATTTCCATGATTCCAACAGTTCACCCAAGTGTTTTGATCTAAATCGCAA

GTCAAATCGCAATTGCAACATTTGGTTAAAAATAAGGCCTAGATTGTTTG

CCCATATCGTGCAGCCCTACGTGGCAGTGTGGAAATGATCTCAAATGAGT

GCAGGAAATGCAGGAAATTATTTTTGGTTGAAGTTGAACAGTATAAAACA

ATCCGAATGGAGAAAGACCCATTGAAATCACTTAGAATG

>rnd-4_family-1995#Unknown ( Recon Family Size = 25 Final Multiple Alignment Size = 21 )

TATCAAGTTGTAAATTACGGTGCATGGAGAATGGTGTTATTTCTATCGGA

AAAAATNAAGTAGATGNTTTTTCCACTTGGAACCGACAGGTTCGCTCGAC

CTTATTCATGGTTTCCTCGCGCGTAAAGGTGGTGGAATTAGGGAATTAAG

TCANGGTCAGAATACGGCNTATCTGTAGACATCTCCGCCACACCTTCATT

TATTCGATCTCCNCCCAAAACGAATAGCGGGCGAATAGCATGCTATTCAT

GCTTAAGTTCAAGGTCAGAATACGCGTAGAGAGAAGTGCATAAAAAGGGA

ATTGCGTTCCAANTACGCACGCTTAACTCGGGCCGGAATCGGGCACAGTG

TCCCAGTGTGTAATGAATACGGTCTTGAACAGTCTTTTTATGCTTCCT

>rnd-4_family-140#DNA/hAT ( Recon Family Size = 24 Final Multiple Alignment Size = 21 )

CTTCGAAGCAGTGTGCCGATGCTTGTATCACTTTATCAGAAGCACGTGAT

CAATGACGTCCGAAGCTTCGTTTCGTCGAACAACCACCTGATTGGTTTGG

TATTGGTTTCGGTAGAAGACAAAAAGGCTACCCTGCGCACGCGCTACGGC

GTGTGGGACGTCGTCTATAATAATTTGGCTGTTCTAAATTATTTTGACCA

GCAGGTGCCACTAGTGTACANTGTGTTGATCAAAGCCTCGAGTAATGAAC

CTATTTTCGACACAATTGGCTGGAAATGCTCAACGCTTCAGGAAGCTTNG

TTTAGTTTCCCAATCACTAGGTGGGGGTATTGCGTCTAACATTGGTTGGC

ACACCACCATAAAATCGCAGCAAAGAAGAAAACATTAAAAGTAACATTTG

AACTTATTACATATTTGTAAA

>rnd-4_family-369#Unknown ( Recon Family Size = 23 Final Multiple Alignment Size = 21 )

CCCTTGGCAGTTACCGTCCCTGTAAACGTCACACCTCGACGTGTGACCAA

TCAAATCGTTTTCACATATTGTGGTCAAAAATGNTCTATTATATTGACAA

GATAGTCGAGTGACCGCTCAAGCGATGGAACTACATGTCCTCAAAGATGG

AAGGCGGGAGGGAGGAGGCCAGATCAGGTGGGACCATTCTAGCCAATGAG

AGGGCAGATACGTGTCTGAACAACAATATAAAGTCGTTTTTTCAAAGTTT

CCGAAATGCCACGTGCGTCCACTTATATCAGCGAATTCCTAACAACCTAA

CCATTACGAAACTTNTANCAGATCAAATAAGCCTCACGTAANAAANTAGC

AATTACATTTTTTGTTGACCAAATTGGACACTCATTGACCTCCATGCAAA

AATGCCTCACTTCGTGGCCTGTTTCGTGGACAGATTTTGGGCGGAGTAAA

CTCTCCCTTCGCCTCTTCCTCTCTGTTGTGGT

>rnd-4_family-3387#DNA/hAT-Charlie ( Recon Family Size = 28 Final Multiple Alignment Size = 21 )

TTGCAGATATGAAACAAACAGACAATCATAATATCAGTCAAAAATATAAA

ATTCCCAGTTTATGCTACAAAACCAACTTTATAAGAGGTTTTAAAAATAG

GTTATATTTGACTCAAAATTCCATGACGTACAGTAAGGCATTGTTGGCAG

AATAGATGGATGCAGTTCAATGCATGATTAATATAATTCACCAATACATT

TCTTGGTAGTCCAAAAAATATTGCTATCAGGTTGTAAATCACAGCTGGCC

TGGTACATTGTTTGCTGCCTCCACCCATTCGGGATGCACTGTTTCAGTTT

CAATGACTCAATATTTTGAACAGAAACGGACGACTGTAACTAAGGCCGGG

AATGTCAATACAATCAAACTAGCAAGGGCAATGATCACAAGTCAGTCATA

ACGTGGCTAATAGGCTAGCGCACATGTGTCAAACACAAGGCCCGCGGGCC

GAATAGGACACACAAGCGAGCATAAATGAGTCGACACTTGAGTCGTTTAC

TTTATGAAATTACAGCCCGATGCGCTGCTTTCGTGTGTCCCGTTTACAGC

GCGCAGCGGAGGGAAAGTGTACAGACACATGCCACAGTCCTAGCTAGAGG

CTACTAAAATGTTGCAGTCTGGCTTCGGTTTTTT

>rnd-4_family-1219#Unknown ( Recon Family Size = 23 Final Multiple Alignment Size = 20 )

CAAGGACGATACTCGTTAGTATCGTGGCAAGGAAACAAAACGCGAAGCGG

ATTTAACTTCTTTAGGAAAACAGCCCTAATGTTGGAAACAAACATTATGT

TGTCATCCAGAGTCACATTTTCCAAGCCAAACAATATTTTACATACAGCA

GGTTTTTAAAGGACCAAAGAGTTTGGTCTGCTTCGTGTTTTCGTTTTTGC

CATGGAAAAAATATTGCGATACTGGTATCCACCTGGCCCTAA

>rnd-4_family-449#Unknown ( Recon Family Size = 22 Final Multiple Alignment Size = 20 )

CTAGAAGAAAAATAAACGCACACCTATTTAGGCGAGGTGCTGGCTAGCGG

AGTAGAAAACTTGAAAATAAAGGAGAGCCGCACACTCTAGGAGCTCAGAT

GCAAAAATGTAATAACCAACGTTTCGACAGCCAAGCTGTCTTCATCAGGG

TATAATCACAAACACTGCGGGATGACTCGTTTATATAGTGTCAAAAGACA

CACAGGTGTCTGTAATCATGGCCAAGAGTGGCCTAATATCATTGGTTAAT

TCTCAAATATTAAAATGGCATACAAAGAACAGCATACAAAAAACAAATGG

ATAGCATACGATCATAGATTC

>rnd-4_family-229#SINE? ( Recon Family Size = 20 Final Multiple Alignment Size = 20 )

ACATTGTTGTTCAACTTTAATAATCTAAGACTGCAAGGCCTGAGAATTAT

TGTGTCTGAATCTGCATCGCAAGATTGAAGGAAAGAGTATCTTCGTACTT

GTTAAATATCATTAGCTTTCCTCATTCGGACAGGTTCACAACTTTGATGT

CTGTTGTTGCTAAGCAACGTCTGTATGATGTCAAATAGGGGTGTATCGCG

TAGACTGCGTGGCCTAATGGATAAGGCGTCTGACTTCGAATCAGAAGATT

GAGGGTTCGAGTCCCTTCGTGGTCGTTTAATTTCATTAGCTCAGCTAATT

TGNACATGTTTGCAATTTCCCNAAATAAGGCATTGGTCTTGTTGCTTGGA

CATTGTTATTCAACTTTAATAATCTTAGACTGCAAGGCCTGAGAAATATT

GTGTCTGATTCTGCATCGCAAGATTGAGGGAAANGGTCTCTTCGTACTTG

TTAAATATCACTAGCTTTCCTCATTCGGACAGGNTCACAACTTTGATGTC

TACTGTTGCATAGCAAAGTCTGTATGATGTCAAATAGGGTCGTATCGCGG

AGAAGGCGTTGCCTCGTGGATATGGCGTCTTACTTCGAAACAGAAGATTG

AGGTTTCGAGTCCCTTCGTGGTCGTTTAATTTCATTAGCTCAGCTAATTT

GAACAT

>rnd-4_family-537#Unknown ( Recon Family Size = 30 Final Multiple Alignment Size = 20 )

AGAATGCGGAGGGCTCCGCATTGACATGATTGGTTGACGGTAGGTGGGGG

CGGGAGGTCCTGTATAAACACAAACTCACTTCCTTGACAACTTCCTTCAC

AACAGCTCTGCTCCGCGAAGCGCAAGAAGTATGAATGCCCTGACTTCTGC

GGAGGCCGTATCGCNGTAAATGCTGTACGGCCACTGCAGACGTCGGATTG

ACCATGCAGAGCCTT

>rnd-4_family-1024#Unknown ( Recon Family Size = 22 Final Multiple Alignment Size = 20 )

CGCTCTCTGTAGTGTTTTTGCGGACTGTAGTGGATACTGTAGTATACTGT

AGTATTTTTCGACTGTAGTATTTACTGTAGTAAAATGACTGTAGTATATA

CATTACTGTAGTATTGTTTTTGCGGACTGTAGTATTTACTATAGTGTTTT

TGTTTTAATTATCTTTGACATAGAAGTGGGGGCTTTCTCCTTGAGGAAAC

CTACTGGAGAAATACTAAAAGAGCACATTTTCCATAACCTGTAGGTAGGT

AGGACTGGGGTCTGAACGGATAGAGCTTCTGCTCTTTTCTATAACCTGTA

GGGAACACAATATATGGTCTATACTTGGCATGTAGGTTTCTCACTTATGG

GTGGCACAAATTGGGATATGGGCAGGGTATATGCAAATTAAATACTGTAG

TATTTACT

>rnd-4_family-765#Unknown ( Recon Family Size = 24 Final Multiple Alignment Size = 20 )

CCTGCTGGCTACTCTTCTTCCGTGGCTTAACCCAACGAGAGAAGGTCACA

AGTGTTTCCCTAAAAGCTGTCTGGGTTTAAACATCTTCTNTTGTACAGAA

AGTGAATAGCTTAATCAATTGATAGTGACAGAATTAGATTACTTCCCAAG

CAAAGTCATTTTTTGTCTCCTCGGCTATAGAAGGTTGTAGCTCAGCCTCT

GAATGTCAAAGAGACGAAACAATGATATCCCCATATGCATCGGAGTCACG

TCTTTCCCGGGTATTTTACAGCTTGTTTCTAGCATAAAGCATCGCGGACC

AAAGCGCTGTTATAGATCACTTTATATAATCGGATCCGTTTTATTTTCTT

CAAAATCTTAAGCTAACAANAGGTAGGCCTGTGCTTTTTATT

>rnd-4_family-995#Unknown ( Recon Family Size = 23 Final Multiple Alignment Size = 19 )

TGGACAGTATATGGATAGAATATGTAGTATAGCTGAAGAATAGTATATGT

ACAGCAATAGTTAAATAGGATAGGCCTTGACTAGAATACAGTATACATAT

GAAGTGGGTAAAACAGTATGTAAACATTATTAAAGTGACCAGTGTTCCAT

GACTATGTACATAGGGCAGCAGCCTCTAAGGTGCAGGGTAGAGTAACCGG

GTGGTAGCCGGCTAGTGACAGTGACTAAAGTTCAGGGCAGGGTACTGGGC

GGAGGCCGGCTAGTGGTGACTATTTAACAGTCTGATGGCCATGAGATAGA

AGCTGTTTTTCAGTCTCTCGGTCCCAGCTTTGATGCACCTGTAATG

>rnd-4_family-99#Unknown ( Recon Family Size = 32 Final Multiple Alignment Size = 19 )

TACCTGCTGTTTATTNTACTCTTATGCATNGTGACNTATACCCCTACCTA

CNTGTACATATTACCTAAATTACCTCGACTACCTCGTACCCCCGCACATT

GACTCGGTACCGGTACCCCCTGTATATAGCTCTTCGTTATTTTATTGTAT

TGTTCTTNTTACATTTATTTACTTTAGTTTATTTAGTAAATATTTTCTTA

ACTCTAATTTTTCTTGAAACTGCATTGTTGGTTAAGGGG

>rnd-4_family-471#Unknown ( Recon Family Size = 21 Final Multiple Alignment Size = 19 )

TGTGACGACCCTCCCACTCTGTCTGCCGTATTCTCTCTTTGTTCTTGTTT

CCTTANTAGGATGCCGGTGGGCGGAGCTGGGAGGGTCGTCAGCNACATGG

GAAACACCTGGGCCCGGTGTGTCCCGGGATAAATANACCTCTTCCACATT

CATGGAGGAGACTCTCCATGCAGACACCTTTACAGATTTTGTTGTGCATC

TTGGTGGCTTTTTGGTTGTTTGCTTTGGCACCTTTCAACACCCCGCATTA

TCACATTCATGCACGCAANACACTCACTTACACTACTGATTACACACACC

ATTGTATATTGTACTTAGTTACTTTAGTTAATAAATATATATTTTGTTAC

TCCTTATCTCCGCGTTGTCTCCCTTTTGTTACGG

>rnd-4_family-1607#Unknown ( Recon Family Size = 20 Final Multiple Alignment Size = 19 )

GAAGCCATCGGCCAAGTATGTACCAGATCACCTTGAGTTTATTTGAGTTT

TTAAAGCTACTTTGAGATTCTGTGTGTAATGAAAAGTGCTATATAGAATA

AATCATAATTATTATTATTTATCCTACAGGGGAGGAGGAGCCCTTGTCAT

AATGGCTTTGCGATATGATGGACAGCCTTCTGTTTGCTACTAAACAGGTC

CTAATATCTTAGTCTGTGTGCCAGTTCATTCTCTCTTTTGACTTTGTTGT

GGCAAGCAGAAACACACTGGAATTTAGGAAATCCCACCAAACCACCGAAT

TATTACTTCAATTAAAGGGACAAATTATGTGGTCGCTTGGGGTAATGCAA

AAATCCCCTTTGGTTATCACATGTAGGCACATGTGTAGTTTATGAGACAG

ATCTTTGATTTAGTAGCTGTTCATTTGTTAGTAAATGTTGGGAGCTGTGA

CTCTTATTTATTCTACTGGAAGGCTGTCCTCTCCTCTGCTTACAGTAGAA

AGGCCTTGGAGCGATCCACTGAGACGCAGTGAGCATAAGCACGACAAAAG

ACAGGAAAACAGTGACCATGTTTCGGGCGACTAACATGCATTTCCCCACG

ATCAACTGATCCTAA

>rnd-4_family-3840#Unknown ( Recon Family Size = 21 Final Multiple Alignment Size = 19 )

AACACAAACTAATAGTGTAATTATAGAACGCTTGTGGATTTATATTAAGA

NCAAAGTGAAAACAACATCGTTGCATGTGCTGCATTGACCATGCAGACTG

AACGAAAGTGTCTCGTGGTCGAGCAACAACAAATGCGCTCCTTGAGTGAC

GGGGCGGGGCTAGGTCTGCGTGGAAAGCGGCACGGAGAGAGAGCGGAGAG

AGATGACTCAAGTAGCGGAGTAAACTATAAAAATGGACGTTACACACGGC

GTATCACATTTAACAAACCAAACATTCAAATACCGTTATAGAAGGTAAAG

TAAAAACCCAAACCGGTCCGTGCATCAATACCGGTATATAGTAAAATACG

GTATACCGCCCAGCCCT

>rnd-4_family-384#Unknown ( Recon Family Size = 25 Final Multiple Alignment Size = 19 )

ATTGACAATACAANATTCATAACAGTTACATACCAATAAAACAATGTTAA

AGACTAGCCTGCTGGCCTTACTGGGTCGATAGGAACATTAGCCCGAGCTG

TTTAGGAGGGATATCACACCTGGCACAAATNATTGTCTAGTTCTGTTTTT

CCCGCCGAGGGGTGCCCTATACCTGCACCCAGAGGGGAGTAGTTCAAAGT

CCGGGTACAGGGGGTGGCTTGGGTCTAAAATNATTTTGTGAGCCTTGCCG

AGGGCCCTGACCTTAAAGATCTCATCCAGGCCTGTCTGTTTGACTCCAAG

TACCTTGCTTGCTGTGGTGATAATCCTTCTCAGCATATTTCTCTGGCTGA

CAGTGGCATTGCCAAACCAACAAACAATACAAAAAGTTAAAATACTCTCA

ATGAAAGATTTGTAAAACAGAGTCAGTATAGTACAGTCTACATTAAAAGA

TCCCAGCTTTTTGAGAAAGTACGGTCTCTGTTGAATCTTTTTGTAGATCA

GGTCTGTACATTTACTT

>rnd-4_family-1539#Unknown ( Recon Family Size = 25 Final Multiple Alignment Size = 19 )

TAAAGGGATAGTTCACCCAAATTACAAAATTACATATTGGTTTCCTTACC

CTGTAAGCAGTCTATGGACAAGGTATGACAGCAATCCATGCTTTGGTTTT

GTTTACCTGGCACTGTTTCAAATGCTAACGTTTTAGCATTTGTGGCACAA

ATCCCATTCAAGTCAATGGTACCNATATTAGCATTTTTCACGCATCATGT

CCAAATCATCTTTAAGT

>rnd-4_family-674#Unknown ( Recon Family Size = 20 Final Multiple Alignment Size = 19 )

TTGGGGTCAATTCCATTTCAATTCCAGTCAATTCAGGAAGTAAACCAAAT

TCCAATTCCAATTTTCCTCAATGAAGCGTTGAAGAGAATTGGAATTGGAA

TTTCGGTTTACTTCCTGAATTGACTGGAATTGAAATGGAATTGACCCCAA

CCC

>rnd-4_family-2206#Unknown ( Recon Family Size = 19 Final Multiple Alignment Size = 19 )

CGGGAATTTTGCTTAAATTCATCAAAAAAGTTAGCTTATAACAGTGAACC

TTTTTTGTGGGATACACATAAGGCAATTCTAGGTCTTGTGGCATATTTTG

GTTAAACTATCCCCAATTCAATGGAATTGCAACCCTCTGCATGCACAGTG

CANTCTTCCGTCACATGCACAGTGCATTCTTCCATCACATGTACAGCTGA

TTCTCAAGATCTTGCACACTAATGAGATGCTATTGAGCCCACACTACTAC

ACTGTCTGAGCCAAGGACTACATGCTTTCTGGTAAGTTTTGATTACAATA

CTGGGTGGGGTGAATATATTTTATATGACATACATGATTTTTTGTTAACT

AGTAAATAGTAGCCTACAGCAAAGTGTGTTTAAATCATTTCTAACTTGTT

AACAATTTCTGCTAGTTAGTTTTTGCTACCATGTGGGTTTTAGCTTGCTT

GAGCCTGCTAACTGAGGAGTGTTAATTCACCTGTTTCCATACATGTTTCA

TTTTAAAACATTTATCTTACAAAGGAGTTGTATTTTTTTTTGACTCATTT

TTTTCTAATCTTTACAGGAAAACGCCACGGGCACTATCTGATGCGTGGAG

ACATTTCAC

>rnd-4_family-2164#Unknown ( Recon Family Size = 21 Final Multiple Alignment Size = 19 )

TGTCAACGTTGTGAACAGAGTGCCCCATGGTGGCGGTGGGGTTATGGTAT

GGGCAGGCATAAGCTACGGACAACGAACACAATTGCATTTTATCGATGGC

AATTTGAATGCACAGAAATACCGTGACGAGATCCTGAGGCCCATTGTGAG

GCCCATTTTTTTAAAGGTANNNNNNNNNNNNNNNNNNNNNNNNNNNNNNN

NNNNNNNNNNNNNNNNNNNNNNNNNNNNNNNNNNNNNNNNNNNNNNNNNN

NNNNNNNNNNNNNNNNNNNAC

>rnd-4_family-601#DNA/hAT ( Recon Family Size = 22 Final Multiple Alignment Size = 19 )

CACTCCAACCCTGTTCCTGGAGAGCTACCGTCCTGTAGGTTTTCGCTCCA

ACCCTAATCTAGCGCACCTGATTAATTAGCTGGTTGATAAACTGAATCAG

GTTAGTTACAACTGGGGTTGGAGCGAAAACCTACAGGAGGGTAGCTCTCC

AGGAACGGGGTTGGAG

>rnd-4_family-232#DNA/TcMar-Tc1 ( Recon Family Size = 23 Final Multiple Alignment Size = 18 )

AAGGCTACCCGAAACGTTTGACCCAAGTTAAACAATTTAAAGGCAATGCT

ACCAAATACTAATTGAGTGTATGTAAACTTCTGACCCACTGGGAATGTGA

TGAAAGAAATAAAAGCTGAAATAAATCATTCTCTCTACTATTATTCTGAC

ATTTCACGTTCTTAAAATAAAGTGGTGATCCTAACCGACTTCTAAGACAG

GGNATTTTTACTAGGATTAAATGTCAGGAATTGTGAAAAACTGAGTTTNA

ATGTATCCGGCTNAGGTGTATGTAAACTTCCGACTTCAACCGTATA

>rnd-4_family-1046#Unknown ( Recon Family Size = 24 Final Multiple Alignment Size = 18 )

CCCAAAATAAGCCCATAGAAACGCATTGGGCTTATTTTGGACTCTCGCTT

CGCCTCTGATACTATGCATGCATACAAGGACCGGACATTTTTCGTTAAGA

GCTTAATGGAAACATGCAACAATAGCAAGCCTATCGTCTTAGTCAAAAGG

CTATAGCCTATTATTGAACATGCAACTCCTGTAATGAAGCAGCTAATAAA

ACGCAATTAGCGGAAAACACAGTTCTAAACAGCGCACCTAATGCGAGCGG

TTCCGTATGTGACAGAGATGAANATCTGTTAGAAACTTAGAAAGAGGGGN

AATCTAATAGCAACAACNATGATGGGTTGCTAATATGACTAGGATTGTGC

CTTTGGCTTCTGGACAATGAAANAAAGTTGATATGAAAACCAATAGAACA

GGAGAGAANTGCTGGTTAATGGCATGAGGAAGTCTATAACATAATTTCCT

CGTTTCTACGGATGGATTTGGCAATGCAAGACACGCCTCANTATTTGAAG

TAAAACGTNGAGGTTTCAAACAATTTTACTGCCTCAAGCTCNCATTGCAA

AGCGGTGGGNGACNCGCTGATNGTCAGCGGTAGGCAGGCTACNGCCTATG

CACCTGAATGGCAAGTGGGAGGCACGCTCNAATTACGAGTTGAGGAATAG

AAATAGCNCCTTTTTAATC

>rnd-4_family-69#Unknown ( Recon Family Size = 19 Final Multiple Alignment Size = 18 )

TAAAATCTGTTCATATACACCCCCAGAAAGAATATGACACGTNTTTTCTG

GCAAGCGAGCACTTAGATATGGTCATTTTCACGTTTTCATAAATTCTTAG

AATGTTTGGGAATTACGTANAGTAAGGCGTTTGCGAAAATTCTATAGCGA

TATAGANTGGGAAAGCGGCCGTGCGTTTGGACAATTAATAGACACTGCAG

TAAATAAAACCTAATAAAAACATCTGTCTTGTCCAGGACCGGAGTCTACG

CAGACCGGTGCGCCATAGCCAATCAGAGCTACAGTAGGCCTATATGCAAA

TAAGCCATTTGCCACACGGGCCTGCCATCATTCACTTTGAACTGGACTGT

GTGTTTACAGGCAGTNGCAACAGCGCGACTTTAGATCATTAGAACGCATT

CGCCAAAAGCCACAAAATACACCTGAATGGATTTCTGCAAATATGTAAAC

ACCACGGGAGTCCTCTTACGTTTGGGAACTTTACAGTCCTATTGATCAAA

CAACCATGAAAAGGTAGGCTCTCTCTCCCTCAGTTGCCATGCACATCA

>rnd-4_family-3143#Unknown ( Recon Family Size = 20 Final Multiple Alignment Size = 18 )

AGGTTGTAATATGGCTTTTTTTCTGGCTTGGCTTCCCCAGTGATTTTACC

CACGCACCGCTACT

>rnd-4_family-1839#LINE/L2 ( Recon Family Size = 24 Final Multiple Alignment Size = 18 )

TGTAATGCTCTCCTGTCTGGTCTACCCAAGAAAGCCGTTGGTCAACTGCA

AAACATACAGAATGCTGCAGCACACGTTACACCGATTTCAAGGTCTCTGC

ACTGGCTGCCTGTAAGTTTCAGAATTNATTTTAAGATTCTTCTATTGGTT

TTTAAATCAATCCACGATTGTGCACCCCAATACATGTCAGACATGCTTTT

AAGTTATGTACCCAGTAGGTCCCTCAGGTCCTCTGGCACTGGCCTTTTAA

CTATCCCAAAGCCTAGGACCAAGAGGCATGGAGAGGCAGCCTTTAGTTAC

TATGCCCCCAGCCTCTGGAATAGCCTGCCAGAGAACCTGAGGGGGCCGAA

ACTGTGGACATNTTTNAAAGAGATCTTAAAACACACCTTTTTAGCTTTGC

NTTTCCTTAGGGTGCTTTTTAGTCGTTCAGTTTTCATTGTTATTCTTTAG

TTTTTTATNCTCTTATGTCTGTTGTGTAGTAAATATNTTCATGGTTTTAA

TNATTTTTTTCCCGTGAAGCACACTGTGTTGCATTCCATGTCTGAAATGT

GCTGTATAAAT

>rnd-4_family-1474#Unknown ( Recon Family Size = 27 Final Multiple Alignment Size = 18 )

CTTAAAACACTATAAAACTAATTGGTAGGTCTACCTTTACTTGTTACTTC

TGTGAACTTTCATTATCCTCCCTCCTCATGAGGGAGAGAAATNAGNAAAT

ATCTTAAAGATATGTGGGTTTTTGGTAACGGAATTNCAAGGCACAAGGCA

ATGTTTCTTAAACTTACAGAAGGCAGAAAATCATNTCCAAAACTAAATAT

AAGTGTTGATATTAGTTGGCAGGGGTCTTTACTTCAACATTATTGTGTTT

TGATGTATTTCTAATACCTTTTAAGACTTTTTCTGGTAGATGTTTTCTAA

GACCCCTTTTCCATCTGTTTGACCAGAAATCAAAGCCTTTGCTTATTCCN

AATTTTTAGAATGAAAATGGTTGAAAAAT

>rnd-4_family-57#Unknown ( Recon Family Size = 19 Final Multiple Alignment Size = 18 )

CTGGGTTCGAACCCGGGTCGTCTCCACAAGACTGTGTTAGCCCGCTGAGC

TAAAGCCTAGGCGTTAGCTCGGGGAGCTAACGCAAGTCTTCAGGTCTCAG

GCAAGGTTACTCATCACGCGAGCGTGGTTCACCGAACCACCTCCGTTACA

>rnd-4_family-1431#Unknown ( Recon Family Size = 42 Final Multiple Alignment Size = 18 )

TTCTTTTAGGCTATTTACAGAAAAATAAGTCCTGGATTGCTTTGCTAGCA

ATAGACAACAAGTAGACAACAAAAAATCCACAGGGNCCGAACAATTGGAT

CCCGGTCTGCTTAAGTGCGCAGCGCCATCATTGCTGGCTCAGTAACCCAC

ATTTTTCATTTAACATTGTTNTTAGGAANTATTCCAAAAGCATTTGTGCT

GCCACTCCATAAGGGCGGGGATAGTAGTGATCTTAATAATTATCGCCCCA

TTTCAAGGCTTCCTTGTCTAGCTAAGATTCTTGAATCCTTGGTAAATGTA

CAACTTTGCTCTTTTTTATCTGAGAAATGTATTTTGAATGTAAACCAATC

AGGGTTTAGGCCTGGGCATAGCACTATTACAGCAANCACTTTAGTTGTTA

ATGATCTTGTCAATGCTTTAGACGCTAAAA

>rnd-4_family-2332#Unknown ( Recon Family Size = 22 Final Multiple Alignment Size = 18 )

GACACAAAAGACTGTAAAAACACCAGNAAATCAGCTCCAAGTGATTTTAA

TTTTGGAAATCTGTTCCAAAGTATTCCCACGCATAATAGAGAGATATTGT

GATCGTATACAAATGTAAGCAAGGTTTGAAATGATTATGTTTTAGTCAAA

TATTATATCCGTTTGAGCTTCTTGCGGTCAATTTGCAGTCTACAAATTAT

TTGTAATTATGTTCCGGCCCCCCGACCATCCGCTCAAGAAAAATCGTCCC

GCGGCTGAATCTAGTTGATGATCCCTGCTG

>rnd-4_family-1129#Unknown ( Recon Family Size = 22 Final Multiple Alignment Size = 18 )

GAGAGAGGGGCTGTCAACAGTTTAATNAAATATGTTTNGTTGCGAAAACA

TGTTACTATCGATGTTCCNAACAGATTTCACTTGGTTTCCCAAATTAAGC

ACTGGGTAGCTGCAGGAACAGGGTTGGAGAGCCCATGGCATACAGAGTTT

GGGCGGAATATCACCTGTCGCGCAGCGAGAGGCCGCATGCTCCTCAAACA

GTGGTTGATGCATGGAGTGAAATAACCAGAGTAGCCTACCCAGCATGANT

GAAAAACNAACCGGCGGGAAAGCGGCCTCCATTCGCTATTCGAGTGCATA

CGGCTGACGTCTTTTTTCCCCTGCCCCTGTCCATTTGATAATGGGCCATT

CTAAATCGAAACAAATTTNACATATTAGTAAAGACGAGATTAAATTGAGA

ATAGTCTGATGGGTGAAAATATGATCACTTGATGAGAGAACAGCGTGCAG

CCTGAGGCAAGGA

>rnd-4_family-2424#Unknown ( Recon Family Size = 23 Final Multiple Alignment Size = 18 )

TGGGTAGGTTACTTTCTAAATGTAATCCGTTACAGTTACTAGTTACCTGT

CCAAAATTGTAATCAGTAACGTAACTTTTGGATTACCCAAACTCAGTAAC

GTAATCTGATTACGTTCAGTTACTTTTAGATTACTTTCCCCTTAAGAGGC

ATTAGAAGAAGACAAAAATGTATGTTACCAATTGAACGACATCTATTGCA

GGATAAATCAATGTTAAAGTTTACATAGCTGGCCATATATGGATGTTANA

TTTTACTTTATGGGTTGGTTATGTAGGCTTCTTCTAACCCATCGCTTTCT

ACTACATATAATAATACGATTAAATTATATCTTTACATTAAAAACCGAAG

TCTATCAGAATTCCAGTCATTCCAATAAATGTTATACCCCTTGATCTTCA

AGAATAGGACTTGGAAATATGGAAGTATAGATTAGCCAAATTGTTTTACC

TGAGCATAACCCCATAACTAAGGACTTATTGGCCAGCCCTACTCTGTTGT

TTATGATTTTGTTGTCATGGAGGACTGATTGGGCTCATTGATTCGAGTTG

AAAAATAAATGCCGCGCTCATGGAATGGCATGCTTTGAGCACTACTGAAA

AGTGCTATTTACATGTGGAAAATGAATNCNATATGCTGCATTTNCTATAG

GCCTATTGTTTATTAGACACTCTAATGTACTTGTCCTCTTGCTCAGTTGT

GTACTGGGACCTCCCACTCC

>rnd-4_family-1003#Unknown ( Recon Family Size = 23 Final Multiple Alignment Size = 18 )

GAATTCATGTAAACATTACAGTTTTGAAAACATAGCTTGTCCAAAAAAAG

TGGTCTCTTGGCACAACTTACCCCGGGTATGGGGTAAGTTGAGCCGCGGG

ACAGGGTAAGTTAAGCCGCCTACANATTTCTGTACTGAATNAAATATTAC

CACTACCTTTTTAAAACCATGTCTATCTTTATTTCCCAAACACAATTCAA

CACAATCACAATTCAAGTCACAATCGCTTTTNGTCTTTTAATAATTTTAA

GCATCTTTTAACACAGGCTTAACACCTAACAAACACTTTGTACTTTAAAC

ACTTTTAACATAGGCCAGGCCCTGTCGTTACCTCAT

>rnd-4_family-36#Unknown ( Recon Family Size = 18 Final Multiple Alignment Size = 17 )

AAATATGTTTTATTGTCACATACACCGGATAGGTGCAGTGAAATGTGTTG

TTTTACAGGGTCAGCCGTAGTAGTACGGCGCCCCTGGAGCAAATT

>rnd-4_family-110#Unknown ( Recon Family Size = 18 Final Multiple Alignment Size = 17 )

AGAGATAAATAGAGATCGCTACTTGGAAATAACGAATGTTTTAGCATGGG

CAGCGCCATTGAGGACTTTCACCATTTTGAAGTAGTCAACTGGGTGGGAC

TTCCTATGGGTTAAGGAAGGATCACATAATTCCATCNAGGTCATCAGGAG

GGATCAGCCAATTAATTATACTCGTGAGNAAACATTCCATAACTGCAGGT

GGCAGTAAATCGCCAACCTTGNCTTTATACCTGTTCAAACAACACACTCC

AGGTGGCGGTATGCACCCTTTCAGTTTGTTTACCAACTCATAGAAGTAGT

AGAAGAAGATNGACTACTTCAAAATGGAGATG

>rnd-4_family-2013#Unknown ( Recon Family Size = 18 Final Multiple Alignment Size = 17 )

CTAGAACAGTGGTTCCCTACCAGGGGTACTACTGTAGGACCCCTGGGGGT

ACTTGGCCTGTCCACAGGGGGTACTTGAGAAGACTCATGAGACCATAGGC

TTACTGGTAAAATGCACATGAGGGGTACTTCAGGGGTACTCCGGGCAGAG

CAAAATTCAGTTGGTGGTACAGTAACCGAAAAAGGTTGGGAAAC

>rnd-4_family-864#Unknown ( Recon Family Size = 29 Final Multiple Alignment Size = 17 )

TGGAGGGTTTTACGCGCATCCAAATTTTTCACAGTAGCTGGACAAAGACC

CAATATAAATAGAAAGGGGAGAATGTCCGCTCACCGTTATACTGCTCCCA

AATAGGCCTATGTTTTATGAACATAATCGGAACCATCTTACAGATCAGAT

CGCATTCACACATCTCCAGAAGTTGCATTGCANGCGCGCCACGGACGTTC

TCACCATAAAACCGGGAAGGTGAATCGTTCGAATAAGTTTGGTTGTAATA

AAATTAAACGAAAAACAATGTAAAATGTAATATCATAAAACCTCAGATAA

NAAAGACACGCATTGCTGTTGAACCAGCCTTCGTTATAGTAGGCCTAAGG

GAGGGCTTGGGTTTTGAACATACGAAACGGAAAACAAGCCATTTTTACAG

GTTACAGATAACTTCTAAATACGAGGTTCACCGGTATTCACCGAAGTTCT

CATCTCTCGTTTCATGATGGGCATGGACACGTAGCCTACATCATGGAGGG

GNTTTTACGCACGTCCAAAACGGGAGCTGGCTAAAATAATGTGAGCCGCC

TACAATGCATAGATAAAAAGGGGATGTCCGCTCACTGTTTTGCTGCTCCC

AAACTTGATNTTTTAAT

>rnd-4_family-215#Unknown ( Recon Family Size = 19 Final Multiple Alignment Size = 17 )

GACAGGGGTATGGTAGGGTGCGGGTACAGCGGAGGTAAGCCCAGGCACTG

AGTGACGATAAGAGAGGTTNCGTCTCTGGACGCGCCGGTTATAGCCGGGT

GAGGTCACCGCATGTGTGGGAGGTGGGACAAAGGAGGTATCNGAGGCATG

TTGAGCGGGACTAGGGGCTCCACAGTAAANTAAAACAATGATAACTAACC

TGAACAACAGTATACAAGGCATATTGACATTTGAGAGAGACATACAGCGA

GGCATAAAGTAATCGCAGGTGTTGATTGGGAGAGCTAGCTAAAACAACAG

GTGAGACAACAACAGCTAATCAGCTAACACAACAAGAGCAGGTAAAA

>rnd-4_family-1837#Unknown ( Recon Family Size = 23 Final Multiple Alignment Size = 17 )

CCTTGTTCCGGCCAAACGCTACGCCACGCCCACGGACGTTAGTTTCTTCT

CCGCAATGAGTCTGGATCTGAGTACCTCCCCGACNCTTCGCCGAATGCGA

ACACATTCGGGGCCGCCTGATTGGTCCAGAAACCGATGGGTTGGGCCAGA

GCCAGAACACACGTGGGTAAAGCGGCGGTTTGAAAATNCGTCATTGGCTT

TGATACTCTGATTGGTTAGAGACGATCCAATCGCTGATGACTTTGTTTTG

TACAACGCCCCTCGTTTCGACGTCACCACAAACGACTTCAACGATGGCAG

TCTCAGACTGAAGTG

>rnd-4_family-2670#Unknown ( Recon Family Size = 22 Final Multiple Alignment Size = 17 )

ATTGTTCACACCGCAGGCCTTAATGCTCAAATCAGTTTTGTTTTTTCAAT

CCGTTTTGGAATACTGACTGTCCAAACAGCAAGTTACAAGTGACCAAATC

GGATTTGTGTGTGTTCAGACAGCAGTCATTTGCTGACATGGCTACGCTAG

TTGTCATAGTAACGACGGGTGTGTGTGTGCAGTGGTGTAGGCTGATTGGT

GGTGGTGCTCGTGCTTCCTATCACTCAGAAGTTATGTAGCAAGCTAAGGT

GACAACAATGCCTGCCATGGACGTTTCCCAGTTGCTTTGAATGTTCAAAA

TCATAGTGTAAGAACACTTTTAAAGCCTCAAAGGATAAGATGATGCAACT

TTCAAAACGAGTCAAATTTTTGGCTAGCCACAGCAGTCAACTAGCAAGCT

AGGTAGCTGTTTAGCTTTCTAGCACATTCACTNATGTGTTTGTAAACAAT

TA

>rnd-4_family-904#Unknown ( Recon Family Size = 19 Final Multiple Alignment Size = 17 )

GTCTCCTTTTCACCTCATTTAACACCTGATTTACTTAACAAAAGGCACAT

CTCGATAGGTGGTCCAGGTAAGTCATGACATAGCACAAGTGGGTGGGGTG

GGCCTTTCCTCTTGTGTTCTCTATTGTTCCTCAAGCAAGGGGGGAGGCCG

ATGACATCACGGATTNCCATCAGACCAACTCCTTGAATGCCCTACTCCTT

GAA

>rnd-4_family-1863#DNA/TcMar-Tc1 ( Recon Family Size = 18 Final Multiple Alignment Size = 17 )

AAANGGTTGTCTAGCAACGATCAACAACTTAAATTTGAGCTTGAANAATT

AAAAAAATAATGATGGCAAATATTGTACAATCCAGGTGTGCAAAGCTCTT

AGAGACTTACCCAGAAAGACACATTTGTAATNGCTGNNAAANGTGATTC

>rnd-4_family-164#LINE/Jockey ( Recon Family Size = 18 Final Multiple Alignment Size = 17 )

TCATGATCAATGATGTTTACTCTCAGGTACGGCCGGATATTGGGAGGTCG

TTATTTGCAGATGATGGGGCCTTATGGAAGAGGGGAAGAAATGTGCCATA

CAGGAAGCAATTGATGAGGTAGAGCGGCGGGCATTAATGTGGGGATTCAG

GTTCTCTGTAGAGAAAACTCAGACAGTGTTCTTTACCAGGAGGAAGGTGG

GAGATGAGGTATGCTTGAGGTTATATGGGAGAAACTTGGAGAGGGTGGGG

GCCTTCAGGTTCCTTGGGGTATACTTTGACACTAGACTGACCTGGGCAGA

ACACATCGAGAGAGTGGTGGGAAAGTGTAAGAAGGTGCTAAATGTGATGC

GCTGTCTGACGGGGAAGGAGTGGACTATGGCAGTATAGCGTATGGTTCGG

CAGCCCGGACCTCATTGGAAAGGCTAGATGTCATACAGGGGCAAGGACTC

AGAATATGTAGTGGGGCGTTTCGGACCTCCCCGGTGGCNGCGCTACAGGT

GGAGATGGGGGATATGCCGTTGCAGATTAGGAGACAGCAGCTGGCAATNC

ATTATTGGGTCAACCTAAAGGGACATGGGGTNTCTCATCCCGCGAAAGGG

ATTTTACAGGCATGCTGGGAACATGAGCGAAGACAGAAC

>rnd-4_family-1149#Unknown ( Recon Family Size = 21 Final Multiple Alignment Size = 17 )

TTTCTATGAAATATGACCTATAATTAATTACAATATGAGTGAAATAGTTT

TCATTCCAAAATNTTGTAATTAAGTATGTTAAAAATCAGCTTTTCTGTGT

TGGAATGGTGTGGGCGTACCCCAACAACAGAATGGTGTGGGCGTATACCG

GTCATTAAAAATATTAACGCAAGTAGACCGCTGATTGGCCAGCTCATCCT

CCTCGGCCGGATGACGTCATCCTCTTCGAGGAAATGGCAAGCATTTTTTA

AACGGTCTGTTTGAGATACAATTTGAGGTGTTTTTTTNCCCTCNAATTTA

TGCTTTGGCCACAAATACGAGTATAGGACGAGTCAACAACATTATTTGGG

TATGAGTTAACAGAATATTAACTTTTAAAAGTGAGATTTTCACTGGACAG

TTACTTT

>rnd-4_family-274#Unknown ( Recon Family Size = 18 Final Multiple Alignment Size = 17 )

TAACCTGCCCAGGGACTGCGGTTGAAAATTAGCCGGCTGGCTAAAACCGG

CACTTTTACTGAAACGTTGATTAATGTGCACTGTCCCTGTAAAAATAAAA

AATAA

>rnd-4_family-1116#Unknown ( Recon Family Size = 27 Final Multiple Alignment Size = 17 )

AATAAAACAATGGCCAGTTTGGGTNGCAGTTGCACGTTTTTTTTATAAAA

TAAATAGCCTATGTCTGGACCGCGAATTTCTTTTGTTTTTTCCATATTGC

CCGTTGATCTGATTGAGTNCGACACCCCTGGGCTAGCGTATCTATTTATG

TAGCTAGCTATTTATTTAGCAAGCTAAAAACACGGAGCCTAACGTTAGCT

AGCTGCTGGGAGGATGTCATGTCATTGGAGGAGTGGGTGAGTGACCGACT

TTTTCCCCTCATATCGTTTTTCGGTGGCTACAGCTAGAGATGCAGGTGTC

ATTTGGTTAGCTAGCAAGAAATGTGAATCGCTTTGCTAGCTAGCTTGCAT

GAACGACTGTTATCCACAGTTAGCACATCTCTTAGTTGTCAATCAAATGT

ATTTGNCGTCGATCAAATGGGCGGGCAGGCAAGTTCCCATTCAGTTGTCA

AAACGTGGGTTGGGACAAGCATGCATTGGCAGGCAAGCTGCAGAAGGACG

AGCAGGCTACTATTCCCCATCGTTTATCAGTGCAATTTTGACTGCCAACT

AGC

>rnd-4_family-4305#DNA/hAT ( Recon Family Size = 18 Final Multiple Alignment Size = 17 )

AGGGATGGGCAACTCCAGTCCTCGGGGGCCGGATTGGTGTCACACTTTTT

CCCCATCCCTAGCAAACACAGCTGATTAAACTAATTGCATTCTAAACTGA

AGATCATGATTAGNTGATTATTGGAGTCAGGTGTGTTAGCTGGGGCTGGG

GCAAAAGTGTGACACCAATCAGGCCCTCGAGGACTGGAGTTGC

>rnd-4_family-458#Unknown ( Recon Family Size = 17 Final Multiple Alignment Size = 16 )

TTACAGTGAGCTCCAAAAGTATTGGGACAGTGACAA

>rnd-4_family-9#Unknown ( Recon Family Size = 19 Final Multiple Alignment Size = 16 )

AGGCAGGTGAGTCCAGCTGTGTATTGACAGGGGTCGTGTTTTATATTGAA

AGTCCACAGTGTTTTTTTGCTTCAATAGAGTGATCAGGGACGTGCAACTA

TAGTTTTCCTTCACAGAAAATACATTAGTGCAACACATTCGGCAGAAAAT

AGCTTNATTTTCATCAGATGACGACAGAAGTGCAACGCTATTTGGCTAGC

AGCCACACGAGTAAATTAGCGTACAATGAAAGGTCGTTTTTTGCAGAAAC

GATGCATGGCCATCATATTTCTAATGTTATCATATAAATAATGCAGAATA

GGCCCGCTACACCGAGAAAAGTAGCTANACGTCAGTCAGAGCGCTGTC

>rnd-4_family-952#Unknown ( Recon Family Size = 21 Final Multiple Alignment Size = 16 )

GAAGTGAAAATCTGAGGCCCGCACCCGACCCTAACCCGCTAATATAGAAA

ATGCGCTGTAGGCTACAGTCAAAGACCGCGGAACGATTTTTTGACAGGGG

GTGCAGGATTTATTTTGCCCGATTTAGTTTCTGCTTATAATTTCCAACAT

TTTGGTAGGCTATTTGTTAGTCAACTTGTCTATAATTAGATACGTGCAGC

TTCTCTTCTGTCATTACNTGTTGCCCTAGAAGACTAAATAAACCCTTGCT

CACCAGAATAATGTCATACATCGATAGAATGAATGCTTCAATCTAGTTGA

CGTCGGTAAAGTTGATCTG

>rnd-4_family-590#Unknown ( Recon Family Size = 17 Final Multiple Alignment Size = 16 )

CATTATTCAATGTTTTATTATTTTCACTGCATCGGGGATAGCGTACACAG

ACGTTTCGGCTTTACAGCCTTCTTCAGTGCGTAGGTACAATTTTCTTTAA

TTCAAAACAGCATTTGTAAAGAACNACAGATGAGCAGCAGGTGCTTCTAA

TCAGGGTTGNCACTCATCTGCCAATCAGGGATGTGGCTTTTCTGGTCTAC

CTGTATACAAATTAGTCACAAAGAAATACAGAATTATAGGGTATGGGAGC

GGGCACGAAGGGCAACTCACGAATCAACCGCCCCAGGTGTCCGCTCACCT

AAATACATCATATCAATTCATGAGATAGCNCACCACAAAG

>rnd-4_family-920#Unknown ( Recon Family Size = 17 Final Multiple Alignment Size = 16 )

CTTTTCACACTACTGAGCCAAGCCGAGCCGAGCCGTACTGCGCTGGCCTG

GTTACGCATCCACCATAGTTGCTGGAACCGTGCTGGAAAGGACAATGTGA

NAAGAAAATATCCGAGCCAGCACAGTACGGTTCGGGTCGGCACGATAGTG

CGAAAAGGGT

>rnd-4_family-1221#Unknown ( Recon Family Size = 27 Final Multiple Alignment Size = 16 )

ACTATATAGAAATAATGACACTCAAATTGAAAATCATAAAACGAAATAAG

GATTTNTATCAGCCTAATCGAGGTGTAGATTACATCTCACATTCCAGTGT

TCGAACTTGTAAACAAGGCTGCATGGGATTTCTCTTAATGCGACTCCGTG

CAGCCAATGGCAATGTCCGCTTTAGGTATAATGCCGGGAGCCGCTCGTGG

ATTTGACAGCTCTAACGCAGTTCCACCTCCGACACCGCCAAAACAACCGC

TATGCGGATGTCGGCTAAAGCGGATCTGATTGAAT

>rnd-4_family-342#Unknown ( Recon Family Size = 17 Final Multiple Alignment Size = 16 )

ACAACTTCAATTAGCACTGACGTGTCAAGTGAGAGGTGTCAAAGCCCTTT

AGCCCAACAATGGCAGGAGAGTCGCACAGTCTACCCCAACCAAATGGAGA

GGCCTTAGAAGCTNCCTCCCGCTGTTCAGAGGTAATTAAAATACAGTACC

CTNTGTATGTAAAGAATGATAAGGCAAGAAAAGACCACAAAATGAAGCTC

CTTATGGAAAATCAAGAGACACTTTTTGCTGACTATTACAAAAATGGGAA

CATCAGCAACCTCATCTTCCACACAAACCATCCCCTGGCATGGCACAGTG

CTATATTAGCACACTACCCCTCTGTTAAGAGAGGGGGGGTTAACGAGGGG

TGGAAACTCAGAATACTCGACAACGAGGACTCTGAGTTAGGTAATATAAA

TATCTATAAATCCGGAACAGTAATGGTACAGGGCAACCNCAAACAGTTTC

AGCTGGACTTTCACCTAATCAAAGAATTAGCCCAGCAGGAGAAGCTCTCC

CTTGATAAAGATACCCCCACCCCGAGCGGGTCAGACCAGACCCCTTCATT

ACGTAACCCCACAGACGAGCAACCCCCAGCGGAGAGTCAACCTCCCAGCA

CAGAGTACTACTCCCTCATTGAAATGAA

>rnd-4_family-254#Unknown ( Recon Family Size = 18 Final Multiple Alignment Size = 16 )

ACTGCTCTTCATTGGGACCTCTTCATCTGTAGACAGGCTTTCACAGCTCT

CTNTATCTGAGGACAGAAAACCTCACAAGAAACAGACTTCATTATANTCA

AATTACACCGCACTGAATATTATGTTACTATTATCTCCGTCCTCACTTCT

AGGGACAGGAACTACACAAAAGTACCTGCCCTATCCAGAATAGCCTACTC

TCTCACTGACAGTTGGGGCTGCTATCCTTTCACCCTCCTCCATGGCTGTT

AGCTAGCTACCTACAAATGCATTTGGAGTTTGTTTTTTACAGTGATAAAT

ACATCAAGATAGCTAGCTAATATGAAGTTAGGTAGCTGGTGATATTTAAT

TAACTTAGCTAGCTATCTATACAGTATGTGAAGTTGGCTAGATAGCTCAT

TTAGCAGCTCATTTGAGTTAGCCTGCACTGTAGCTAGTTAGCTAATAATA

CATTCGGTTTTTTTTCCATTTTCGAAATGTATTTACTTACTTACTTGAAT

AGGTTCTCCCAGCCATGTGGACGATTGGAAACAGGGCAGCAAA

>rnd-4_family-568#Unknown ( Recon Family Size = 22 Final Multiple Alignment Size = 16 )

TAAGGCAAAGGGTTCTACCTAGAACCTTTAACATCCTAAGAACATGTTTT

GGGGAAGAAACATTTCTTTGATGATGCAGGAAGGATTCTTTGGTGANGCA

AGAAGGGTTCTTTGGAAGGCATAGAANGGCTCTACATAGAACCATATCNT

ATTTCCCAGCATGCTCTATTGCAGGTAGATTTTCGGAATTGTTTGTTTTA

ATGTAATATCTGTGTTTTTGCATGTACATTGATTGGTTGATTCATTTTAT

GCTACACAAAGATAAATAACAAAGTTTTCTAAACTAATCCAATATGTTAG

TTGGATTTATTGCACCCGTTACTGAGATGGTTGTTCCAGTTTAGATGCTT

AGGGTAGTCTCATCTTATCAATCTTCCCCACCCTGGCAGTCATTCTGAAT

GCAGGTTGCGGGTGATTAAAAATTCCGACTGTTGGATATCCTAACTCTGN

CTGGATTCCAATAGGAATTACACATCACTTTGCAAGCCAGCATAATGTGA

CTTGCAGGCCCGATGTGGCCTGTAAACCAGGAGTTTCCTAACGCCACTGT

GTTAGGGTATAACTAGGCCGTCAAAGAAAGGCCTTATGATATATGTAATG

TATTGTCATAAATAATTAAATTTTAAAGGGT

>rnd-4_family-6#Unknown ( Recon Family Size = 29 Final Multiple Alignment Size = 16 )

AGTCAAGATTCAGGCATTCATGACTAGGGTNAAGTGACTCTTGATATGAA

GAGTTTATTTCCAAAACGCTATATCCATTTTCAGAAATGTTGGTAAATTA

GCTTTTGTTGTTTAAAGAACTTATGAAAGAGATTGTGTTTTTTCACTATC

TANTCATGGCATGGGGTTGTTCAATGACAGACATGTATTTGTTTGAAATG

TATCACTTGATGGTTTCATTTCAGAGAGACAAATAATCGTGTTTTTTTGC

AAAACGCTATATATCCGCCTCACTCTCNGAAAAATAAGTAGTACTGCTAG

GTTTTACACAGTCAAATGTAACAAATAACTACATTTTTNATAAAGAACTG

TCTTTACAAATGATACGTTTGTGACATCAATATAATTTTGTTTAAATGAA

TCAATACAGTAATATGAGATCTGTATGTAAAACGTTTACATTTG

>rnd-4_family-1158#LTR/Gypsy ( Recon Family Size = 18 Final Multiple Alignment Size = 16 )

ACAGAAGAATCCGACCAAGAATGGACCCAGCGACTTCGGATCCTCTCCAC

TCAGCCGTCGAGATCCAGGGAGCGATGCTAGGCAGACACGAGCAGGAATT

GTCTGCTGCTCGACATGCCGTTGAGACCCTGGCCGCCCAAGTCTCCAACC

TCACAGAACAGGTTCACCATCTCCGCCTCGATCCACCGGCCACTTCCAGG

GCTTTCGAATCTCCGGAGCCCAGAATCAATAACCCGCCGTGTTACTCTGG

GGAGCCCACTGAATGCCGCTCGTTCCTCACCCAGTGTGATATTGTGTTTT

CTCTCCAGCCCAACACTTACTCCAGGAGCACAGCTCGTATCGCCTACGTC

ATATCTCTCCTTACTGGACGGGCTCGTGAGTGGGGCACGGCAATCTGGGA

GGCGAGGGCTGAGTGTACTAACCAGTATCAGGACTTTAAGGAGGAGATGA

TACGGGTTTTTGATCGTTCTGTTTTTGGGGAG

>rnd-4_family-656#Unknown ( Recon Family Size = 18 Final Multiple Alignment Size = 16 )

GGGGTACTCAACTCTTACCCTACGAGGTCCGGAGCCTGCTGGTTTTCTGT

TCTACCTGATAATTAATTGCACCCACCTGGTGTCCCAGGTCTAAATCAGT

CCCTGATTAGAGGGGAACAATGAAAATGCAGTGGAACTGGCTTCGAGGTC

CAGAGTTGAGA

>rnd-4_family-719#Unknown ( Recon Family Size = 29 Final Multiple Alignment Size = 16 )

TGTTTTCAAGTACGGGTGACAAANCATGCATTCTGATTCTTGCATAGATT

GTAATGGACACATATTATGTGTGTAAAATACTTTTTTGAAGGTTGTACTG

ATTATGATGCGCTAAGCTAATTCCAGCTATGTTGTGGAGCCATGTTTGTT

GACATTATACAATGCATTCTGGGTGTCACGTAAACGTCTGTCGGACCAAA

GATGTTATAACAAAACGAGGTGAAGGAATGGTTCACTCGTTCTTTCGAGT

AAACTTCCGGAAGTGAATGGTGGGATGTGAACGATGGTAGACGACACACC

CCCTTCAACATGCATACTANAAACAGACCAAACTCATCTTGTCTCCTCTT

ATTACCTTTGGTTTCCGCGAAAAAACAAACAGCGGCCCATCGTCGGATTA

CTTTCGATTTCTAGAAAGTGTTTTACTCAATTATTTTGATCAATGGTG

>rnd-4_family-668#Unknown ( Recon Family Size = 17 Final Multiple Alignment Size = 16 )

GTAATAACACGGTAACAGAATGACGCTGAGACTCAGATTTTTCACTTTAA

AATGTATGTCAAACAAAAACCAATGATTGCAAAGTTAAACAAACCATACA

ACTCTATGCACAAGGACTACTTTNAACAATTTCCAAAAAACACATTTACT

CGAAGAACAGTGCAGATGCAAAGTTTGGTAACAGAATGACGGTTCGTGCC

GTTCGCGCCGTCATTCTGTTACCAAACTTTGCATCTGCACTGTTCTTCAA

TGTATTTATTTGTATGTCAAAGTAGTCCTTGTGCATAGAGTTGTATGGTT

TGTTAACTTTGCAATCATTGTTTTTTNTTTGACATACATTTCGAAGTGAA

ACATCTGTCTCAGCGTCACCGTTACCGTGGAATTGCC

>rnd-4_family-3666#Unknown ( Recon Family Size = 19 Final Multiple Alignment Size = 16 )

TTCTATAAACCAATTTCTGTGTTCATGCAAGTGACTGATTGTAGAAATCN

TCACTATCAGTATCTGCAATTTGGCAGTACGCCCAAACNTTGTTTTGAGA

ACGAACAAGGGATCTCTCAGCTTAGAGAGGAGAAGCCTCGTGAGGTATTG

GTCTGTCACATGTATGAACCAGTATTGGTCGGTCACATGAATGAANCAAA

ACGGTAATGATTAATTAATTATGCTAAATCATGCAAATATAACTTGTCTG

TGTATAGCCGTATATAAGACAACTGNCGGGACTGCCCGAGGAGAGCTCCT

GACCGACATGTGTACTATGGTGCATTGAGTTTGTTGGAACCTCTCCAGCT

CGCTGATAATAAACAATGATTCATTTAAGATTGACTTCGAGTGTCCCTGT

GTGAGAATTTCCACGAC

>rnd-4_family-191#Unknown ( Recon Family Size = 17 Final Multiple Alignment Size = 15 )

GTTATTGTTTCTCTGTAATACTACTAGCCACCTAGCAATTTTATGAAGTT

GGCTTTAGCTAGCCCAGATAGGTTCCCAATCTCCCAACCTCATAACTAGC

TACCAAGAAGCCATTTCAGGCTATCAATCAAGTTAGACGAGCTAGCTTGT

CTAACTATCTTAGCTGGCATGCCTGCTGGCAAGGTTGGTAGACTTTAGAA

AAGCAAGCAATTACTAAATGTACTGAATAAGACTCACATTCCTTTCAATA

TTTTACCCAGATTTTAGCAGAGATGCAAAGAAGCATATTNATTCTTTAAA

AAGCTCAAGAGATATGCAGA

>rnd-4_family-474#DNA/Tc1 ( Recon Family Size = 19 Final Multiple Alignment Size = 15 )

AAATTTTTCTTCCACTTTGACATCAGAGTATTTTGTGTAGATCGTTGACA

AAAAATTACAATTAAATCCATTTTAATCCCACTTTGTAACACAAAACATT

TTGGGAAAAGTAAAGGGGNGTGAATACCTTCTGACGGCACT

>rnd-4_family-252#Unknown ( Recon Family Size = 16 Final Multiple Alignment Size = 15 )

TAATGGAAGCCTCTCCAACATAATCCAGGTAGAATCAGGAATTCCCCAGG

GCAGCTGTCTAGGCCCCTTACTTTTTTCAATCTTTACT

>rnd-4_family-1400#DNA/Harbinger ( Recon Family Size = 16 Final Multiple Alignment Size = 15 )

AAGTGACGCGGGAGGGCAAAAAAGCATCTAACGACGCACTTAGCTGTTCT

ACGAGCGGTCTGAGGCAGGCGGTAGATAACGAGGTTTTNAAGTGCAACTT

TAGTAACGATGNTTTTGGGAAACAGCTCGGAGATTTAACGACGCTCCTAC

GAAGGTTCTAACGATGAACTTAGCCTTAAGATGCTTTTGGGAAACCGGGC

CCTA

>rnd-4_family-3716#Unknown ( Recon Family Size = 22 Final Multiple Alignment Size = 15 )

CAGCCTAGGAAAGAACCAGGGTCTGTAGTGACGCCTCTAGCACCGCAGTG

CCTTAGACCGCTGCGCCACTCGGGAGGCCA

>rnd-4_family-362#Unknown ( Recon Family Size = 17 Final Multiple Alignment Size = 15 )

CGACCCATCAAGTTAGCCAGGTGTGTCTGACGGTGATTACGGCCATCTGT

TGTATAATAATGCTAAAATATTTGTATCTGGAATTTGTCTTTCAGCATCA

GTAGAACACGCCTGACTCTCCTCCACCAGTCATACAAGGAGAATGGTCTA

ATGCCTCCCATCAAAAAGAGAGCTGGCCCAAGCAAGCACAGGTTACACCT

GAAGCATGAGGACTTGCANCGACAACTACGCAGAGGATAATGCAATAGTA

TTACCAGGACGCCACCCAGGACACAAACACTTTGGTGGAAAGCTGCTGCC

ATCCCATGTGACAAAAGCAGCAGTGTGGCGTCTCTACAAGGAATCGATGA

CAACACTTGGTATGTAACATAAACACGTTTTGATTATTTAATTGACCTCC

GACACGATTGGCACNACTTTTATTGATCTCACATTATTTCCGNATTTTCC

GCCTTCAGNAATCTGTGGAGATTCCTGAAGCACTAAGCCCAGGACAGACC

TGTGCCGGCAGTGC

>rnd-4_family-100#Unknown ( Recon Family Size = 20 Final Multiple Alignment Size = 15 )

TTAAGTAAGCAGANAGGGTAGTTAACCTATGGTTGAACCGACTCAACTTA

GCTCTGGGATGTTTTAGATAAGGCAGCGAGTGCGTTCTTAGGTTTTCCGT

TANCTGGAACTGTCNGCTAAGTGGTGATNGACTATGGTGAGGGNTCAGGA

GTTAATCCAGNTATATTGTGTNACGTGTGAGTGCGCTAGTGAGTTGGAAT

GAACTTTTGACCTCGCTTTGTCCCGGTCGAGAGGAGGGGATTCATGTTAA

AGCAACGAAATGACGTCATGTTTTGTATATAAACCGTTGCNCGTGGTNAC

GTGGCAGCGCNCTCCGAGAATAAATGCTGTTGCCTATTNTTGANAGGCTG

GTCTCCGTCTATTTTATGCAAACAAGAATCTTACAAATTCTCATAAAATA

GACTAAGTGNTTTCAATTAATGAAAACACATTGGAATAATTAAATTACAG

TAACA

>rnd-4_family-632#Unknown ( Recon Family Size = 16 Final Multiple Alignment Size = 15 )

CCGCGATACCTTCGTCGCGTCCACCCGGTCTTCCATGTCTCCTGTGTCAA

GCCCGTTCTTCGCGCCCCCGCTCGTCTTCCCCCCCCCCCCC

>rnd-4_family-1039#LTR/Gypsy ( Recon Family Size = 18 Final Multiple Alignment Size = 15 )

TCGCCANCGGACCCCGGCTCCCCGGTATCGTCTCGGGCAGAAGGTATGGC

TNTCCACNCGGGNCCNCCCCCTCCGGGTGGAGTCCCGCAAACTGTCCCCC

CGGTTNATCGNCCCTTTTCCCATCTCCAAGATCATTAGCCCCTCTGCTGT

TCGTCTTCTGTTGCCCCGTACCCTCCGTATACANCCCACCTTTCATGTGT

CCAGAATTAAACCCGTGTCTCACAGCCCTTTGTCTTCCGTTTCCAGNCCC

ACCCCTCCTCCCCGTGTCGTCGATGGCCANCCGGCGTACACGGCGAGACG

CCTCCTGAGGGTTCGACCACGGGGCAGGGGTTTCCAGTACCTGGTTGACT

GGGAGGGTTATGGCCCGGAGGAGAGGTGCTGGGTCCCCGCTAGAGACATC

CTGGACCCGGCCCTCATCGCCGACTTCCACCGCCGGCACCCCGGTCAACC

AGGTATGCGCCCGGGTAGGACGCCAGGTGGCGCCCCTGGGGGGGT

>rnd-4_family-1652#Unknown ( Recon Family Size = 20 Final Multiple Alignment Size = 15 )

TTGCATACGTTCATTTTCAGTCCTGTGGTCTTACCTANGCGAATATCGTC

ATTAAAATGTAATATTAAAATACAATATGTATTTCATGAGGNCTTATTTG

AGTTGAGGGCACGCACAAAAAAATACTTGGTTGTTTGGNTGANCCAACGG

CNGGGTTGCAGGTGTTNGGTCACTTGGTTGGGTTGTTTCCNNNAAAAACT

AAGACCAAGGCTGGNTCACTNGCGCTNGNTTATTGAGATATGACCCAGCG

GGTCAGATCANAAGACTGGAGGCGTGGCTTAGTAGGGGCGTGGCTTTCAG

ATAGTTATTTTTGGCCACCCGTGAGAGTAAATGTCATTCCCGTTCATCTG

TTCTGTTGNATTGTCATCNCATNTTAAGGTTGAACACCGACACTTTTGTA

GCGTTAATGAGGCTTAAAGAAATGTGATTTCCCCAATGGGATAGTTACCA

CTTTGTTGCGATATGTAAATAATAATGTTAATNTTTTTGTATTANAATAT

GTAATGTGCAANAATATTNAAGAAATGTTACATTGCAGTGTACAAACTTA

ACATGATNAACAGGAATNACATTTACTCTCACGGGTGGCCAAAAATAACT

ATCTAAAAGCCACGCCCCTAATAAGCCACGCCTCCTGAAAATAACCTATG

GATTAC

>rnd-4_family-660#Unknown ( Recon Family Size = 16 Final Multiple Alignment Size = 15 )

ATTTCCATCGAAAAGGACCCATGAGCACCAACATGTGAAGTTCATAGGAG

CATGTCAAATTGGGTGTCAAATGAAAGCTAAGAGTCTATATTTTTGGGA

>rnd-4_family-3088#Unknown ( Recon Family Size = 16 Final Multiple Alignment Size = 15 )

AATGGCCTGGAAAATATGTTTCCTGCACAATTTTTCCCCACATAAAGCTC

TTTTGTGGAATAATTCAGACATAACTGTAAGGAATAAGTCATTGTTCTAC

CCCAGCTGGCATGAGAGGAATATTGACTTTGTTCTTGATGTTTTCGACAA

CAAGGGTAATATTCTCACATATGAACAATTTATAACATTGAAAGAGTTTC

CAATACCTTTCAGAGAGTTTATTTCTGTGATCAAAGCCGTTCCCAGTGGT

CTAACTACACTAATGAAAACTCATCTTAGCTTTGGNAATGATCACAAAGT

TTATCCAGAACTCAGATTGGAAGGCGTGGGCTTACTTGAGAAATCTTGTT

GTAATAAATATATAAGACAAATTCTTCATTCACGAAACCAACTTACGCCG

AGAGGAAAGTTTTTCTGGAACATGCTTATTCCTGACATTGTCTGGAAAAA

TGCATGGTTAAGGCCTTACAAATATTGTATACCAAACAAAGTTAAGGAAG

TGCACTTCAAAATTTTACATAAGATATATCCATGTAATTCTACGATGTCC

AAATTTGTGGCTATTGATGATATCTGCGT

>rnd-4_family-2170#DNA/TcMar-Tc1 ( Recon Family Size = 19 Final Multiple Alignment Size = 15 )

TGTTCGAGGCATGGTTCACATCAGGCAATGCTTCTTGTGAATAGNAAACT

CAAATTTTGGGAGTGTTTTTTATAGGGCAAGGCAGCTCTAACCATTGATT

GGACTCCAGGTTAGCTGACTCCTGACTCCAATTAGCTTTTGGAGAAGTCA

TTAGCCTAGGGGTTCACATACTTTTTCCAACCTACACTGTGAATGTTTAA

ATGATGTATTCAATATAGACAAGAAAAATACAATAATTTGTGTGTTATTA

GTTTAAGCACACTATGTTTGTCTATTGTTGTGACTTAGATGAAGATCAGA

TCAAATTTGATGACCAATTTATGCAGAAATCCAGGTAATTCCAAAGGGTT

CACATACTTTTTCTTGCCAC

>rnd-4_family-1351#Unknown ( Recon Family Size = 16 Final Multiple Alignment Size = 15 )

ACCAGCCAACAGATAGAACTTTAATGGTAGAAGGCTGATGTGGCCTCTAG

AAAACTGATTCAGGGTCAGTATTGTTTTCAGGTTCAGCTTCTCTGCCCTT

ATAGGAAAACAGATCTTTCAAAATACAGAAATCATTCCCGTATGATGCGT

TGTGCAAGATGTGGTGCGAAACGCAGAATAAAATCACGCAAAATGCATCA

TACAGGCTGTATTTCTGTATTTTGAAAGTTACNTATCTTGAAAACTTGAT

TGCTGACANGCAAAACGTTTTGGGACTATATCAACAATGGACTAATGAAA

CAAATACCAAAATATAGTTTTTGGGTGGAGTTTTCCTTTA

>rnd-4_family-356#Unknown ( Recon Family Size = 16 Final Multiple Alignment Size = 15 )

CTCTCANTCCTCCCTGGCTTTTGGGAGGGAGGGTGGACAATGAGCTTGTC

GTAGCGGATGTAAGCAATGTCCCCACGCTCTCTGGCAGCTTTCATAGCTG

GGATAAGTTATTTCCNCCTCTGGCGCACAGCTTCAGGGTAGTCCTCGTTG

AGGAAGATGTNGGTTCCTCTCAAGTTCTTGGCTCTCTCCAGAACAGCCAT

CTTGTCCTTGAACCTNAGGAACTTGACCACTATCGGCCTGGGTCTGTCAC

CTGGGCCGGTGACGGGTTTTCCAGACCTGTGNGCGCGCTCCACCTCAATC

TTCCTGTGATCCATCTGCAGCTTTTCGGTGAAATCTTTTCTCACTTTGTC

CTCGGACTCCGTCCAGGTCTCATGTGGAGACTCCGGTATNCCGTCCACAA

CAACGTTATTCCTCCTGGATTGTCCCTCTAGATAGTCTGCTTTCCCAGTC

ATCT

>rnd-4_family-1404#Unknown ( Recon Family Size = 17 Final Multiple Alignment Size = 15 )

TTTTAAAGGTAGACTCAGCGATATGACGTAGATGCAGAAAGTAAACAGCA

TAGCGGGTCAATTTCCGCAACAACTAAGAGCGTTGAAGCGCGAGGCTCAA

CTTCTCCGCTGTTTTGGTNCCCTGGCTACCACGCTGTGAACGGCGTGAAG

CGAACCCGTGCACATGCGCAGATACTGTGTGAGAGCGAAG

>rnd-4_family-1912#Unknown ( Recon Family Size = 17 Final Multiple Alignment Size = 15 )

GGCGGCAGGAATGCGTAATAGGGTTTTTTATTNNACCCAAATTACGGCGC

GCCGTGTAAAGGCACGGGGACGAAGACCAAACAAACACGTANACAAAACA

CAGGGTTGAAACCCAAACAAAAGAGCGAGGAGTACCTCGAATAAATACAC

ACGCGCGCAATGATTAACACACGGGACGAGACCCGTAATCATCTGCGCAA

TCCACAAGGGCACGAAAGCCAAAACACACAGCACAGGTACTCACACGCAC

CAACGGACATTGTAACAATAATCGACAAGACCATGGAAACCAAAGGGCAC

ACTTATACAATTACTAATCAGCGGGAATAGGGGACAGGTGTGCGTAATGA

CAGTTCCGG

>rnd-4_family-693#Unknown ( Recon Family Size = 16 Final Multiple Alignment Size = 15 )

TAAAGGACGTCACGCTGCTTGCTTAGCGAGTACCGCTTCCTCCCGATTCG

GCCCATACCTGAGGCTCGAACCCGGGACCTCTGCCTTGCTAGCACACGTG

ACCGCCCTCCNGAAGCGTCTTACCAGTCGGCGCCACGCGAAAAGCTAGCT

ATTCGCTGGCGCAAGTGGNGACACTTCAGGCTGAGGAGTAAGTTTCACAC

ATCCCCATGTGCTACAC

>rnd-5_family-38#Unknown ( Recon Family Size = 291 Final Multiple Alignment Size = 215 )

CCCCCTCGGGTTGTGCCGTGGCGGAGATCTTTGTGGGCTATACTCGGCCT

TGTCTCAGGATGGTAAGTTGGTGGTTGAAGATATCCCTCTAGTGGTGTGG

GGGCTGTGCTTTGGCAAAGTGGGTGGGGTTATATCCTGCCTGTTTGGCCC

TGTCCGGGGGTATCGTCGGACGGGGCCACAGTGTCTCCCGACCCCTCCTG

TCTCAGCCTCCAGTATTTATGCTGCAGTAGTTTATGTGTCGGGGGGCTAG

GGTCAGTCTGTTATATCTGGAGTACTTCTCCTGTCCTATCCGGTGTCCTG

TGTGAATTTAAGTATGCTCTCTCTAATTCTCTCTTTCTCTCTTTCTCTCT

CTCGGAGGACCTGAGCCCTAGGACCATGCCTCAGGACTACCTGGCATGAT

GACTCCTTGCTGTCCCCAGTCCA

>rnd-5_family-4#Unknown ( Recon Family Size = 330 Final Multiple Alignment Size = 171 )

TCATTAGTCGCATCATGCAGCCTTACAATGTATTAAAAATCAAAACATAT

AGCCCAACGTTTGTATCACAACTAAAGTTGCACAAATAACTCTAAATTAA

GCATATAGGAGTACCTGTTTCTTTGTTAACCGCTCAACACAGAATAGCCG

CATGTGCGTTTGGAGAAAATATCCTTTCTATTTTATTCAGCTNTGTTCAA

TTGTATTCTTCATACTATAAAATAATGCCACGGAATTCTAAGCAAATCTT

GTCTGCTAAATGAACTAGTGTAGCCCACAGCCATATGGCATAGCCAGATC

AGGACCTAACATAAGGACAACTCAGAGTATGCTATTCTGTTCTTCTGAAA

TAGACTACATTTTCTTCATATCATGTTTCTTTAGACCTGTCTAAAATAAA

TAATGGATTTATTGTGATGGTGTAGGCTATATTACATGGATTTATTAGAC

TTTTTAAATGTAGATGTTCCAAAGGTCTGCATCAGCGGCTTGTGTGGAAG

CCAGGAGATGCTAAACGTGTTTATGTTAATTAACGGTCAATTACCGTGAG

ACCGGCAGTTATTTGCTTGACAATCACCGGC

>rnd-5_family-155#Unknown ( Recon Family Size = 211 Final Multiple Alignment Size = 170 )

CTCGCCTTCTGGATGATAGCGGGGTGAACAGGCAGTGGCTCGGGTGGTTG

NTGTCCTTGATGATCTTTTTGGCCTTCCTGTGACATCGGGTGCTGTAGGT

GTCCTGGAGGGCAGGTAGTTTGCCCCCGGTGATGCGTTGTGCAGACCGCA

CCACCCTCT

>rnd-5_family-146#Unknown ( Recon Family Size = 214 Final Multiple Alignment Size = 169 )

CCCGTCAGGAAGTCCAGGATCCAGTTGCAGAGGGAGGTGTTCAGTCCCAG

A

>rnd-5_family-1038#Unknown ( Recon Family Size = 172 Final Multiple Alignment Size = 162 )

TTATTAGCGGCTTGTGTCTTTTTTAATATCGAGGAATATTTCACTTTCTC

TGGTCATAGGAGTAACAACATGAATTGGTGCATGAGGCAGAAATAATGCA

GTGCGACTTGAGTTTCGCCATCAGCTGGAAGACTGTGTCCCCTTTTCTCA

GCGGAGGGAGGGAGAGCGGAGGGACGGTGAGTCGGGTGAGAGGCAGCCTC

ACCGCTGCTCCCTCCCTCCCCTCAGACTGACCATCAGATGCAGGCCATCA

GTCCAGTATAAAAAGCNAATTATTATGCTCACTCAGCTGTGCCTCACAAG

TAATACAACAAATGATCTATTACCAGTGTGATCATATACCTAAAATTTTG

AAATATAATTTCAAAATGGTCTGAGAAGAACAACATTGGCAGGGCAATTC

AAGCATAGCCAATATGCGGTGATAATGTATTGGGCCTATAGCCTACTGCA

CAAACCTCATTGCTACAGAACTGTTTTTAATNGGTTAATGTTGCATAGGC

TTACGTTTTTTTTAAGTCATGTAATAAAAACTCAGAAAGTGATCTTGACT

CAGAAAAGGTTGGTGACC

>rnd-5_family-495#Simple_repeat ( Recon Family Size = 153 Final Multiple Alignment Size = 137 )

TGGACTACTGTCCAGTCGTGTGGTCAGGTGCCGCAAAGAAGGACCTAGNN

AAGCTGCAGTTGGCCCAGAACAGAGCGGCACGNCTNGCCCTTAAATGTAC

ACGGAGAGCTAACATNAATAATATGCATGTCAGTCTCTCCTGGCTNAAAG

TNGAGGAGAGATTGACTGCATCACTNCTTGTCTTTGTG

>rnd-5_family-151#Unknown ( Recon Family Size = 150 Final Multiple Alignment Size = 126 )

TTGGTATTTTATTAGGATCCCCATTAGCTGTTGCGAAAGCAGCAGCTACT

CTTCCTGGGGTCCACACGAAACATGAAACATAATACAGAAGACATAATAC

AGAACATCAATAGACAAGAACAGCTCAAGGACAGAACTACATACATTTTT

AAAAAGGCACACGTAGCCTACATATCAATGCATACACACAAACTATCTAG

GTCAAATAGGGGAGAGGCGTTG

>rnd-5_family-14#Unknown ( Recon Family Size = 218 Final Multiple Alignment Size = 115 )

ACTCATACAAAAGTTNTACTTAAACCCGAAATGGTTCTCNAGTAGATTAN

TAAGAAAGGCTCATCCNTTGTTCAAAAATGGCCTTTTTGCCTTTATNCAG

ATTGCAACGTCTCATTTTCGATTAATTGAAAATTANACTTTNTTCAAAGT

ATCGCTCTTTTTCAAACAAGCATTGCAGAGCTGGTTACAATTTCAGTTTN

ATCCNCCAGAAAAGATAGAACATTTATGGGAAAGATGTTTGAAAAGGGTA

TTTTGTTCTTAAATGATATCGTAAATNGGAATGGTGGAGTTATGTCACAC

ATGCAGCTATCGGAAATGTATGGGAATGTCTGCTCAATCCAAAATTACAA

CCAACTGATTGCAGCATTACCGCAAAAATGGAGGAGGCAAGTGGAAGCGG

GAGGAGGTAGGGAACTGGTCTGTCTGCCCNATATAAAGGATCAAAACTGG

CGGAGAAATAAAAATGGCATAAATAGGAAAGTATACCAGTTTCATTTGAG

GACCAAAATGTTGACAGCTGTGCCATACAGATTGCAAAATAGTTGGGAAG

AGATTTTCGATGTACCGATTCCATGGT

>rnd-5_family-1162#Unknown ( Recon Family Size = 123 Final Multiple Alignment Size = 115 )

ATGTATTCATGTGATATTTGAGTGACTNAAACATTACAACAANATCTATG

GGCTAAAAAACNAAATAAAAAAACGTTAGCTGACATGGGCTAGTTGATCT

GGACATTTCTGACAAGTTATAAATAGCTCTCTAAGGTATGCAATGACTGA

CATGACAAGAGGAACTGCTGATGCACNACCAAATTTCGAAATTGCACCTT

GTGCATTCTACTATTCCAACTCTCAACAGTAAGTTGAGACCCCGACTGAG

TTC

>rnd-5_family-60#LINE/L2 ( Recon Family Size = 137 Final Multiple Alignment Size = 109 )

CACCATTAGCTCTGACAAATTGAAAACTCTAGTCATTGGCGACTCCATTA

CCCGCAGTATTAGACTTAAAACGAATCATCCAGCGATCATACACTGTTTA

CCGGGGGGCAGGGCTACCGACGTTAAGGCTAATCTGAAGATGGTGCTGGC

TAAAGCTAAAACTGGCGAGTGTAGAGAGTATAGAGATATTGTTATCCACG

TCGGCACCAACGATGTTAGGATGAAACAGTCAGAGATCACCAAGCGCAAC

ATAGCTTCAGCGTGTAAATCAGCTAGAAAGATGTGTCGGCATCGAGTAAT

TGTCTCTGGCCCCCTCCCAGTTAGGGGGAGTGATGAGCTCTACAGCAGAG

TCTCACAACTCAATCGCTGGTTGAAAACTGTTTTCTGCCCCTCCCAAAAG

ATAGAATTTGTAGATAATTGGCCCTCTTTCTGGGACTCACCCACAAACAG

GACCAAGCCTGGCCTGCTGAGGAGTGACGGACTCCATCCTAGCTGGAGGG

GTGCTCTCATCTTATCTACCAACATAGACAGGGCTCTAACTCCTCTAGCT

CCACAATGAAATAGGGTGCAGGCCAGGCAGCAGGCTGTTAGCCAGCCTGC

CAGCNTAGTGGAGTCTGCCACTAGCACAGTCAGTGTAGTCAGCTCAGCTA

TCCCCATTG

>rnd-5_family-257#Unknown ( Recon Family Size = 113 Final Multiple Alignment Size = 108 )

CAACTTGCAGACTTGTTTACGTGTTGCTGTGCGTTTTGTTGCCAACCTTA

CTTTGCTACCTGACAACTTTACGGTTTTTACTTTTTAATTACCGTTTATA

TTTTTAGTTTTTCCCTCACTCAACTTTTTTTCACTCAACTTTTTCACTCC

GGACGCTTTATCTGGACACGGTTCGTCAGGACCTCCAACAGCCGAAGCTA

AGTAGTAACATTAACATGATGCCTTCTAATTGCAGTCGCTGTACTCATAA

TATACAGGAGAACGATCGCCTTACGGCGAGGATAGCTGTGCTACAAGCCC

AGCTTCAGACGCAATCGTTAGGCAAGGGTAATTTCAGTGTAGGAAAGGAT

GAAACAGCGTCTGTGCCACCAGTAAGTACAGATAGTAATTAGTATAAATC

CCCTCGCACGGTCCCCGCAGCCGGACAACTTTCTCACGGTTTCTGGAGGG

AAATGCTGTAGGAACGCTCAACCGGTGTCGCTCATTCAGCCGACAGAAAC

TTTCAACCGGTTTTCCCCATTAAGCAGCGAGTCGGAGTCAGAGGCCGAGC

CTTCTCTNGTCTCTACTCCTCCCGTTACGGGGTCTGAGACG

>rnd-5_family-108#Unknown ( Recon Family Size = 275 Final Multiple Alignment Size = 104 )

ACCGGTCAGGCACCGTGTTATGCGGTGGNGCGCACGGTGTCTCCGGTGCG

CGTNCACAGCCCGGTGCGCTACGTNCCAGCTCCCCGCATTTGCCGGGCTA

GAGCGGGCATCCAGCCAGGACGGNTGGTGCCGGCTCNGCGCNCCAGGCCT

CCAGTGCGCCTCCNCGGCCCGGTNTATCCTGCGCCGGCTCCGCGCACCGT

GTCTCCGNCCTGTGCCAGCNCCCCGCATCGCCGGGCGAAAGTGGGCATCC

AGCCAGGACGGGTTGTGCCGGCTCCGCGCTCNAGACCTCCGGTGCGCCTC

CACGGCCCGGTGCGTCCNGTGCCGGCTCCACGCACCGGGCCTCCGGTGCG

CCTCCCCAGCCCGGTGCGTCCTGTGCCTGCTCCCCGCACCCGGCCTCCGG

TGCGTGTCCCCAGCCCGGTGCNNCCNGTGCCGGCNCCCGGACCCAGGCCT

CCGGTGCGCCTCCCCAGTCCGGCGACGGTCCNCGGCCCGGAGCCTCCGGC

GACGGTCCACGGTCCGGAGCCTCCGGCGACGGTCCCCGGTCCGGAGCCTC

CGGCGACGGTCCCCGGTCCGGGGCCTCCAAT

>rnd-5_family-479#DNA/Tc1 ( Recon Family Size = 104 Final Multiple Alignment Size = 97 )

AGCGTCATACCCAAGAAGACTCGAGGCTGTAATCGCTGCCAAAGGTGCTT

CAACAAAGTACTGAGTAAAGGGTCTGAATACTTATGTAAATGTGATATTT

CAGTTTTTATTTTGTAATACATTTGCAAANATTTCTAAAAACCTGTTTTC

GCTTTGTCATTATGGGGTATTGTGTGTAGATTGATGAGGGAAAAAATTTT

TTAATCCATTTTAGAATAAGGCTGTAACGTAACAAAATGTGGAAAAAGTC

AAGGGGTCTGAATACTTTCCGAATGCACTGTATG

>rnd-5_family-346#DNA/TcMar-Tc1 ( Recon Family Size = 135 Final Multiple Alignment Size = 93 )

GTACAAACAATAGTACGCAAGTATAAACACCATGGGACCACGCAGCCGTC

ATACCGCTCAGGAAGGAGACGCGTTCTGTCTCCTAGAGATGAACGTACTT

TGGTGCGAAAAGTGCAAATCAATCCCAGAACAACAGCAAAGGACCTTGTG

AAGATGCTGGAGGAAACAGGTACAAAAGTATCTATATCCACAGTAAAACG

AGTCCTATATCGACATAACCTGAAAGGCCGCTCAGCAAGGAAGAAGCCAC

TGCTCCAAAACCGCCATAAAAAAGCCAGACTACGGTTTGCAACTGCACAT

GGGGACAAAGATCGTACTTTTTGGAGAAATGTCCTCTGGTCTGATGAAAC

AAAAATAGAACTGTTTGGCCATAATGACCATCGTTATGTTTGGAGGAAAA

AGGGGGAGGCTTGCAAGCCGAAGAACACCATCCCAACCGTGAAGCACGGG

GGTGGCAGCATCATGTTGTGGGGGTGCTTTGCTGCAGGAGGGACTGGTGC

ACTTCACAAAATAGATGGCATCATGAGGNAGGAAAATTACGTGGATATAN

TGAAGCAACATCTCAAGACATCAGTCAGGAAG

>rnd-5_family-421#LINE/Rex1 ( Recon Family Size = 112 Final Multiple Alignment Size = 86 )

CAGACGGCATCCCTAGCCGCGTCCTCAGAGCATGCGCAGACCAGCTGGCT

GGTGTGTTTACGGACATNTTCAACCTCTCCCTGTCCCAGTCTGTNGTCCC

CACATGCTTCAAGANGGCCACCATCGTTCCTGTGCCCAAGAAAGCA

>rnd-5_family-663#Unknown ( Recon Family Size = 136 Final Multiple Alignment Size = 84 )

ATCTGCATTGACCTTTTATTTATTTTTGCACTGNCTCTATGCACACTCAC

NGGGCCACACACACTCACACACTGCACTCCAACACACATAATTTGCTCAC

TCACACATAACACGCACATACATTCATACTGACTCTACACACACGCACAC

NCACTCACATACAAGCTGCTGCTACTCTGTTTATCATATATCCTGATGCC

TAGTCA

>rnd-5_family-1434#Unknown ( Recon Family Size = 88 Final Multiple Alignment Size = 79 )

AGACTATCATTTTACAGATGTATGTTGAAAACGTTTTTGGGAGATGCGAT

GGATCATTGGGGATCATTCAATATTCCCTTTCTTTTGTTGTTCAGTGAAA

TCATCCCATGTGAAGAGTCAACTCATTTAATTAAAGTTCAATTCGTAACT

AAATCGTTTTTAATATTTCTATTGGAAGGATTTAATCATTTGCAATTATG

TCTACTTATGATAAGGTAAAAGGTTTATGTTTCTGTCTCCATATGATATG

GTAAATATATCCAATGCAAAAAACATCTACATTTAAATGGTATTAATATT

AATTTGCATATATTTCCGTTAATTCCCATATATTCCCGTTAATTCCCACG

GAAAGTTTCCACCTCTGAATATTCCCCAAAATGTGC

>rnd-5_family-64#Unknown ( Recon Family Size = 115 Final Multiple Alignment Size = 79 )

AAGCTTTGTAAACAAAAAGGGAGTAATGAAGTGATCTACGGGACTTCAAN

GAGGACCAGCCGACCTTTTGATACAGGATGCAGTGAGTATTAAAACTGTC

ACCCGTGATAAAGCGAAGTGCGCTATGGTAAACGGCATCCAAAGGTTTAA

GAGTAGTGGCAGCTGCATTCTGATAAATGGTGTCACCATAGTCAAGAACC

GGTAGGAACGTTGACTGNACGATCTGCTTCCTGCTATTTAGCGAGAGGCA

NGACCTATTTCTATAAAAGAAGCCCACTTTAAATCTTAGCTTCTTAACTA

GCTCATCCGTATGTATATTTTTTACATTGAATCCAAATGCCCAGATATTT

GTANGCGGGGACCCGATCGATNGGAGNACCGTCCAATGAGTAAATATGTA

GTCCATCTGAAACATTTTTACGAGAATTAGAGAACAACATGTATTTAGTT

TTGCCCGCATTAAGTACNAGTTTTAAATCAACNAGGGCTTTCTGCAAGGC

AACGAAGTCGGATTGCAGCTCTGANACAGCCTGGTCAGCAGTCGGGGCAA

TAGCGTACATAACAGTATCATCCGCATACAGATTAATATTGATACAATTG

TTTA

>rnd-5_family-121#Unknown ( Recon Family Size = 83 Final Multiple Alignment Size = 77 )

GGGCGCCAGAGATCTAGATAACCAGAANATTTTTTTTTAACCTGACCCGA

CCATCCTCCTCCCGCTCCTGCTGGCCTTCGCAGATTCTGCCGTTACGCTC

CTGAAGTTGCCGGTAATAGGCTACACCAGGAGTCGGCAACCTTTCCCATT

TGGAGTGCCAATTTATCTTACCATTTCTACCGATCTGCGTGCCAGTTATG

ATTTTCATATGCACATTTTCGTGGAACAGTTTCATTTAATTTATAATAAC

GTCTTCGTATCTCAAAATCATTGTCATGTGGTTAATCAAAATTCTATCCA

AATCTAAATGAAAATTATACAAACCTAAAAGTAACTTCTATTGCCATTGC

CAACTATGTAAAAATAGCCTACATAAAGCCAACAAATAAAAACATTGCAG

CCTGCAGGTAGAAAATATCCTGATAAAAATAAATCACATTGGCTACGCAT

GGCCTGTCTGCAACGAACTTGAAACATTGTATCAACTATCAACTTGG

>rnd-5_family-223#DNA/TcMar-Tc1 ( Recon Family Size = 190 Final Multiple Alignment Size = 75 )

ACATCCCAAGGAGCACTGTGCAAGCGATAATNTTGAAATGGAAGGAGTAT

CAGACCACTGCAAATCTACCANTACCTGGCCGTCCCTCTAAACTTTCAGC

TCATACAAGGAGAAGACTGATCAGAGATGCAGCCAAGAGGCCCATGATCA

CTCTGGATGAACTGCAGAGATCTACAGCTGAGGTGGGAGACTCTGTCCAT

AGGACAACAATCAGTCGTATATTGCACAAATCTGGCCTTTATGGAAGAGT

GGCAAGAAGAAAGCCATTTCTTAAAGATATCCATAAAAAGTGTCGTTTAA

AGTTTGCCACAAGCCACCTGGGAGACACNCCAAACATGTGGAAGAAGGTG

CTCTGGTCAGATGAAACCAAAATTGAACTCTTTGGCAACAATGCAAAACG

TTATGTCTGGCGNAAAAGCAACACAGCTCATCACCCTGAACACACCATCC

CCACTGTGAAGCATGGTGGTGGCAGCATCATGNTTTGGGGATGTTTTTCA

GCGGCAGGGACTGGGAGACTGGTCAGGATCGANGGAAAGATGGATGGAGC

NAAGTACAGGGAGATTCTTGANGAAAACCTGNTCGAGNCCGCTNAGGACC

TCAGACTGGGGCGAAGGTTCGCCTTCCAACAGGACAACGACCCTAAGCAC

ACAGCCAAGACAACGCAGGAGTGGCT

>rnd-5_family-71#Unknown ( Recon Family Size = 79 Final Multiple Alignment Size = 74 )

CATTTGACACGTCAAATAACACAGTTCTATTATAGAATGTTGTGTGTTCT

GAATTTGCACGTGCAAGCCAAGCGCCACCACTACTATCNGTAAAAATAAA

NNNNNNNNNNNNNNNNNNNNNNNNNNNNNNNNNNNNNNNNNNNNNNNNNN

NNNNNNNNNNNAAACCAACAACCAGAAAGCATTATTTGTTCGACCGCAAC

TTCTGGGGTAGCTAGCTAGCACCGATACAACCAGCCTGAAAACAATGACC

AGTAGAAACTGCAGTCATTTTCATTATTCTTAGCAATGATTTAGGAATCC

TTGTAAGTATTAGCTAGGTNGCCACTTGTTGTTCGCCTATTGAAATTGAA

CTTCAGTTCATGAAAATAAATAGCTAGCCAGCTACTTAACCCTGTTGCCC

AAAGCTAACGTTATAAGCTAGCCAGCTAGCTTAACCCTGTTGCCCAAAGC

TAACGTTATAAGCAGCCAGCTAGCTTCATCTGGCTAGTGAGGCTCGACCG

GACCGGGTTATG

>rnd-5_family-1081#Unknown ( Recon Family Size = 84 Final Multiple Alignment Size = 68 )

GGTCTGCATATTATGCCCATAGAAACGCATTGGGCTTATTCTGGACAGAT

TTTGGCGAGTGAGCCCTCTCGCTTTCGCCTCTCCTCTCTGCTGAAACTAT

CTCACCGGAGAAAGCATCCGAGCGAGCGAAACGGCGCCCCTCTATATGTA

GCCCATGTATCTGATGCTGTCTGGACAGAAANAGTATGACATGCCATACT

CTTTTTGTCCAGACAGCATCAGATACATGGNCTACACATACTGAGACAGA

GGGGCGCTGTTTCGCTCGCTCGGATGCTTTATCTGAGATTGATGCGTCTT

TCTGTCGGCGCGCGTCTCGGTCAAATAAATGATCAATATTTCAATATTTT

ATTTGGACGGGCAAGGAGGTACGGTAGGGCGGGCC

>rnd-5_family-611#Unknown ( Recon Family Size = 100 Final Multiple Alignment Size = 67 )

TGGTGTGTGTAGCCCAATCACNAGGCATGCCTACAGTCGGGAACGCGCGG

CAAATCTGTCAGTGAACAGCACGCAGCCAAAACAGGCCTTTCGCAATATT

TCAAATACAATCGCGGGAAAAAAAACAGNTTGGAAAGCAAATGGCTACTG

CTGAAAAGAGAAGACTAATCTGTCTGTAGGCTACCAAATATGTTATCAAC

TTCCAAATAGGCCTATTGAGCGAACAGCATTGTTTTACAAAGTAAAACAA

GAGAGAGATAGGCTTTCGGCCATCGCCGGTGAGTGAGCTGCGCATCATTG

GGTGAGTCAGTGNNACTGGAAAGTTTTTTAGGACNATAATTTCCTCCTCA

TATCGTACAATATGTGTGTCTCCACACACCTAGGCCTAGGCTATTGATGG

ATTCAAGACAAGGTCGTTTTTATTGATCTCAGTTTGTCAGTGTCAAAGTA

GCCTGTCATTTCGATCATTTGTGNGGTATTAAAAAANATTCTGCTAANGT

CTCCAGTCGTNTAAAATGACGTAGAATTGCATGAAATGCGTAATTCTCGA

AAAACATCCTCTGCAA

>rnd-5_family-900#Unknown ( Recon Family Size = 84 Final Multiple Alignment Size = 67 )

AGTTTTAACAGTGGCCAAGTAGGCTACTGTGGCTATTTGATCATAATGTA

GGCCTACCAACAAAANCAATGGAGAAAATCCCATAACATTTTAACATGGA

AATAGCTTTGGTTTCTATCATTCAGCCTACAGTAGCAGCCAATGTGTGGT

GTTCAATGTAGGCCTACATTCCATGAGACTTTTAAAAAAGTTTTTACATT

ATGCAGGGCTTGACATTAATTCATGTTTNTCCACTTGTCCTTACACCATG

GGCCAAACGAGGTGACTGNAAATGTTGTGTTGTNTGATGCAAGAAACCAC

TTTACAAAATANAATGCATTATTATTACCATACAGAGAATCAGACAATGT

AGGCTACCCCTCTGCCTATTGGCTTATTTGCATATTCAAGCCTGTCTCAA

AATACAACACTGCCCCTTTAATTAAGACANAAGCTCTTTACCTGACTNGC

TTTTCAAAGACGGCTTGAAATGTAGCCTACACGTTTTGTGCTCTTGTAGG

AAGCAGTNACTCCCCATTGCTGACCTATACTTATAATAATAATTNTACTC

CGTTGCGCTCTGGCTCTGCCTACAACAAAATCACAGAC

>rnd-5_family-2491#LINE/L2 ( Recon Family Size = 74 Final Multiple Alignment Size = 66 )

GCCTCTAACCCTAGGAAACTNTTTTCCACCTTCTCCTCCCTCCTTAATCC

TCCACCCCCTCCTCCCTCTCTGCGGACGACTTTGTCAACCACTTTGAAAA

GAAGGTTGACGACATCCGCTCCTCATTCACTCAGCCTATTGAGTCCACTG

GTCCCACTCACACAGAACTACCCTACGCCTTGACCTCTTTCTCCCCTCTC

TCTCCAGATGAAATCCTGCGACTAGTGAGGTCCGGCCGCCCGACAACCTG

CCCGCTCGACCCCATCCCCTCCTCCCTTCTCCAGACCATCTCTGGAGACC

TTCTCCCATTCCTCACTTCCCTCATCAACTCATCCCTGACCACTGGCTGC

GTCCCCTCTGACTTCAAAATGGCCNGAGTCGCTCCCCTCCTCAAGAAACC

AACACTCGACCCCTCTGACGTCAAAAACTACAGACCGGTATCCCTTCTTT

CTTTTCTTTCCAAAACACTTGAGCGTGCCGTCTCTGACCAACTCTCTCGC

TATCTCTCTCAGAACGATCTTCTTGACCCTAACCAGTCAGGCTTCAAGAC

GGGTCACTCAACCGAGACTGCTCTTCTCTGTGTCACGGAGGCTCTCCGCA

CTGCCAAAGCTGACTCTCTCCTCTGTTCTCATCCTCCTAGATCTATCCGC

TGCCTTCGACACCGTGAACCA

>rnd-5_family-1463#LTR/Gypsy ( Recon Family Size = 69 Final Multiple Alignment Size = 64 )

AGCCCGGGAGCTACCNACGGATCTCGACTCCCTCATCGCCTTGACCATCC

GGATCGATGGGCGGCTACGGGAACGNAGGAGGGAGAGGAGGTCCGATTTC

GCTCGCNCGCCCAGGGATCCCACCTCGCCTCCGAGGNATCCCGGAAGTCC

CCGACGNCTNCGTTNCCGAGAGAACCCGAGGCTACCCGAGTTCCCCCGAG

AGTCGCCGAAGACTGCCGANTCGCCTCTTCCCGAGCCNATGCAACTAGGC

AGAGCTAGGCTGTCNCCAGCGGAACGNCNACGCAGGCTCGACACCAAGAG

TTGTCTGTATTGCGGGACTNCCGGTCATTTCCACTAAAAGACCAGGCTCA

CCGGTAGGAGCGAGTACTCTGGTGGGCCATACGGAGAACTTTTCCTCTCC

CCTTACTCGCACCCCTTTCCATGCCATCCTGCTGTGGGGNGACCAGTCCA

AATCTCTCCGGGTNCTCATCGACTCTGGGGCCGACGAGAGCTTTATGGAC

GCTACCCTGGCGTCCGAGCTGGGCATCCCCACTCAGCCCCTCTCCATTCC

CATGGACGTTAGAGCGCTGGACGGGCGCTCTATAGGCCGGGTCACCCACA

ATACCGCCCCCATCAACCTACGNGTGTCGGGGAACCACAGCGAGACGATC

CAGT

>rnd-5_family-2138#DNA/hAT ( Recon Family Size = 103 Final Multiple Alignment Size = 59 )

TCCCAACCCCGGTCCTCGAGTACCCCCAACAGTACACATTTTTGTTGTAG

CCCCGGACAAACACACCTGATTCAACTCGTCGANGGCTTGATGATTAGTC

GACGAGTTGAATCAGGTGTGTTTGTCCGGGGCTACAACAAAAATGTGTAC

TGTTGGGGGTACTCGAGGACCGGGGTTGGGAAACACT

>rnd-5_family-1292#Unknown ( Recon Family Size = 62 Final Multiple Alignment Size = 56 )

TAGTACATTTACCGTTAATTCCCATATTTCTAATCTACAATGTTTGTTTG

GTTACGGTAATTTCTGTTAATGCATTCAATATTACAGTCTTACCGTTCTC

ATTGTCGGAGTGGACACGTTGTTTGCAGAGCGCACAACCTANGCTACACT

TGTGAGAAACAAGTTTTGGTTTATTTCATTTACGAGTTTTGTCAATTTAT

TCATTGTCTTTTGTTTGGAGCGCTCCTGTCAATGTTGA

>rnd-5_family-1753#Unknown ( Recon Family Size = 57 Final Multiple Alignment Size = 55 )

TTGAATATATTTANTTCATTTCATTTGCGATTTTCATGAATAGGAAAAGT

TTCTAGGGGTATTTATGTCCGCTGCGTTATGCTAATTCGTTTGAGGCTAT

GATTACGCTCCCGGATNCGGGATTGCTNGTCGCAAGAG

>rnd-5_family-386#Unknown ( Recon Family Size = 64 Final Multiple Alignment Size = 55 )

TCAGGGATGGGCAACTTTGATGGGGTGGGGGCCACAAAAAATCTGAACTC

ATCATGAGGGGCCGCAGTGGCTCGCGGGTCTGCGTACCCACATCCATACC

CACACATGCAGTCAGAGCCGGCCCTAGCCTTTTGGGGGCCCTAAGCGAGA

TT

>rnd-5_family-2244#Unknown ( Recon Family Size = 57 Final Multiple Alignment Size = 53 )

TGCCTTTCCGGTAACTTTCCCAAAATTCCCAGGTTTTCCAGAAATCCCGG

TTGGAAGATTCCCGGAATCAGGAGGGAATAAGCAGGAAATCCGGGAATCC

TCCAACCGGGATTTCTGGAAAACCTGGGAATTTTGGGAAAGTTACCGGAA

TTTTGCA

>rnd-5_family-1853#Unknown ( Recon Family Size = 67 Final Multiple Alignment Size = 53 )

GAGAGCAATAGTAATGGCGTCTTTTTGTAGGCACTAACTCCGCCATGGTT

CGTTGGACAAAGCCTATGGGGAAATTAATGGGGTTTTTGGATAAACGCCG

AAAATAAGGTCTGTGGTAAACACAGGCTTAGGAGATCTTATACGTTTTGT

TCTATGAGATAATCTTCATCAGCTAACGTCACTTTTTGTGAATTTTGAAG

CATTTATGTAATCAAAAAAAGCACATAAAGGCTTCATAATTCATAAAGGT

CATGTTAACTGAGTATTATCTCATAGAACAAAACGTATAAGATCTCCTAA

GCCTGTGTTAACCTCAGACCTTATTTTCGGCGTTTATCCCAAAACCCTAT

TCTTTCCCCATTCATTATTCATTTTCCCCATAGGAATGGCTGAACGAACC

AGAGGTAACTNATTTCCGTTTTTAGGACTAC

>rnd-5_family-48#Unknown ( Recon Family Size = 61 Final Multiple Alignment Size = 52 )

CTGGGAATTGCCAGGGACCTCACGATACGATATTATCGCGATACTTAGGT

GCCGATACGATATGTATTGCGATTCTCGCGATTCTATACTGTGATTTTAT

TGCGATTCGATGTTCCAAACATATTGCTCACCATATGTCTGCTGCAGAGG

GACAAGAGAGAGCCATGAGAAAACGAGTTTTGATCAGTCATGGAAATAAA

AGTGCTGAAAACAAATTGGCTCCCTATTTAAAAAGAAGATGGAGAACAAG

CTATGAAGGAAAAATACTGGAGTTTTGGTGCAGGTACAGCCAACTAGCGC

AAAAATAATATTGCGATATTGTCAAAACGATACGATATATCGTCAAAAAT

AATATCCCGATATGTAACT

>rnd-5_family-1331#Unknown ( Recon Family Size = 79 Final Multiple Alignment Size = 52 )

GTGTGTGTGTCAGAGCCAAGATGATTTGGAGTAGTCCAACCTGAGACTAC

CGCACCAGGTATTCCTCTTCTGTTCCCATGCTGGAGACCAGGGCTTGATT

ACAACCTGTTACAATGGTGCCAACATTTTGTGGCTGATCTTTCAGCGGGG

GAGTGGGTGAATGTGTCAAAGACCTGACTGGGCCCAGCACCGCGCGGTAT

GGCTCGTTTTTGTTGTGTGCGTGTTTGTGTACTGAGGAACATGTAACTTG

GTTCCAGAAGAAAGCCACTTTCAATTTCTTTAGGTTGGGGGCTTTCATCA

AGGTAAGTGTTTCTTGGTGTAACGTCGTCCCCAGGCTATTGCGCACACAG

CACATATAAGCCTGGGATATCAACCATTCAAAGTTGGCCTTAGTGGGAGT

GACCATAGAATTCTATAGGAGTGACCTTACTGAGTAAAGTATTTGTCGTC

GTCACTAGTTACCACAGCCACAAAGTCATAATTATGCTAAACCCCGCCCA

TTTCTACAATTTATCTTCTTAAAATTTGATTTTAAACCTAACCTTAACTA

ATGAAGGCCAAGTTGGGCGGAAGTGTCTGCGAACGAGATTAGGTGTAATG

TGA

>rnd-5_family-674#Unknown ( Recon Family Size = 59 Final Multiple Alignment Size = 52 )

CATGAACGTGTACATGTCTAGACAATAGTGACCCATCCACTTAGCTAGAT

GTGGCTGGGGGTTGGTTATAGCATTTCTTTCACATGACCCATCAATTTAG

ACAAGTGTGTCTGGGTAAGCGTCATCTAATANTTATAAAATATTTTTATC

TGGACACTTTCTGTTTTTGATATTGCTACTATGCAAGTAACCATTTCGAT

GTACCGTTTACACCTTCTGTATCATGTGCATGTGACAAACTTTTATTTGN

TATAGTGTGTGTTTACCAGAGACGGTAATGTGAAGAACAACATGACCTGC

ACCAAAGTCAGATTAGGATATAGGCCAAGGACTAGATAAAGTGTATTTTT

ACCTGGAGTTTTTCCTTATTGTAGGCTACTACTTTCACCACTTTTAGTCT

TGAAATCTTTGGTTGTTTACTACACTACTCACTCTGTTTAGCACATGGCC

TCACATGTGAATCCTTAAAGAGATGGGTGGGGCTAAGG

>rnd-5_family-400#Unknown ( Recon Family Size = 56 Final Multiple Alignment Size = 51 )

TGTCAACACAACCAAAAATCAACATTTGAAGGAGATGTATTTTTGTGCCA

CTGACTTAGTCTNGCTTTAATTCCAGTTTGTCTACAAGTTAATAATTGAT

ATGTTGGATTCACGTCTCCATCTCAACCAAAAATCNAAGTTAAAGAATAG

GACTAAATCAAATCAAACTTTATTTAAAGTGCATTTAAAGTTTGATTTGA

TTTGATTTAGTCCTATTCTTTAACTTNGATTTTTGGTTGAGATGGAGACG

TGAATCCAACATATCAATTATTAATTTGTAGACAAACTGGAATTAAAGCC

AGACTAAGTCAGTGGCACAGATGGAACTATCCAAGCAGAAGATACATCTC

CTTCGGATGTTGCGTTGACACCGAACACGATTCATATNTCCAGTTCGTCT

CCATCTCAACCAACAAAGTTAAAGAATAGGACTAAATCAAATCAAACTTT

AAATGCGCTTNAAATATAGTTCGATTGATTTGGTTTAGTCTAGTATTCTT

AGTAGTTTAGAACTTTTTTGGTTGAGATGGAGACGTGAATCCAACATATN

AATTATTAACTTGAAGATTACATTTGA

>rnd-5_family-896#Unknown ( Recon Family Size = 60 Final Multiple Alignment Size = 50 )

AGGGTTGGGGAGTAACGGATTACATGTAATCCGTTACATGTAANGGATTA

CAAAAAAACGGTAACTGTAATCCGTTACGTTACCAGCAAAAATATTGTAA

TCAGATTACAGATACTTTTGAAAAACTAGATGATTACTTCTNGGATTACT

TTTAAATTCAGAAAGGATGTTTGCGAAAAAAAATACTTTGACACTTCTCT

GTTTTCTCAATGACATTCAAATCAGCATTGAAAAAAGGCGCAAGTTTAAG

TTTGTTCCACCTGAGCGAGTCTGACCACAAGTCAGAGAC

>rnd-5_family-1178#Unknown ( Recon Family Size = 50 Final Multiple Alignment Size = 49 )

AAAATGACGACATTTAGAAAAGTCCCAGAGNCGCAAGACTAGGTGCATTG

AAACCGGCTCGGCCCATAGAGACGGACCCCAACGTTTCTGTCCGATAGCT

CATTCAAGGACCCCGTAGCAAGGCATGGAAAAAAGTGGATTTTCAGCACC

AATTAAGGTCGTTGCTCGGGCACCGAATGACCTATCGAGCCGAAACTCGG

GATTCGGGGTCGCCTCANCTAGGCCTACACATAACGTCAGAACTGGACCC

GCAGCTAGAACGTAACTACGTGTTTTACGTTTTTATTATGTTTTAAACCG

AAGGCGCTGTGAATTNTGGGCCTGCTCTG

>rnd-5_family-284#LTR/Gypsy ( Recon Family Size = 67 Final Multiple Alignment Size = 49 )

GTGCGCCTTCCTCTCCCATCGTCTCAATCCCTGCTGAGAGGAACTACGAC

GTGGGNAACCGNGAACTCCTCGCGGTGAAGATGGCGTTGGAGGAGTGGAG

GCACTGGCTGGAGGGGGCGGAACATCCGTTCNTNGTGTGGACCGACCACA

AGAACCTGGAGTATATCCGGCACGCCAAGCGNCTGAACTCTAGGCAGGCT

AGGTGGGCCCTGTTNTTCACCCGGTTCAACTTCACCCTCTCCTACCGGCC

CGGGTCCAAGAATGTCAAGCCGGATGCGCTGTCCCGAGTCTACGCCCCTG

CNACTCGAGACACCGGACTGCCCGTCCTTCCCGCCGCTAAGATCGTGGCT

CCGACCTCGTGGCGAGTTGGGGACAGGTCCGTGAGGCGCAGCGTTCCCAG

CCGAACCCCAGAGGGGGCCCGGCTAACCGGATGTTCGTTCCCGACGCGGT

NAGGNCTCNGGTCCTGGAGTGGGCCCACTCCTCCAGGCTCGCCTGTCATC

CGGGCGCCTGTCGCACCCTGGCTTTCATCCAGCGTAAGTTCTGGTGGCC

>rnd-5_family-1145#Unknown ( Recon Family Size = 57 Final Multiple Alignment Size = 48 )

TTGCCCAGTTGTTTGTGTTAGAGATGCGAGAATACAAAAATCTAAGCCTC

GTGTCATCACAAAACTTCAGTGAACAGGCTTTTTTTACACGATCTTTATT

TTAGTGACCTTGATTGTATTTCAGCTATCCCTGAACCTGATTTAGCTTTG

AGCCTTTTTTGCAGATGTCTTCAATACTATCGTGGATAATCGTGCTCCCT

TCAAAAAATNAAGGGTAAAGATCGAATGCCTGGTACACTCCGGAATTATC

AGAAGTCATTCATAAAAGAGATGATGCTTGGGTCAAGGCCAGGAACACAG

GCTTAGGCCCGGACTGGCAAGCTTTTAAGCAACTGAGGAATCATTGTGTA

AGACAAATCAGAAAGGCTAAATCTGATTATTATGTAACCGCTCTTTCGGA

TTGTAATGGGAACCCGGCTAAATTCTGGAAAACTGTCAAATCCCTGAAGG

GTTCTACTTCCTCCTCTCTGCCACAACAAATTAATTCAGACACTGGCCTC

ATTACGGAAAAAAATGCCATCATTGATGCATTTAATCACCATTTTATTTC

AGCGGGCTCTCTCTTTGAAATAACTTCTAAGCCTATTCACAATGATATTG

GGCTGGATGCTGATAGG

>rnd-5_family-420#Unknown ( Recon Family Size = 72 Final Multiple Alignment Size = 48 )

AGACTTTGCCTTTGTTTTGGTTTGTTTGTCAATTAGGGTGTGCAGGGTGA

ATACGTGGTCTGTCGTACGGTAATTTGGTAAAAAGCCAATTTGACATTTG

CTCAGTACATTGTTTTCGCTGAGGAAATGTACGAGTCTGCTGTTAATGAT

AATGCAGAGGATTTTCCCAAGGTTGCTGTTGACGCATATCCCACGGTAGT

TATTGGGGTCAAATTTGTCTCCACTTTTGTGGATTGGGGTGATCAGTCCT

TGGTTCCAAATATTGGGGAAGATGCCAGAGCTGAGGATGATGTTAAAGAG

TTTAAGTATAGCCAATTGGAATTTGTGGTCTGTATATTTTATCATTTCAT

TTAGGATACCATCAACACCACAGGCCTTTTTGGGTTGGAGGGTTTGTATT

TTGTCCTGTAGTTCATTCAATGTAATTGGAGAATCCAGTGGGTTCTGGTA

GTCTTTAATAGTTGATTCTAAGATTTGTATTTGATCATGTATATGTTTTT

GCTGTTT

>rnd-5_family-1356#Unknown ( Recon Family Size = 48 Final Multiple Alignment Size = 47 )

ACTGACCTTTGTCGATAGCGCCTGATAAATTCAGGGCAGCAATGTTATTG

AGAGCAGTAGCAACACATTTGCAGTTCTCCATG

>rnd-5_family-1932#DNA/TcMar-Tc1 ( Recon Family Size = 48 Final Multiple Alignment Size = 46 )

ATACAGTTTATTAGGTACACCACCCCGTTCACGAAAATGGNTCGCTCCTA

CAGACAGTGAGTCACGTGGCCGTGGCTTGCTATATAAAGCAGGCAGACAG

GCATCGAGGCATTCAGTTACTGTTCGATTGAACGTTAGAATGGGCAAAAC

GAGTGACCTAAGCGACTTTGAGCGTGGTATGATCGTCGGTGCCAGGCGCC

GTGTTCCA

>rnd-5_family-119#Unknown ( Recon Family Size = 57 Final Multiple Alignment Size = 45 )

TTTTGCTCTGACATGCACTGTCAACTGTGGGACC

>rnd-5_family-163#buffer ( Recon Family Size = 58 Final Multiple Alignment Size = 45 )

CGCACCCATCTCTTTAAGGATTCACATGTGAGGCCATGTGCTAAACAGAG

TGAGTACGGTAGTGTACTAAACAACCAAAGATTTCAAGACTAAAGGCTGG

TTTATACTACGTCTATCGACATGTCTGTAGACAGTTGTCGCAGTGACATC

ATGAACATTCTATTGTCGTCCGACGTCAAACTTGTCGTTGTCGTTATGAA

AAGTATAAACAACGGACGCGGGCCAAAATAGCATAACTGGTGTATTTTAA

CACCAATAAACCCATCCGTTTAAAAATGAATTTAGTATATGTCAATCTAG

CAAACCAGGCAACTAAAAGCAACTTTCTAAACAATGTTTTGGTTCGTTTC

TAGCTCGTTAGCTAGCTAGCTAGCGTTAAATTAGCTGGCTAGCCAGTTCA

AATAATGACCGTATCATATAGCTGACAACGTCTTAACTTTAGCTCATTTG

TATTCATTATTACAGGAAAATAAACTCACAACAAGATCATTATTTACAAG

TTAATGGCGAGCTAATTACAGAAAATAGCTTACGGTTGTGAGTGCGACGA

AATAAAAGCAGGGCATTCTACCGGAGAATTTTAGAACTTGGACACTGTCT

CGTTGGCCTAACGTTATATCCTAATTTGAC

>rnd-5_family-150#Unknown ( Recon Family Size = 72 Final Multiple Alignment Size = 45 )

ACCTCTGCAAGCTCCTTGTAGTTGCCCTTGTTAGCGGAACTTTCCCTCTC

GTCGTGCCCTCTAAAAGCCAATTCTTGCATGCCCCAAAAAACGCAGGCAA

GGCCAGCAATACAGGCGGCTTGATGAAAGGCTCCCTGTTAACCAGCTATA

CTTTTGATACCAGTTGGTATTGAACGCCCTCACCTTTTCATCCTTCTTCA

TGAAACTGATCTCAGGCAGAGGTCGACCCTCGCTTTTAATTCGCACCTTT

TCCGTGTACCCCAGAGAATGAAAGGGGTTCTTCAACAAAAAGTCCACAAC

ATTTTCGGCAGCGTTGGCCATTCTACCATTCTGGCGGAGAAAACTGCACA

CTCGCGCTGGTAGCTAGCTAGCTACTGAAGTTAGCAAGGCAAATTTAAAT

AAATTGCACTAACTGGACACAAAAATAAAGTTCTAAAACCAAGAAAACAA

TTTATAATATACCTCCTCCGTCTCCTGTAATTTGAAAAAACTAATTTGAA

TTGAATAATATCCCAAAAATGCTCAACTATCACTCTTCACTCTTAATCTC

TATCCAACAATGTCACCAAGCTTCTCAACACATAGAACCCCCATAATGCT

CTGTGGCACAATCCCAATACAACT

>rnd-5_family-433#Simple_repeat ( Recon Family Size = 69 Final Multiple Alignment Size = 45 )

AGGGGCAGATTGTACAGAAGCTGGGACCAAGTCACTATTATTCGAGTCGC

AAGGTCCGAGTCTCGAGTCGAGTCCCAAGTAGAACGGGTCGAGTCTCGAG

TCAAGTCCAAGTCGTGCATTCTAAGAGCAAGTCGAGTCAAGTCTCAAGTC

AAGTAAAATAAATCTATACAGGCAATGACTTGTTCAACTACAAATCTTTG

TCTATTTTCATTATTTTGTCTACAACACATTTTGAATTTGTAATAATACA

TTTNACAACAAATTCCAAATGCAAGCTTCATAAGTAAATTATCAATTTTG

ACATACCAAAAGACCAGTAGGCCTATGCTAGTAATTGTTGCTTGATGCCC

CATTGCTGGTGAGCTTGCAAAGTAAACAGTTAATATTCTTGATCACCAAA

CATTTTTGGAGCTGCATATTTATTTATGGCAATCCTCTCTCCCTACAATT

TGACGTTTGCGCTGCACCCGATCCTATAGCTGTACAGCAAATCAGCAATC

TGTTCGCTAGCTCAGCGGTTTCAAACTTTTGACCCGCGACCCCCAAAAAG

GAGTGGT

>rnd-5_family-1795#Unknown ( Recon Family Size = 75 Final Multiple Alignment Size = 45 )

TCCAAAAAATGCAATATCTGTGCGCGCGGACTAGGCCTACTGATGTCCTG

TTCAGTTTGAGAAGGAGAGCGCGAAGATGAGAAGGAGGACCGGAGGTCAA

CTTTGATAGCTTGCTACTACTATAATTGATTTTATTAAAAACAATATGTT

TCTTGCCCATNATGAAGCTATTTATCAGAGTTATTGACCTCACAATAAGC

CAGATTCGAGTAACTTACATTGGTGCTGAAACTTGAAGCAGCAGCCGCGG

CACAATCAATCGGAAATGGACAGCTCATGGTGCTGAAAGTAGGCAAATTC

GAGTAGGCATAATTCATTTNAACCGTCTTCATTTTAATTNGACTTTACTA

ACAACAAGAGGGCTTTGTGTTGGAGCCTATTTCTTCCTATTTAAGAAATA

AGAGGTAGGCCTACCTGTTTGACAGACGAAATTAGGCTATAGGCTGCTA

>rnd-5_family-59#Unknown ( Recon Family Size = 101 Final Multiple Alignment Size = 45 )

TGTTCTGTGTTAGTTTACACAAGTATAGGCTGTTTCGGTTTTCGTTACGT

TTATTGTTTTGTAGTGTTTGTATTTAGATTCGTGTTTCGTTTATTNAANA

AACATGGATCGCAATCACCACGCCGCGTTTTGGTCCGCCTCTCCTTCGCA

CCAAGAAAACCGTTAC

>rnd-5_family-162#SINE ( Recon Family Size = 50 Final Multiple Alignment Size = 44 )

CTCAGCATATGTGGGAACTCCTTCAAGACTGTTGGAAAAGCATTCCAGGT

GAAGCTGGTTGAGAGAATGCCAAGAGTGTGCAAAGCTGTCATCAAGGCAA

AGGGTGGCTACTTTGAAGAATCTCAAATATAAAATATATTTTGATTTGTT

TAACACTTTTTTGGTTACTACATGATTCCATATGTGTTATTTCATAGTTT

TGATGTCTTCACTATTATTCTACAATGTAGAAAATAGTAAAAATAAANAA

AAACCCTTGAATGAGTAGGTGTGTCCAAACTTTTGACTGGTACT

>rnd-5_family-832#Unknown ( Recon Family Size = 53 Final Multiple Alignment Size = 44 )

GTTGTACAATTAAAGGTCAAATACACTGCATTTCAAACAGTCAGCAATAA

TCTAATGAATTCAGGGCTTGTGAAATTATACCTAGGCTAAATATAAGCCT

TCCACAACCATAAGACCCACTAATAATTTAATTATTTTATCAAAATAGTT

TAACCTGCTTTTTTGTTGCAATAATCACTGATCTGGCTTTCAAGTCTGTC

TATGAAAATGCCCTTTTTGTTACAAATTTAACCAGAACCATGCATAATGC

ACATTCACTAATAATGACTAACTTCTTGTAGCAGGCAT

>rnd-5_family-424#Unknown ( Recon Family Size = 58 Final Multiple Alignment Size = 44 )

AGCGTGTCAGCCTGACGGAACCGCCCCTTTCGACCAGAGTGCATAAAANG

ATGGGTNAAGAAATTAAACATCAGACCAGAGAGACGCGAGGCTGCAGCTG

CGCGTTTAAAGTGGTTCGAACTCTGAATCTCAACACGAGGTGGAGACGAT

AAACTCACCTCCCGGACAATCACTGGTACGGCTGATTAGCTGTCCTAAGT

CAAATATCTAGAAAAGCGAATTTAAGTGGGACCATCCTACTACTCTGCTC

AAACCATCGTATTACGTACTCTCATCACCCCACTGGGAACCGTCGACACG

GCTGGCTAGCCTATCTTCAAAGAAGCATCTTCNAGAGCGAATGGAAGNAG

AGTGAACTCTGTAGTTCTGTTCAGGACTACACGACGAGTCACCGGATACC

GGACGACTGCAGAGGAGAACAACAGTAGAAGACTTGTTGGAACCCTTTTG

GACAATCAGAGCCTTACAAGCGTGCCGCGGAAAGGCCCAACCCCCTTCCT

AAGGAGGCCCCGTTCCGACAGAGATAAACGGCGAACNAAGGCATTCACAC

GTAAATACATTCATGATTTCTTACTCCAAACGGGCGGCGGTTCGTGTGCA

AAGTATATGA

>rnd-5_family-27#SINE/Deu ( Recon Family Size = 52 Final Multiple Alignment Size = 43 )

AGCAGCATACCACCCTGCATCCCACTGCTGGCTTGCCTCTGAAGCTAAGC

AGGGTTGGTCCCTGGATGGGAGACC

>rnd-5_family-1282#Unknown ( Recon Family Size = 69 Final Multiple Alignment Size = 43 )

GATGAAGGGAGTTAGATATGCTNGGTTTTTTTTCTGTATATATGAATTAC

AAATCAGCCTTTACTTAAATCATGTTTCTAGTCTATTGCATAGTTACAAT

GTGTAATCATTGATGTCCCAGGAGCAGGAGTGGTAAACGCTGTTGAAGTG

TATAATTGTAATACATACATGCAGACACAGCATCATTCAAAGAATGGAGT

TTGATACACCTGGTATTGGATATGACTGAAAAAGAATGTCTCAGAGATTC

ATACCTGAACCGCACCTTTATTTGCCAAGGAAGAGTACATGATCGGTGAA

TATATTCAATGTACAGGCTACAATGTGGGGATCTCATGCGTGGTAATAAC

TACAGTACATNNNNNNNNNNNNNNNNNNNNNNNNNNNNNNNNNNNNNNNN

NNNNNNNNNNNNNNNNNNNNNNNNNNCAGAGTTGAAAGTCGATTTGACAC

AACAACTATGATAGCTGAANNNNNNNNNNNNNNNNNNNNNNNNNNNNNNN

NNNNNNNNNNNNNNNNNNNNNNNNNNNNNNNNNNNNTACTGTAGCTAGCT

ACACATCCATAGGCATACAGTTATTTTCATCCTCCACTACACAATAAGCA

AGTAAATAGTTAGCTACGTTACTTCACTGCATTGC

>rnd-5_family-63#Unknown ( Recon Family Size = 57 Final Multiple Alignment Size = 42 )

GGCAGCAGCGCAGTCATCAACTACATGCACCATTTCTTCACCAACTACGG

AGTTGGGAAAACACGTGTGGACCTGAATTGTGATAACTGCAGTGGCCAAA

ACAAGAACAAGTTTGTGCTCTGGTATTGTGCCTGGCGGACCATGCACAAG

CTCCACCACAGTCTGGACCTTCACTTCCTGATCACAGGCCACACCAAGTT

TGCCCCCGACTGGTGCTTCGGCCTCATCAAGCAGCGCTTCAGAAAGACCA

GAGTGAACACTTTGTCTGAGATTGCTGGTGTTGTGAAGGACAGCACTGTG

ACGGGGGTCAACATCCCACAGCTGGTTGGACTGGAGGATGGTACGGTGCT

GGTGGAAAGCTATGGCTGGCAACAACACCTGACTCCGTACTTCAGGCCGC

TGCCACAGATCAAGCAGTACCAGCACTTCAGGTGAATATCATTTTCATTG

TATGAGGTTATTCTTCTTATCTAAACTTGGGAGGTGAATTGATGTTGTGC

AAGGTTGTAATGTCCTCTACGTTTTTTTTG

>rnd-5_family-2878#Unknown ( Recon Family Size = 47 Final Multiple Alignment Size = 42 )

CCATATATTTATATGTACATAATTCTTATTCATCCCTTTACATTTGTGTG

TATAAGGTAGTTGTTGTGAAATTGTTAGATTACTTGTTAGATATTACTGC

ACTGTCGGAACTAGAAGCACAAGCATTTCGCTACACTCGCANTAACATCT

GCTAACCATGTGTATGTGACCAATAAAATTTGATTT

>rnd-5_family-220#Unknown ( Recon Family Size = 58 Final Multiple Alignment Size = 42 )

CCGTTAGCGATGATGCTAATGACAACCGTCTTCTGGTAGATGGAAAGGCT

TTCCCAAAAACCTTCTCAATTAAATGTTAACTACAAAGTAGCCTATGCCT

ACCTGGCAGAATGATATCATGATTATTTGCATCAATCCAGTGGCCATTTG

TTTTGCAAACTCTGCAATCACATGNGCGCTACAGAAACACCGCTAACCAA

ACAAGGCTCTTGCTGGTGCACGCTTGAATTAAAGGGTAACTACACCCAAA

AATCGACATTTCTTAGATTTTTCCCAGACCTCAAAAGTGGTCTCCTGATG

TGGTTTAAGCATTGTTGTGGACTTAGAACATCCAATTTTGTTGTTTTCTA

TTAAAAAGTGTGATTTTGAGAGCGAAAACTGGAGAAGCAGGGACAAACCG

TAAACCTGGAAAAACTAAACGGAGGAAATGGGGAATTAGGTTCAATAAAA

CGGATTAGAAAGAGATGTTATATATAGATAGAGATGTTATAGAGCCATAA

ATATTCTATACATTTTCTCTCATTACTGNATTATCTAGGGATAGTGTAGA

GTAACAGCTGTTTCCTGAAGTGACCTTTAACCTCCCTGCTCCTCAATACT

TCTTC

>rnd-5_family-1112#LINE/Jockey ( Recon Family Size = 44 Final Multiple Alignment Size = 41 )

GGTGGGTGGGTAATACCCAGGCGAGGGAGATGGNGTTGTATGGAAGGGAG

TTTAGTCCAACGGTAGCTATTCCTGTNAATCCACCATGGCTACTCCCGCC

TCCAGTAGTNGATCTAGAAGTGTTGGAGAGACTACGGAAAGATAGGGAGG

GTGTTGATCCATCTGATTTGTTTAAGAGACGTCTGGATACTGTGTATCAG

GATTTCGTGGCCATNTACACAGATGGTTCAAAGGATCCAAGGACAGGACG

TACTGGGTCAGCATTCGTAGTGCGGGAATGTGGGGTGGCAGTCAGGAAAC

GTATTACGGATCATCTGTCTGTNTATACGGCGGAGCTGATGGCCATACTG

TTGGCCTTGCAGTGGGTGGAGGAAGTNAAGCCAGACAGAGTAGTCATCTG

CTCTGATTCNTGTGCAGCGTTGATGAGTCTNCAGTCCTTTGTGTCACGGA

GCAGACANGACCTACTGTATGAGGTNCTGCAAACCCGTGGCAGGGTTAGA

CAGATGGGTATACNGGTGATATTCACTTGGGTNCCAGCTCACGTGGGAGT

GGAGGGGAACGAGGCGGTTGATGTACTGGCTAAACAAGCACTCAGAAGTG

GGGATGTTGANGTGGTAGTTTCAATGAGCAAGGCAGAGGCAG

>rnd-5_family-1455#Unknown ( Recon Family Size = 46 Final Multiple Alignment Size = 41 )

TGTGTGCAACAGGGAGTGGCAATTGAATGCAAGCTTCATTA

>rnd-5_family-1290#LINE/L1 ( Recon Family Size = 44 Final Multiple Alignment Size = 41 )

ATAAACAATCCCCAGACAGCACCTCCTGGAGTCANCTTAAAGCAGCCAAA

GCCAAACTGAATTTGGACTACACTCGGGAGATAAAAAAAAAGTTTTCTTT

ACTAAACAGAAATACCATGAGTATAGCAATAGGCCCAGTAGATTGCTTGC

TTACCAATTAAAAAAGGAGCAGTCAGAGCGTACAATCATGGCTATCCGAA

CAGCAGAGGACGAGGTCACATATGACCCAAAAAAGATCAATTTAACTTTT

CATGATTTTTACTGCAAACTATATACCTCTGAGAGAAAACACACGGAGGC

AGAACTCCACTCTTTCCTAGAGGGAATCTCGCTACCTAAACTATCAGAGA

CCGACCAAGAAGATCTCAACTCCCCCTTCACTCCTGAGGAGATCCTGGAG

GCAATTACCTCCATGCCACCTAATAAGTCCCCAGGCCCAGATGGATTCCC

CAGAGAGTTCTACAAAGCTTTTTGGCCCCAGCTCAGCCCTATCTTCATGC

CAATGCTGGAGGATTTTTGCAAAAACGGAGTTCTCCCAGACTCAATGCAC

ACAGCTCGCATTACAGTGTTGCTAAAAAAAGACAAGGACCCCCTATCCTG

CTCGTCCTTCCGGCCCA

>rnd-5_family-624#Unknown ( Recon Family Size = 41 Final Multiple Alignment Size = 40 )

GCATTGGTGGCAAATCTGATGGCCGAATGGTAAAGAACATCTAGCCGCTC

GAGAGCACCCTTACCTGCCGATCTATAAATTACGTCTCCGTAATCTAGCA

TGGGTAGGATGGTCATCTGAATCAGGGTTAGTTTGGCAGCTGGGGTGAAA

GAGGAGCGATTACGATAGAGTT

>rnd-5_family-1123#Unknown ( Recon Family Size = 55 Final Multiple Alignment Size = 40 )

TGGTCTGAAGATAGATTCCCCGGGTGGGGGTTTTATTCGGAACGGCAGAA

AAGGGCTGTCCCATGACGCCNGACCCTANCTGTGCTCGTGGGCGGTCCTC

TGATTTAGTTAAACTCCAAAGGGAATTGGAGTTTCCTTCATTAAACAGTC

CAAAATCACATTACACAATTTCACAAACAGTATCATCCTCACTCATTCAT

CTTATACAACAATTAGATGTAAACCTCATATCTGAGGCTATTGTATAAAC

AGCGTTATGGTAATGTGGCCGCACCGTCTCCCATGAGCTTCACAAAATTG

TACCAAACGGACCAGTTCGTAGCTGGATTCTTCACCGATCTTTTATACCT

TCTCCGGAACATAAATGTTGTTCGGACCTCAAGTTCTGTGAGGTGGAAGA

AATTCCTTTGTTCTCTCTATGAAAACTCTCTCTCTCTCTATACTGTGGCC

ATGAGGAGAGAGTCTCCTCCAGGAATTTACGACCTGAGGCCGCAGCAGCC

TGGTTGTAGGAGGCAGAGAGAGGGGGATGGNGCTCGCTGTACCCAAAGAG

GGCAAC

>rnd-5_family-489#Unknown ( Recon Family Size = 65 Final Multiple Alignment Size = 40 )

CCGAAGAGGTCCACCAGCCACTCCGGGGCTACAATACCTATTTTGCCAAT

TGGCCCGGACCCCTTTTACTGCCGGTACTGAGCTCCTTCACGACTGGACT

AACGACTTAATCTGCCCGAGGGGGTTAATCAAATCAATTCCTACGTCGCG

ACGTCCCCTATCTTGGGCTACAATACCTATTTTGCCAATTGGCCTGGACC

CCTTTTACTGCCGACACGGAGCCCCGCCGATCCATCACGACTGGACTACC

GACGTAATCCGCCCGAGGGGGTTATCCAACAGGCTCCTCCGTCGCGACGT

CCCCCGAACGCCCATCTGCTAGCCCGCTAGCCGCGGCCCGCTAGCTGTCT

AGAGCATATCGGACTGTTAGCTGAAGAGGTCCATCGGCCAATTTCTTGGG

CCACTATACCTATTTTGCCAATTGGNCTGGACCCCTCTGCCACACGGAAC

CCCGCTAATCCATCACGACTGGTCTACCGACGTAANCGCACGAGGNGGCT

AAAAACAGACTTCCTCCGTCCCTCTAAGGCCT

>rnd-5_family-2019#Unknown ( Recon Family Size = 41 Final Multiple Alignment Size = 40 )

GGGCTCTATTTTCGGCTGGCGTTAAGGCGGCGCNAGTGTCAAACGCACGT

TAGTTTGCAATTTCGNCNTGTAAAAACTGGCGCACTGNCATTTTCCAGCC

CTAGCGCCAGGNTCGGCAATTTACCTGTCTTATATCAGCTCGCTTGCGCT

NAGATGGGAGGGGTGGAAATATTTGAGGCGTGTCCTTAAAAACGTGATCC

AAAGTGCCAATTTCAGGCAGCGCTGGTAGGGATATTTAAGACCAACCAAA

AGCTGGTTTTAGCGGTAACGCGGTTGGTTATGGCATGAATTTTGACAGCG

GAAACGCAGCCTATCCAGCCGTGACGCACACTGCCCATATGCGCATGGAA

CAAGAGTAGCCTAATGTGCTTTTGGTGCCTGTATACTTCGTATTTAGAAA

CATAAAAAACNAATGTTTTTTTCCCCATTTCGTTTNATTTGATTAATTCG

TTTNATTTGATTAAAAACCAAGCATAGCCTCCCCTCCTCTCAANTGAGTT

TTTTTTTCGTCTGCCCGCAGTAGTCAAGACTGTCGATAAAGGGTTTGGCT

CGTGAGACCGCTTGGAAATAAAA

>rnd-5_family-1022#Unknown ( Recon Family Size = 45 Final Multiple Alignment Size = 39 )

ATGTGTGTAGCTGGTGTAGAGGAGTCAGGCGCAGGACAGCAGATATGAGT

AANNAACGTACTTTACTCAAACAATAAATGCAATACAAAAACATAGCCCA

CAATAACGGAC

>rnd-5_family-271#Unknown ( Recon Family Size = 41 Final Multiple Alignment Size = 39 )

GGAACACGATGGGAGACAGAGAGCTGGTTTCAAGCGCAGGGCGCGGCAGG

TGTTTATTTGTAAAGGACCACAGGAGGAGGCAGGTAGCTGGGTCCAGGGG

CAGGCAGAAGGTCATACACAGGGGTCCAAAAAGGCAACAGTACAGGCAGG

GAAAAGGCTAGTAACGTCGTCCGGGAGATCAGGCAATAGGTTGATAACAG

GAAATCCGATAGGCTAAAGTACAGGCAGGGAATAGGCAAAAGGCGTCGTT

AGTGAGGCAGGCAAAAACTATCATACACGGGAGGATTAAATTACGGGAAA

AACAGCGCTCCGAATAGAAGTGTGTCGCAAAACAAACAATACCTCACAAT

GATGGGGTGCAAAGAACTGAACTAAATAGTGTGTGATAATGACATACAGG

TGTGTGAACAGGTGATCAGAATTCAGGTGATTGGGATCTGGAGAGTGAGC

TGCGTTCAGGGGATCTACGTGTTTGAGAGTGTGAGCTGGAAAGTGAGCTG

CGTTCAGGGGATCTACGTGTTTGAGAGTGTGAGTTGGAAGCAGACGTTAC

A

>rnd-5_family-521#Unknown ( Recon Family Size = 41 Final Multiple Alignment Size = 39 )

ATTTTAGTCAATGCCACTCCGACATTGCTCGTCCTAATATTTATATATTT

CTTAATTCCATTCTTTTACTTTTAGATTTGTGTGTATTGTTGTGAATTGT

TAGATACTACTGCACTGTTGGAGCTAGGAACACAAGCATTTCGCTACACC

CGCAATAACATCTGCTAAATATGTGTATGTGACCAATAAAATTTGA

>rnd-5_family-168#Unknown ( Recon Family Size = 44 Final Multiple Alignment Size = 38 )

GAACTGCAGTTTCCTCTGCTTGGAGCAACAGTGAAGTGGGCAGGGCAGTA

CGGATTTCTTCTCTTACATCTGCAAGCAGAGTCTGAACATCAGACAGGAT

GGCATTGTCTCCCTCAATCCGTGCAATGGCTACTGCTATAGGTTTCAGGA

GTTTCAGGCTGCTTACCACTCTCCCAAAATACATCATCCAGGAGGATCCT

CTTGATGGGGCTGTCCATATCGGCAGACTGTGATATGGCCATTTCTTGGA

GAGACTCCTTCCCCTCCAGGAGACTGTCAAACATGATGACAACACCACCC

CAACGGGTGTTNTTGGGCAGCTTCAATGTGGTGCTCTTATTCTTCTCGCT

TTGCTTGGTGAGGTAGATTGCTGCTATAACTTGATGACCCTTCACATACC

TAACCATTTCCTTGGCTCTCTTGTAGAGTGTATCCATTGTTTTCAGTGCC

ATGATGTCCTTGAGGAGCAGATTCAATGCATGAGCAGCACAGCCAATGGG

TGTGATGTGAGGGTAGGACTCCTCCACTTTAGACCAAGCAGCCTTCATGT

TCGCAGCATTGTCTGTCACCAGTGCAAATACCTTCTGTGGTCCAAGGTCA

TTGATGACTGCCTTCAGCTCATCTGCAATGTAGAGACCGGTGTGTCTGTT

GTC

>rnd-5_family-4266#Unknown ( Recon Family Size = 40 Final Multiple Alignment Size = 38 )

TAGGGGAGAGTGGGGTAAGTTGAGCCATTTTTACATTCAGCATCACTCCG

TCAAGGGAAATATAGTATTCTTTCTAACAAAGATATCTACATATATTTCA

GGATGTTGTGTATCCCTGGAAATAAT

>rnd-5_family-842#Simple_repeat ( Recon Family Size = 49 Final Multiple Alignment Size = 38 )

TAACACTTTATTTGGATAGTCCATCTGTAGATGCTCTACAGACTATCAGT

AACATTTCAACTAACTATCTACTAACCCTAACCCTAACCCTAACCCTAAC

CCTAACCCTAACCTTACCCTTATTCTAAACCTAACCCTAACCGTAACCTT

AGCAAGCAGTTGCTTATCAACAGATAGTTTGTTGATAGTATGACCATCTG

TAGAGCATCTACAGATGGACTATCCGGACTATCCAAATAAAGTGTGACC

>rnd-5_family-5922#Unknown ( Recon Family Size = 50 Final Multiple Alignment Size = 37 )

ATCATAACCAAGTGGGAAGGTGGTAATTTACCAGTTGTGAAGTCGTAAAT

ACCAGTTGGATGCATTCACGTGCTTTGAACTCGTTGAGAAACGCCGATTG

GCTAATGGCCAACAAGCTGCGTCAACCATAAACTAAAAGTACAGCTATCA

TGCTTGTAAACAAATTATAGTGTTCAAAAACCATATTAATAGATCGCTTT

TTATAAATAATGTTTTGTTGTTGCATTTAACTGCCGAAAATGCTGTTATC

GAGGTAATTTCCTTAGTAGGTGACGTCAGAGGTCAGCATGTGGGAGAAGT

CGGAGCTCAGGGATGATAGACGAGTTTCCCACT

>rnd-5_family-1804#Unknown ( Recon Family Size = 44 Final Multiple Alignment Size = 37 )

AGGTCAGGCTTGTCTTTCACATGTCCCTGGTGCTACGCAGAATATCAGAG

AGGGGAGAGGNCAGGATGGAACATTGTCTTCATATGTAAACGTATCTTTT

AAACCATGTGAAGGGATGGCGTCGTNNNNNNNNNNNNNNNNNNNNNNNNN

NNNNNNNNNNNNNNNNNNNNNNNNCTACTCTTTCCCATTGGGGGAAGTGT

ATGGCAGTGTCTGGAACCATTCTATGTTCCCTCTGACGTTGCCCTTATCT

TGGGATAGTGTACGACCCAGAGGC

>rnd-5_family-325#LTR/Gypsy ( Recon Family Size = 49 Final Multiple Alignment Size = 37 )

TTTAAAGCGGGATGTGNCTCAGTTCTGTCAGGACNTGTCACACCTGTCAG

TTAACNGGNAAACCNAATCAGGCTATTCCNCCCGCNCCNCTNTGTCCTAT

NCCTGTCATAGGCGAACCATTCGAGCATGTGGTGGTTGATTGTGTCGGAC

CGTTACCGAAGACAAAATCGGGTAACCAGTTTCTGTTAACGGTNATGTGT

ATGGCCACAAGATACCCCGAGGCCATTCCTCTGCGAAGGATTACAGCCCC

GGTAGTGAGTAAAGCCTTAATNAAATTCTTCACGACGTTCGGGNTNCCTA

AGGTGGTACAAAGCGATCAAGGTNCCAATTTCCTNTCCAANCTNTTCGGN

CAGGTTCTCCAACAGCTCCATATTACGCACCGTTTGTCTAGCGCCTATCA

CCCAGAGTCTCAAGGNGCGCTGGAACGNTTCCATCAGACACTNAAGTCTT

TGCTGCGAGAGCTTATTGTTTGGAATCTGAGAAGGATTGGGAGGAGGGAG

TTGCCCTGGTTACTGTTTGCCGCTCGTGAAACTGCGCAGGAGTCCCTAGG

TTTCAGCCCGGCTGAACTAGTGTTTGGTCACACGGTGAGAGGACCAATGA

AAGTCCTTAAAGAACAGTTCTTGTCCCAAGAGTTGTGTACCGGAGACGAG

AATGTGTTGGACTACGTTAGTCGCTTTCGTGAGCGCCTAC

>rnd-5_family-249#Unknown ( Recon Family Size = 39 Final Multiple Alignment Size = 37 )

AAAGGACAACAGAAAACACACCTCTTTTAACAGGGAAATCATAATGAGTA

AATAGGACACACCTGAGTGTCGTTAATGTCTCTAGGACGGTCTCTGCCGC

CCTCTGGTGACAGGTGGAACCATG

>rnd-5_family-194#Unknown ( Recon Family Size = 40 Final Multiple Alignment Size = 37 )

TCCCTGTCACGGATGCCATGCCACGGTTGCCCTTAGTTTGAAGATGTAAT

CCGGA

>rnd-5_family-79#Unknown ( Recon Family Size = 41 Final Multiple Alignment Size = 37 )

CTGCAAAATGTTCTCTCCGCCAACAAGAGGGGTGTGAACAGTTTGTGTCA

TGAACAGTGCTTGTGCCCATAGAAATAGACGTGGCGCGCGCAGGGGGGGC

GCGGGATGTTCCCCAATGCTGGAAGGGGGGCCCGAGTGAAAAAGTTTGGG

AACCCC

>rnd-5_family-2227#Unknown ( Recon Family Size = 47 Final Multiple Alignment Size = 37 )

TATCGACAGTTGTCGCAGTGACATCATGAACATTCTATTGAAATGGTTAC

TTGCATAGTGGAGTCTTTTGTTAAGACANGTAGCTAGCTAGCTAGGTAA

>rnd-5_family-501#LTR/Gypsy ( Recon Family Size = 56 Final Multiple Alignment Size = 37 )

TGGACATTATAAGTACCGGGTCATGCCGTTTGGACTGACCAACGCTCCTG

CCGTGTTCCAGGCTCTGGTCAATGACGTGCTNCGNGACATGCTGAACCGG

TTCGTGTTCGTTTACCTGGACGACATCCTCATCTTCTCCCGATCTGCCCA

AGAACACGTACTCCATGTCAGACAAGTCCTTCAGCGCCTCCTGGAGAACC

ANCTGTNCGTNAAGGCGGAGAAGTGTGAGTTCCACGNCTCCCAAGTCNCC

TTCCTGGGATACGTCATCGCTGAGGGGAACGTNCAGATGGACCCCGGAAA

GGTGAAAGCGGTGGTGGATTGGCCTCAACCNACGTCCAGGGTGCAGTTGC

AGCGGTTTCTT

>rnd-5_family-1448#Unknown ( Recon Family Size = 58 Final Multiple Alignment Size = 37 )

TATGTGAGATACGCCGTGAGAGCNTTCGTGTCCAAACCGTTGCAGTGTGA

GAATTGTAAAGGATTTGGCCACGTTTCAAGTGTGTGCAGACGGANAGAGT

ATATTGAAGAAGGTGTGTGAAGGAGACGGTGTTGCAACTGTGGTGGGGAT

CATGNTCCCGAGTTCCCGGAGTGCCCTGTNAGGGTGAAGGAGGTCGAGGT

GGCAAGGGTCAGGGCTGTCCAGCGAATCTCCTATGCGGAGGCGGTGAAAA

GAGTCGAGAAAACAAGCGACGNTAGTGAAGATATGGCAGTGGACGCACCG

CAGCCTGTAGTAAATGTTTGCTGCCAGNCGAAAGATACTGCGTGTNAAAA

AGGTGGACTTTGTGGCGTTTATTGCCACGGTNATAAACTGCACGGCACAA

GTCTCAAAGAAATCGAAGAAACTGGACATCATTGTGGCTGCGGCAGAAAT

GTTTTTGGGG

>rnd-5_family-141#Unknown ( Recon Family Size = 46 Final Multiple Alignment Size = 36 )

TGATGTGTCATTTCTGTTCAAGGTGAAAACAGATCGACTTTTGTGATTTT

GAGTGGGCTTTTAGCGAAGCTTTTGACCTTNTGCGTCGCAACNTTTGTAT

CAGAGTCTATAGACAAAGCCCGTGGGCTTGTTTCTTGATCGAGCGGGCCC

CGGACAGGGTCTCGAGTGAGAGCGGGAGAAATGTGANTTTTAACGTCCTT

GCTAAGGGTGTTAATTCCTGCGGACCTGGTGTCCGGCAATTCCCCGTCTA

GTCCTTGTGACTTATCTTTTACCCTTTTCTCAAATTTTTCTCGCTTTAGT

TCTTCACGTAGTGGAACTTCTGGAGAACATTGGTCTACGTTTTGATCGTC

CTTCAACCCTTTGTTACCCTTGTGCTCTGACACTTTGTGTGGGTTGGGTG

CAGGAACGTAGCGAACAGGTCGATTGTAGCGGTAGTCGTTTCGATGGCGA

CGTACTTTACAGTTATTTCGACCGCGGAGGTTATTGGAATCGTGGTTTCG

TGGTAGAAACCGTTGTTG

>rnd-5_family-451#Unknown ( Recon Family Size = 37 Final Multiple Alignment Size = 36 )

TTGTTGTCATCTATCGCCCACCAGGTGCCCTTGGAGAGTTCCTCAA

>rnd-5_family-630#Unknown ( Recon Family Size = 37 Final Multiple Alignment Size = 36 )

TACGCACTAACGCCAGATAAAAATAATGAAAGAAAAACCTCAATGTAGCC

TATAGATATGAATTGCACAAGAATTATACATTTATGGATTTTTTATGCAT

TGTTTTCTCTTTATTCAACCCGCCCGCCACCCTCATCCACACAATATTTC

ATGACCCTAAACCCGCCCGCCCCGCGGATATAACCGCGGGGACTGCGGGT

TATGAGTCAACCCGCGCATCACTA

>rnd-5_family-1305#Unknown ( Recon Family Size = 43 Final Multiple Alignment Size = 36 )

GCGCTGCAGTACAGCGCCCTTAACCACTGCGCCACCCGT

>rnd-5_family-885#Unknown ( Recon Family Size = 38 Final Multiple Alignment Size = 36 )

GCAACAATGTTGCTACTAATCCATGCGATCCATAACGGGCGCGGTCCCAA

ACGAAAACAACCACGACCTAGAGCGATTACACCGATGATCGAGTTAAACA

CTCTATAAACTACACACGCTGAAAACAAACAAAACTTCTGCAGCACAGCA

CACACAATCAAACTGTCGCGCCACCAAAGAGCCCCTCCCCAAAGAGCCGA

ACGAGCTCTCTTTATTTCCTCCTTCCTTACTGCTCATGCGCTCTTAAAGT

GGCAGCGCACGGTGAAGGCTCCGTGCAGATTTCGCCA

>rnd-5_family-107#Unknown ( Recon Family Size = 51 Final Multiple Alignment Size = 36 )

TCTGTTTTTTTAACATCCATTGCGAATGATAATATATTAAAACTATAGTT

CCCCAGAGTAGGCTATTGAATTTTCTTTCAACAATCTCCCCCTCGATAGT

CACCAATCCTCGGTGTGAAAGAGCGATGGAAGTTATAGGCCTACTGCTGC

ATTGGCCTATAGGCTATGAGTTCCATGCGCTCATTTCTTTAGCCGCCAAT

GGATCGCGGCGTATCAATGTGTCCATATGGCAGAGGCTGGTGCTCTCGCA

TTAGTTGAATTTTAACTTTTAGATTTTTATCATTTTAATTCAGATTTTTT

ACGATTAACCACGTGACAATGATTTTGAGAAACGAAAAATGCGCATATGA

AAATCATAACCGGCACGCAGATCGGTAGAAATGGTAGGATAATTGTAAGC

TTCCCCGAACTTGAAACTCGCGCGCCGCCTATGAAGTCTTTATAAAATAA

TTGCCTCCACGTTTCTATGGTCGGATTTCGNCTATAGGCTACTTTGAAGC

AAGGTAAGACATGCCTCATAATATGAAGTAAAACGTTCGGGTTTCAAACA

ATTAAGTATGTTTTCAAAACGCATNCTGCCTCCAGCTCACATTGCAAAGC

GGTGGGTGACTC

>rnd-5_family-1122#Unknown ( Recon Family Size = 37 Final Multiple Alignment Size = 36 )

GTACTGTATGACAGAGTCATAGACCGTTTCGNCAACATGAAAGAGGAGGA

TGGCATTGGCGTTTCTCTACAAGTAGGGTGAGTCAACATGTTTTTTCTAC

TTGCGCGCACGCACACACACA

>rnd-5_family-1474#Unknown ( Recon Family Size = 39 Final Multiple Alignment Size = 36 )

CTCATTATTTATTTATTTATTTAGAGAGCGAGAGAGAAACTCCAAACTGG

TCACACTTGTAACGCAGCCAGTCTTTCATTCAAAACCTGTCATTGTGCAT

TCATTTGGATTGTAAACATCTCTCACCACACAACCATTGATTCAAAATAT

AAGTTTATTGTGAAATGTAATGAGAACATTGGTTTAATCGCCGAAACACA

ACATAATGATATCTTTTCAAAGTTGTAGTTTTANCTACCAGTTAATCCAA

TAGCAAACGATGCAAATCGCCCNTAGAAGTGCTGAAAGTAATATGCTACA

GTACTGTAAATGACAGATTACAGTTTGCACATTACGCAACACACTTACTT

ACATTACCCAACAAAACTGACAGAAAATCTAGAATTTCCATAATATCTAT

CCAATGTGTAATCAAAGGTGTGATCAATGAAGACATCCTCTACAATCATT

CATGCAACATGTATTTAATTTTGTTCAGATATAATTGCAACATAGGTGTA

CTGTCTGTAATCAAAGAAATTAAATGAAACAAATTAAATGAATGAAAATA

AAACAACGTTTAGCACGCATTCGTTTGCATCAAGCCTGTCTTGAGCATTT

GGCCATAAATTCTCATCCACATCGCAGTGAATGTCCTCATTGTTCATGCA

CCGCGGGAAAAACCTTTTGGCATGGCGAAT

>rnd-5_family-1416#LTR/DIRS1 ( Recon Family Size = 37 Final Multiple Alignment Size = 36 )

CTCAACATTGACAGGAGTGTTTCAAACAAAAGACAATGAATAAATTGACA

AAACTGACGCATCTTGCGGGGTCAGGTCTGGGTGGTCTGCCAGGGGCCGT

TTCATTTAGTATCCGTTTGGGTCGGCATGGGGAATGATTNTATTTCCCCA

TAGTATGTTGTACCGAAGTTGACCGACTGAAAGGGAGCGTAACGTTATGA

CTATAACTACTGTTCCCTGAAGGAGGGAAACGAGGTACAACACCTGTTTG

GNCCCGCCCCTTCCTGGGTCGGCGAAGAGCGGTATTAAATTGAAGGAATG

AATCCGAGCCTCACGCTCATCACCTGTGGAAGGCGGGGCTTCCAGCGAGG

CGCGGATTTAATAGTATGTTGTACCTAGTTTCCCTCCTTCAGGGAACAGT

AGTTATAGTCATAACGT

>rnd-5_family-485#Unknown ( Recon Family Size = 44 Final Multiple Alignment Size = 36 )

GGGTTGCAAAGGGAGGGTATATTACTGGAAACNTTCGAAGTTTACCAGTA

AACTACCAGAATTTTGGTATCTTTCAAGGATTTTATGTAATCTATCACAA

GACATCTAGTGGCCCTTTTGGGTACTTCAGATTATCACAGGTGTCTGTAA

TTATCTCTGGCCCTCTGTGTGGCCTTATCACGTGTAAAATATATGAAATA

ATAAATAAGACGATTTTAAAATATCACAAAAANAAAATAGAATGACAAAG

CTGTAAAACATTATCCTAAATATAAACCATCAACTTAGTGAATACCATTG

GTGTTTAATATGAGGGTTTCAGCATGAAATATCCTTTATATTTTTTTACA

CACTTTATTTATTTTACTATGTCAATATGTATTTGTTGTNAATGTTTTGG

CGTCAAACTGGTGGCAGTTGTGAAAAAAAGTCAATAGTTGGAAGAGTTGC

AGAGTTAATTGAAAATAATGCCATTGTTTTCTCTTNACCCATATGGT

>rnd-5_family-1478#Unknown ( Recon Family Size = 40 Final Multiple Alignment Size = 35 )

TTGGCCTAACCCCAGAGAGATAGAGATATGCCGGCGGCATGCTACGACAC

CGACACGCATTTCTTTATGTCAGATGGCTACTGCGTCTGCTATACAGATA

TAGACGTACAGAAGGAGGCTAATTCGTCAATTTTCNATCAGAATATTTTG

TATAAAGATAAGCATTATATTGTTAGATTATAGGAGTAACTCTGGTAGGC

CTGAAACAGTCTTCCTCACCTCAAGTTCAACTGTCGGAGTCAGTTGGAGC

GCCGGGGCGCACTGCCTTCATATCATAGGCCTGCAGTAGCTAAAGCTTAA

TAATAGCCACACCATACCAAACCTTCACAAACGTTTCTAAACTTTATTAG

GGTAATATCAAAAGTAAGTCAAGCCATTGTTTATAAGGAAAATACGAGCA

GAAGAGCGAATGTAAACCAGGGAAAAAACGCACTACCCTCCCCTGTGCCC

CCCCAAAAAACACACGACCCTCCCCAAAGCAACAAAAAAAAGTTTGACAA

CCCTCCCCTATTTTGGACCACCCCTCCCCCCAGTAAATTTCGATCTG

>rnd-5_family-117#DNA/TcMar-Tc1 ( Recon Family Size = 53 Final Multiple Alignment Size = 35 )

GGAAGGCTACCCGAAACGTTTGACCCAAGTTAAACAATTTAAAGGCAATG

CTACCAAATACTAATTGAGTGTATGTAAACTTCTGACCCACTGGGANTGT

GATGAAAGAAATAAAAGCTGAAATAAATCATTCTCTCTGCTATTATTCTG

ACATTTCACATTCTTAAAACAAAGTGGTGATCCTAACCGACTGCCAAAAC

AATGAGTTTTTACTAGGATTAAATGTCAGGAATTGTGGAAAAACTGAGTT

TAAATGTATTCGGCTAAGGTGTATGTAAACTTCCGACTTCAACTGTA

>rnd-5_family-4787#Unknown ( Recon Family Size = 39 Final Multiple Alignment Size = 35 )

TGTGATAGAAAAATTACGTATTTACCTGATTTGTTTGATATTAATAGTTA

GCAATTAATACTTAACAAATAGTAAGACATTTCTGTCCTGCTAGTGTCTG

TGTTTTCATGGTCTCCACTCTAGTTCCCTGCAGCTAATCTGGGCGTATGG

TCACCGGAGGCGGCTTGAGCTGACAAATGAGTTAAAGACTGCGTTACGTA

GACAAGAATGTGTTTTACTAGAGATAGCTTAGGAGTAGAACAATNCCTCA

TTGTCTCAAAATCATCCTTGCATCTGGGAAACTAAGCCGGGGTGGGGTTA

AGACAACACCCACGTGCAGATAACGACAGGATGAACCCAGAGTGGCTTAT

GATGCTCCCCGGTCTGCATGGGAGGGGTGGAACCGACCATGACTGAGCAT

TNTTAGTGTCAGCTATATAAAAACTCTGCATTTCTGTAAAGGTTAGGCTC

TCGGCCNNACACTCAAGAGTGTTGGGTCGACCGGCCTCATTATTGCAATA

ATTAATCGATATTAAATAAAGATGATTGTTTGAAGAAATGACCAAGTCTC

TCTCAGTACTGAATTTCCACA

>rnd-5_family-1471#Unknown ( Recon Family Size = 38 Final Multiple Alignment Size = 35 )

CCTGTAATCTAAGAAATCTCTGCCCTGTGAGTGTCCGTCTCCTAGGTGAC

TATCTGCATTCCATGTGGGAGGAGCGTCGCCCTCCACTTTCACAGTCACC

TCATCTACGACTATAACCTCCCCTTTCTTATCTAGGCACCCTTCAGAGTA

TACACTACTACTGTACCGGTTCCAGTCCCCTCTAGACAGATCAGTCTGTG

TCTCTAAACCCAAGGGCATGTTGCCAGGGTCCATCTCTGTAGCGTAAGAA

CAAGACGGATCATCGCCGCCAGTGTCTAACGCGTCACCGTCCCCACGGGA

ATNAACCGTCCTCTGGCTCTGGTGAAATACCGGTAAATACTCTGAGCCGG

GAGCAGGAGGACAGCCCAGTCTCCCCAGCCCCAGTCTCTCTGGGTCTGAT

CTGTGGTCAGATCCTGTGTGTAAGAGCCTGTGTGTTACAGTTAAAGTCTC

GGTGTCCGTCTCTGACTTGAGGACGGCGTTCGGCGTTCCACTGACCTCCG

TGATGCTGCGTCGGGTCCTGGGCTGGGGCGCGGCGGCGGTGGCGGGTCCT

CCGTGGCTACAGGGGGCGCTCCAGCCGCTCCAGTCTGGATGTCTCTGCTG

TGTCGTGGGTCCTCCTCTCCTTCAGNCCTCTCCTGCTTGACC

>rnd-5_family-202#Unknown ( Recon Family Size = 39 Final Multiple Alignment Size = 34 )

GCCTGCTCCCGCTCTTCCTCCCCCTGGCGCTCGAGGGCGCCAGGCTNCCC

TGCATCACGCACTCAAGCCACCATCAGTACGCACACCTGCCTTCCCCCGT

CACGCGCATCAGCGATCATTGGACTCACCTGGACTCACATCACCTGTTCA

TTACCTCCCCTATATCTGTCNGTTCCCCAGCTCTGTTCCCCGCTGCTGCA

TTAATTGTCTTATGTCCGTGTTACCCGTGTGCTGACGCTGTTCCTGTCTC

GTTCCATGTCCGTTCCTTATTAAATGTTTGACTCCCCGTACCTGCTTCTC

GTCTCCAGCGTCGGTCCTT

>rnd-5_family-3575#Unknown ( Recon Family Size = 40 Final Multiple Alignment Size = 34 )

ATATTAATATGGGCTATTTTCAGCCCTTTCCCGGGTAGCTTATCAGAGAT

AGACATAATATGGAAAAGAGCAAACAAAGCAAGAGAAAAATAAATACATT

CAGCAGTCCATTAATCAGTGTGTGTGTGTGTGTGTGTGCTGCGGGGTTGA

AGCTACGAACCCATAGGCTT

>rnd-5_family-763#Unknown ( Recon Family Size = 36 Final Multiple Alignment Size = 34 )

TACAGTTCTTACACATGTGTAATTGACAAAAAAATCGAAAGAATTTCGAA

AAACGGTCCAGAAAGCACTTTTTTAAGGGGGTGATAAGTTTTTGACAAAA

TTTTCAATTTTCAAAATTTTGCTTTTGGGTGTTGACTTGTGTTGCATTTG

CAATATGAAAAACCACGGTGGAGCGCACTCGCCATCTGTTGGATTTTTGA

AGTGTTACACTTGGCATTTGCCATATGGCATTTGCAATCAACTTTGAACG

AAACGACGCCGAATTGAGTGGTATTGGACATCGTGTATATGNTTCGTCCG

GTGCCAATANNTTCCCATTCATTTTTGTCCATTTCAGGGGGGTNTTATAT

TA

>rnd-5_family-1359#Unknown ( Recon Family Size = 39 Final Multiple Alignment Size = 34 )

TTATTTTCCGGTTTTCGGTTCTGTTCCCTGAACCGGTTCCAACCCA

>rnd-5_family-1834#Unknown ( Recon Family Size = 40 Final Multiple Alignment Size = 34 )

AAACATGGCACTTCGATACAGCTTCGACCGGAAGTTACTTTTTCGTAGCA

GGTTAGAATTTACGCAGCAGGTTAGGAGAATTAACGTAGCAGGTTAGGAG

AATTAGGTTAAGGTTAGGAAAAGGGTTAGGGTTAGCGAAATGCTCTCCTA

ACCTGCTACGAAAAGTCACTTCCGGTCGTAGCTGTATCGAAGTGGCGTGT

TTTTAGGAAGTC

>rnd-5_family-1588#Unknown ( Recon Family Size = 38 Final Multiple Alignment Size = 34 )

GGAGAATCTCAATTGCATACTCCTCGCGTCCTCCTCGCCTCCTCAAAACC

CATTGGANGAGAAGGTCAGAGGTCCCGCCCCTCTGACCTTCTCCTCCAAT

GGGTTTTGAGGAGGCGAGGAGAGAGGACGCGAGGAGTATGCAATTGAGAT

TTC

>rnd-5_family-355#Unknown ( Recon Family Size = 44 Final Multiple Alignment Size = 34 )

GTTTTAACGATGCATCTTTCCTACAAAACGGCCGAAGATGTAACATGCGT

TTCCCAAAACACCACGCAGAGAGAATGTTCGCTAAGCGCGTCGTTGGAGC

ATGTGTCGATACTGATAGGGTCGAAAGAAAGGCAGCGCTGCTCTCGGATC

AGCTTTCTCCATTCAAATCTTACCTTAACATTATAATTATTCCACAATAC

TGACGACGGATCAGCTCCTAGAGAGATATTACCACCTATGGCTGCAAATA

TTGAGGCGGCTTATTCTAATGCTTACCCTATAANAATGCACTAAATCACA

GATTTGGCGCATCATTGCGCACGTTAGGCTGCACAGCATGAAATATATTG

AGACAAAAATTACAGACAGTAGCCTACAAGTAGCAAAATAGAACTCAATT

GATTGAACATGAAATTTATTTCAATATGATTGAAATATATGGCCTATGTA

GCCTATACTGTATTTATATTTTAATGCAGTCATCTCGGTTTTCATTTGAA

GCATTTTTATCCTTCGCTTGTTTGTAACAATTGCAAAGACATTGGCAACT

TTGTATTTGTAGTCAACTTCGTTCTGAAGTTATCGGCGGTCACAGAGAGA

>rnd-5_family-785#Unknown ( Recon Family Size = 40 Final Multiple Alignment Size = 34 )

AAACAACCAGAATTACAAATTCATACTCATGTCNCAACACCAGCTAGCCG

GCTAATGTTAGCTAGTCAGNTAACTTGCTAAATCGCGATTTTCGTAAATT

AACTTTATGATAAAAAAAATATGCATAATCGTTTGTCTCTACATTAACTA

ACATATTCCTGTCAACTGTATATTTTTTTGCATGATTCGTTATGACGTTA

TAATCACTTACATTTGCTTCCATCNTAGTATTTTTGTCAGCCATCTTTGC

TGAAGAAAGTCTCCAGCCTTGGGTCGCATAGCGTCAAATTCGTCATGGGA

ACCACTCGATATGATTGGTCATCGCCCAGCGGTCGCCGCTGGTGATTTGC

ATAAAGTTGGGAACG

>rnd-5_family-3563#Unknown ( Recon Family Size = 40 Final Multiple Alignment Size = 33 )

GTGCGTAGTAGAGCGAGAAAAAACGAGTGCTAAATGCTTGTAAGTTTGTA

GCTCAAACGGTTCGGTCTGGACAGTCGGTTTTGTGAGANGACGTCTTCGA

AAATAGGATGTGGTGTCTGAAGGTTCNGACAACTGCCATAAAGCTTGTGG

ATGTCGTAGAGCAAAACAACCGACACTGTCGTGTTCGTGAGAGTCTCCCC

TTTCGATGATGGGGTGACATATTCGTTTGTAGCTCACACGGTTCGGGTTT

TACAGACGATTTCGTGACGATTTTCTTGTCCGGATCCTCTCGTGTGTAGA

AAAAGGCCGCTTTCACAAGCCCCATGTTATGGAATAGGCGCAAGCTTTAT

ATCGGCGGAAAGAGGGGATTGAGCTGCAGCCACTACAAGAAACGGTAAGT

CTCTAGCATAAACACACGGGGAGCTATTCAAAA

>rnd-5_family-932#Unknown ( Recon Family Size = 73 Final Multiple Alignment Size = 33 )

TGGGATTTGAACTCACAACCTCTTGGTTCACGGCATTCCGATCTTCCTGC

TACGCCACCATGTCCGTGTCAGTTATCAGTTAATCTGACTATTCTCTCAT

CATTGATTTGATTNACTTATTTCAGCAATTTAGGGTTTTCTGTATAGTTG

TCTAATGTTGGTGGGGCATATTACTCTGTTTTAATGTTACTCCTGAATCA

CTATGATAAATAAAATAGTTTTTCTTGAGTGTACTTTTAACAGAGCTGAG

ATTTACTTNGAAGTCCAAAGTGTAGGAATACAGGTGAAATCAGTCATCGA

CACAGGCATGGCGGCGTAGCAGGAAGATCGGAATGCCGTGAACCAAGNGG

TTGTGAGTTCAAATCCCAGGTGAGGACATGTTGAATAATAATTACTGTAT

AAATGAACATACGCAATGTAATCATGTACAGTATGTCAACGTGTGTCAAA

CGTGTAAGTTGAAAACATTGTATGTTAAAAGCACTGTGTGTGTGTAACTA

GTTCCACAGCCTTTTTTTGTTGTAGATGAAGACGCCCAAATAATAATTTC

TAGTTGAACGAACATATGAGACAANTTGAGCAAANACATCATTTTAAGTT

GACTGAATTTTTGCCTAAGTTGGAGCTAAATTTGGGCCAACTTTTTGAGA

TGGTTCAGGTAACACTTTGACCGAA

>rnd-5_family-697#Unknown ( Recon Family Size = 34 Final Multiple Alignment Size = 33 )

TTAGGGTTGAATATTTTCCCGGTATTTTACAAATGTTCCATCCCGAGAAT

AAATCACTTTTCTCCGGGTAACCCGGTATTTCCCGCCAAAACCGGAAGTG

TCATTCTAAAGCATATATGTCTGGATTTGATTAGAGCTTTGATCGGCATG

AAAATTCAAGCCTGATGCTACCTGAGCCTGATGAGCCATACATTAAATAC

ATTTTATGAGCCCTACATTAAAGACATTTNATGAGCCCAAGCCCAAAAAA

GCCCAAATGATTGTGCCGTTATCCAATACATGGCCTATGATATATAGGCT

ACCGCACATTACGCACGACAGAAAAACATAAAAGCCCATAGATGTAGCTA

TGCTCTCTTGGGTAAATAAATGAAACNAGCTCCAGTAGGCTAATNTTTTT

GAGTGTGAACTGTATTACTGTACTGTATTGTATTATACTGTATTATATGG

>rnd-5_family-656#Unknown ( Recon Family Size = 40 Final Multiple Alignment Size = 32 )

GCGGGTTGCAGGAAACCAGGTGCGGAGGCTGAGGCTGGAGCGAGNGGGGT

TGGGACAGGGTAAGCAGGTCCGGAGGGGAATCCAAGGGAGCGGTGGAGTG

GGGAATCCAGGACAGGGTAGCGGGGTGGCGAGACGTGGGACNGGAGACAG

GGACCGGAGTCAGAGCGGGCAGAACTGTAGCGGAGAGGAAAACAGCGTCA

GGCAAGGAAAACAGGCACAGCAGGATAACAGGATCTGAACAGTAACAAAC

GGCTAGAANCGTAGACTGACTGAGCAGAGATTACGATCTGGCAGAGTGGA

AGTGGCAGGGACTGAGTATTTGTAGAGGTCTTGATTATGGAACGGGTTGC

AGCTGGTGGGGATCTGCTCTGACTCCAGCACACCTGTCTCCGCCCACACA

ATCACACACACAGAGAGAGAGGGAGAGAGTACTGGGGGAGTGGCGGCAGG

TCAAGGAGACACNGGATGAGCANTAGAGGGCGTGGCAGGAGCAGAT

>rnd-5_family-895#Unknown ( Recon Family Size = 33 Final Multiple Alignment Size = 32 )

GGCTTCAAAGCCTCTCAATGGCCAATACATAGCATCAGCAATCCAGGGTT

TATATACATCA

>rnd-5_family-903#Unknown ( Recon Family Size = 33 Final Multiple Alignment Size = 32 )

TCAACCTTTTCTGGCCCATGGTGTTGCGTGTGAATGTTTATCCTTTTAAG

C

>rnd-5_family-1594#Unknown ( Recon Family Size = 35 Final Multiple Alignment Size = 32 )

TCTTGACATCTAGCCACTTAGCTAGCAAGTTAGCAAACCAAATGCATAGC

TGGAGCCCTGAGCTGGATATAATTTATTTGACGCATCTTAGATTTCTTAT

AGTTATACTTAGCGCCCTCGTAGCGAGCACCCTCGCAGCTCCGCTGTATT

GAAAATCATACCGTCGGTATTTCGAAAAATACCCCGGTATACGGTATAAA

CGGTATATCGCCCAA

>rnd-5_family-8#Unknown ( Recon Family Size = 32 Final Multiple Alignment Size = 31 )

AGCTTTGTGGAAGTTACCTGTGGCGCTGATGTTTAGGCCGAGGTATGTAT

AGTTTTTTGTGTGCTCTAGGGCAACGGTGTCTAGATGGAATTT

>rnd-5_family-554#Unknown ( Recon Family Size = 54 Final Multiple Alignment Size = 31 )

AGAATTGTACTTAAACTCCCCCCTCATACTCATCTGGAGGTCGAGCATTT

TTTTTGTCTCTATCTCTGTAATGTGTCTGTATCTATGTAATTGTATATGT

ATTTATGTAAAAAATAGGGACCACAATGGAAATAAGTCCATTTAAAAATG

TTTTTGGTGTATACTGTATACTTTTTTTTTTTTNACTGACAAATNAAATC

AATCAATCCAAACTGTGCANCGGCCAATTGATGAATGGAAAGAGACATCT

GTTACAAAACACCAAAAAGTTTAATGACATTCGGAGATTGTCAAGCCAAG

CCTTCGATACTGGGTAGGCCTACAAGGTAGGGAGGGATGTATTTTCTGTT

TGTCGAGATTGACGTAGTCTATTTATTGTGGTGTTGAAAACGCAAACCAT

GATATTGAAGCGCCCGAAGTCGGTATGCTTTGTTTATTGGTTGTGTGGTG

CATTGGCACAGAAACCTGTTGGTGGAAAATGTGTTGCGTATATT

>rnd-5_family-123#Unknown ( Recon Family Size = 82 Final Multiple Alignment Size = 31 )

GAGTATTATGTGTGCCAAACAGGTGCTGTTAGATTACAATATTATTTTTT

CTGACCGTTTGGAACAGTGTAAACAACACTAAATAAATTATAAGTAATAC

CAGAGNCTGTTCTAACGANAAAACATTGTAAAGCCTTTATTACAGCATAG

CAAAGATTAAAAACAGCCGAATTTGTGAAATTGCTTTATCCAACATTTTG

CGGTTGCGTGAGGCTTGGTGCTCACGGAATCAGTAGGCTATTAAACAAAC

ACTCAAACAGGCAACAGAAGCAGGATCTGTCTTATTTCTGTATATAT

>rnd-5_family-83#DNA/hAT-Charlie ( Recon Family Size = 33 Final Multiple Alignment Size = 31 )

TCCGTTTGCTCGAAAATTGATAAATNTTTTNAAAAAAGTAAGGCCCGCGT

CCATAGAGACACATACCAGCTCTACTGGTAGTACTGCTACTACCAGCAGT

ACTACACCTGCACCTGTCGACGACACAAGTTGTTCTGCTTCCACGAGCAC

ATCCAATGCTAGCATCAGTAATTCTACATTTGTTGTTAGCCCAGCTAGCA

TGGACACTGACAGTTGTGAATCTGATGCAGCCGAAGAGCTACTGCCCCCT

TACCCGGGAAAGCACCGAACAACAGACAGGGACGTTGGACCATCGAAGAG

GCGCAAATATGATGAGAACTACATTGATTTGGGGTTCACTTATATTGGGA

GTAGTGCCTTTCCTCAGCCACAGTGTGTTATATGTGCAAAAGTACTATCT

CACAACTCGATGAAACCTTCACTCTTGCGCAGACATTTAGAAACAAAACA

TGCCAATTTGAAAAATAAGCCACGGGAGTTTTTTGAGCGAGAATTAAGAC

GACTTTCGAGTAGTAAGACATGTATAAAAGCAACAGATACCATTAATAAG

AAGGGGCTAGAAGCGTC

>rnd-5_family-786#Unknown ( Recon Family Size = 33 Final Multiple Alignment Size = 31 )

GTGGTTTAGGCCCGGACCATCACAGTAGGTGTGAGCAGAGCATGTTGAGC

AGGATGGGGCTTGGGCGGGTGTAATAGTGGGGGTTGGGCCTGTTGCTCTG

CTCACGGCCTGGGCGTATGTCCTGCTGTCATGTTGAGGTCCTTGCCGCGG

GGGTGGGGGGCATGGGGTGGGCAGAAGGGGCATAGGTCTGATATGGGGGG

GCCTATATAGGGTGTGGCCAGGGTTTGCTTGGGGTGGTCTTGGCTGGTTG

GGGTGTGGCTGGTGGTGCTGTGGTCTGGGTGTAGGTCCTCTCGGCGTGGG

TTCTCTCGGTGCGGGTCCTTCGGGAGGGGGTCTCGCAGGTCTGGGAGGGT

GTCTCGCTGGTCTGGGCGGGGTGTCCGTTGCTCTGTTGCTCCTGTGTGAG

GTGCTGGGGCTGCGGTTGCGGTCCTGGCGAAGGTGGGGACTGCTGCCTTG

TAGAGGTGGACCTGGTCGTAAAGGCTGTTCAAGTCCAGGGTGGAGTGGTG

GGCCGGGTAAACATTAGGTTTTGAGGCACAGTCTCGGGAAATGCTTGCGT

TTACCCGCTATANGGTGGCAGGGTGGAAGTCTTTTCGTGGTAGCAGGGTG

GAGATAACCACTTGTGCGTTGGGGAAAGTAGAA

>rnd-5_family-3247#Unknown ( Recon Family Size = 36 Final Multiple Alignment Size = 30 )

TCGTTCCACCTCAAAAAGCACAAGAAAGAGGATTTCGACACCCACCATCT

CAGATTGTTCTGAAATCGTTTCTGTTGTTAGAAACAGATAAGATTAGCAT

TCCTGAAACATTATTTTGTTGAAATATAATTTGATCTCTGAGAAATTAAG

CTAATTGATTGCACCCAAATTGGCCATTTTAATTTATAGGATTCATATAA

TATTCAATAAATATAGTACCTAACATCCGATTTGGACCANACTTCTTCCT

AACAATGAGTAAGACGTGAGGAATCCAAAGAAATGGTCAAAAGCCACCCA

CGGACCCCCCACACCCACGCCAATCCCACCCCAACAACCAGTATACAGTA

TCAGTTCTCTATGTTTGACAAGTAGTATGGAGCTGCGGCTCGGAGTATTG

CTTCTNCATCCTATGTAATGT

>rnd-5_family-804#Unknown ( Recon Family Size = 32 Final Multiple Alignment Size = 30 )

TTTTTGCGAGAGTGAAACCTCTCGCTTCGCCTCTTCCTCTGTTTACACAC

ACCAAGCCCCTCCCCTGCCACTCACGACAACAAAGNTGAGAGATCACTTC

TTCCTC

>rnd-5_family-262#Unknown ( Recon Family Size = 43 Final Multiple Alignment Size = 30 )

AGTGTTGGGGAAGCTACTCTGAAAATATAGTTTACCAAGCTACCAATTAC

TTCACACTGGAAGAAGTTAAGCTACACTAAAGCTACCCTTAAGAAANATA

TAGTTTACTTAACTAAAGTTACTTTGAAAAAGTAGTTCACTACATCCAAA

CTACTTCGTGAAAAATNATCATATCTAAATCTGAAATGTCATAGACTACA

AATTGCAAGAACAGATCACTCTGGGGTCAGATGTTAACAGAATGTGTAAT

TTAGCCTATTAAACACAAAAACAATGTTTCAAGTGAGAATTAGGCAGGTC

TGATGCCGAAAAT

>rnd-5_family-1098#Unknown ( Recon Family Size = 31 Final Multiple Alignment Size = 30 )

CTTACATCAGCAAACAGCTAGTTTGTCTTTTCTTAGCAAGTTGCCCTAAA

TCTTGTGAGACGCTAATTGTTAGCCGCTAATGCTAATAGCTAGCTAGCTA

ATAAATGTACTGAGTAAGAGCAAACGTAGCTAGCTAATACAGCCTGATAA

TACCAGTGATGGTGTAGACCTAAATCAGCATGTTGT

>rnd-5_family-1267#Unknown ( Recon Family Size = 55 Final Multiple Alignment Size = 30 )

CTCCACACATTAGCACACACGCACTCATTCATACATATACATTCATGCTA

CACACACATCACAACTGCTGCTACCAGACTCTTATTATANTGCTCAGTTT

ATACACTGCCCCCCATTCCCCCCTTCCCCAATACACGTGTAAATATTGGA

CTATAAATTGTGCCTTCCTGTATTATACTTATGCTAAAATGTTTATTCTA

TTCTACTGAGCCATTTACTTTATGTTCGTATTCTTATATTTTATTATTTC

TTATTGTTG

>rnd-5_family-1989#Simple_repeat ( Recon Family Size = 44 Final Multiple Alignment Size = 30 )

TTTAGCTGTCGGCTATATTAGCCACGACTTACCGTTCTTTGTGCAGCTTC

AAATGTCGAACAAAGTTGGAAATTGTTGCGCCTCCGTCTGTAATTTTCTT

CCCGCATGTTTTGCAAGTTGCAATCCGTTTTTTGTTGATACAGCGTCGTC

TTTATATCCGAAAATAATAATTTGGGGTATCATCTTTCCAAGGGCTCCAT

CTGAATTCACCCGCCGACGTTCCTCTGCACTGCCACGCACAACTTTTTCT

CAGCTGGCACGATTTGATTGGCTGCTGTCCGATTCAAACTGTAATCCGTT

AAATGAAGAGTTGATGCGCTGCACACTTTTTTTAATAGCATCATTTTTAA

TATTTGGGCTTGGGGAGGGTATCAAGTCAGGTCGAGTCAAAAGGCTCAAG

TCCGAGTCAAGTCACGAGTCATTGACGTCAAAGTCAAAGTCGAGTCGCAA

GTCGTCATATTTGTGNCTCGAGTCCGACTCGAGTCCAAGTCATGTGACTC

GAGTCCACACCTCTGA

>rnd-5_family-1755#Unknown ( Recon Family Size = 33 Final Multiple Alignment Size = 30 )

AGGGGACGGGGGGGACATGCCCCCCACATTCTGAAATTGCATTTTTGTCC

CCCCCAGTTTTATCATTGGAATGTGATACAAAACGAGGCAACGGTGTGCT

TTAGGACCATGCGGACGCCTCCGAGCGGTCGGGTAGGCTGTTTGGAGTGT

TTATCCGACTGGATAAAAAAAATAAAATAAAATAATAATAATTATGTCCC

CCCCACTTCTAAAACCAAAGTTGCGCCCCT

>rnd-5_family-24#Unknown ( Recon Family Size = 34 Final Multiple Alignment Size = 30 )

AGAATGGGTCACAAGGTTAAGATTCGTATGAAAGATACTTATAATTCACA

GCAGACAGTATCTGCTGTAAAGACNGTTCTCATTGTGTAGAGACCAGGGT

CTGGCCCTGGGGCCATCTCTCCCTGGTACCNTATAGAACAGAAACATTAA

CTCATGCTCTGGAATGCGGTCTCTAGGTTTTATCACCCAAAGTACATCGT

AAATCTCCTGTCAGCGTTATCTCCCAGAGGCCCATCCTCAGTAGAACACA

CACACGATAGGTTCTAAGAACCCTCTATTCTGTCGCATAAAACAACCATT

TGATGCAATAAAAGTATTATAACATAA

>rnd-5_family-207#SINE/5S ( Recon Family Size = 33 Final Multiple Alignment Size = 29 )

GGAATGGGAGATGCAGTGCTGAGGCAGAGGGCTCGTAGTGAGGACGACTG

GCTANTACTCTGAACTGTGTGTGGTGAGCTTTCTGGCGCCCAGAGCTGCT

GAGGCATCACCCAAGTGGGTGCCATACATTGTGAAGGAGGAGAAGTCCAG

TAGCTACACGACTCAGGCTGGTTCCCAGAGTAGCCCTCTACAAGATCGTC

ACCAGACTCCATCTTCATCAACCATCTCCCTGACCACCTTCCTCTGATCA

AGCCATGAACTGTCCCTCTCCATCTTTGGAGGATGCATGCACTCAAAGAC

TTGGCCTCTATTATTGGTTGACCTAGCTAACTATAGCTAGGCTACTCATG

ATGATGTATTGG

>rnd-5_family-2806#Unknown ( Recon Family Size = 30 Final Multiple Alignment Size = 29 )

AACCCTTTACACTCGTGGGAATTGGCCTATATGGATAGGGCTAAATTG

>rnd-5_family-124#LTR/Gypsy ( Recon Family Size = 33 Final Multiple Alignment Size = 29 )

TTTTTGTGAAGAAGAAGGAGGGAGGTCTGCGTCCTTTCATTGATTATCGA

GGTCTAAATTCCATCACAGTGGGGTTTAGTTACCCACTACCTCTCATCGC

TACGGCGGTGGAATCATTTCACGGGGCGCGCTTCTTCACAAAACTGGACC

TGAGGAGCGCGTATAATCTGGTGCGTATCCGGGGAGGAGATGAGTGGAAA

ACCGCGTTTAGTACTACATCTGGCCATTATGAGTACCGCGTCATGCCGTA

TGGGTTGAAGAATGCTCCAGCCGTCTTCCAATCCTTCGTAGACGAGATTC

TCAGGGACCTGCACGGGCAGGGNGTGGTGGTGTATATCGATGACATTCTG

ATCTATTCCGCCACACGCGCCGCGCACGTGTCCCTGGT

>rnd-5_family-533#Unknown ( Recon Family Size = 31 Final Multiple Alignment Size = 29 )

GGGCTGGGCGATATGGCCAAAATATCATATCACGGTATTTTTCAAATTTT

TGACGTATGACGGTATTTTATGTTTTTGAATAAAAGTTCTAAATNTGCTT

TATGAGTAGTGCGTGACCCTAGGGTGGCAACA

>rnd-5_family-4231#Unknown ( Recon Family Size = 30 Final Multiple Alignment Size = 29 )

AAATGCGAGATCCCACTGATTTCGCCGTGGTTCCGGTGCTTTCTTCGATT

CCAAGCTGCTTTCAAACACCGACCTCTACTGGACACCGTACTTACAAATA

TAGTTAGGATTTTGTACANGTAGTTTCACCTGACCGGTAGCTTAGCAGGT

AGAGTAGGGAACTATGGAACATTCAGGGACGTGAGGGTGAATCCCGTTGA

AGGTACACACTATTTAGATTTTTTTTAATGTGAAACCACACTTTCTGCAC

TGTTGTTCAAATAATTTGTTTTATTTAGTATAG

>rnd-5_family-211#Unknown ( Recon Family Size = 61 Final Multiple Alignment Size = 29 )

ATCTGGCCACCGTCCACCCGGCTCCCTCGGACCGTGAGAGGGTGTCCGCC

CTCGTCTCNTGCCTCACGGGGAGAGCCCTGGAGTGGGCCAACGCCGTGTG

GGGAGGGGGACGCGGCGTTGGACCACTTCGCGGAGTTCACCCGTCGTTTC

CGGGCNGTCTTCGACCACCCGCCCGAGGGCAGAGCGGCGGGTGAGCGNCT

CTACCATCTGAGGCAGGGGACGAGGAGCGCGCAGGAGTTCGCNCTGGAGT

TCCGGACCCTGGCCGCCGGCGCGGGATGGAACGACAGGGCCCTGATCGAC

CATTACCGCTGCAGCCTGCGCGAGGACGTCCGNCGGGAGCTGGCCTGCAG

GGACACCACCCTCACCTTCGACCAGCTGGTGGACATGTCCATCCGGCTGG

ACAACCTGCTGGCTGCTCGCGGACGTTCTGGATCGGGGTCCGGTCTGTTC

CATCCTCCCGCGCCCTCCCACTCCCGTACCCATGGAGCTGGGAGGGGCGG

TGCGCAGGGAGACCGGAGGGGGTTCCCGCTCGTGCACCATCTGTGGCCGC

AGAGGTCACACTGCCGGTCGGTGCCGGGTTGGTTCCTCTGGGAATCGAGG

CAGCAGGCAGGGCGCTCNGGCGTCACCCCAGGTGAGCCGGCACCA

>rnd-5_family-522#SINE ( Recon Family Size = 33 Final Multiple Alignment Size = 29 )

AATCCGTCGGGAAGTGAGCTGGCAACCGGAGGGTTGCTGGTATCAAATCC

TAGATGCCATTGCCTGCCGTTGTGCCCTTGAGCAAGGCACTTAACCCCCC

ACAACAACAGCTCCCCGGGCGCCCAGTGTGGCAGCCCCCCGCACCTCTCC

AAAACCTGTGTATGTATGTGTNTTGTATGTGTGTCTTTCAGAGGGGTTGG

GTCAAATTTCGGTTGGACCTTGTGTGCAATTGACCAATAAAGTGATCTAA

TCT

>rnd-5_family-740#SINE/tRNA-Lys ( Recon Family Size = 42 Final Multiple Alignment Size = 29 )

TTTTAAACTATACTGCAACTCAGTGGCCTTGTGGTTAGAGTGTCCGCCCT

GAGATTGGAAGGTTGGGAGTTCGATCCCCGGCCGAGTCATACCAAAGACT

GTAAAAATGGGACCCGATGCGTCTCTGCTTGGCACTCAGCATTAAGGAGA

TAGATTGGGGGTAAGGCCCTGCGATATAGACTAGCGTCCTGTCCAGGGGG

TGTACTTGTACATCAAGCTGCCTCACGCTCCTGCTCCCATGAGCCGTTCC

GGCTCGCCAANGCTCGTGCAAGGCTACTTA

>rnd-5_family-360#LINE/L1 ( Recon Family Size = 31 Final Multiple Alignment Size = 29 )

CAATGTTTACCCATTTTTATTCCAAAATCTTTTTTTATTTCACTGGATCA

AACATTCATGCATTTTATTTGGGATGGCAAGGTACCACGGATTGGTAGAA

AACATTTACAGAAGCCTAGGTCATTGGGGGGTTTAGCTCTACCAAATTTT

CAGACATACTACTGGGCTGCAAATTTCAGAGCCGTTCTGTACTGGCTGCA

GACTGATCCTACTGGCCCTAGACCACTCTGGGTCCAGATGGAGTCTGAAT

CGTGTAAACCTGCAGCACTTTCCTCTGTGCTGTGCTCGTCTCTCCCAGTG

TCCCTAGGCAAAAGGTGTGCCAACCCAATTGTAAAGCAGTCTCTTAAAAT

TTGGAATCAGTTCCGTTTAGCCTTTAGCCTCCGAGGCTTTTCTCTATCAG

GCCCAATCAATCAGAACATTTTATTTCCTCCATCTTTGAATGATGGGGCT

TTTGGCATTTGGCACTCACTAGGCCTCTCCTCGCTAGCCCAATTATTCTT

TGATGATACATTTGCCTCTTTTTCTCAGCTGCAGGAAAAGTTCAATCTCC

CCCAATCCCACTTTTTCCGCTATCTCCAGACTAGAAACTTTGTCAGGGCT

AACACACCTGGATTTCCCAATAGGCCTGCGAATACAGCTATAGAGAGCAT

CTTGGAGCT

>rnd-5_family-3449#Unknown ( Recon Family Size = 32 Final Multiple Alignment Size = 29 )

CGTTTATTCACCCAATGGGTCAAACAGCTGAACAGACAAAGACATNTTCG

CACAAGCACTGATATTTNACTCTTCCTCCTATGCTGAGTCTCCTCCTACA

CATCTGAACAGCCAATGCATCTCTGTTGCTAGACAGAACTTTAGTGATAT

CTGTTCTTCCTCACTTCATCTGACCTGACCTCGCCCCAAATTGCTCACTC

CTCCCCAACTCACGGCTGTCATGTTGACTTGTACCAGACTGTGTCTCTTC

TCCTTCCCATAGGATCCCTGATGGCTAACAATAACATATCCTGACAGAAT

GTAAACGTTNTACATTCCCCTCTCTCCCTCAGTGACATGAATAAGTATAT

TTCATATTCTCAAGTTTAGAAGTCAGAACCC

>rnd-5_family-351#DNA/TcMar-Tc1 ( Recon Family Size = 30 Final Multiple Alignment Size = 28 )

GAGAAGGGCCTTGGTCAGGGAGGTGACCAAGAACCCGATGGTCACTCTGA

CAGAGCTCCAGAGTTCCTCTGTGGAGATGGGAGAACCTTCCAGAAAGACA

ACCATCTCTGAAGCACTCTACCAATCAGGCCTTTATGGTAGAGTGGCCAG

ACCGATGGTCACTCCGNCAGTAAAAGGCACNTGACAGCCNGCTTGGAGTT

TGCNAACAGCCANCTAAAGGANTCTCAGACCGTGAGAANCAAGATTCTCT

GGTCTGATGAAACNAAGGTTNANNTNTTTGGCCTGAATGCCAGCGTCANG

TCTGGAGGAAACCTGGCACCATCCCTACGGTGAAGCATGGTGGTGGCAGC

ATCATGCTGTGGGGATGTTTTTCAGCGGACAGNGACCCAAAGACTACTGT

AGCCAGTAAGACGACACAGGAGT

>rnd-5_family-5684#Unknown ( Recon Family Size = 29 Final Multiple Alignment Size = 28 )

TGCATGCTTCAAAATATGTACAAACGGANTACGCATTCGAGAAGTGCCCT

CCGTGCTCCGTTTCGCATACTTTGATTTGGACTCATACTCCGACCCTCCC

GTGCTCCAATTTGCGTGCTCGGAGCACGGTAGTATGCATTTTGAGAAAC

>rnd-5_family-1106#Unknown ( Recon Family Size = 37 Final Multiple Alignment Size = 28 )

AATACACCAACATTACTTTTCCGTTCAATCATAAAATGTCTAGGGTAGGC

TANATTGCCACAGTAGATTTGCCGTGGTAAACAAGGAAATACTTTATTTC

AGTTGTACGGAATGNTTGTCAAATAGATAAATCGCCCATAGTCTGTTAAA

AAGCAAGATTAAGTTGTTCCGATGATAATACCCACCAGAGGGCGAAATAG

TCTTGAAAAATCGACATCATCTTTTGGGAGCGGTAATGAGGTGACCAATA

ATGGTTGCAATTGAGATCAGCTGTGCGGGTGANTTTAGCGCGTATTGATG

GTTTAACGAGACATCAGCTGGCGATAATCTAGTGGCCATCGATGGTTGCA

ATAGGCCCCTTGTTATTTGATTTAAAAGGAATCGCGCAGAGAGTGGTGCG

AAGAATATTTTTTGGAAANNNNNNNNNNNNNNNNNNNNNNNNNNNNNNNN

NNNNNNNNNNNNNNNNNNNNNNTACATGTAANAAGAATCAAGTTAGCTAC

CTGGTAATTTTTTTCCCGTTAGCTAGTGATAGCGATGCACAAGCTAGGCC

TATTTGAAACATTGCCATCTCCTGGCNGCATTTACCCGCCAGATTCAGTA

GCTTGAAAACC

>rnd-5_family-5198#Unknown ( Recon Family Size = 42 Final Multiple Alignment Size = 28 )

TCAAAATACAACACTGCCCCTTTAAGACAAAAAAAGCTCTTTACCTGACT

CGCTTTTCAAAGATGTCTAGAAATGTACACGTTTTGTGCTCTTGTAGGAA

GCAATCACTCCCCTATTGCTGACTACAAATGATCTATAACTGGGCTAATA

ACTCACTAACTAGCAAAGGATATGAACAAATGTGCACACGTGGCTACATG

CAGCTCTCGCTTTGATCTCAAAACAAGCGCATCTACTCACGACCGCTCAT

GCTGTNNNNNNNNNNNNNNNNNNNNNNNNNNNNNNNNNNNNNNNNNNNNN

NNNNNNNNNNNNNNNNNNNNNNNNNNNNNNNNNNNNNNNNNNNNNNNNNN

NNNNTGTGTAAGGGCACGGGCTGAGTCGTGCGTGTCAATGCAATAGAATC

CTACTCCGATGCGTTCTGCCTACAACAAAATCTCTTGCATAGTTCGTTTT

GCATACTAAGTCTTGCATAGTTTGTTTTGTTTCGGTATGTTGCATTGAAA

GTGGCTAATATTGCGTTGATTCGATCACAATTCCAC

>rnd-5_family-1667#Unknown ( Recon Family Size = 35 Final Multiple Alignment Size = 28 )

TTATGCCACATATAAAAGGTAATACATTTTAAAAGTTAAATGACAAATAT

TTAGGCTATATTGAAGCGTTAGGAGGTTCTAGGTGCGAGGAATAGCCGCT

CGGATCGGAGAGGAGGCAGAGCGCGCGTTAAGCTTTCCTGAATAACTGGG

CCTGCTGGGAGCATAGGCCTATGTGGCCACATTATTATAGGACGACTTGG

GAGAATGATGATCTGCAACAGACAGCGCATCAGTATCAGAAGTAAAAATA

CAGCCAGACTGGCCTGCTACTGTGCCTTTCTAGACCTATAAACTGTCGAG

AGTAGACCCACAACTGATCCAAATGGAATGAATGAAACATTCAAATCACA

GGGCTTTTGCGCCGTTATTCAAAAGTTACGNAATTGTACTCACTTGAAAA

CAATAATGCAATTATTCATTGNATTGTGGCGTTGTAGGCTAGTCTAGGTA

GGATAATTGTATTGTCCCTAAACGAGATCAAT

>rnd-5_family-3837#Unknown ( Recon Family Size = 29 Final Multiple Alignment Size = 28 )

TTAGTATAGCTTTATCTAAAAGATTTTTATTTTTATGAAATTCACTGAGG

AGGATGGTCCTCCCCTTCCTCCTCTGAGGAGCCTCCACTGGT

>rnd-5_family-2493#Unknown ( Recon Family Size = 29 Final Multiple Alignment Size = 27 )

AGGTTGTGCCTTTAGATTTCGAGAAAATTAACAACTAAGGAAGAATTTTT

CACTTCTCTCATTGACTTCTCAAACCCCAAACCCCGGCCTGGTCTGTTTG

GTCTGTTTCGCGAGCGTTCCCGGAAGTCTCGCGATGTTGCGCCTCTGGGT

TTAGAAAC

>rnd-5_family-2576#Unknown ( Recon Family Size = 32 Final Multiple Alignment Size = 27 )

CGAATTTATGCTTGATCCGAAAATGTGGTCGGAGGCTCCGTATGGAGGGT

GTGACGCAATTGCGGAGCCTCCGGAGGCATGCGGAGGCCAAATCGAGCTC

CGTACCGCATCGCCGTGCGCCTCCCAAATTTT

>rnd-5_family-629#Unknown ( Recon Family Size = 30 Final Multiple Alignment Size = 27 )

TGTTGTTGGCTAGCTCCTCTGAACAACAGTGTCCTGAG

>rnd-5_family-1664#Unknown ( Recon Family Size = 30 Final Multiple Alignment Size = 27 )

AAAATATGGCCCTTTATAATGTCCACTTATGTACGTAGGATGCTGTATAT

AGCATTTTAACATATTAAAACAAAATAAGAGATAAATAATATTTGTCCAG

CCTTTGCTGCTATGCTTGCAGATGTGCATAATATGCTGCGACCCTAATCA

AAAAGTATTTAATAACTCCAAATAACAAAAACGTAGCTATAGGTTGTTGT

AACATTTAAACTTTTNNATTTTTTTGCACTGCATGGATCACCGGTGCGGT

TGAATGCAGCATTGCTGTATGGTGCGCTCAAAATGTATTGTAGTTGAGTA

GCCAGCCTAGGCTACTGCTCAAATGTTGCTGTAATAGGCCTACATGTTTC

TCCTTTGCATGGTGTTTGTTGCAGGTTTTGTTTTTTGGTTATTCA

>rnd-5_family-801#Unknown ( Recon Family Size = 39 Final Multiple Alignment Size = 27 )

ATGTCACAACAGGTGTAAATATATGGGTCATGAGAGTGTTATGACCATAT

TTTGACAGGTTATGACAAGTTATGTCAGCTGTTATGACATATTGTATTAT

GACCGTGTCATAACGTGTTATGACACTGGGTGTCAAGTAAAGTGTTACCG

TAATATATTATAAAAATAAAGCGAACTGGATGGAATATGGGGAAAAATGC

ACCAAATTNTTTTTCAATCTTCAACATAGAAATGCTACCAAAATAATTTA

CAGAAACTTGTTACAAATGACGGAGTCACCCATGATTCACCAAACGATAT

TTTGAAAGAGGAAGCAAAATACTTTAAGCATATGTTTTCATTTCAGTCTC

CTCCATCTCCACTAACCGAAGTTAA

>rnd-5_family-2500#Unknown ( Recon Family Size = 40 Final Multiple Alignment Size = 26 )

AAAAATGTACCTCTTCAATTTACCTCTCCAAACGACTTTAGGACCANCGA

CTGTCCGAGCCCCCCAGGAGGTATCCCGTCCACCAGANCCAGTGAGTTCG

GATCAGTCTGATTCTCTGTGCTCTGAGTCGGAGGGGGACCAGCTTGAGGT

AGGCTATACCTTCCAAAAGTGTTGAAAAGTACATATCTCCCATTTATAAC

ACCGCACTAATCCATCAAATTTTCTCACAGTAAAGTGTAACCTGTGTGTT

TGCTTGTTTACGTCTCTGTCTCCTACCCCGTTCCCTTTAACTCTGCTCCT

GTTCTCCAGGACCTAGGGGTTCTGCCCAACACCCAGACGTGGATCCACCT

GATGTCCTCCATTACCAACCACCCTCCAACTTTTCATTACATCATCTACA

GCTACTGTTATTGTCATGTTGTCATTATCTGCTAAGAAAGTTTGCTTGCC

GTATCATACTGGGCACATAATATGTGAGATACACAGATGAACTAAAAACA

CTAGCACTGCAAACACTCAGAACAAAGAGAGCAGCAGTGCCTGGACTTCG

CAGTCTCCCTCTTCAAGTCCCCCTTCCGAGACTGGCTGCCTGTTGAGAAC

AAGTGACAGGCAGAACCTCCCACCCACACAGCAACCACCTCCTCTATATT

CCACACACCAGAATTCAGTATTTTAGTCTATGATTGCCATGTGAGTGTAC

AA

>rnd-5_family-635#LINE/L1 ( Recon Family Size = 27 Final Multiple Alignment Size = 26 )

GATGATGTCCTGATATTCATCTCTAGTCCCGAGACTTCAATTACATCTCT

TATTAATATTATTGAATTATTCAGCGAATTCTCAGGCTACAAGATTAACC

TAACTAAATCAGAGGCTATGCCACTTGGTAGCCTGCACTCTGTACCTAAT

ACTTCTCCCCCCTTCCCTTTTAAATGGTCTCCCTCAGGTTTCATGTATCT

GGGTATATTTGTAACTCCTAAATTCCAGCAAATGTACAAAGCCAATTTTG

TTCCCTTGTTTGATACAATAAGACAGGATCTGGAGCGCTGGAACTCTCTC

CCGATTTCTTGGTTGGGTAGAATATCCCTCTTGAAAATGAACATTTTACC

TAGACTACTTTACCCAATCCAAATGATCCCAGTATTACTCTCCAATAAGG

TAATAAAGGATGTAAATGGATGGCTAAGTTCCTTTATATGGAGTAAACGC

AAGCCAAGACTTAAGATGGCAATATTGCAGCTGCCAAGTTCTATGGGCGG

CTTGGACCTGCCCAATATCAGGTTCTATCAGTGGTGTGCCCACCTTTGTT

ATATTTCTGACTGGATCACAAACGATGACTCCTCTATTTGGTTAGACATT

GAGACTTCTCTTTCAA

>rnd-5_family-6493#Unknown ( Recon Family Size = 31 Final Multiple Alignment Size = 26 )

GCTTAGCGATTTCGACGCGTGCCTCGGAGCTCCGGTATCAAACGTAACAT

CA

>rnd-5_family-2050#Unknown ( Recon Family Size = 29 Final Multiple Alignment Size = 26 )

CCTTGAAAAAAATTCAAGGGCCAAGGGCCACACCCTCTCANGCAATGCCA

TGCCAATTGGCCACATGGTGGCGCTATAACAAGCGATTGAANATTCAAAC

TTTGAAAGGTCACGCCCCTCGCCCCGTGNGACCTAGAGTCCTGAAATGAT

GTACAAGGAACACATGTCCCTCAAGGACCCATAAGGTCCGCCATGATGGA

TTTTCCGCCATTTNGAATTTTGTGAAAAACACTTAAAACGCTACTCCTCC

GGCACCGAATTACCGATCTGCATGAAACTTGATATGTGGCATCTACGGAC

CAAGGTCTCTCAACGCAACATTTCCAGACTAGTANCTCAAACAACACGGC

CGCTACTGACCAATAAACTTTCANGTGGGCGTGACCTGGCACATAAATGC

ATATAAATCAGACACGGATATTCGTATTGTAACAAAACTTGGTACACATG

TTNCAAACGCTGTCAAGACTCAACATNTGCAAGCACATTCANATCGACCA

CACGGTGGCGCTATAACGGGCACATGTTTATATCTCTTGATCTTTTGACT

CTGTTTGACNCAGATGAAATTTGGCACGCACGCTCAGGGATGTGAGTCTA

GCTAACCTGTAGAGTACGGTTCA

>rnd-5_family-1722#SINE? ( Recon Family Size = 27 Final Multiple Alignment Size = 26 )

AACAGAAATCCCAAGGAACCTTGTCGGTGACAGANGCTATGACTGTGTAC

ACTGGCCTAGGTGTGNCCTGACTTCAGCTTGGCTGTGCGGCCAGTGGCGC

AATGGATAACGTGTCTGATAACAGGTCAGAAGATTGAATATTTGACTCCT

GGCTGGCTCGATGCTTAATATTTANTTGATCGAAATCAAGCCGGGATTGA

TACTGTATACTTTTCAGTGCTTCTGGCTGTTCTCGCTTCAAACTCATTTT

AGACTGATTTCATCTGGACATTCCCCAACATCTCAGAGAAAAAAAGATGT

AGCTTTTAATCCAACAGGCATAATGTCAACATTTAAAGATTTGTCTGTGA

TGTGTATTTTAAGTATGTGTCTTCCCATATGGTCTAGCGGTTAGGATTCC

TGGTTTTCACCCAGGTGGCCCGGGTTCAACTCCCGGTATGGGAAAGAA

>rnd-5_family-2810#Unknown ( Recon Family Size = 28 Final Multiple Alignment Size = 26 )

CCCTCTGAAGCCCCCTCCTGCTGTTCAAAGACCATCCACTGGCATTTTCT

ACAAGAAATCTGCCTCCAGAAATAAAA

>rnd-5_family-1647#Unknown ( Recon Family Size = 29 Final Multiple Alignment Size = 26 )

ACAGAATAATACGAAATGCTCTGAGACCAGGTTGA

>rnd-5_family-152#Unknown ( Recon Family Size = 28 Final Multiple Alignment Size = 26 )

GAAGAGTTTACGCAAATATTAGCGTTGTAGCTCTTATCGCGGGACTGTGA

CTGTGTGAAATCACCTCCCCAGTCAGCCTATTGTGTGTATTGACATTCAT

ATTGCACTGTACAGCTTTACCTAAGGATTGGGGATCAATGAAATGGGGTA

TCAGTCTACTCGGTGACACCCAATATATTTTTCCCAAGTCCACCTCAGAG

TTATCAGACTCCAAAACATCCACGCAGTATTGTTTTTCCTCGGGAATAGT

GTTCAATACACATAGGTTGACAATAAATGTGTCTCAATTCACAGTTGTTT

CAGAGTCCCGCAATAAGAGCTACGACGCTAATGTTCTCTGGGTGTCACCG

AGTAGACTGATACCCCATGTCATTGATCCACAAT

>rnd-5_family-608#LINE/Rex1 ( Recon Family Size = 39 Final Multiple Alignment Size = 26 )

GGCCGCCCCCAGGTGGTAAGGGTAGGNAACAACACGTCCGCCACGCTGAT

CCTCAACACGGGGGCCCCNCAGGGGTGCGTGCTCAGTCCCCTCCTGTACT

CCCTGTTCACTCATGACTGCACGGCCAGGCACGACTCCAACACCATCATC

AAGTTTGCNGACGACACAACAGTGGTAGGCCTGATCACCGACAACGACGA

GACAGCCTATAGGGAGGAGGTCAGAGACCTGGGAGTGTGGTGCCAGGAAA

ACAACCTCNCACTCAACGTGANCAAGACAAAGGAGATGATTGTGGACTAC

AGGAAAAGGAGGACCGAGCACGCCCCCATTCTCATCGACGGGGCTGTAGC

GGAGCAGGTTGAGAGCTTCAAGTTCCTTGGCGTACACATCACCGACAAAC

TANNATGGTCCAAACACACCAAGACAGTCTGGCTCGACAAGCCTATTCCC

CCTCAGGAGACTGAAAAGCATTTGGCATGGGTCCCCAGATCCTCAAAAGG

TTCTACACCTGCACCATCGAGAGCATCCTGACCGGTTANNTCACCGCCTG

GTATGGCAACTGCTCGGCATCCGACCGCAAGGCGCTACAGAGGGTAGTGC

GTACGGCCCA

>rnd-5_family-1659#Unknown ( Recon Family Size = 30 Final Multiple Alignment Size = 26 )

AAGTCAATAAACGTTGGGTAGTTAGATAGCATATAGTTAATATACTGTAT

TAGTAGCCAACTAACGTTAGGTAGCTAGCTAACATACCGGTAACATACTG

CTGTAATGATAGCGTAGCTAGCAAATTGTCAGCCAACATAACGTGTAAGN

TAACTTATTTGAAAAGTCATTACTTTATTACATTGCTCAACATTTTCTTA

ACATTTGTCATAATTAGTTAAAGCAATGAATTTGTATCCGCTCTCGTCGG

ACTTCGGCTGCATATTTTCCGCCATTTTCTTCAAATCTGAAAACGATGTG

AAGCCACGCCCATTTTCTGAAGAATTGCATTATGGGCCCTAAAGTACGGA

AATAGTGTCCTCTGCGTGTATACTTCGTATTTTGGCGAATTTAGTACGAC

ATCCGGGAACTTTTGGCATACTAACTATATCCATACTATGACCAATAAGC

ATACTATATACTCAATTCACGTCACAAATAGTACGGTTAGTGCGGTAGTA

TTCAAA

>rnd-5_family-1496#DNA/TcMar-Tc1 ( Recon Family Size = 27 Final Multiple Alignment Size = 26 )

GTGGAGGAGGCCGTAGGAGGGCAACAACCCAGCAGCAGGACCGCTACCTC

CGCCTTTGTGCAAGGAGGAGCAGGAGGAGCACTGCCAGAGCCCTGCAAAA

TGACCTCCAGCAGGCCACAAATGTGCATGTGTCTGCTCAAACGGTCAGAA

ACAGACTCCATGAGGGTGGTATGAGGGCCCGACGTCCACAGGTGGGGGTT

GTGCTTACAGCCCAACACCGTGCAGGACGTTTGGCATTTGCCAGAGAACA

>rnd-5_family-403#Unknown ( Recon Family Size = 35 Final Multiple Alignment Size = 26 )

GCAAGGAGATTTGGAGAATATCAGGGAGTGGGTTTGCCGAAACAAACTTG

TTTTAAACACCAAGAAAACCAAAGTTATGTTGGTCTGTTCCACTAGGAAA

AGGCCAAAACAGCATGGGATACAATTAAGTATGGGAGGAGTACAAATTGA

AGAAGTGGCAGAAACCAAACTACTGGGAGTGCAGCTAGACAACTGCTTAT

CATGGTCGTCTCAAATAACTAATCTATGTAAAAAAAAATATTAAAACAGC

ATGCATAATCAGAAGGATAGCTAAATATTTACCAGGAAAAATTCTTCAGC

AAATAACACAAGCATTAATTGAGAGTCAGGTGAACTACTGTTCTGTGGTC

TGGGGAAATGCATCATCAAGTGAAGTTAGGAGGCTGCAGAATGCACAGAA

CAAAGCAGCAAGGATTGTTTTAAGGTGGAGATATGGTTCTTCTGTTGCAG

TCATGCGCAGTGTTCTTGGTTGGTCATCAATCGACAAGATAATTGAAAAA

AACATGCTTATTTTATTTCATAATATACATCATTTAAAACGGCCAAGTTC

TATTCACAACGGTATTCAGTTGGTAAGAGACAGACATTCCGTAAATACTA

GGAATAGATTGTCCACCATCTATGCGTTATCCAGACAGAAAAAAGAAATA

GGCAAAAGAACATTTCGATTTA

>rnd-5_family-2532#rRNA ( Recon Family Size = 26 Final Multiple Alignment Size = 25 )

AAATATCGGGGTGAACGCGCTTCGGGGGTCGTANCTATGACTCTCTTAAG

GTAGCCAAATGCCTCGTCATCTNNTTAGTGACGCGCATGAATGGATGAAC

GAGATTCCCACTGTCCCTACCTACTATCTAGCGAAACCACAGCCAAGGGA

ACGGGCTTGGCGGAATCAGCGGGGAAAGAAGACCCTGTTGAGCTTGACTC

TAGTCTGGCACTGTGAAGAGACATGAGAGGTGTAGAATAAGTGGGAGGCC

CCGGCCGCCGGTGAAATACCACTACTCTTATCGTTTTTTCACTTACCCGG

TGAGGCGGGGAGGCGAGCCCCGAGCGGGCTCTCGCTTCTGGTTTCAAGCA

CCCGGCTCGCGTCGGGTGCGACCCGCTCCGGGGACAGTGGCAGGTGGGGA

GTTTGACTGGGGCGGTACACCTGTCAAACGGTAACGCAGGTGTCCTAAGG

CGAGCTCAGGGAGGACAGAAACCTCCCGTGGAGCAGAAGGGCAAAAGCTC

GCTTGATCTTGATTTTCAGTATGAATACAGACCGTGAAAGCGGGGCCTCA

CGATCCTTCTGACTTTTTGGG

>rnd-5_family-365#Unknown ( Recon Family Size = 32 Final Multiple Alignment Size = 25 )

TATGAAAATNTATATAAATCAGAATTAAAAACAGTTGGACGGATCCTATA

GCCTTAGACTCAACAACCCTGAAGCAACTATCAGCAGACAAAANAATCAC

TAAAAACTGAGATCACCCAAGAATAAATATTGGATACTGTTAAAACATTC

GCACGGAGAAAAGCACAATTTTGGGACAAAGTAGCCNCCTATTTACACAA

ATGACAACACGCGTCGCTCAATTCAGTGCTTCCTAGTTCCATGTATCAAA

CAGTTATTTCAGTTATATTAAAACCAGGAAAATCTGGAGAGTCCCCTGCT

GATTATAGACCCATAAGTTTAATAAATTGTGACAATAAAATAATAACAAA

GCTCATAAGCAATAGAATGGCAAAAGTCTTACCAGACTTGATACATATCA

ATCAAACAGGGTTTATTAAAAATAGACACTTACAAACAAATACAAGAACA

TGTTTAATACAATACGCAAAAAAACAGATANATATTTATCAATAATGGCT

GTTGATGCCGAAAAGGCTTTCGACCGTCTTGAATGGCCTTTTCTATTCAA

AACTTTGGAAGCTTTCAACTTTCCAGCT

>rnd-5_family-2534#Unknown ( Recon Family Size = 28 Final Multiple Alignment Size = 25 )

TGTCATGTTTTGGCTGATAAATGTACTAAAATGGGAATAAAATTNANATT

TCAACTTTCAAATGGTACCACAAAGATGGTTGGAGGTCCACACATCGGAG

AATGTTGACTTGAATGGGAATATCGTTGTTTTAAATTATACTGTCAATCC

TCCATAGGAAACCTATTGAAATCATAGAAATATAGATAATAGAATAGACA

TGACCATTTAAGTTGACATTCGACGGTGGGTGGACCGGCGGCCATCTTTG

TGGTAGTAATTAGAAGTTAANATTTCAATTCAATTNAAATTTCAATGGTG

TACCAGCTAAATTGCAGTGGTCTGAAGGGATAGGTCCA

>rnd-5_family-1806#Unknown ( Recon Family Size = 31 Final Multiple Alignment Size = 25 )

ACNCCCCTNCGTATTTATTTGGACAGTGAAGCTAAAACTTNTAATTTNTG

CTCTATACTCCAGCATTTTGGATTTGAGATCAAATGTTTCATATGGCGAC

AGTACAGAATGCCACCTTTAATTTGCAGNTTAGCATTTTGTGTCATGNNN

NNNNNNNNNNNNNNNNNNNNNNNNNNNNNNNNNNNNNNNNNNNNNNNNNN

NNNNNNNNNNNNNNNNNNNNNNAATCAAGCTTGTGACT

>rnd-5_family-1796#Unknown ( Recon Family Size = 30 Final Multiple Alignment Size = 25 )

TTGCGTTCCTCTGCCCAGGCGTTGCTGACTCATGCCAGATCGATCTTACA

ACGCCTGGATAGGTATTTTACGTATCAGCTAACCACATCATACGTTATAG

CAATCTGGTGCCCGACTGCACAGTCGATGACGTGGCACGCAACCATTGGT

TGATGCATGCAACGTCTGAGCAGGGGAACGCGACCTA

>rnd-5_family-7034#Unknown ( Recon Family Size = 27 Final Multiple Alignment Size = 25 )

GCCGCGTTCACGTGCTAGTCGGAACTAGGAAACTCGGAAATTTCCGACTG

GTTGAACGCGGCACGTGTATAACTACAACCAGTTAGCAAGTCGGACATTT

CCGAGTTTCCTAGTTCCGACTAGCACGTGAACGCGGC

>rnd-5_family-1477#Unknown ( Recon Family Size = 26 Final Multiple Alignment Size = 25 )

TGGCTGGTAGCTTATTCTTTAGATGTTTGGGGCTGCTGGTGAACCATGAT

GTGCAGGCATAATCAAAGTGACACTGGACTAGCGCACTTGCCAGAGTCTT

TAGGGTGTCTAT

>rnd-5_family-833#Unknown ( Recon Family Size = 27 Final Multiple Alignment Size = 25 )

AGAGATTTGCCCTAAAGAGCATTGTCACATCTGATGACAACATCACAAAT

GCTGAGTTTAAGGAGACAGTTCCACTTCCTTGCTTAGACATGAAATATAG

GCCCAGTAAGAGTGAGCCTCAGTTGCCAGAGTCCACTGGTGAAGTTACTT

TACAAAGAGGCCTAAGTGAGAGCTCAAAACTTCTTTGGGCAAAAACTGAG

TCAGAAGAAAGCAAGCTCCTTCTGACAACTTGCACCAAAGAAGTCGTCTC

AGAGCTTCTGGTCTTGTACAACACTGAGATGTCAAAGGAGGACCCCCTGT

ACACTGTTGGAGAAAAAGGATCAGGCTTCTCTATTGAGAGAGAGAAATTT

GTAGAGGGCGTTCTGGCTCAGCTTGGGGATATCGCCCATTCCAGGGCCTC

ATCACCAATAGTATGTGAGTTCCCCTGTCAAATTGAGGACAGCAGAAGTA

AGGCCACAGCTTCTTTGACCAGTCTGTTAGATTGTATTAGAAAACTCTCA

AGTGAGGAATTTAAGAGAAACGCCACTCAAGCAGTGAGTGAGTTCCTAGT

TAAAAAGTCCACCAGTAGCTTAACTAGTGCACAGCCTGCTTATTCTGTAG

CATCCAGGTCTTGTTCCGTTCAAAACCAGTACACATGGTCAAAGGATATT

TGTGCGGATTCTGCTGCCTTTGGCAT

>rnd-5_family-466#Unknown ( Recon Family Size = 31 Final Multiple Alignment Size = 25 )

TAGGAAGACGAGAGTTCTAGAAGCTAGAGAGTTCTAGAGAGTTTTAGGAT

TCTATAGAAAGAAAAANTTGAAAAAATAATACGATTGTATATTATTATTT

AGAAATGGTGTTTTTGTCTTGTTTTTAATTGTTGACAGCCAGTAGACGCC

ATGTTTATATTGGCGAAGCATTGTAGTCGATTATAATGTGAAACGGAAGT

TGTTTTCTGTTGGTGGCGAGCAAACTTGTCCAACAAAAATATAGGAAATA

TTTGTTGTCTTACGGGGGAAGGTGTTACAGGCTTTGGTTTTTCCATTTAT

GTTTTGAGGTTGGACTTTAGCACGTAGGACGCACTGATTGGTGTCACCTC

GTTAGTCAGTATGTGTTACACCTGTGCTGGCTTGTCCATCTCGTTAGTGG

GAAGGTGTTTCACCTGAGCTGGTCCAGGTTCTATTTAAGAGTGNCTGGCC

CAGTGCTCCAGTTGTCTTGATAGATGTGGAGGGTCAACACCTTTAGTTGC

TCCACCTTTTTGGTTTGCTTCCTGTCTTTAAGTTTGGCGTGGGTTTTTCT

TTTGTTTGCCTCTTCTTGGGCAAATTTAGTGGGCGCTCATGGTGGGTCTC

TTTAGGTCCCAGTTGTTGTTGCTAGTCAACTTTCAGCGGACACCCCCATG

AGTGTCTTTCAGAACCCCTCCTACA

>rnd-5_family-1592#Unknown ( Recon Family Size = 46 Final Multiple Alignment Size = 25 )

ATGATGAACTTCAACTTAAATACTGGATCTTGCTGTCGGACAGTTTTTTT

GTATTCAGATCTCTGAAATGCNCTCTAACTACGTAACATTTAGTGTCTCC

TTATGTTACGCTGGGAAAACAGTTTTCATGGGCACAGAAATCCTAAATNA

TTTCAGAGTTTGCTAAATCCAATGGTTCTAACCGAGAGTCACCTTAACTT

TATTGACAACACCCAAATGGATACTGTCATTAACGCGGTTCTTCAATAAT

TACGCATAATTCTTAATACTGTAGCTGTTTAGTTACAGTCACATGTTCAA

CTATCATCAGCTGATCCAGGAAATCATTTTCTGAATGACAAACATCACGA

CATGCAACAACAACCAAAGTGCTTCTTCATTCATTACTCTGGTAAAGACA

GTTATGCCGTGCTCCACGTTGGATCCAACATCCCTAACAAATTGATGTTT

ATAATGTGTTATTGTCTGCTTGATTTACAGTAGGGTCTCGTTAGTCTGTT

TGGACACAGAGATACTTTGCTCATAATGTGTAGAGAACTGTTCCTTGATG

GATAGGCTATTGTACAACACACAGAGCTATTTTCCTTTATTCAGGACATT

TAGAATGACAACACATTACAGAGTAGCCTAGTGGGGTTATTTCACANAAA

TNATCTG

>rnd-5_family-587#Unknown ( Recon Family Size = 27 Final Multiple Alignment Size = 25 )

TAAGGGCACAAGGCGAGACCCAGATGCAGACACGGGAGGCAGATGGTTGG

AGTCTCCGATATTTATTATAATCCAAAGGGGTAGGCAAGAGAATGGTCG

>rnd-5_family-75#Unknown ( Recon Family Size = 29 Final Multiple Alignment Size = 25 )

TGGCTAGTATCAAGAGGTTCGGACTTGCCTATTTACATAAAGGTTAAAGA

AAAAGATAAAATCACACCTTAAACTATTTTTGTTTCCTCCTTATCATGTA

ATCACAAGGCAAGCCAGCAAGGTGCAAAGCAAACATACACAAGAGGTTCA

AAGGGTCCTTGTTTTATTCTTAACTCCATTGTAAACAGGCTTCCCACAGC

ACTACTTTCATTTATCTGCNTGCCCAGCCCTTATATTTAGCCTCACAATG

AAGTGTTTTACCATTTTCTTTTCAGGACAACCAGGGCTACAAGTAGAACA

CAAAACAATTTGACATAAACAGTTGCATTCATGAGTTTTATTGACAAATG

TCAATAAAACCGAAAAACCCGATCGCCAAATAAAAGCATTTATATGAATG

CTGTAAGTACAGAAATTGTTTGCTCAGTGGGAAATTAATATCCAACTAGT

CAGTGACAACTGTGTGGAGTTTGTGACAGCAACTATGTGAGCTTATTACA

GTATTATAGACACTATGTCCTGGGATTTCCTATGATCTTCTATGTAGAAG

AACCC

>rnd-5_family-1364#Unknown ( Recon Family Size = 49 Final Multiple Alignment Size = 25 )

ATACTGAACAAAAATATAAACGCAACAATTTCAAAGATTTTACTGAGTTA

CAGTTCATATAAGGAAATCAGTCAATTGAGGCCTTAATACATCCTAATTA

GTG

>rnd-5_family-3277#Unknown ( Recon Family Size = 27 Final Multiple Alignment Size = 25 )

TGTGAGAAATCTTCGAAAAAGAACAGGAATTTCGAATTAATACGAAAAGC

GTATACTAAAATATGAAAATACTACATGTCATGTCTCAAAATCGATATCT

ATCTTACCTCTATATTGTTTTATGTCCTGCGGATTCGAGAAGAAAGATGA

CGAGTTCAACGGGAAAGTGAGCCTGTCAGTTAGCCTTCATTCTCGCACAG

CATCCAGCCTAGCTGAAGTGACGCAATGCNATTTTCCTGATTTTTGACCA

CTTTTTCTCTCTGGCCTCCATTGATTTAGTCACCCTAATTTTGGCCATCC

TACAAGGGTAAAAACTCCAATAACTTTTGAACGGATTATGATAGAGACAT

GAGGTTTGGACCATTGGTTTTCTTAGAGGATTATCTACACAATTAACATA

TTATTTTACCCATTGGTCAAAATATGTTTTTTATTGGACACCGT

>rnd-5_family-405#Simple_repeat ( Recon Family Size = 29 Final Multiple Alignment Size = 24 )

ACAAAACCTTTTGTATGACCTCTTATACACTATNTTAGGCCCAGCCTTTG

GAACTTTGGTTTTCC

>rnd-5_family-4179#Unknown ( Recon Family Size = 27 Final Multiple Alignment Size = 24 )

CGCTAGGTNTTGGGTTANNGTCGCTNCNTTACTCAAAACCNTCGNGGGAG

CGTGTCCCCACGCTTCTCTATACCGANNCCCTCACGTGTANACGCCTCTC

TGATGGACGCCATCCCACTTCTGACACCAATGTAGCGAATTCGGGGGGTA

GCCCCGACCGGGACTCGAACCCGGGTCCAGCGACTGTCAAGCCAACACCT

TAACCGTTACGCCAAGAGGTCCGAACCTCTTGACGAGGTCGCTA

>rnd-5_family-256#LINE/L2 ( Recon Family Size = 27 Final Multiple Alignment Size = 24 )

CTTTCACNCTGCATTGGTATCAGAAGTCTGATGGCACTATGGTGTGATAA

GAGCGTCTGCTAAATAACTAAATATAAATGTACATGTAAAAATGAACAAT

TGCAAAGCATGCTGAGTATTATTCTAATGAGCTCCGCCCCGAAACAAGGC

CAATAATAATACCCAGTGTGCTTTGCAGTTCATTTTGGTATGTTCATTTT

CACATATACGTTTACATGTTGGTCATTTAGCAGACACTCTTACGGTCGGT

GCATTAAACTAAAGGTAGATAAACAACAAATAGTCATAGCAAGTAAAACG

GTTTCTGTTCAGAACCGTTACTGAGAATGTTGTTGCTGTTCGGGCCGGCT

ACTTTTAA

>rnd-5_family-4251#Unknown ( Recon Family Size = 31 Final Multiple Alignment Size = 24 )

AAATTCAGCTCACTTCGAGCAGAGTTTGTTGATTCAACGTACAATTGTAA

GGCAACCAGCTGCAATAGGATTGTGAGTTTGCTCAACATTTGGGCATATA

GCTGAACCGACGTACAGTGTTGCCTTCGAAAGGCAAACATNAATTTTGTT

TTTATTACATTCAGCTAGCTCACTNCGAGCAGAGTTTGTTGAGTGAACAA

ACGTTNACACATTAGCTAAATGTCTCGTCCTTTTGAAGTCCACTCAACTC

ACAGAATAACGCTGATTAAGCAATTGGTTAAGTTGTCTTTTTT

>rnd-5_family-3308#LINE/I ( Recon Family Size = 25 Final Multiple Alignment Size = 24 )

GTATATATTTTGAAAATTAGTGAGTGTAGTGTTAGATGGTAGGGTATTTG

TTTAGTACTTTTTTATTTTTCAAGCAAAGTATAAGGGAGTTGTACTCCAG

TCTAGTAGGTGGCGGTAATGCAACATTTATTGGATGCCAACCGCCGTTAA

ACCTCATCGAAGAAGAA

>rnd-5_family-7749#Unknown ( Recon Family Size = 27 Final Multiple Alignment Size = 24 )

AGTTTATTAGGATCCCCATTAGCTACTGCACATGCAGCAGCTACTCTTCC

TGGGGTCCACATAAAACGTACAAATACATGACAAAGTACAGAACAGTNAT

AGACAAGAACAACATAAGATATAACATTAAATTCAAAATAAAGTTGTATA

TATGGGAAGGACACCAAGACACAACAAAAATACTATTTACACACTATTCA

TATATAATATTCATATATCCAGTACAGTTACATTTGATCTTTAGAAAGAG

GAGAGGCGCTGCGATACAGTAATGGG

>rnd-5_family-966#Unknown ( Recon Family Size = 28 Final Multiple Alignment Size = 24 )

GACCAATCCCCGAGGTTTGTAAGACCCTGGGTTTAATAATAATTAGGCAA

AGACTCAGCTTCTGCAAAAGGTTCGTACGNTTTATTCACGAGAACGTTCT

AAAGTCCACCAAAACGTGAGCAGTTTTTATAACTCGCCCCCTACTTACGC

ACATACATACACACAAACAGTAGGTGGGCTACGCACGTACATACACACGA

ACAGTAGGTGGGCTNTATCCACCGTTTATCACCACCGAGCCGGCAGTTCC

ATCCTCCCNAGATTAGAGGGACCTTGACAGGGCTCTCTTTCCTGTCGTAA

GTTTCTCAGAGTTCCAACCAGGTCAGGTCATGCATAGTTTAATTGTCCTC

TGTATTTTCTTAGTCACACACACACATTTCTCTCCCTTACTTGTCTCGAC

CGAACCTTATTGGACTTACGTTTAGTACTTTAATTATTCATTATACATGC

TACATAAACTAATAATCACTAATAGTTACGTTTCAGGGTGGAATTCTTTA

GTCATTACCTTAAACATATAAATTCCCTTATCAGT

>rnd-5_family-1020#Unknown ( Recon Family Size = 25 Final Multiple Alignment Size = 24 )

CAGTGCTTAATTTGAGCCGGAACAGGATCCCGCACCTCTCAGTTTTGGAC

TGTTTCGTTCCGGAACCCATTTGGCCGGATCCGGTACCTCTCGTGGCGTG

AAAAACANTTTTCACTTTTTGCAACGTAAAAATTNTAATAAAAGCGATCA

GAGTTCATTCGAGTTGCCTCNTCTGTTAATTCTCCTGCACGCCCCAAAAA

AAACGTAATGTGAAAAAGTNGATTTTCAGCCCGGGCCTGATATTCTAAGA

GTTGATTCGGGTTGGGCCGGCCCGGGCTTATGGTTTGCGCAACATTGTAA

CCTATGTTTGAGACCAACGCGTGGCCGTGGCTGTGTGAGAAGCGCGCCTG

TATCTCTCACATAACTAGAATGAGATTCACTTTCTATGATCTCTCTCCCT

CTCTGCTCTGATAGACATGAGCCTGCAACTCTCATCTCTCCAGCGTTGCA

CTTNATCATTTATTTCCT

>rnd-5_family-2298#Unknown ( Recon Family Size = 28 Final Multiple Alignment Size = 24 )

CATCATCTCCTTTTCTCTAAGGTTCTTTAATTGCATCAGCTCTTTGGCGC

GTTTAATGGCTACCACTCTGACTTTATATTTGAAAAATTCCCATCTACTT

CCATGACCCAAGTCTTTTCTTGCAAAAATATCTTTAGCTAATGATTTGAT

GTTTTCAATGAGAGCAGTATCTTTAAGAAGTGTGTTGTTTAATTTCCAAT

ATCCTCGAGTACCTTTTGATTTTTTAGTAGCTTGTAAATTCAGGGAAATC

AAGTGATGATCAGAAAAGGGAGCCAACTGATGATCTACATCTATAACAAA

TTGTAACAAAAAAGGGGAAATTAGGAATAAATCTATTCTAGATTTACGTG

ACATAGTTTTGTTGGTCCAGGTATAACCCTTAGCATCCGGATTGAAATAA

CGCCAGGCATCCTCAACACAGAGATCCTTGCATAATGTAATGATAATGTT

ACTATTTTGAGGGTTTTGACTCGTTCTAGGAGGAAAACGATCAGCAGATG

CATCAGGTGTTTCATTAAAATCCCCTGAAATAATTAGAAAAGCCTCTGAG

TATTTGTTGCTTAAATCCTGTACTTTCCTGGTAAATTGGG

>rnd-5_family-1344#Unknown ( Recon Family Size = 25 Final Multiple Alignment Size = 24 )

TTGTGGCAGACCAGGGGGTTCGGTCAAGACGTTTACACAGATCAGACACG

GACAGAGTAGGATTAGCTCGGTTTCAAGGGTGTTTATTTAAATAATAAAT

CAAAAGAAAAGAATAGGTCTCCCCCATGAGACCCTCTCCGGGATACCGTC

TTCTGGGCTCCGGGTCTTGCTGTATCCTGTCGGGCACACAAAACTGAACT

CCCTCCTGTTACCTCTGNNACCGTCCCCAACTATACGGGAGTCACCTTTC

CTCCCCCAGTCTCCCCTGTGTGCTGCCCTTCTGGCAGCTTTATGGGACTC

GCGCAGCTGGTGAGCAATCAGCCCTTGATTACTCACCAACTCCCAATCAG

CCCCAATTAGTCCTGGCCGGAGAGCCCGTCGAGACCTGGCACGTCCAGCA

GATGGAGCCATCGCCTCGTGATGTATACTCCGTCTGTCACCAGGCCTCGA

CGAGTCTCCCCCTGGTGGCTGACCTGCTGTACGCC

>rnd-5_family-750#LTR/Gypsy ( Recon Family Size = 25 Final Multiple Alignment Size = 24 )

AGCACCACATCTGCAGCTGTGATACACAAGCTGAAAGCAGCCTTTGCCAG

GCATGGCATTGTAGAGACTTTAATATCTGACAATGGGCCCTGTTACAAAT

CAAATGAGTTTGAATCCTTCACAAAAGCATGGGAGTTCACACATGTCACC

ACAAGCCCACATTACCCTCAAAGTAATGGCCTTGCTGAAAAATCTGTGCA

GATTGCTAAATCACTCATGGACAAAGCAAAAGCAGACAAGAGAGACCCCT

ACCTCAGTCTCCTTGAATACCGCAACACTCCAGTTGACAACTTCAAATCA

CCAGCCCAGCTGTTGATGAGCCGCAGACTTCGCTCAATCCTTCCCAGCAC

CAACCAGCAGCTGCAACCTGAGGTCGTCAGCTACAAGGAAATGCATGAAA

AACGTGCACGGAGACAACAACAACAAAAGCGATACTACGACAGGTCAGCT

AGACCACTGCCACCACTGATCGACGGAGAGTCAGTTAGAATCCAGGAGCA

TGGCCTCTGGAAGCCAGCAGTCGTCATCCAGCCAGCTGACACTGAACGTT

CATATCACGTCCGCACCGCAGAAGGAGCGGTGTACCGCCGCAATCGTCGT

CACCTACTGAACACAAAAGAACAACACACTGAT

>rnd-5_family-545#Unknown ( Recon Family Size = 30 Final Multiple Alignment Size = 24 )

TTATAAAAGGTGTCGAACTGACTGAATAANGTCGATCTCGTATATCCTTG

CCATTCATAAATCATACAACNTAGTTATATGTCCATGGTATCGCATCGGG

ACAAGTAAACTAAAGATCTCGTTTGTATTTTCGATATGAAGTCCATCAGG

ACTCGCCAGACAGTGACGTTAAAGTGAGTGACTCGTCAGTAGCCTACCGA

ATCGCGTCGGTAAGAGNGCTGTTCGTTTGNCTCCCTAATTTGCATACTGG

TAGTCTTTTNGGCGATTATCTGCAGATANATTATGAAAACATTCGATGAA

GATGGTGAATCCTCCTCACTGCAGGAACAATAGGTAGGCTAGTGGAGAGA

GCCTCACTCTGGTCCGAAATATGATAAAAGAAAACGGATTGAATTGTTTT

AAAAGCTAGTGATACTTATGGTATTTTCAAAGATATTGATCTAGGTCTAC

TGATTTAATCAAAGTGTTGACAATGGAGTAGTCAACTGCTGCTTTCTGTG

TAGCAAAGCCCATGTTTAACACCTGCTTCATTCTTCAATCATACCTGTAT

TGCAGATAGCTGAGAAAATGCCTCTTAAAAG

>rnd-5_family-2965#DNA/Tc1 ( Recon Family Size = 28 Final Multiple Alignment Size = 24 )

TTATTGTGGGAAGCTTGTGGAAGGCTACCCGAAACG

>rnd-5_family-2302#SINE? ( Recon Family Size = 24 Final Multiple Alignment Size = 23 )

TGGATGAGAGCACAAATGGCCAGCGATCTAGCGTCAGTATTAAATACACC

ACAAGTCTCTGTGGCGCAATTGGTTAGCGCGTTAGACTGTTAATCTAAAG

GTTGGTGGTTCGAGCCCACCCAGGGACGACAGTTTTCAGTTTGTTGTGCT

CCATGATGTCGATGTGACGTCCTGTACCTGTTATGAGATTCCTGTTAAAT

TGCCGTGCTGAGGGTCAATGCCAACACAAGCCTCTCTCGTGCAATCAGTT

AGCGTGTTTGACTGTCAATCTAAAGGTTGGTGCTTCGACACCACCCAGGG

ATGACATTTTTCAGATTGTGGAGTCCCCCTTCCTGACTTGGCGTGAGCTT

CCATGAGACTTTGGCCTGCTGAGGGTCAGTATAACCACAAGTCTCTGTGG

TGCAATTTGTTGGCGCGTTAGACTGTTCATTTAAAAGTTGGTGATTTGAG

CCCACCCAGGGATGCTGTCTTTCTATTTCTAGTGATATGGAAGACCTTCC

TGGCCTTACAG

>rnd-5_family-1988#Unknown ( Recon Family Size = 29 Final Multiple Alignment Size = 23 )

TCTGGGATTTAACGCATCGTCTCGACACTTACATCACAAGAAAGAGATGA

AAGATAACTTTATTTCGATGGGTGTTCATTTTTTTGTGGAATTGAAAAAA

GTAATNATTTAATACAGTTGAAATGTTTACAAATTAGATGCGCTGAAATG

AAAGCAACTTCCGAAAATGAACACTCGGATTATAGTGATATTATGTCTGA

TCTCGCAAACAATGTAAAGTATGTATAGATAGGTGTAATCTAGAGCCAAG

CGTGTTGATTTTTATTATCCGAGGGTATCCTGCTATTGACACACTTGTTG

AATTATGCAACAGAACGTGACAACAACAGTAATTAATGAGGCAGTCTAGA

CAGCGCAGGTGAGTGCAGGTAGGCCTAGACAGCGCAGGCGAGTGCAGGTA

GGCCTAGACAGAGCAAGTNTTTGGTGGTTGCAGCCCCGTTCCACCCCTTT

ACGTTAATTNATTAATATATGCAAATACACCCTATGAAAAAAAGTGATTT

AAAGGTTTTTAAAAACCTTTTAACA

>rnd-5_family-883#LINE/Jockey ( Recon Family Size = 29 Final Multiple Alignment Size = 23 )

GATGAGACCACAGGGCCTCATTTACACCCTATCCGATTAACCAATGCCGT

AGAGAAAGAGGTAGGTGAAGTNAAACTAGCNCGGTTCATTGGAAATGGTA

GATTGTTAATATTNTGTGNTAGCCAGGCTCAGCAAGGGAAGATTCTTCAA

ATGGAAAANCNTAATGGGAAGAAGATCAAAAGCCATGTCCCTGGNGCTTA

TGCTAAATTGAAGGGAGTCATNACTGGGGTCCCAATATCTATGTCCATAG

ATGATATTAAAGACGATGTGAAGGGAGNCAGAGTGATTGAGGCCAAAAGG

TTGATCAGTAGGAAAGAGGGTCAAAGAAGTGAAAGCTTATCAGTGATGTT

TTGCCTGGNAAAGTACAAATAGGATTCCTTAGTTTCAATGTCAGAGAATT

TGTCCCATCCGCATCGCGGTGTTTTAAATGCCAAAGAATGGGACATGTAG

CTGCTCAGTGTAAAGGAAAGAAAATATGTGCCAAGTGTGGAGGGGAACAT

GATTACGGTGAATGTGGGAGCAATGTGAAGGTTAAGTGCTGTAATTGTGG

GGGGGAACGCAGTGCGGCATTTGGTGGATGCCAGGTGCAGAGAGAGGTTA

GAGAGCAGTCGTGAT

>rnd-5_family-408#Unknown ( Recon Family Size = 32 Final Multiple Alignment Size = 23 )

TGTAGTGGGTAGTAATATTTGATATGTGTATATACAGTATATTATATATA

CCTCACATGCGACTCGTAATAAGACTATGCAATTAGCCTATTAAAATGTT

TATCTGACTCCATAAAGATAATTAAAATATGCAAGCAAGTATTAGGCAAT

GAGTTGACGTTGACTAGCAAGAATANGTCTGAGAATGGTCTANATCAAAG

GCAGATATTTAATAAACATTGTAAAATGAATGGATTCACATACAAGGCAT

AAAGCTTAAGTTACAAAGCATTAACAAGCATTACAAGGCATACTT

>rnd-5_family-714#Unknown ( Recon Family Size = 55 Final Multiple Alignment Size = 23 )

TACTTTATTCTGCACTGCTATTTACAGCTGGATANTTGCTATTGANAAGA

GCTAGGTAAATGAGTAATCCGTTTGGATAAGGGGATTGTAAATATAAATG

ACANTTGTTTTTAGATATATTTTACCTCANTATCGCTGGAGACGAATGTG

AAACCAGTCATATGGCAGCAAACTGAAGCATATCAACATATCAGCTTTCC

CACAGTGAAACGAAAAGAAAAGGTGACTATGCAGAATTTAAATTGTACTG

CCTTTAAATTTGCAGGGATGGGCACTCTCTAATGTCTTAATTATAGGCTA

CCCCGACTTTCTCCGTTTCCTATTTCTATCATGTTAAATATTAGTCACTA

TCAGTATCGACCGCCAGTAGGCCTAATAACCACACATATTCAACTGCCAA

TTTACTGTTAGTTCCCTTCAGTTGGTGTAGCCTACATTATCATTCCATTT

AATTACATCGTTTCGTTTACCTTCATTTGCTTCCTCTGTAAACGCCGTCA

CGGCCGTGTGGTTGTTGCTGAGGATCATTAGTAACAGTTGATAATATAAA

AAACAAAGACACGTAGGCTAATTAAATCGTTATGGAGCGGAGCGCAGACC

AAGCCGTAACAGTTTGAACCCGACGCATGGCGCAATACTTGGACCGTGTC

>rnd-5_family-135#Unknown ( Recon Family Size = 25 Final Multiple Alignment Size = 23 )

AATAGGTCAGTAGCCTACAGAGTAATATGAGAAGAATGCAATTGCTGAAT

TTATGTAGATATTCTAAACTAAGTGTTTTGATAACTTTCAAATGTAGTAA

CAGGTCCATGTAGAATAAACAACCCTTTACATAATACCGTAGAAGCAGTG

TTCTGCTAATATAACAATGTGCACATTTCACCTTTCATTGTCATTCCCAG

CCGATACAGATACTGTGCCTATCGGCATTTTGTCTGGTGGTAATGGGGCT

ACCTTGGCAAACATGTCAGAGTGGTCATACCTTCCTGTGTGGTGTCCCGT

ATACAACAGGAGTTCCCTGATCCTGGTGCAGAATACACAGGGTTTCTCCC

TCCCCATATTGACTGATACCATTCAATAATAATAAAAAAGACAATACAAG

TAAAAGTTGTGTCTAGTAAAATTTTAATGCCGAGTGCCAGGTCTCTCTCC

ATTCAGAGACATCAGTACCAAGCCCGTCTGTCAGTCTGGACTTCATCACA

TCGTCTTGCAAGTCTCCATGTGCTGGCTCCATACATGTTTCCGTGAACAC

ACACACACAGTCACTGTTCTATTACCAATGGCAGTTAGGATAGGTAATAT

CTGACACACAAACAGGTACTATGTTGAAGTTTNAACAGGT

>rnd-5_family-1953#Unknown ( Recon Family Size = 26 Final Multiple Alignment Size = 23 )

TCCTTGTATGTTTATTGGTAGCACCTGTGAATGTTTAATTAGTTTGTCTT

TATTAGACAGCCGGCCCGCCTGGTTGTTGTGCGGGATTATTTCTATGTAA

ACCTTCGGCTCTGTGGTANAGGCACGTGTTAGTGCCTGGTCGGGATTGTT

TCCCGTTGTACATTTTGATTCCCTGTGTTTTGGGAACGTAACGTTTTTGT

GAGCACCCTGTGGTGCGTTGGTGCGATTAAAAGACGCGCAGCATTGAACT

CTCTGTCTCCTGCGTTTGACTCCACACCCACGACACCCGGAGCATTA

>rnd-5_family-2037#Unknown ( Recon Family Size = 40 Final Multiple Alignment Size = 23 )

TGGATTCTAGCTTGAAATATACTTATTCATGTTACCATACTAGAACTTAT

TGATTACTTTACATGTATAAGGAATGTATGTGTTGGGGTCAATGGAGGGT

GGAGCTTAGGAACTAACGAAAGTTACTGAGTGAGAGAAGGGNAGTGCAGA

ACGTTTACATTCTGTCGGGATATGTTATTGTTAGACATCAGGGATCCTAT

GGAGGGAGGAGAAGGGCAACAGTCTGGTNTCAGTCACCGATAAGGAGGAC

AGTCGTGAATTGGGGAGGAGTGAGGAATTCGGGNCGAGGTCAGGTCAGAT

GAAGTGAGAAGGAACAGCGGTAT

>rnd-5_family-595#Unknown ( Recon Family Size = 32 Final Multiple Alignment Size = 23 )

GAGGTGTGAGCATGGCCAATAAAGTGCTAGGGAAGGTTAATGCCAGGACT

AAGTTTTTGGCTAGAAAGTCCAAGCTGCTTGATAAGGACTCCATGAAAGT

GCTAGCTACTGCCCTCATTCAATGCCATTTTGACTACGCTAGTACTTCCT

GGTTTGGGGGCTTATCTAAGCTTATGAAGGGGAAGCTCCAGATAGCCCAG

AATAAGCTGATCAGGGTAGTATTGAAGGTGAGTCCACGTACTCACATAGG

CAGGAGCTGCTTTCAGGAACTCAACTGGCTGCCTGTTGAGGCTAGGGTGT

CCCAGATTAGACTAGGTTTGGTTTACAGGAGTATTTACGGTCCTGCGCCC

AGATATCTAAGTGATTACTTTCCCCGTGTTAGGGATGCACACAATCACAG

CACCAGATCAGGTGTTGCTGATGTGTGCTTATACAGGTTCAGGAGTAATG

CTGGGAAAGGTACTTTCTTGTATACTGGAGCCTCAGAATGGAATGAGTTG

CCTCTGCCCATAAAAACAACGTCCTCTCTGGGCAGCTTTAAAAAATAAAG

TAAAAATATGTTTGATGTCTTCTGTGCCCATATG

>rnd-5_family-1049#Unknown ( Recon Family Size = 32 Final Multiple Alignment Size = 23 )

AAACAATTACAATGGCAAAGTCACAATAATCACAAGAATGGCTTCAGATC

AAAGTCTACGTTGAGACCGAAGGGAGCAAGGGTCTTTAAGTTAAAGATCC

AGGCAGCCTCTCGTTTTAACAATAAATTATCAAGGTCACCCCCTCTCCTA

GGGAGGGTGACATGTTCGATGCCGATATAACGCAGAGACGAAATCGAGTG

GCCCGCCTCCAAAAAGTGGGCCGCAACTGGGTAAGTCAAGTTTTTGCACC

TAATGGTGCTACGATGCTCCGAGATACGTACTTTTAATTCGCGCTTCGTT

TTACCCACATCATTTTTACCACAAGGACAAGTTATAAGATAAATAACTGC

CTTAGTGGAGCACGTAATAACGCCTTTGATTGGGATCGATTTCCCTGTTT

GTGGGTGTTTGAAGGATCTACATTTATAAGTGCCATTGCATTGAGCACAG

CCATTACACTTGTAGTTTCCATCCGGTAGGGGCGCAAATAGACGTTGTTC

AGGAATATCTTGGGGTGGTAAATCAGAGTNTACCAATTGGTCTCTGAGAT

TTCTGCCCCGCGAGAATACGACCAAGGGAAGGTCCGAAAACACATTACCG

AGACTGTCATCGTTTTTTGCAAATTCTTTTGATTCGACAGAATTGGCT

>rnd-5_family-1190#Unknown ( Recon Family Size = 32 Final Multiple Alignment Size = 23 )

GACATGCCAAACGTGTGCTCAATGTGTCCGCTTCNGTGCCATCATTACAT

GAATGCGGAAAAAACTGTTTTGCATGACTTCCACCAATCCCCGTGGCCTA

GATGATTAGTATCTATGCGGATCAAAGTTGCCATGTGAGGGTGTATTAGG

ACAGTACAACCGCGTCAATACTAGGTTTTATTCCTGTCAATGTCCATCCA

TGCAATTTTTTTGTATTTGTAAATAAAAATGAAAATGTAAGAGCCTTTTT

GGGAGCAGGTATGAAATTAATAGATGAACTGAGTTATGACAGTGAAAATT

ATTATGATAATGTTTGCACAAGATAAAATCAACATTGATTATATCCTATA

TTATAGAATAACTGAAAAGGATTATGATCATATGTTTGCACACCACAAAA

TGTCAGTATTAATTATTATCATCCATGACAGAATAGAATCATTAGGATGC

ATGTCCCTGATTATTTGCTCTATTTATTTCAGTTGAGTTTGAGGGCCACA

TTCCAACAAAGATTCCTGATCTACCCCCTTTAACTGAAGTCACAGTGTCA

ACACTATTTGAAGAAATAGAGAACCAGTGGGTGGTCTCTGACAGGTAATC

ATTTAATGTAATTCAAACAATTTCAGTGCTGCA

>rnd-5_family-6991#Unknown ( Recon Family Size = 31 Final Multiple Alignment Size = 23 )

TAAACAGGACAAATCTGAGGGGGCACGTGCCCCTGTGCCCCCTATGGGCA

TGACG

>rnd-5_family-2315#Unknown ( Recon Family Size = 25 Final Multiple Alignment Size = 23 )

CACTGGGCACACCACGTCATTTCAACGTGGATAATTGGGTAATATTTGGT

TGAGACGTTGATCAATGAGATTA

>rnd-5_family-47#Simple_repeat ( Recon Family Size = 25 Final Multiple Alignment Size = 23 )

GGTTGCAAAGGGTCGGAAACTTTCCGGTAAATTTCCGGAANTTTTCCGGA

AATTTTCCATGGGAAGTTAAGCCCGGGA

>rnd-5_family-1056#Unknown ( Recon Family Size = 28 Final Multiple Alignment Size = 22 )

AAGAGCGACTGCCTTTAAAAAGCAACGAATCCCTTTGAAAACGGCCTATG

TGGCATCGATATGAGTCAGAAACANTTATTCTAGTGTCAAAATTGACTAC

AAAGTGTAAATAGGATAATTTTGGTCTCGTCTAAAACGGAGTTTGGAACG

TCCGTGCGTCACAACTTACATGAGGGTTGGGTTTGGATTCGACGACGTAC

ATTTGCCCACTAACACGGGGCGAACAGCTGCGNTGATTGCTCTCTATCCA

ATAGCATAGACAGTGAGACAGACCTGCC

>rnd-5_family-224#Unknown ( Recon Family Size = 28 Final Multiple Alignment Size = 22 )

CTGCCATCCAGGACCTCTATACCAGGCGGTGTCAGAGGAAGGCCCTAAAA

ATCGTCAAAGACTCCAGCCACCCTTTTCATAGACCGTTCTCTCTGCTACC

GCACGGCAAGCGGTACCAGNNNNNNNNNNNNNNNNNNNNNNNNNNNNNNN

NNNNNNNNNNNNNNNNNNNNNNNNNNNNNNNNNNNNNNNNNNNNNNNNNN

NNNNNNNNNNNNNNNNNNNNNNNNNNNNNNNNNNNNNNNNNNNNNNNNNN

NNNNNNNNNNNNNNNNNNNNNNNNNNNNNNNNNNNNNNNNNNNNNNNNNN

NNNNNNNNNNNNNNNNNNNNNNNNNNNNNNNNNNNNNNNNNNNNNNNNNN

NNNNNNNNNNNNNNNNNNNNNNNNNNNNNNNNNNNNNNNNNNNNNNNNNN

NNNNNNNNNNNNNNNNNNNNNNNNNNNNNNNNCT

>rnd-5_family-439#Unknown ( Recon Family Size = 23 Final Multiple Alignment Size = 22 )

CTCCTTAGGAGGGATGGGGACACCACCACGGGACAGACCCCACACTCATC

GCTAGAGATCGAACTTCAGACTCCCTTCAAAACCACTGAACCCCTCAACA

CACCGAAAGAGATTACGGTGCACACCCTTAGAGGGAGCCGGAACAGGATA

ACTCCCTCCACCATCACAGAGACACAGAGGAGGAGGAACTATCCAACACC

CAGGGAGAGAGAACAAGTGACGTCAGTCGAACACAGTCAGAAAGAGGAGG

TGGAAGAGGCGTTAGATAGAGGAGGATATTACCCCAGAGGCCATTCCTCC

TGTTCATGTGAGCAAGAAAGAGCAGAAGGCTTCTGATCAGCCAAATGGCA

GGCAGAGGGAGAGGTCATAGTGATTCACACCCACAGAGAGAAAGACAACC

CACACA

>rnd-5_family-401#Unknown ( Recon Family Size = 23 Final Multiple Alignment Size = 22 )

TAGATAACCTCCTTCAACGAATGACAGAATATCTCCTATATTTGGCAAAA

TCGAGTTGATGATTATACAGATAGCAGATCAGCTCATGAATAACGGGGAG

ATGAAGAGTGGAAATAGTTTAAATATCAAGGTATCTTAACGGGGACCAAC

TCTGTTTAAAAAATATCCGTATCTACTCTCGTACCGTTTGTTGATCACAT

TCTCTACCGAAGCTCTCGTATGGGCAGCAAGTACGAGACAGCGCGGAGGA

GACGTTATAGCGGTATTTTGACATGCATAGGCGAGAGACNTGCTTTGATN

ATGCAACCTATGGATTACAGAATAAAGTTAGGAATTTTCACGCGAGACAG

GAGTCAGGATTTTGGGTTTCAGTGGGATTGGGACTGGATAGGAAAATAGC

CTAGGCTCTAGGACAGGAGAAGATATAATTTTCCCTCATGCCTCTCTGAA

TTGTTAACANACTTNATGTCTGTGACAGAGATGCCCATGTAGTGAATTGT

GCATAGGCCTATCAAATGTGTGACTGTCCGAAGAGTCACAACGACGGCTC

AAAGAGGCACGCTATTCTTNGATAGGCTGTACCGTAGGTGTTTCCTGTAG

GGCTTACTAACTGTGT

>rnd-5_family-300#Unknown ( Recon Family Size = 24 Final Multiple Alignment Size = 22 )

ATATGAGAGGTTGCTTTTTCCATTCAAAACTCTTTGGAATTACCTGCACA

CCAAAGAAATNTGGTTCCTCAAGGTTCTTTGGGAAGGGTGATGGTTCTAT

GTGGAACCATANTGACCCAAAGAACCCTTTGAGCTCTTCAATGGTTCTTT

ACAGTTCACAAAAGGGTTATTTGCTCTTTTAGTGGTAATTAAAATGTAGG

GGCGGCGGCTCATTAGAAAATATTTTGGGGCGTGGTTTGCTCACAGCTCT

TTTTGTGCAACCGTGAGTGAGAGTGTCATTCCAGTTTATCTAATCTGTTG

TGTTACGCCATTACACGTTATACGTGACCAGTGCTGTGTCTTGTGAAAGA

CTCACGTATGGCCTTGTGTCNATTGAGAACGGTTGACTAATCAGTGGTTC

TCAATTCTCTCCTCGGGGACCCCAGCCGTTCCAGAGCTAGCACACCTGAT

TCAACTTGTCAATTAACACAATAATAAAACCTATG

>rnd-5_family-736#Unknown ( Recon Family Size = 24 Final Multiple Alignment Size = 22 )

TTAGGCATGACATGCCAGCAACAAACGCCGACACTACACATCCAAGTATA

NCTGACCAAATTATGAAAGACAAGCATTGTAATTTTGAATTCCGTAAAGT

GAGTGTGGAAG

>rnd-5_family-443#Unknown ( Recon Family Size = 23 Final Multiple Alignment Size = 22 )

TCTGAACTCCCCGTAAGGNGAAACAGCGCCACTGTTCGCCCCAGCGCATT

TGTTATTGTTTTTGTTTTGTTGAAGAACAGAGGNGAGGAGCGTCCGGCAT

CGCGGTAAAAAAAATCGCAGTACATACTCTGCTGTTCTATCGCGCGTGCA

ATGATGTCCGAGGGAAACAAACGGTGTTCGTTGTTTGAAGTAACTTCTTT

GTTGTTGTAATATCGCAAACGGACGTGGCAGTTTCACCATTAAGGATTCC

AGCTTTAAATACGAACCTATCGTTTAGTAGTAATCTGCCGTACAAAATAC

TATATAGCAACATCACCCTTTCAATNTGGCATATTCCATGTACTCNTATG

TTTAGTATTGTATTGAGCATGGANACAAACATNGTAGATAATGTTGCCAA

TATGTCACAGTNCAGCCTACTTCGTAGTATACTAGNCTGCGTGATGC

>rnd-5_family-3351#Unknown ( Recon Family Size = 34 Final Multiple Alignment Size = 22 )

AAATAAAGAGAGTCGCACACTCCATGTATAAACTCCCAGTAATTTANTGG

GTAAATCACCAACGTTTCGGCATCACTGTGCCTTCCTCAGGGTAACGTCA

TGAATACTTGAACCAGGTTATGTAGACAAACAGTGCAATTAGTGCAACCG

ATGACAATAGTGATGGTGTGTCATAATGATTAAGTTAATTAGAATGAATT

AGTGAAACTGTTAAAAAACATAGTTTACGGCATATTAAATATATTAGGCT

ATTGTTATCATATGGAAACATAATTGAATATTGTACATNATATTAGCGTC

AACATACATTATTGTTTAATTCAATTTCACATAATTGTTTCGTATTTCAT

TTCGTATTTGTTACATGTAATCTTTTGGTTCAACCTTAATGCATAATGAG

CATCATCAACGGGTGTACGCTAATAAGCTGTAAAGAATATATCAATTTAT

AAGACTTGTTCATAAANGAAAATTGTTCATAAGAAGGGCCTGAGATCAAA

GTCAATATTCAGACCACTAGGGGTCAATGTTTTTAGATAGGGTATCCAGT

AGGCCTCCCTTTGTAGTAGTAC

>rnd-5_family-3157#Unknown ( Recon Family Size = 24 Final Multiple Alignment Size = 22 )

GGTAGCGGGCCGCGTCGGTGGCACTGTATTATCCTCAGCGGGCGAAGAAG

GTGTTTAGTTTGTCCGGAAGCAAGACGTCGGTGTCCGTGACGTGGCTGGT

TTTCCTTTTTGTAGTCCGTGATTGGTCATAAACCCTGCCACATACTTCTC

ATGTCTGAACTGTTTGAATTGAGATTCCACTTTTGTATTTTATACTGACA

T

>rnd-5_family-1001#Unknown ( Recon Family Size = 41 Final Multiple Alignment Size = 22 )

GCGGGNCGCCTAAGCGTTTTTTCGGGTCGCCTANGTCCAAACAGTGTAAA

ATAAATAAATACATGTAGATTTTCGGGATGGAAAAACGCTTTCAGTGTAA

ACTTAAACGACTAAAACCAGATATAAAGTATGTAGAAAAGATAATGGACC

TATATATTTTTTNATAATATAATCTTTGAGAACTAACAATCACCAAAATA

AAAGCTAGACAGTCAGGGAGAATTGAAAATTCAAAACATTTATTATGTTT

GAGCAAAATAACACA

>rnd-5_family-2533#Unknown ( Recon Family Size = 25 Final Multiple Alignment Size = 22 )

GCAAAGATGGTGGATAAATGAAGATGGTTCACTCAGGCCGTTTACCATTG

AAAAAAATGGTCAGTTCCGGTCTACTTAACTGGGGGTGTCTCCCATAGAC

ACCAATGCGATAGCAGCTGGGTCTGGTCTACTTAGTTCATTGACTTTCAT

TGAACACAAAT

>rnd-5_family-132#DNA/Tc1 ( Recon Family Size = 24 Final Multiple Alignment Size = 22 )

ATTCCAGAAAATGATGTCATGGCTTTAGAAGCTT

>rnd-5_family-3529#Unknown ( Recon Family Size = 30 Final Multiple Alignment Size = 22 )

AGGCCTATGAGCCCAAGCCCGAAAAAAACCTGAATTAAAATGATGATTGT

GCCGTTATACAATACATAGCCTACCGCATATTACGCATGGCAGAAAAACA

TAAAAAAAACGTGATTTAAGATGTCTTGGTACATAATTGGTCTAGCCTAT

ACTCCAAAATTAAACAAATTTGAGTAATCGCCTTTGAGTGTGGACTGTAT

TATTATGCATACTGGATGGACCGGTTACCTCTACGCTCCAAAATGTNTAT

CCACGAGTCTGGGAGAGAACGTATAGCCGCCTAGGCGATGCTGTTGGTTC

ATTGATTGTGCAGGGCGGCTTACAGTTAGCCTACAATTAGTGAATTTGTA

TTTGATTTTGAATAGCCTAGTAATAGGGCATTTTTTTAAATATTTTCATA

ATTAATTGGGTGACACATTACCTTTAGCTATACAGAATATCTCACCACCG

TGCGTTTCCATCTCCTCCTCTCTCTCCTTCATTCCTTCTCGAGCGCGC

>rnd-5_family-5300#Unknown ( Recon Family Size = 25 Final Multiple Alignment Size = 22 )

AATTGCACCCACCATATACCTTCCTCCTACAGATAACCATTAACTTCTGG

TGTAGACCCTCTAAACCNTAGCACTCCATATTTCAATAGATACCTGATAA

TTAACCCTATTCCGAGTTTAGGAGTTAGAACCCAGAA

>rnd-5_family-12#LTR/Gypsy ( Recon Family Size = 26 Final Multiple Alignment Size = 22 )

GCCATAGTTTAGGGATCCCCATTGTTCCCGTGGATGTGCCCTTCCCCGTT

CACGCCTTAGATAGTCGACCATTAGGGTCAGGGCTGATCAGGGAGGCCAC

CGCTCCTCTGGGCATGGTGACGCAGGGGGGTCATAAGGAGCGGATTAGTC

TCTTCCTTATCGACTCNCCTGCGTTTCCAGTGGTGCTGGGCNTNCCCTGG

CTAGCTCGTCACAACCCGGCGGTTTCNTGGCGACAGGGGGCTCTCACGGG

GTGGTCGCGGGAGTGCTCAGGNAGGTGTNTAGGGGTTTCCGTCGGCGCTA

CTACGGTGGAGAGTCCGGACCAGGTCTCCACCGTGCGCATTCCCCCTGAA

TATGCCGATTTGGCTCTCGCCTTCTCTAAAAAGAAGGCGACTCAATTACC

ACCCCATCGACGGGGNGATTGCGCGATAGACCTCCTGGTAGACGCTGCAC

TTCCCAGGAGTCACGTGTATCCCCTCTCCCAGGCGGAGACGGAGGCCATG

GAGACATACG

>rnd-5_family-388#Unknown ( Recon Family Size = 24 Final Multiple Alignment Size = 22 )

GTGGTATTTATGTATTCATTTGTGATGCTATCCTTGATATGATCCTGTTA

TTGTCACATGCTACACATGCACATGTGTGCCCATGCATCTATCAATCCAA

TGTTATCATGATTTGTTATCAGGGATATTATACTTTAGTAATCCACAATG

ACCTGGTGATGTAAAAGTCTAATAATTACATTGTCTTCCATATTCCTACA

GATACCTTGTAACTGGAGATTCCTTCAAAACGATTGCCTACAGTTAACGT

GTAGGGCACTGCAAGGTTGGGTGACCAGGGCCATCTGGGACTGCCTCCTG

GAGGAATTCAGGTGTGTGAAGGCTACCTGTGTCCTGCATAACTTCATGAG

GATGGACACGAGGACCAGGAGGGGATCTGCAGCTCGCCGCCGTGTGCCAG

AGGAGAAGTCTGCTGCTCTGCAGGATGTTTCAAGGATGGGGTCCAACAAC

GCAGCAAGAGAGGCAATCCGTGTGCGGGAGATCTTCACCTCCTACTTCTT

CGAAGAGGGTGCTGTTCCCTGGCAACACCATAGACTACACTATGCACAAC

CAAAGGCTCTTTTAAGAGCCATTCACATTGCAATAAGAGTATTCTTCCT

>rnd-5_family-2275#Unknown ( Recon Family Size = 30 Final Multiple Alignment Size = 21 )

TGACTTTCAAGAATGCCTGAATTGCTTCGCCTTGCTTTTCTGGTTGGCTG

GAGCAAGTTTCACCATCCAATTATTTCAGATCTACAGGAGTGCAAGTTTC

ACCATGGCACGGGCCGTGGCGATATGTTGGCACATGATAATTATTAGAAT

ATTAGTCAATTATTGCATTCTATCCATGCTGGCTTTTGCGGGCTACCTGA

AACATGAAACAGATAGGTGGGAGGGCATACAGGCTGTCGCCAATTTATTA

ATGCGCAATTTGCCTAAATGGGTAATGGAAACACTTCAACCACTACGTTT

TTATTCTACCGTGTTTTTGTTGAAGTGTGACGTCATTACGTCCAGCCGTT

TTTATCGACACAAGATAGTTCAATGGAAACGCACCCTAGCAGGCAATTGT

CGNATCTATTTTCTACGCAAACTTTCTAAATGTCGACAACAACGAAAAAT

CNCTGGACAAGTTAATGGAAACATAGCTAA

>rnd-5_family-2625#Unknown ( Recon Family Size = 27 Final Multiple Alignment Size = 21 )

TCATACTCATTTCGGCTTTTCATCTAGTAGAATTCGCTGCACGCTATTGA

GGAAGAGAATCGTCTTTCGAGACCCACGTGTGTTTGACAACAGCTGATAA

TCAGATAAGAGGACGGGCTGTACCAAAATGAACGAATGGCGGGAANCGGC

GCAATTGCGCATTAGATGAGCATTCTCAGTATGGATGAGCCTAGTATGTT

GATATTTGTTGCTTACTGCATTCGTTTTACTAAACAGTACGTTCTAAATA

GTATGTACACGATTAGTACGCAGTATGCAGTTTTAGTAAGTAGTAGGCAA

GACAGATTTCGGACACAGACGATATCTACAGGAAAGTAGGCCTTAAATCG

ACTTCCAAACACGGGTCTGAGTGTGGCTCTCAGGGTTTGTTTGAGAACAA

AATGTAAATACATGCATACAATGCATGCA

>rnd-5_family-372#Unknown ( Recon Family Size = 22 Final Multiple Alignment Size = 21 )

AACCTGCTGATTAGAAGGTCCTGTGTAGATTGTATTTTCAACCAGCAACT

ATCAGGAAATAACGCTGATACATTTTTTTCACACTTTTACAGTGTTAGTT

TCATCAGCTGTTGTACAATATGATACAAAACACAGGAAAAACTGAATTTT

GACCGCACTGGGCCTTTAAGAGCCCAGTGCAGT

>rnd-5_family-615#Unknown ( Recon Family Size = 39 Final Multiple Alignment Size = 21 )

AAGTACTTGTGCTCCTGGATAGCACAACGTTTTTTGCTCCAGGGACTGAG

ACATTTCTCACCATTGAACTGCCCAATACAACGGCCGGAGAAGGGGATGG

AGAAATCCGCCCANTCCCACCCCTCACACAATTCGTGGAACCTTTCACGG

GCCCGCGAGAAGCAGGATAGCGTACGCCCGTAGGTCCAGACCTCCCACAG

CAGGCAGTGCCCCGTCAGATCCAGAGAGAGGGAAAGGAGCCGAACTCGAC

GACGAAGCCGCTGCTGGAGTCAGCGCGGATGGAGTCAGCACAGCCGTCGC

AGACACAGGCAGNCCCAGCGACGAAGGCGCAGGTAGATCAGGCACCAGGG

CGGCGAAGCTATTCCTCGTCTNNATCCGCTCCGGACCTCTCGCGGNCACT

CTAGTCCGCTTNGCGGCATGCCGCTGCCTTCGGTTTCCACGACTCGTGAC

ACGGGACCAGCGCTAATTGGCACGNTCCTCTTGNTGTTCTCCNTCTACTC

CGCTACGAGATGGGGGAGGAGAGGCAGATGGAGACGACTTCCTTGGCAGG

AAAGTTCCGGGCACAGGCCATTCGGATAGGGACAGACG

>rnd-5_family-3396#DNA/TcMar-Tc1 ( Recon Family Size = 23 Final Multiple Alignment Size = 21 )

ACAGATGAAAGCAGGTTCACACTGAGCACATGTGACAGACGTGACAGAGT

CTGGAGACGCCGTGGAGAACGTTCTGCTGCCTGCAACATCCTCCAGCATG

ACCGGTTTGGCGGTGGGTCAGTCATGGTGTGGGGTGGCATTTCTTTGGGG

GGCCGCACAGCCCTCCATGTGCTCGCCAGAGGTAGCCTGACTGCCATTAG

GTACCGAGATGAGATCCTCAGACCCCTTGTGAGACCATATGCTGGTGCGG

TTGGCCCTGGGTTCCTCCTAATGCAAGACAATGCTAGACCTCATGTGGCT

GGAGTGTGTCAGCAGTTCCTGCA

>rnd-5_family-1196#Unknown ( Recon Family Size = 49 Final Multiple Alignment Size = 21 )

GAGCCGCGAGTTGAGGACCAATTAAATCAGATTCCCTCCCTCTCGCAAAG

ATTGCGTCATCATCCCCTTCATTTGAGGAAGACGACTGATTAATTAATAT

ATTTATTCAACGCGTTCTTTTTTGTGTTAAAAAAAAAGTCGTGTTAAAAT

ACCTATCANATTACACGTTTAGTAATGACAGAATGCANTTGAAACTTCAC

GCGNAGTAAAACTTTTGCATGACTTAATAGAAGTATTTTGATAGGAGTGG

GAAAAAAGGTGCTTGTGCATTGATAACACCAAAGACGGCATTAACGATGA

GTAATAAAATAAAGACGGCACTTCTAAAAGCCTATTTCACACCATAGCCC

GCTGAATTTGCAAGATAACTTTTCTTTCATTTTGATTTCGGCACAAATGA

AAATGTGGCACTGACTCGCCCATGGATTGATGTTTCAGCATTGTCTCAAT

GAAGAGTTCGGCGTTCGACTAGCCACGTGCGACATGCACGCGCAGTTACA

AGCCTGCTAGAGTTAGCGGCAGGCGGACGAGCAATATCCACCGTCGTAGT

ACGGATGAAGTCCTGAGCTGGAATGTGAAGCTAACTGAAGC

>rnd-5_family-492#Unknown ( Recon Family Size = 23 Final Multiple Alignment Size = 21 )

TAAGAATATTTTGAAGATAAATACACAACGCAGAGGACGAGAGGACTACT

AAAACTAGAGTACTAACGGTCAATAGGGAATTGTAAATAGGAGTTGGGTT

TCAGTTCAACAGCTGTTTAGAATCTGTGGAATGTCACTCCTGAACTCAGA

GCTACGTGTGCTACGTTCTGTAGTAGGGAGCAGGATACTTGTTATTTGGC

CATTGGCCCAGTTGTACTGCAAGGACTTCTGATTGGTCAACCCCAGGCTA

GGTTGGGGGGGTTATAAATGACATCCTGTCCCTTTGTTCGGTGGAGAAAA

GCTGAGGACAGGGGAGACTGAACATCTACCTCCCAGCATAACATCTTGAT

TTGTCCTTCTCGAATAAACCTATTTTTCTCCCCCTGATTTGCTTTGGGGT

CTGTGTTATTGAAGAATAACATCAACTGCTAACAAC

>rnd-5_family-1665#Unknown ( Recon Family Size = 29 Final Multiple Alignment Size = 21 )

GAGCGACAAAGGGAGAAGCTGTGCTATCGTAAATACAGTATCTTATGCAT

TCTAAATNACCGCCCATTTGGAAAAGGAAAATGCAATAAATATTTACTCT

GAGCTGCGCTTCGGTAGGTTGGTGGTAGATGGAAGGCCGTGTTGCCCAAC

CGAGTCCTTTGTCCTTTGAAGAATGTCTCTGGTGGTCAATTGGATACGTT

GTAGTAACGTCGTTGTGTGGTAGACGGGATACTCTGTCCGTTCCTTCCTA

ACCCGCGTTTGCAGCTGCTGTTGCTAACTCGACGGCTAGGAGGTATCACT

TCTGTAGTGAATAAGAGTTCAAAGTTCATACCATTCGCAACCAAAGCTCA

CGCTGATGTTGGCTTCGTTCTGTAGTTATTATCTGAACCATT

>rnd-5_family-1560#LTR/Gypsy ( Recon Family Size = 23 Final Multiple Alignment Size = 21 )

AGAGCAAAATACACCCGGTGAAAATGAAGGTTACTGGTTATACTGGGGAG

AACGTACCAGTCAAAGGNAGCTGCATAGTAACTTTACAGCACAAAGGAAA

ACAGTTCAGAGCACAGCTGCTGATTGTGGAAAAGAGTGTACAGCCTATTC

TAGGAATCAATGCATGTGAAAAGCTCAATTTGTTGAAAAGAGTGTATGTA

GTGACATCACAGACTGAAAATGACCAAGAATCAATACTGGCTGAGTATGA

GGATGTGTTTGAGGGTCTTGGATGTTTACCAGGAGAACACAAAATATGTA

CTGATGACAAAATTACTCCAGTTGTGCATGCATGCAGAAAAGTTCCATTT

GCACTGAGGAAAAAGCTCAAAGAGGAACTCGGACGCATGGAAAAGATGGA

CGTCATCACAAAAATGGATGAACCTACAGACTGGGTAAGCTCACTGGTTA

TTGTGGTGAAAAAGAACGGCGATCTCAGGATATGTCTAGACCCGAGAGAT

CTCAACAAAGCAATCAAGAGAGAGCATTTCAAGTTGCCAACCAGAGAAGA

GATAATGTCGCAGTTTGCTGGAGCAAAGTGGTTCAGT

>rnd-5_family-2320#Unknown ( Recon Family Size = 22 Final Multiple Alignment Size = 21 )

TTTGTTGGCCAAGAGGCGCCATATTACTCAGTGCCGAATGGCCAAGAGGA

GAAGACTGAGGGACACATGATGGAGGCAANGTCTGAAACCTATCGTCTTC

ACTCCTTTGAGTACCGGGCTCCGGTTGCTTGGATGGGTAGTCACGCTGTA

GAGCGTGACCGTTCTGGACTTCCTCCAGATGGTACCTAAGGGTGGTATTC

TGCTGCATTGCAGAGTCCACTTGAGCGCTTAGAGTACTGATTTTGGACAC

GTGAGTGTCGCACTTGCTCCTTTCGGTCTCAAACTTATCTGTCATGGTTT

GCAGATAGAGGTCTTGAGTACTGAGCGAGAGATTCAGTGATGAGATTTCC

TCCGCTTGCTTAGAAATCAAGATTT

>rnd-5_family-2732#Unknown ( Recon Family Size = 22 Final Multiple Alignment Size = 21 )

TGGTGAAACCTAGCCAATTTAGCAGGTAATCTTTCAGGAATATAATTACA

TTTCAGTAAAAATTGAAGACCTCCCAATTTATTAAACACATTATTTGGAA

TGAAATACCATATTGAATCAGTATCGATCAAACATTTTTTCAACCAGTTT

ATCTTGAAAGTGTTATTTATGTCAACAAAATCCAACACTTCTAGACCGCC

TTCAGCTCTTTTGTTAGAAAGGACAGATTTTTTTAGTTTGTGAGACTTAT

TTTTCCAGATGAAGTCAAGAAAGGTCTTATTGATCTCTTTACAAGTAGCT

GGATTTACACATAACGATAATGAGGGGTACACAAAACGAGACAGTCCCTC

TGCCTTGGACAGAAGTACTCTCCCAAGTATAGAAAGATCTCTTTGTAGCC

AATTATTAAATATATTTTTAGTTCTCTTAATTTTAGGAGAGAAATTCAAA

TGTTGTCTGACTAAGTGTTTTTTTGACAGATGTATTCCTAAATATTTAAC

ACAGTCCTTTACAGGAATATTTTCTATTTCTTTATCATCAGAGTCAAATA

AACATAAGATTTCACATTTAGAAACATT

>rnd-5_family-863#Unknown ( Recon Family Size = 22 Final Multiple Alignment Size = 21 )

GGTGAGCTCACCCGTGATGTGATTAATTATAAATAGTAATTGCTGTTAGC

TAATTCATATTGTNGTTTGTCAGCCGAGAGTTAGCTAAATATGACCGCTT

GTGTTAGCTCANTCTTTCCTCAGTTAGCNCTAGGACAAATGTAGCCTACA

TTTTTCCGGTTTGGATGATTGTGTTATGCTGTAGCTACTTCTGTGAATGT

CTGAACATTGCATCCAAAAATAAACTGCTCACATTCNGGACATAACTTTG

TAAGGACTGTGTTTGTGCAAAATGTTTCTGTGAAAAAAGTGTGGCCAGCT

CAAACGCTGATTGTTGCGTCATTCGAAACTGGACGTTCTAAATAAGCGTT

CTAAATAAGCGTTCTAATCACACGGGTTTGAGCGTACGCTGCAGGGACCA

CTGTAGAACGATCACACCCGTGTGTTGGTACGTGTGAAAGG

>rnd-5_family-57#Unknown ( Recon Family Size = 28 Final Multiple Alignment Size = 21 )

CAGCATTATTCTCAATGAGCGCCGATGAAGATTTAGTCTAAAGCGCATTA

CAGTGGCTTGGAAACACGATGACATTTCNAAAGGAGGCAAATAATTAGTA

GGAAAACCTTTCGTCATCATTGTGATGTCGTCAGATTGACTTTAGATGCA

GTATAACTTTAGCTAGAAGATTGAGGGGAGACCGGATTTTAGCTAGCTAA

CGTTAGCATTGCTAGNTATTTTTGACGAACTTTGCTAGCTAATGGCAACA

TTGCCATCTGTCTAAAGTAAATTTGACGGAAAAGTTGTTTCCTACTAATT

ATTTGCCTACTTTAGCAATGTCATCGTATTTCCAAGCCACTATAGTGTAA

TTTAGACGAAACAGTCATTAGCCCCCGTTCAGAATGACTGGGCATTAGTC

AAC

>rnd-5_family-692#Unknown ( Recon Family Size = 26 Final Multiple Alignment Size = 21 )

TAGAAGCCAGAATGTTGAGTACAATTATATTTTAATTTACTGCACCAGTT

GACCGTGCCGTTGACCCGCTTGCTTACAGCTGCACTGGGGTCGGACAGGC

TTCCAGTTTAGATTAAGACACCGCGGAGCCGGCGCGAGATATGGCTCGGA

AAACGTACCTCAATCCAGGGATGAGAAATGCGTTGTACTCCGAATTGTCC

CATTATCCCAACTGTTACACCGAGAAGATTGAAATACTGCTGTTTTTNTT

GAAAAACTGCCGTTTTCGTCCTCATGCTAGCTCGCAGTAGCCATTATGGA

ATGGCACATCGCGTTGAACGACAAAGGAGCCGATTGGCTGACGCTGCCAT

TCCAAAATGGCTGCTGTGGCTACTGCTAGCTAGCAATGAGGACGAAAACG

GCAGTTTTCTAAAAAACTAGCAGTATTTCAATCTTCTCGATGTATCAGTT

TGGATAAAGGTAAAATTNGGAGTACAGTGGCATATCCCATCCCCTGCATT

GAGGTACGTTTTCCGAGCCATATCTCGCGCCGGCTCCACGGTGTCTTTTT

AAATGTCCCTGATTGCGTTCCGACCCCCAGTGCAG

>rnd-5_family-1127#Unknown ( Recon Family Size = 24 Final Multiple Alignment Size = 21 )

CTTCTTTTTTTATATTTTCAGTGGTATTGTTAACAAACACGCCCCATAAA

GAAAATGAGAATTAAAAACAGGTTCAGCCCCTGGTTCGACCGTGATCTNG

CAGAGTTACTCCACCTCAAGAATTGCATTTGGCGAAAGGCTCGGCACACG

CATACTCAGGCTGACTGGCTCTCGTTCAGGCAAATGAGAAATAAGTGCAC

TCAGGCTATCCGGAAGGCCAAAGTTAGTTACTTTAAGGAGCAGTTCTCTC

TCTGTG

>rnd-5_family-2064#Simple_repeat ( Recon Family Size = 24 Final Multiple Alignment Size = 20 )

GTAGTCATTGCGTGCTANGAATATGGGACCACCTTTTAAACTTATNACTA

CTTTAATA

>rnd-5_family-2334#Simple_repeat ( Recon Family Size = 24 Final Multiple Alignment Size = 20 )

CACATATAAAATAATACAGTTTACTATAGAATACTACAGTACTTACTATA

GAATTCTGTAGTAAACTGTAGTATACTGTAGAATACTATACTACACACTG

TAGTATCCCTCGATCATGTGTAGTACTTACTATAGAATTTTGTAGTATAC

TGTAGAATACTATAGTAAATACTAGTATTATCCACAAAAAAAACACTAGC

CAATACTATAGTAATGTTCGCAAAAACACTACAG

>rnd-5_family-3453#Unknown ( Recon Family Size = 21 Final Multiple Alignment Size = 20 )

CAGTGAAGTAGGGGTGCTGAGGGTGCTGCAGCACCCCCTGAAAAANTATA

ATAAAAAATATATANTATAAGTAGTGCACTGGGCCTTTACTAGTCCTATA

TTAGNGGACCGATATAGCCGTCTGTAGTGATATAGCGTTTTTTCGCTGCA

CCATCACTCGCAAACTACTTCCTACGGCTA

>rnd-5_family-517#Unknown ( Recon Family Size = 21 Final Multiple Alignment Size = 20 )

ACGGAAAGTATGCAAAGAAATATGACGCAACCACTTAAAAATTACGCAAC

ATTTCTTGAAATCAACGGCGGCCATTATTTGAGCTGAAGCGAGAGAAAAT

ACACTCCGACTTCGGAATGAAATGTTGCCTATTCACATCTCATATGTTGC

CCGACTACAATATTTTATCTTGATACGTTAATAAATACATGCCGTGTTAA

TTTTGAC

>rnd-5_family-2343#Unknown ( Recon Family Size = 21 Final Multiple Alignment Size = 20 )

AACCCAGCTAGCACATTTGGTTCCTTGGAAGTTGTGGGAACGTACGTTTT

TGGTTTCCCATTGGTTCTGGGAACGAAGCCATACGTTTCCTGACCGGTAA

ACACATTTAAAAAAATGTTCTGAGACCGGAAGTGAACATTTCGCCTGTTC

TGGGAACGTTCATTTTTAGGTTGCAGGGAGGTTCTGAGAACGTTTTACTA

TGGTTCCCTGAAAGTTTTCCTGGGAGGTTTTATTAACGCTCTGAACATTC

TGAGAACATGTTTCAATAAGACTTTTAACAACACTGCTAGCTTATTTGGG

CTAACTTTTGTGACCTCCGAGCACAGATAGGACACATGGAAATTAATTTC

CTTAGGCATTAATCATGCAAACACATTNCGTCAGTGAGATTCGAACCTAC

GCTCTTTCTCTATCCATGGAATTAGTCCACTGCGCCACCAGGATGGAGCT

AGCATGCCATGTTTTTTATG

>rnd-5_family-824#Unknown ( Recon Family Size = 20 Final Multiple Alignment Size = 20 )

CCCTCCCCTACACCCNAAATAATAATTAAAACAAAGTGGATAGCGGAGAA

CATGCCTACCGTTTACCCTCACTTCTNGGACAGACCAGAGCCTTAGATAT

GCCGTTTTAGAAACTGTTCTTCGTCCTGTAAGCATTACATGGCAACGCAG

GAATTTTGATAGCGCACAGGGCTGCGGGCCAGAAGGTTGTGGGTTCGCAG

ACCACCGTGGACAAGAGTAGGGGTGGAAAGATCTCCTTTATAGTAAAAGC

ATTGCATGAATCTATCAATTCGCTTTTATAAAGCATTTCATGCAATTTTA

CGCAATTTTACATATTAGCGGAATATTTTTTAATACCACACAAATTACCT

AAATTACAGGCTAAGAATGGACAGAGAGATAGGCCNATGTGNTCATCTNA

TCATATTTCTCCAATGCCAAATCAGGTTGCCACAGAAACTGTCCCTGTTT

GCAAATAATTAAATCTGAGACATCAGTAGGAATCTGAGCATGGTACTTTC

AAANGTTAGGCCTACCTTTCCACCCCAGACAGAGTAGGCCTACCGACGCA

GAAA

>rnd-5_family-5232#Unknown ( Recon Family Size = 21 Final Multiple Alignment Size = 20 )

AGCTCTAAGAAACATATGGTTCTCAGAACGTTATGTGCTAGCTG

>rnd-5_family-773#Unknown ( Recon Family Size = 20 Final Multiple Alignment Size = 20 )

AGTTCTTATAGATGCAACGGAAAGACAATGTATTCCATTGAGCCCATTTC

AGCCATTACCGGGGTGTGTTTGACAGGTCATTACAAACGTTTCCGCGGTC

GTAACGTTAACGGGAAAAAACGACAGGCACAAGCAAGAGTATGAAAGAGT

CGCAGGAGTAAAATGAAAGCAATCAATCCAGCGAGATTTAAGTGACAAGT

GGATTTTGCACCCCTCAAACACAGGAAATAGATGGCTGAAAAAGACAGAT

CCTCAGGTGGGCGGGGC

>rnd-5_family-445#Unknown ( Recon Family Size = 21 Final Multiple Alignment Size = 20 )

AGAGGAGGAGTGCGAGCTCTGTTTGGTGCAGGGTCATCATCTGAAGATGG

AGCACAGGGAGAATGAGGCCATGGAAACCCAAGCCTGCCAAGACAACAGC

CATGCAGCACCTGAATTCACCATCACCAACCCCGACTGCATGCAATGTAG

AAAATATGAGGAACATAAGAAGTCTGCAGGGCAGAGCAGAAGCCATTACC

AGGCTGATGCACAGAAAGATTGGCCTGAGGACTGGTCTGTGAGGAGCGTT

GATATGCAAAAAGTCATCATGCTCCCTCGCATGCCAGGTGTGAAAACAGC

ATTGTTCACAAGAAGAATCGTTGCCTATCACGAGACATTCGCCACCATTG

GAAAGAAGTCTCAAAAGAAGAAGAAAACAATCTCAGTGGTATGGCATGAA

GGAACAGCTGGTCGGAAGGCAGAGGAGATTACTTCATCTTACGTAACAGC

ACTGGAGAGTGAGAGAGATGTCAAGCATGCAGTGTATTGGGTGGATAACT

GCACTTCTCAAAACAAAAACTGGTGCCTCCTAACATCACTGGTGAGCGTT

GTCAATGCTGACTCAACTTTGATGGAGGACATCACCCTCAAGTTCTTCGA

GCCAGGACACACCTTCATGAGTGCAGACA

>rnd-5_family-225#DNA/Tc1 ( Recon Family Size = 27 Final Multiple Alignment Size = 20 )

AAAATTCACCCAACTTATTGTGGGAAGCTTGT

>rnd-5_family-217#Unknown ( Recon Family Size = 21 Final Multiple Alignment Size = 20 )

TTTTATGGTGAACGTCACCAATGAGCAGCCCCTGTATAGCAGGCGTTACC

>rnd-5_family-2446#Unknown ( Recon Family Size = 28 Final Multiple Alignment Size = 20 )

CAAACCTCCCATCCTTGCCCACCCGAGCTTGTCCCAACCTCCCATCCTTA

CCCACCCAAGCCAAGTTTGTCCCAACCTCCCATTTTTCCCACCCTCCCTN

CCTCTCAGACACACTTACACCCANCCATACGTACTCATCCATTCTCGTCC

ATTCCACACAAACATATACCCTATGCATCCACACACCCATTCTCTCCCAC

CCTCCTACACATATTCATCATCCCTCACNCNTCCCTCACACNCCCATTCA

TATACACANACGCGCACATCCATAGAACAGTTATTGACATACATGCTATA

TTTCCCCCTTTGAATTGTCTTTTCAGTGTCCATGTAGCCCATTGGCATCG

GCTACTTCTATCGTTACTTCATAGAGAAGAACAGTTACTTGGGGAAAGTA

GAGTATAGATTGTCGGGGGAGGGGGAAAGGATATTGTCTCAATTTATGAT

AAGCTGGTCTTAGGCATCACTGTATGTAGCCTAGTGCGCTCATCGTTTTT

NCTACTTCGNCCTCTTC

>rnd-5_family-1637#Unknown ( Recon Family Size = 21 Final Multiple Alignment Size = 20 )

CCCAAGAACCTGAAGAGTCACAAAGTATACAGCACCGTCTACAAACATCT

GTTGAAGGAGTTTGGCTCAGAGAAAATACTCCAACTGGCCGTGTCGACTC

AGGACTCCACTTTCAATAGGATTCTTGTGAAGTCATTGAGCAAGGAGCTT

CTCCACAGTTGTAACGAGGCATCAAGAGCAGCCTCAAGAACATCTTTCGA

AGCCACCAGGCCAGAAGCTCTTCTCATGGCTGAAGATGTGAAGGCTACCA

GAGGGAAGCTGTCTTTCCTACAAAGGCTTGCCAGACTGAAATTCAACTTA

AAGGTACATCACACTTATTTAAGTTAACAATTGTGTCAATGTGAGTAAAC

AATGTTATTTAGAGAAAATGTAAAACCATCCATTAATTCCAAAACCATTT

ATAAATCTTGTTGTGCTGGTGTGTAAGATGTGATCGTTATTACCGTGAGA

TTGTTCTGCTGTGACAGTTGTTTTGACATGTTTTTGTTTTAGCCATTCAT

GATGGGAAACAAGAAGGATTCCAATAAGAAATCCCGCCATTCTGAATCCA

AAGACCAGACTACTGCTGAAGATATCATGTGTAAGTCCATACAGCAGGAC

ATTTACTGTTTGAGATATTGGTGTACAAAGCTGCTATTGCAAAAATATTA

AACCCTATATACAGT

>rnd-5_family-1438#Unknown ( Recon Family Size = 23 Final Multiple Alignment Size = 20 )

ACCGCTTGCTCTGTAATATGGGTAGTATTTTGATTTCAATCATTTTCATA

CAATTTATGAATATTGAAAAATNTTTATGTGAATTTTGNATATTTTCTAC

ATATTGTTGAACATAATATACTCTACGGGCATATCAAAGTTCCAGAGTGG

GATCTCTGCTAGTTTTAGAGTTTTATACAGAGAAATTGGCATAGTAGGCA

ATGTAACCAATCACAGCCCTCCTTTTACTTGTATCATTGCACATCCTGCA

AATCACCAGCAGAGGGCGACCATTTTGAATCCATTTTCACGTTCACTCTG

TTGGTAATGCTTACAAATTGGATCATAACTCAAAGTAGCAGAGATCCCAC

TCTGGAACTTTGATATGCCCGTAGAGTATATTATGTTCAACAATATATTG

AAAAATNTGCCAAATTAAAAATGTTATATTTTCAATATTCATATTTTTCA

ATATTCATAAATGAATGTAAGAAAATTGTTATTGAAATCAAAATACTACT

CATATTACAGAGCAAGCGGTAAAGCTTCATATGACACCAATGTTCGCTTT

GTAGCA

>rnd-5_family-738#Unknown ( Recon Family Size = 21 Final Multiple Alignment Size = 20 )

AAGAGATGTCCTACCCTAAATGACAAATTAGTCCACAGTTATCTTCCGGG

TGACTCTCAAAAAACTTGGCTTGACCACAAACCCAAGGGCTCTTTTAAAT

GTAACCATTGCAACCATTGCAGAAATATTGCACAGAAGAAGTATTTTGTT

GACACAGCTTCCAAAATGGAGTATTACGTCAAGCATTTCATTAACTGCAA

AACCACTCATGTCATCTATAGATTGGAATGTCCACAGTGCAAGGTGTTCT

ACATTGGACGGACAAAGAGACGCCTTCAAGACCGCTTAGCGGAACACAAG

TACGCCATACGGGTAGGCAATGAAGACTACCCCATGGCAAGGCACTACAA

GTCCCTACACCATGGCAACCCTGCCTCCCTACAAGCTATGGGTATCGATC

ATATTCCGGCCTCTATTAGAAAAGGGGACCGTCTTAAACAGTTAAACCAA

AGGGAANGTTTTGGGATTTACAAACTACAGGCCACTAAATACCCTGGTTT

AAATGAAGATATGGATTTCTCACCCTTCCTGTAGGGTCGTGATGGGTCCA

TTTGCTGTCATCTAGTGGACATTTTGCTCAATTGCCCTCAGCTATGTTTA

GACTGTTGTTCATGTTTTGCTGTGTTCTAGT

>rnd-5_family-134#Unknown ( Recon Family Size = 21 Final Multiple Alignment Size = 20 )

GACGAAGGACTTCCACAGGGCCGCCATCTCCAAAGTTCACTCAAAAAGGC

AATAGTTCTGAACTGGGTTTGATTTAGTTTTTGTTGTTTTAATTATTTCA

ATTTTGTTNATTCTNTTTTAAACGTCCATTGGTTGAAAGCTCATTGTAAT

GTAGTCGCCCCTTTAAACATCCTAAGGTCGACATCCCCGTAGTGTTCACA

TTTCTCTCATGTAAATGTTGTAGCTACATNAAATGAACAATTATTTGGGA

AATATTCAGTCTTTGCTCTTTTCTTCATTAGCATGCTGGTGATTTCAAGT

TGTACTGGATTTATTTTTTATCTGTTAAGACATTGAATACATGTTTAGTA

TTATTGAGTTTANTTTTCTCTGTGAGAAGCAAGTTCATTTAAAGTTATAT

GTGATTTGTTTTATGTAGATACAACATTGTGTTAATGCACACAGTAACCA

AAAACCTGATCTAATTTGCATATTCANAATGAGTTTGCAGCAAATTTGCG

GCAAATGTTCGCCACAAGTTTCCCACAATTGTTTGCCAGAAGTTCACAAG

TCGCCGCAAATTTGCCGCAACGGTTTGCCACAAAACTAATTTGCATGTGA

AAAT

>rnd-5_family-52#LTR/Gypsy ( Recon Family Size = 21 Final Multiple Alignment Size = 20 )

AATCACTCACCGTCCAGGCAAAGACATCCCTGTCGCAGACACACTCTCCA

GGAAGTTTCTTACCTATAAGGACAGCAGCCTCAGTGAAGGCATGGACATG

CAAGTGCACACTGTGTACAGCAACTTACCAGTTAGTGACACAAAACTGAA

GGAGATCCGAGCAGAAACAGAAAAGGACTCACAACTCGCACAGCTGAGGA

AAGTCATACAGGATGGATGGCCTGAGGAGAGGAGAAAATGCCCTCAGAGC

GTCTCAGAATTCTGGAACCATCGTGATGAACTATCACAGATCAACGGAAT

AATTTTCAAAGGAGAGAAAATCATTATTCCTACCAGTCTCAGAGAAGAGA

TTTTGACAAAGATCCATGCTGGACACATGGGCATGGAAAAGTGCAAACAG

AGAGCACGGGACATTTTGTTTTGGCCCGGAATGTGCAAACAAATAGAGGA

CATTGTTGGTAAATGCGCCATATGTCTTGAACGACGCCCCTCAAACACCA

AAGAGCCAATGTTACCTCACTGTATCCCAGACCGACCCTGGCAGGTCGTG

GCAACCGATCTGTTCACCTGGAACAACGAGGACTACATCGTAACAGTGGA

CTACTACAGCAGATACTTCGAACTCG

>rnd-5_family-464#LTR/Gypsy ( Recon Family Size = 23 Final Multiple Alignment Size = 20 )

CAACATGGACCCAGCAGAGATCACCCAGATCNNGAATGTTGTAACCCACC

AAGGAGCACTGCTGGGGCGGCAGCAAGAACAACTCTCCCAAATATCAGAG

ACCCTCCGGGCACTTACCGACTCCCTCCAACCACAGTCCAACCCCAACCC

AGGAGGAGCCAATGCCCAAGTCTCATCGCCCAGTGCTACAGGCGTCGCCG

TAGTTCCTCCAGGGAACCTGCACCGGGAACCCAAGATCCCAGCCCCCGAG

CGGTATGACGGCCACCCGGGAGGATGCAAGGGGTTCCTCACCCAGTGCTC

TCTGGTGTTCGAGCTACAGTCCTCCTCGTTCCACACCGATCGGGCCATGA

TCGCCTACATAATCTCTCTACTCTCGGGCAAGGCCCTGGCGTGGGCAACG

GCGGTGTGGGAACAGCAGCCACCATCCTGCAGCTCCATATTAGCCTTCGC

TGCGACGAGTCTTCGACCACCCAGTCGGCGGACGAGAAGCGGCCAGCCGA

CCGTTCAACNTCCGCCAAGGGGCCAGACCAGTGGCTGACTTCGCCATTGA

>rnd-5_family-3253#Unknown ( Recon Family Size = 22 Final Multiple Alignment Size = 20 )

TCTCAATTTACATCAACAACATAGCTCAGGCAGTAGGAAGCTCTCTCAT

>rnd-5_family-1938#Unknown ( Recon Family Size = 22 Final Multiple Alignment Size = 20 )

TTGGGGTTCGTTCCTCCTTGTTCCTTGTCAATCATTGGCTCATTGGTGAA

ATTAGCATTATGACAATATCTTTAATTAAATAATTCAAAAACCTTTATTA

ATGCAATTGCAGACAGAAGTTGACAATCAGGAACATAGCACGCATGTTTC

CGAGTAAGTTCTGCATCAAAGNAAAGGTCNCACGTTGTTTTATCANACCC

GNAGTCAAGCCCTAGTGATGTCACTGCCTACGTCATTATCTTTTACTCAT

GAGACCAAANCCATGCCTACACAGCTATATAAACAAGGCTTTTAATGTGT

TATCGAAAAGCTTAGCAGTAAATGTCCAAGTTGATTTATAGTGGAATGTC

TGACTAGTCTCTTATCTCTTACACTCCCCTGCAGAGTTCTGTCTTCACAG

TCACACATTTCTCAGACGGTTTCTTCATAAGAGGCAGAGAGACTAGAAAA

GTACTGTGGAACAATATTAAACCTCATAATGTATATATATTGTTAATCTG

TAGTCTAGGACCCTATAAATGTAAGGAGGGGTCTTATAACCCCTCCCCTT

CTATTAATACAACATAAGCATAATCAATTATTCTAATACATCAANCATTT

GGTACAGCCCCNATC

>rnd-5_family-66#Unknown ( Recon Family Size = 24 Final Multiple Alignment Size = 20 )

TTGTTTCATATGAGGCGACAGTACCGAATAGGGATTCATATTTTCCTGAA

AATCTACCAAGTTTCAATACCGGGAATAATTCATGTTATCCCGAGAGTCC

CGGGAAATACCGCAGAAATCAGAAGTTCCAATTTAAAGCGTTTAAAACCA

AGATATGGTCACTATGCCAGGGTTCTCCCAAAATAAGAGNGGCGCTGCGC

CACCTTGTCGGCTTGGTTGCGCCACTATGTAAAGATTTCAGAGCAAATGA

AACTGATATTTAGTAATGATAGTNACTCTATCTTTGATCGCCAATGACAT

TGTTGTGAATTGCGAAACAGGCCGTTCATAAATTCCGAGCGTTATCATGT

TCCTCAATTAATTCAGCGCATTTCAGCAGTAAGGCTATCTCGACACAGAG

CGCTCAGGACTCACGTCGAAATCATACAAACTTTGTAACGGCAGTCAAAC

AAAAGTCAAATGACCAAA

>rnd-5_family-2462#SINE? ( Recon Family Size = 22 Final Multiple Alignment Size = 20 )

CTGCGTAGCTGGTGATACAGAAGGCTATGAATTTGTATTCTANCTCTGCC

TGCACTCTGACCTGTCATCTCAATGACATACCTATTACCGATGAATTGTA

CTCCTAAATGGATTGAATAAATTATCATTTTCCGGACAAGAAATAGACAG

GCAGAGGCTTCAAGAACAATACTTGCTCCTGGTGGGGCTTGAACTCATAA

CCTTGTCATCTAATTGCTGTATACTGCTGTATAAGTACAACACGCTAACA

GATTGCGCCACAGGATCCATCAACAACACTCGCTGATCACCATATTTTGC

AGAAACTGGGCTGTACGATATTCGNACCAAGTCAAGANNACNATTGAGGT

ACCTGGCTAGCTCAGTCGGTAGAGCATGAGACTCTTAATCTCAGGGTCGT

GGGTTCGAGCCCCACGTTAGGTGATTATTTTCTCCGCCGAAGCACCAACT

CACTTCGCAAACGACCATCCGCTTGGAATT

>rnd-5_family-4366#Unknown ( Recon Family Size = 21 Final Multiple Alignment Size = 20 )

ACGCCCACATCAGTAGAAATCCACTTTTTCGAGCTGAAAGACGATGGCCA

GAGCTTACCCATGATGTTGCACAACGATTTAAGTCAATAAGTCATAGTTT

TAGTCAAAGTATGATATAGTTGGCCTTGAAGTGACAAATCCGAACTGCCC

ACTTTGTGCAAATCCGTCTGAATGCGTTGTCTTTNCCTATGANATGACAA

ATTATTTTCAGCCTACGCTTCGTTTCAGGGCTGGGTTTTATTTNAATGTT

ACCACCCCNTNGCATTGTTTATTAATCAGAAACAGCTGCAAAGTTATTCC

TCTTCGTGAATGGGACGAACAGCCTTGTGTCCACTTGGCCACCCGAGCCA

TGGANCAAATAATAATAGGGCCAGAAAATTATCATAATCCATTGGATAAT

TTGTAAATTTTCACTTATTTTTTAGCTAGTCTGTATACAAAATACATATA

TCTCTGANCANTTTATAAATGGTATAATTGCGTNACATGGTAGCATTTNG

CAAACCCTCTGCCCCCGTCGCCCCAAGGTGAATNNTTTTGACNACTAAAN

TCTTGCTTCACTNCACGCTCCACTGCTTTGATTGGTGT

>rnd-5_family-383#Unknown ( Recon Family Size = 36 Final Multiple Alignment Size = 19 )

CTAGCTAACGTAAACTCACCCCATTCCGTACAAATGTGCGCAACCGCGAC

ATTCAAACGAGGCTGCAAAGAAAACTAATGGGACTGTAGCGACTGTGTTG

ACNTCAAAATCTGGGGTGTGAACTACGTTTCTATTCAAGCGTTGATCGAC

ATGGTAATGGCTCTATAGTATTGGAGAAAAGTTGAAAAAAACTGACCCTC

CGTTACATCGTGACGCGTCACGTCGTAACGTACAGCACGCATAAAGCAAC

TATTTCTGTCTTACAATCTCTCTCCACCAGGTGTAGCACTTCTCTCATCG

TTTAAAAACAAGAAATGGACAGTGACGGGGGTAAGGGGGGATACCTAGTC

ATTTTTTGCGTCATCATTGCATGCGATCATCATTCTCAAAGCCGCTGTTT

ACTTCTAAGATCACTTTAGCGCCGCCCTAAAAACCCGATTCAAATTCGAC

ACAAACCTTCGAATAGGTATGTAATGACACATTATATAAACTCTT

>rnd-5_family-427#LINE/L2 ( Recon Family Size = 27 Final Multiple Alignment Size = 19 )

GCCATAATAACGGCCATCTTTAAATCAGGCGACCCAACGCTGACGTGAGT

AACTACAGGCCCATTAGTATACTACCTGTGGTGTCGAAGGTTGCTGAAAA

GTGTGTAGCAGAGCAACTGATTGCCCACCTCAACAACGGCCCCTTCACAT

TACACTCCATGCAGTTTGGCTTCAGAGCNAAACACTCCACAGAAACGGCC

AACTGCTNTCTTCTGGAANACGTGAAGTCCAAGCTGGACAAAGGGGGCGT

TGTTGGGGCTGTGTTTCTGGACCTAAGGAAGGCTTTTGATACTGTTAACC

ATGAGATTCTCATCACAAAATTGTCCAA

>rnd-5_family-376#Unknown ( Recon Family Size = 26 Final Multiple Alignment Size = 19 )

TATGCCCACGGGGAGCAATTATGGTGCCTGTAAGTGTGACGTAGCCTATT

TAAAACAAGTTGTTTCGTTTCGTTTAGAATTTGATTAGAATTATTCGCTC

TCTTTTTAGGCCTTATCAANAATNAATTTAATCTTGCTTATTGACAACGT

TTGGCATGTCCATGGCTCAGCCATAATGCAATTTACAGTAGGCCTAGCCC

CTATATCAGTGCGAATGCTACAGTATGTACANTATATACAGTATTTCCAC

ATTGAAGCCATGCAATTACTTAGAACAATGTGGACTGCAAACGGGTTCAA

GTTAACGTATTTAGCAAAGACGGGATAGGTCTACATTTTGTTATTTCGTT

TCTGTAGGCCGTTTAACAAACTATAACGGACTAACATTGGAATTGGCGTT

GATTCCAAGGCGATACATAAAAGACCGTAAATACACAACTTGAAGCAACC

ACATATCTAGCCAATGGAGCACGTTCTGATTGGCCAGTGAGGGG

>rnd-5_family-733#Unknown ( Recon Family Size = 21 Final Multiple Alignment Size = 19 )

TTAATATATTTGTAGGGGTTGATATGGTCTGTGATATTGAGTTGTAAATA

AAATACCTTAGAGTACTTAGATTTACATTGTAGTAAATATCTGTTTCTTG

TTAAGGAATAATAGCCTACTGTATGTAGTCATAAAAACAATTAGGCTAAA

TACATTTATTATTTTGTTGGGTAACGGATCGGCTCTCGGAACTCATTAAG

AGGCGGCTTCTATCTTCAATATAATCATTAATTCAGAAGGCTAAACTCAT

GCAGTAAATGAAATTATATTACCAGGTACATTTTACAAGGTCCAAANGTN

ATCGGCGGAGTTAGAAATGTGGCTGTCAGAAGTATTAAAATCAACCAATG

NTAGCTTTAAATGCTCTATTATCCAAATGTTGAAAGTGTGTGGGCGACGG

AAGAAACAAAACGGTGCAGTTTGAGGCCATGTGGCAGAGTGATGCGGGCG

CTGGAGATGGGGGTGTGAGCAGGTGGGTCTGGGCAGGTGTGATGTGGTTA

CTGTATGTTTTGTATTGTCTAAAAAGATCAAATAAATTCGTAGTAAATAA

ATAAGGAAAAA

>rnd-5_family-1504#Unknown ( Recon Family Size = 21 Final Multiple Alignment Size = 19 )

TGCAGGTCCATCAACCCATTTAAATACCTCTCACACCCTACAGTAACCTG

TGGATCATGAGAGAACCTTCATAAATCAGCAGAACGTTAATAACCTATTT

ATTGACACTTGTAGTGTCCTGTAATACTTAGTTTGAAGTGAGACACCTTC

GGTCATTCATAACACATCCATAAAGACTTATATCGCATGACGCTGTACTT

ACTTTAAAGTCAGACCCCTTTGGTAATTTATAACACATACATAAATGCCT

TGTATCGTATGACGCTGTACTTACTATAAGGTCAGACNCCTTTGGTCGTT

CATAACACATACATAAAGGCTTAATGATGTAAACACAAATGTATTAACAC

TTAGGGAATTATCATTAACAGCTAAAACAAATAAACATTAAANACCTGTT

TAAACCATANATTNCCTTTNATTTNTACATNNCTTAAGCAATACAAATAC

ATTAATTTTCAAAAAGTCCAGCAATGCAATTACATTGAACGTTACACAGG

GCTGTGAATAGCGTAGCTTGTCCCTGAGCTGGCGAGACGGATGGTTNTTG

CCTCTCTNCCTGCTGGNGGTACTGCGTGTTGGTTCCAACCTTAAAAGNAA

CAACAAAGAAAGTTAGCA

>rnd-5_family-1593#DNA/Helitron ( Recon Family Size = 23 Final Multiple Alignment Size = 19 )

ATTTGGACATTTTCACTTAGGGGTGTACTCACTTTTGTTGCCAGCGGTTT

AGACATTAATGGCTGTGTGTTGAGTTATTTTGAGGGGACAGCAAATTTAC

ACTGTTATACAAGCTGTACACTCACTACTTTACATTGTAGCAAAGTGTCA

TTTCTTCAGTGTTGTCACATGAAAAGATATACTCAAATATTTACAAAAAT

GTGAGGGGTGTACTCACTTTTGTGATATACTGT

>rnd-5_family-2188#Simple_repeat ( Recon Family Size = 30 Final Multiple Alignment Size = 19 )

AGCCTCCTTCCCTTGCTAGTAGTAGCTAGCTGGCAGCCTGGCTAGCTGGC

TTTAGCCTGGCTAAAAACATAGCAAGCTAGCTAGCCTTTTTAGCTTCCCA

CATCTCCATGTGAAATAATCNGATTTATAAATTTAAAAAATATGCCCTGG

CAAATATTCTTATACTTGCCGGTGTAACCTAAAACATTATCCCAGTTTGA

TATGCTGCTTGTGTATTCATTCCCATATCAGCTAAAAATAGAACGTCATC

ATGTTTTGGTCACGGAGAAAAAGTACATGGCTAGCTAGTTGGCTACAGCA

GGTTCTACCATTTCACAATAGCCTGTAATCACTAGTTATTTACTTCTAAA

CAAAATACGTTTTTGGGTGGATTCTTGTTGTTTGGTTTACTGTTTGAACA

GTCGCTGAGATTATATTTGGTTATCAATGCAAAATAGGCTACTTTGATGA

ATAGCCT

>rnd-5_family-799#Unknown ( Recon Family Size = 23 Final Multiple Alignment Size = 19 )

CTTGGAGGAGTGGCGTCACTGGTTGGAGGGAGCGGAG

>rnd-5_family-1842#Unknown ( Recon Family Size = 24 Final Multiple Alignment Size = 19 )

AGGATAAAGTGACTAGGCAACAGGATAGATAATAAACAGTAGCAGCAGCG

TATGTGATGAGTCAAAAGAGTTNGTGCAAAAAGGGTCAATGCA

>rnd-5_family-8479#DNA/TcMar-Tc1 ( Recon Family Size = 21 Final Multiple Alignment Size = 19 )

GGGGAGAACAAGTATTTGATACACTGCCGATTTTGCAGGTTTTCCTACTT

ACAA

>rnd-5_family-1547#Unknown ( Recon Family Size = 21 Final Multiple Alignment Size = 19 )

CAGCAGCCAGCGGCACTTGCCGCACTGGTAGCCTATTCGCGGGTGCTTTT

TCAAAGCCTATGTCAGATACTCCAGTGAGTGTAATTGGCTCCAGTGAGTG

TAATTTGCTCAATATTAGGAGTTGAGAAATAAAGTTGACATCATTAGAAA

TGATATTCTCCTATTTTCTACTGTAGGTATGTAATTCAAAATGCGTTTAT

TCTGTTGTTGCTAGCGATATTCATAAAAACATNTATTTTTATAATCAATA

TTTTACNANTATTT

>rnd-5_family-3078#Unknown ( Recon Family Size = 20 Final Multiple Alignment Size = 19 )

TGTCATCTCTGCTTCTTTCGCGGAGCAAAGACGTTTAGGGACCGGGGAGA

AAATGCAATAACTAAAAAAAACGGGAAAGATTTTCTGTGCAAAATGTCCA

AATGACATCAGTTTGACCGGTT

>rnd-5_family-497#Unknown ( Recon Family Size = 21 Final Multiple Alignment Size = 19 )

TATGAAAACATATATGTACTATAGTACATATAATACCAAATTGCTGGAGT

ATAGAGCCGAATTAAACGTTTTAGCTTCACTGTCCAAATAAATACGTAGG

GGAGTGTA

>rnd-5_family-2003#Unknown ( Recon Family Size = 21 Final Multiple Alignment Size = 19 )

CAAACGCGCAGACCGGTCTGGAATGGTGTGCTTGTATACAGCTTTTTGAT

ACCATTTATACTTTTCGAACTATAACCGTTTCAAATGTGCATAAAAGCTC

CATAGGCTTGAATGGGAAGCATTTGGATTCGCCACTNCTCTTGAACCGTT

AAAGCTACNAACACCAAANCAACTTTAAAACGTGCAGACTGACTCGACTT

AGATTGCTTGTAAAAAGCTTNTTGATACTACTTATACTTTTCGAACTACA

ACCATTTTAAATTTGCATAAAAGCTCCATANACTTCAATGGCAAAATGTG

GATTCGNCACTGCTCTTGAACCGTTTAAGCTACAAACACCAACCCAACGT

TGACATGTGCAGACTGACTCAACTTACATTGCTTTTCNAAAGATTTTTGA

TACGATTGTACTTTTTGAACTATAGCCATTTTAAATGTGCGTAAACCTCC

ATAGGCTTCAAAGCTACAAACACCAACCCAACGTTGAAATGTGCAGACTG

ACTAAACTAAGATTGATTCAACACAGCTTTTTGCTACCATTCACATTCCA

TAAACTAT

>rnd-5_family-2076#Unknown ( Recon Family Size = 27 Final Multiple Alignment Size = 19 )

TGACATTGGTCTGCATTGATGTCGTCGCATGCCTCATTCATGGCCAGAAG

GAGGGTGGCACGCTCATGGGACGGCGATCGTACACCTTCCACCTCCATGC

TGAAAAANAATCCTCGATAGGGTTGAGGAAAGGAGAGTATGGGGGCAGGT

ATAGGGTCACGAATCGGGGATGGGCCCGAAACCATGCCTGAACCACCTCT

GCATGGTGGAACCTGACATTGTCCCACACANCGACATAGGTGACACCTTG

ACAGGCCTGCTCAATTTNATTGAGAAACACAATGAGGTGTGCAGCGTTGT

AGGATCCAAGTNATGGCCTACGTCCTACCACACCATCTTCAGAGATAGCT

GCGCACATGGAGATGTTTCCCCCACGTTGTCCAGGCACTTGGACGGTCGC

CCGTTGGCCGATGAGGTTCCGCCCACGGCGCCGAGTTTTGTCCAGGTTGA

AGCCCGCTTCATCAACAAANATATACTTGTGATGGCTGACAGCCTCATAA

AAATATATTTTTGTTGTTTTGTTGAAGTTCAAATATGATATGTGAAGTAG

CATGTGCATCAAATAGTAACGGTTTATTTATTTATTAAAATGGATATGGA

TTCTTGGATATGACACT

>rnd-5_family-3300#Unknown ( Recon Family Size = 30 Final Multiple Alignment Size = 19 )

TCAGGCCACTTTTCTCTCTGTAATTGATTACGTTGACTTGTTGTATACGC

ATGCAACCTCCTCCGTCTTACNGAGACTGGACTCTGTTTATCATGCATCC

TTNCACTTTATTACAAATGCCAAGTCACTCACCCACCATTGCACATTGTA

CCAAATGGTAGGTTGGACCTCACTTTATATGCGCAGAAAGATACATTTGT

ATGTGTTCATCTACAAAGCCCTTTTGGGTAAACTCCCTCTTTACCTCTGT

AGTCTGGTCTCCTTCACCACCAGCAGTTACCATACCCGGTCTGCTAGGTG

GTTGCTACTTAAAGTCCCCAGGACATTCACAGTATTAGGCAAGACTGCCT

TCTCTTCTTGTGCACCAGACGCATGGAATAGTCTACAATCCATGCTTCAT

CTAGATATGTTAGTGCCACTGAATGAATTTAAAATATTGATGGGAGACTC

TGTTACAGAGGAGTGTAAATGCTTTTTTTAGGCTGGATCATGTTGTATTT

GTTGTATGTTTTAATTCTGTAATGTATTGATTGTTGCTGCCTTCTTGGCC

AGGTCTCCCTTGA

>rnd-5_family-2185#Unknown ( Recon Family Size = 20 Final Multiple Alignment Size = 19 )

TTTCAGACATAAGGAAGTGGATGGCTGCAAACTTTCTACTTTTAAACTCG

GACAAAA

>rnd-5_family-229#Unknown ( Recon Family Size = 22 Final Multiple Alignment Size = 19 )

AACCAGCCAGGAGGATACAGACAGCTNAAGAGGTATGCTTAGATATGCAG

AAAATNTGACATATTCGTTTACATAGAATTAAGCATAATGATTATGGCTC

TAGATTGCAGGAAAAAGCTGTTTCGGGTGCAGAGACCGCCTCCGGACCAC

CCCCCCAGCCATCCTCACGTACTTTGTGCCCCCTCAGATTTTTGGGGTGC

ATGACGCCCCTA

>rnd-5_family-2472#Unknown ( Recon Family Size = 19 Final Multiple Alignment Size = 19 )

AAGTCGTAAAATGTCTAGCTAGTAATTTGTTATGCTAACNAGCTAGCAAG

AGGTTGCATAGCAACAGCATCAACTTCCGGTAGACAGGCGAAGCGCTAGT

ACGCTCAACTGAAAGGATACCGTTCGTTTACAGTATACTAAAATGAACTA

ATAGTATGTATGTATATACTCATTAAGTATGTAGTATACACGTATGTTAG

TATGGGTATTCGAACACAGCTTAT

>rnd-5_family-399#Unknown ( Recon Family Size = 31 Final Multiple Alignment Size = 19 )

TAAGAAAGAAATNATCCATGCGGGCCTAAGACNAAATGGCCAATAACCTA

TCCTCCTACTAGGCCTATCGTACAANCTTCAAGACAAGTTTAAAAGTTAT

GAACAGTAGCCTATTTCCCAAATATTCTAGCCTANGAAGGTGTTGTCCTA

AAGTTGCCGCTTTCAAAAATAACAAGAATGAAAGTCGGAGCCTAAGCCTA

GGCATAGGCTATTCTCAATCACAGCTTTATGAGAGATNGAACAATAATAT

TTAGGTTCTANTGATTTTGAGGCAAATAAAGCAATTATTATCCAGTTCTG

AAGACTGTAGACTGCTTCTATCCAGCCCATGATGTAGGCTATAACCTAGC

TCACTACGGTAAGACAGCCCGTTCCTCATCAGACTGTCACTGCTCCATCA

TGTGCGCGCCACACAGACAGACACAGACGCACACGCAAACCCGTTCTGCT

CCTCCACCAGAAAGGAATATTAGAAAATATTTGCCATTGCCTGCACTTAG

CCTCTGCCCGGCTTTCAACAACATTATCTNTCGTCATGATTCNTCCAGTT

GCATTCAATTATTGGGGTGGCCAATAGGGTGATAGCAT

>rnd-5_family-2818#Unknown ( Recon Family Size = 24 Final Multiple Alignment Size = 19 )

GAAATAATCTTTCAAGTTGTTTTGGATGCTGTGGTAAATGTGAACAGCAG

CAGACCAGGCACTCTTCTGTTGGGGACTCTCTTCAGATTCAGCCAAGGAC

TTCATATCTGACACGAAAGTCTTAACAACTACTGACGCTGCTGAGCCCAC

TGGGTGAATTGACTCTGTCTTTACAATGCCAGACAATGTTTGACCTGATA

CAGCACCTGTTAACTCAGACTGAACGACTGAACTAGGAAAGCTCAAACTA

CTGACTTTTTTGTCAAGAACTCCACTCACTGCTTGTATTGCTGATGTTTG

AAACTCCCGGCTAGAGAGTTTTTGGATGCTCTTAGATGTGAGCGTGCAGG

AGGAACCAGATTCACTAATATTGTAGCTGCTCTCGTATTTCCTTATCAGG

GCATCAAGATCNTCAATGAAGCCAACATGCGATGCCAAAAACGTGATCTT

GTCCTTCAGATCTTCCAGCAAATTCTGTTCGTTCTCGTCAACAAAAGCCA

CCATTGTGTCAGAGATCGTGGTCATGACTTGCCCTGTTAGACTCTTGCTC

TCTTGGTCAACTTTTTCAAGTTTAGCAGCCAGCTCTCTCACCATGCTCTC

AGTCACCTTGGTCTGTGAGTCTCCCCTTACCGGTGTCACT

>rnd-5_family-753#Unknown ( Recon Family Size = 29 Final Multiple Alignment Size = 19 )

TGGGTGGGGTAAAGGGGGAAGAAAAANGGGAGGTGGAGAGGGATGGGTTT

GGCTTGGGAGAGAGAGAAGGAAGGATTTAACTATACTACAATGTAATTGT

AATTATTTTTGTGACTTGCAAACCTGCTGTAAAATGAATAAGAGTTCTGG

ACAGATTAGGAGAGGATGTCGAGTCATTATTATTCCTTGTTTGTCATTAT

AGCTAAGATTCAATGGTGGTTATTGGTGAGAGTGGAGAGGGGTTGGGACG

GGGTGACTGGGAGGGCGTCAGACACTTCTCTGAAGTATTGTGTGGGGCCT

CCACTCATCTCACTTCAGTCTCTCGGNGGACCCACTCNCCCCAAGTAGAC

CCCCACCAGCCACTATACTTTGATCGAATTTAATATAAACTTTCTAATGT

ATGTACAATGACANAATCCACAGACTTTTGGTACA

>rnd-5_family-2078#Unknown ( Recon Family Size = 20 Final Multiple Alignment Size = 19 )

CAAAGTTCTGCCAAACGAAAATATGTCTAAGTGCACGTTTTTGCGTTTAA

TTTATGGTTTATCATGCATCGTCATTCATCACCATATACAAAAACGTAAC

TCGCTGCCATCCATTAGCTATAATTCTGAGGTTGTAAAGCTGCAAAACTA

GGACGTGCAATAAATTATAACCACTTGCAGAATAATTTCAAATCAAAGAC

GTTCNGCTGCAGGGGGCTTGANCCGTTTCTTGGCGCCGNGGGACGGTCCG

CTGCATAGAGCTGACACGTTTCTTGGCGTCAGGGGACNGCGCCATGGTGC

TGAAATGTTTCGAGTCTGGGCAGCACCTGAGACCGCGGCATGGTGCTGAA

ATGGAGCAGGGCTCAGTTCTATGGATATCTCATGCGGATTTTCCTGCTAG

CCTATGTATAACGATACTATAATTACATTATTTTCATCCATCCGTCAACA

CAAATGGCATGTCAACGTTCCCCAAATAATAATGCAATGAAGCCAAGCCG

AGATGTATGGTTCTTCATCCAGAGGACGAAGCACCACTGTATCATCTAAA

ACCTTCAGAGAGATCCCTTAAAACTGTAGTATAAATTCTCCACGGCTGTT

ATGGTAGCTAGGTTAATTTAGCGAGACATGGACAAATCCAAC

>rnd-5_family-639#Unknown ( Recon Family Size = 32 Final Multiple Alignment Size = 19 )

AAATAAATGTGCAATCACTCTTGAGCGACTGAAACCTCTTAGATGTTAAG

TCCCCTGTTTAGGGGACCTGCGTGGTTCTGGTACCGGTTCCGACCGACAT

TTTCGCTTATAAATCGATCTGTGGACACCGTATAGATCGAAATGGCTTGC

ATTGAGCGGTAGCCCTGGTTCTCACGGACACAGGAGTATGTNTCTTCTGC

CTGTGTGACGCAGGATGTGAAATCTGACACCACTTGAAGCTGGGATGTCC

CTCCTACCATTTCATTCGCTCTTCCGACTGTCCATCAACTCCTGGCTGAA

CCCTACGTCACAGCCACTAACAAAGCACGCGNCTGGAATCCTTACATCGT

AACGCAGCGGGAGAGAGTGGTTTCGTCGCTGTAGCACATTCAGAGATCGA

TTTATAGCCAGTAATCTAAAATAATTAATAGATTTTAAACAAAAAAACAC

TTTCTGTTTGTCATAGACGGACAAGATTAATATGAGATGAAAGGCGAG

>rnd-5_family-4606#Unknown ( Recon Family Size = 20 Final Multiple Alignment Size = 18 )

CACTCCCTTNAGTGCTGTGGCCACCCTTTCCTGCTGTGCTCTCAGGTCGT

TTGTGCCCGTGTGTATTATTATGTGGCTGGGTGANCCTAGTTGGTCCTCA

GACAGAAGGTCTAGGGCGCGCTGGGTGTTTGGACACCAGAGTTTAGACAC

ACTGTGTTTGGGAAAAAGTTTATTTTCTTGTATATATTTCCCGTTTGAGT

CCATAAGGAGTACAATCTGTGTCTTGTGTATGTCCTCAGTGGGTGTGGGG

GGGTTGTCAGGAGGGCTATCAGGGTGGCTGACAGGGGGGTGCTCAGAGGG

GGTGAGATCCCCTGGGCTTGGGGTTCTTCATTTGTCTGTCCCGCTGTGAT

GTCGACGCTATGGTCAGGGTCTGGGGTGGACTGTTCTGCTGTGGTGTCGA

GACTTTTGTCGGGAGCTGAGGTGGGCTGTTCTGCTGGCTTCTCTGCGGGG

GTGGCCACCTCTCTAGTGGGTTGTTCTCTGTCACACGCCATCCCCCTCAC

CCTCCCCTCNAGCAGTCTGACCCTCTCCTCTAGTGCTCTGTTCTTCCCCT

GCTCCTGCTTCTTATATTGTTGAAGTTGTCTCACCACNGTCCAGAGTGCA

GATATGTCTCTCTCGANCTCCAGC

>rnd-5_family-273#Simple_repeat ( Recon Family Size = 19 Final Multiple Alignment Size = 18 )

TTTGTTAGGAAGAAATGGTCTTCACACNNNNNNNNNNNNNNNNNNNNNNN

NNNNNNNNNNNNNNNNNNNNNNNNNNNNNNNNNNNNNNNNNNNNNNNNNN

NNNNNNNNNGNNNNNNNTAAGAAATTCATGTTAGCAGGCAATATTAACTA

AATA

>rnd-5_family-2371#Unknown ( Recon Family Size = 33 Final Multiple Alignment Size = 18 )

GAGGCGCGGGAGCCTGGCGAGCCGGCTGAGGCGTGGGAGCCTGACGAGCC

GGCTGAGGCGTGGAGCCTGACGAGCCGGCTGAGGCATCCTCGGTGGGCCG

GCGGCGGACGCCCGACGAGCCGACCGAGGCGNGGAAACCTGNCGAGCCGG

CTGAGGCGTGGAAGCCCGACGAGCCGGCTGAGGCACCCCCGGTTCCTTCG

GCGGCGGCACCCGGACCCGACGTCACCAACCAAAACAAAAAACAAAAACA

CTCCCTGATGCTTCCCTTCGGTGAGGCGTTATTCTG

>rnd-5_family-2563#Simple_repeat ( Recon Family Size = 20 Final Multiple Alignment Size = 18 )

AAGGTTNCATAGCCTTNCCTACTAGCTATACTAACGATCNGCTGTCCAGA

GGGNAACAGCAAACATATCAATGTTTTCCCGCAGTAAAGTAAACACACTG

TTGACTCGAGAGGATGCTAGTCAGTCCATCTCTGAAACCTCTCAAAAATC

CAGACAAAAATAATNCAGCTAGCTAGGGTAGCTACAAGTCAAGTAGTACT

TGAAGTAGGCTACCCACCCCTCACCCACAGACACAAACAAACAATCATTA

TCAAGAACGCCTAGCCCTAGCTTCACCATCTAAGTTAGCTAGCTAGTTAA

CTAGCAAGCTAGACTGTTGGTTTATAAAAGCCAAAGAAAGCTAAACTCGT

TTTTATGAATTTCTCATGNATTTAAATGGCTAGACAAACAGACGGCTATG

CCATCTAGCCAGGCTAATGTTGCTAACTAGCTAACCATGAGCTAGCTAGC

TAGATAACAGGCATTTTCAATTTAAAACTTGCACAAGACAGTTCACAGAA

TTGCCCATTTTAAACAACTTTAGCCAATTTATTAATTACNAAATNTAGCT

AACATTAGATAGTTAATCCAGAGATTCTTACCTTTGCCTCGATTCGGCAG

TCTCGTCCAGATCATCATGGCCCTGGTGAGTCACGT

>rnd-5_family-2751#Unknown ( Recon Family Size = 19 Final Multiple Alignment Size = 18 )

GATTTGTTTTCTAATGACATTGACAATTCATATANATATTAGACTTAGGC

CTAATTGTGACATCATGTGTGTATGACATCTTTGAAGAATGCTCTGGCTT

CAGCCAATCGGAATCGAGTATTCAACAATGCTTTGGCATAATTCAAGGAC

GTTCATATTCACTTCAGTTGTCTGCCGATGGTTCACTGACGTTTTTTCAA

AGGAGGTATTAGTATTTCCTCTAAACAGCCTACAACAATAAGCTACTCAT

CATGTCAGATGACAGCAAGGTATGGTTCATTTGCCTATTTTGAGTTTAGA

GGAGAAATTACTAACAATATAGTAGGTCACTAACACAGCAATAGGTCTCA

CTGGTTTGATCCATGGCCTGAAATCTGACCACTATTGCCGTTTTGTTTAA

AATAGGAGCGAATAAGTTGTTTCATAAAGTCACAACCTAGGACTGATATT

GACCTCTTATTTATCCACCAGAGCGTGAAATGGGAGGATCCCCCGGATGA

CCAAAATAAAGTCGTGGAGAACATGAAGGATGAAACAGAGACACATGAGA

CCAACAAGAACAGGACAGGAGGCAAGGTAAGAAATATGCTGTCCTTTACA

CATGCTCTG

>rnd-5_family-1720#Unknown ( Recon Family Size = 26 Final Multiple Alignment Size = 18 )

NNNNNNNNNNNNNNNNNNNNNNNNNNNNNNNNNNNNNNNNNNNNNNNNNN

NNNNNNNNNNNNNNNNNNNNNNNNNNNNNNCCCTATCCCCTCAGCCCTCA

AATTAAGTGGACACTTCTGATGACGTATCATGACGTCTGACGAGTATACA

CTTGCAGGGCAAGGGGCGAGGGAGGAAGAAGAAATGATTTTTAAATGGAC

CACCCTTGGCCGGAAATTCGTCACTCGTTCGTCCCGCGATGATTGTGTTT

TCAGCCACCGGTAGCNTGTGGGTGCTTTATATGCGCTACAGTCAATTATG

ANTGCATGTCTACTTATATTAANTATATAATTATTAAT

>rnd-5_family-1403#Unknown ( Recon Family Size = 20 Final Multiple Alignment Size = 18 )

GCAGTTTGAGCTACTCCAGGAAGTGACGTTGCAGGCTAGCTCAGTGCTGC

CCCCTCTCATTGAGTAACTCAAGTTGAAGGCCGACCGGGGTACTGCAGGC

TGCCATTAGCAACTAAATTATCCTTATAACTTCTTCTGTGTGCGATTTTA

AAATAATCGGTTCAAGGACATACGTCTGGTGTGTTTTTCTCAATATGTAT

TTNTTTACACGAAGATCGACCACGAAGATCGCAATTTTTCCATTCACTAT

AATGGGGGATCCTGTTTTCTGNNAACAATGCCTGCAGTACCGCGGTCGGC

CTTGAACTTCCGCGGCTTCAATGAGAGNGGGNAAGTCGTTCTCCCCTG

>rnd-5_family-9007#Unknown ( Recon Family Size = 18 Final Multiple Alignment Size = 18 )

GTGCTGTATTGACGTATCCTGAAAATGACATCTGCTGCTTTAGATTGAAC

GGTCATATCTCAGTTATTGTTTTCATATCTACAAAAAATAAGCTATTTTC

TTTACTTTCAGAGNGCGAGCTGATCAAGGGGTCGAAAATGTAGATATTGC

CAGATGTTCACTGTCCGAGGAACAGGCCGTA

>rnd-5_family-2911#LTR/Gypsy ( Recon Family Size = 22 Final Multiple Alignment Size = 18 )

CAGGGTCCTGTCTTCCCTATGTCAAGGTAATCGATCCATAACAGACTACT

CTATTGAGTTTCGCACTCTTGCTGCCTCCAGTGACTGGAACGAGCCGGCT

TTGCTCGCTCGTTTTCTGGAGGGTCTCCGCGCGGAGGTAAAGGATGAGAT

TCTCTCCCGGGAGGTTCCTTCCAGCGTGGATTCCTTGATTGAACTCGCTA

TTCGCATAGAGCGACGGGTTGATCTTCGTCACCGAGCTCGTGGAAAGGAG

CTCGCGTTCTCCGTTGCCCCCCTCTCCGCATCACTACCATCTTCCTCTGC

CGGCTCGGGTGCTGAGCCTATGCAGCTGGGAGGTATCCGCATCTCGACTA

AGGAGAGGGAACGGAGAATCACCAACCGCCTCTGTCTCTATTGCGGTTCC

GCTGGTCATTTTGTCACTTCATGTCCAGTAAAAGCCAGAGCTCATCAGTA

AGCGGAGGGCTACTGGTGAGCGCTACTACTCCTGTCTCTCCTTCAAGATC

CTGCACTACCTTGTCGGTCCATCTACGCTGGACCGGTTCGTCAGCTTCCT

GCAGTGCCTTGATAGACTCTGGGGCGGAGGGCTGTTTTATGGACGAGACC

TGGGCTCGGGAACATGACGTTCCTCTCGGACAGTTAAGGGAGCCCACGGC

CTTGTTCGC

>rnd-5_family-3909#Unknown ( Recon Family Size = 21 Final Multiple Alignment Size = 18 )

CACTACAGACCCTGGTTTGATTCTTGTTTAGATCCCGGGCTGTNTCACAA

CCGGCCGTGATCGGGAGTCCCATAGGGCGGCGCACAATTGGCCCAGCGTC

GTCCGGGTTTGGGG

>rnd-5_family-397#Simple_repeat ( Recon Family Size = 27 Final Multiple Alignment Size = 18 )

AGCTCGTTTTGTAGTAGAGGAAATGCTGCCCCTNTCCACAGTAGAATCTG

TCATTATCAGGCTACTTGCGTTCGTNTANAGGCCTACGTAATGTAACGTT

ACGTTGACATTGTTTGCACAACAATTTGGGGTGTGACGCAAAAGTANCNC

AAGTAGTGTAACTAATTACTTTCCCAATGGAAGAAAAGTAATTTGTTAAT

GTTAAAAGAAGTAACCAAAATATCTGCTCCGAATTAAGATTCAAAGATGT

CTGCAGAAAGAACGGGGTGTCAGCTATGACGTGACACCTTATTGAACTAC

AGTAAGTGAAGTGGATTTACACCCGGTAACGGAATTACATCATGGGTCCC

TGATCTGTACTATACAGAAATGCATAATTATGTCATTCTCTTCATGGTGA

TGTACCTGAATNGGTACACAAAGGTAGANANNTGTGGGAAATATCCTCCT

TTGCATGTTTGGGTATTATTCTACACACTGGCTATTATTCTAATGAGCTC

CGCCCCCAAACTAGACCACATTTGGTCGGTCCGGACCGGACCAAATCTGA

ACCAATCATAGACGTCTATGTTTCACGAGTTCGGACATCACAGTACAGTA

CAGCACAGTAGAGCGCAGTANANTACAGCGCAGTAGAGTTCAGTANAGTA

GAGTACAGTANAGTACAGTANAT

>rnd-5_family-1152#Unknown ( Recon Family Size = 21 Final Multiple Alignment Size = 18 )

CCAGCGTCCCACATAGTTCTTGACTTGATTTCTCTCGTGAATGATTTGAT

TTCTCTCTAGAGAAACGGCGCGTTGGGCTCGCAAAAAACAAAAACTAAAC

TAAATGGAATTCAAGTAATTGAACCGACGTCGGTCAATTAGTTGTTTTAA

AAAAAGGTAACACCACGTGT

>rnd-5_family-808#DNA/Maverick ( Recon Family Size = 28 Final Multiple Alignment Size = 18 )

GATGTGGAATTGGAGACAAGTGGGAGTGGAGTTGCTGTGCGGGGGATAGA

ATGACGGTCAAAGATAAAANTAAAAAATAAAGTCAAAATAAAACATAATA

AAAGTAAGTTTGAATGACACTGAGGGGCAGTGTTTTTACAGCTAATGCCG

GTTTGCCTGAGGCTGATGCCGTGCAGGTGTTTGTACACATGCATATACAC

ACACTCTCATTCAAATGCACACACGGACGCACACGCACACAAACATATAC

AGTTGGCATGCTGTTGTGATTTTCGTTGTCCTTGATGTCTTTTGTTTTTT

GCATTGTTGTTTTCTGTTTTCCTTTGTCTTTCTCTTTTTTTCTGTTTTGT

TCATTTTGTTGGTTGTTGGTGCATTGGGTGACGGGGGCTTGGGGCGG

>rnd-5_family-743#Unknown ( Recon Family Size = 28 Final Multiple Alignment Size = 18 )

TACGTCTGTGTACAGGAANGTATTATTTGTAAGACGTAAGAGAAAATATA

TCNGTTTATTGTTAAATTATTNCGACGTTCTTTCCANCACAAAAAGTGTA

GTAACTATTGTTTCCAAACTGCGTTACCCACTCCAGCCAGCAGATGGCGA

TNTGCGTCTTTCAGGNGATGCTTCTTGCGTGACGTATAATGGACTGGACG

GGACGCTTCTTCAGGAACAACAACACGGCTACTCTTTCAACCACCTCGGT

AGCTTGCTAGCCAACATGAAAAGAACAGATTGACGCTTTAGACCGTGTTT

GTGTACATTAGCTAATCTCAGTGTTTTAAACACATTTCTGTTGACGTATC

AACTGGTTAACTTTAACCTAATTTGCTTGTTCAATTTGCAAACTTGTTAT

AAGATTGATACTGAGCCTAGCACTAGGCTAGTCGCTAATGCTAACTAGCT

AGCTAACATCCCCGACCATGAGCTCACTAAACTACTCCTCCCCTGCTAAA

GAAGAGGTGGTCTGCTGGACGGAGAAAGAAGCTCTGGGACTGAACGTTGT

CGTGGAAGAAGAGGCTGTTACAGTGAAAGAAGAGGANGCTNTCACAGTGA

AAGAAGAGGAGGCTGTTACAGTGAA

>rnd-5_family-488#Unknown ( Recon Family Size = 40 Final Multiple Alignment Size = 18 )

TGTATAAAAGGCTATGNGCATTGTNTGANCTTGAGAGGGCAGAGCTCTCT

GAATAAAGAACTGATCATTTGTATGCTGGGGCTCTGTTTATTAAAACTTA

GAGCCTTACAACCTCTAGGATACAGATGGAGTTATAAAATAGTTCAGTTA

GAACATTGGGATAGAAATTCTCGTGACAAAGGGGTTAGTAACTACACAGA

CTCATTTCATAGAACTTCAAAACACTGGCAGTTTGTCTACTCCACTTCTT

TAGTCTACNCTCTGATCACTCCAGATAGCCCAGTGAATAAAACAAATGCT

GGCAATACTGTTGTCATCCATACAAGTCTTAGTAGCATGAAATAACGTCT

AC

>rnd-5_family-1299#buffer ( Recon Family Size = 22 Final Multiple Alignment Size = 18 )

TTTAAGGATTCACATGTGAGGCCATGTACTAAACAACCAAAGATTTCAAG

ACTAAAGGCTGGTTTATACTACGTCTATCGACAGTTGTCACAGTGACATC

GTGAACATTCTATTGTCGTCCGACATCAAACTTGTCGTTGTCGTTATGAA

TAAGTATAAACACAAAAACGACAGCTGCATNACATCAGCAGCCTGGTGGT

CCAAAATAGCATAACTAGTGTATTTTAACACCAATAAAGGTACTATACGT

TAATCTATCTAACCAGGCAACTAAAAGCAACTTTCTAAACAATGTTTTGG

TTTCGTTTCTAGCTNGTTAGCAAGCTAGCTAACGTTAAGTTAGCTGGCTA

GCCAGTTCAAATAATGACCGCGTCATATAGCTGACGACGTTTTAACTTNA

GCTAATTTGTATTCATTATTACAGGAAAATAAACTCACAACGAGATCGTT

ATTTGCAAGTTAATGGCGAACTAATTACAGAAAATAGCTTACGGTCTCGT

TGGCCTAACGTTATATCCTAATTTGACTTTGGTGCAGGTCATGTTGTTCT

TCACATTACCGTCTCTGGTAAACACACACGCTATANAAAATAAAACTATA

TTAATCACGCTATATCAAAT

>rnd-5_family-1841#Unknown ( Recon Family Size = 27 Final Multiple Alignment Size = 18 )

TCTCGGGAGTTGATAGGCTTGAAGTCATAAACAGCGCAATGCT

>rnd-5_family-357#Unknown ( Recon Family Size = 19 Final Multiple Alignment Size = 18 )

TAATTGATCGTTAAGCCATGTTTGTCAGGTAAACTAAGGTCATTAGTGGC

CTATATAAGCCTCATACAGGCCATGGGAAGACATTACCTGTTGATAACAC

AGACTATTTTCATGATCATGGGAGGTGTGAGAGAATTGCTGCTCGTTGTG

ATGACTGTGGGGGTTGTCAAAGGTTGGTTCACTAATGTTTAAGGGATTTG

TTTGGTAAATATGCTTAACTTTATAAGTTGGGTATTAATATTTGACTGTC

TGTATCTTCCGCGTTGGTCATCTTTTATTCTTCACTGCTATTAATATATG

TAGCTATTTTGTGTGAACTCAACTTCTGTCAACACTTTCTCCTCAGTTTC

TTGTTACCCTGTTGGAAAGTCCCAGAAGCAAGATCAAGTCTCTCTGCAGA

GGAGACTTGGAGGTAAGTGTTTCTTTCCGCAACCCTTCTAGCTATGTTCT

TTGTCACTGTCTATGGTGCTGCTGTGTTTGACACTCATTCAACAGCATAG

AGTAACTCAGTCTAAATAATGTTGTCAAACCTTAACCTACATTTTTCAGA

GCTGTCATCAAATGACGTCTCCATTGTGCATGCCCTGGCCTTGCTCCGAT

CCATAGGGTCTGACGCTAAACAAGCCAGAGAAGGTACGGCC

>rnd-5_family-88#Unknown ( Recon Family Size = 19 Final Multiple Alignment Size = 18 )

TTTGCATATCCGATTCGAATCTGTTATTTTTCCTGCAGTCTGAACAGCCA

AAAAGCACATGGAATCGGATATTTCAAGCCACATTTCAAACCACCTTCGT

ANGGGGGTTNNAAATCAGATACAAATCTGATTCCTGGCCATGCGACTTGT

GTCTGAACGGTCAAATCTGATTTATTTNCCCTCAAGTGGTTTTTAGACTG

TTATTTGGCATATCTTGTTGCTTGCTAGCTACTCTGTTGACAGTTTGACA

AGAACATGTGGTAGCTA

>rnd-5_family-2634#Unknown ( Recon Family Size = 20 Final Multiple Alignment Size = 17 )

CTCACATCCAGGTACTTTCCGCAGGTGCTGGAGTTGTAGGCAAACTCGTG

GAAAACAGAAAGGAAAACGCCGCACACTGCTGCTCATGCTCCCAGGTGAT

ATTTATTTACAACAACGTTTCGACCCTTAGGTCTTCATCAGGCATCCATA

TCCCAGGTGGGGGTGGAAGGTCCTATATATAGGGGCAGTACTCAGTGACA

TCACTTCCTGAAACAGGAGGAAAAATATATAACGTAGGTTCACAATATTA

ACATTCAATGCATCAAAATATATGATCATACACAAGGAACGTGATCAATA

TTTACAGT

>rnd-5_family-2034#SINE/Deu ( Recon Family Size = 18 Final Multiple Alignment Size = 17 )

GCTGATGTGTGGTGAGCGTTCTGGCGCAAAAATGGTTGCCGTGCATCACC

CAGGTGGGTGCTACACATCGGTGGTGGATGAGGTGAGTTTCCCCCTACTA

TGTAAAGCGCTTTGAGTACCTCAGTTGGTAGAAAAGCGCTATATAAATCC

AATAATTTATTATG

>rnd-5_family-4431#Unknown ( Recon Family Size = 22 Final Multiple Alignment Size = 17 )

GCCTACACTTTCTCGTTCTGAACTTCTAACGCGGGTGGGACGGGTGTGGC

TTCGTGACAATGATCACAAGAGCAGCTGCTCACTAATTTGACATCTCCAG

CGCTGTTAAACCTGCAGTTGTCGGCTACCGCGGGGTAACGCTGATCTAAT

TGATCCCTGGCCTTAGTCTATGTATTAAACAGTGTACTGCTGCTGCCCTC

TAGTTTTCGGACAAAGTAACATAAAAACATTTGTCCTCAATCTCTTCCCT

TTCTATTTCTGTCTCTAACTATTGTTTTGTTTTCTTCTCTTCCTGTCCCT

GTAGGCTGTAAAGTAGAGTCTGTCTTTTTGAACGTGGAAGCTGTGAACAC

CCA

>rnd-5_family-2555#Unknown ( Recon Family Size = 21 Final Multiple Alignment Size = 17 )

ATCGGCGAGGAGGGCACTCACCGCTCGATCATGTTGTCAACAAGGCAGCA

TAGTGCGTGTTCCAGAAACATTCAACATAACTTTTCCATGAAATATATGT

TTGAATTTTCTAAGTCGTTTTCATTGGAAAGGCCGATAACGCGTTTTCAA

CAAATAAAACAATCACTTTTGCAGGTGAAATCAGTGAATCCTACTCATTA

CTCGACATACATAATTACGTCACTTTTGTTTTGAGCCGAATTTCGCCGTG

GGCTTTGAACGGTGCTCACGCCCGGGAAGCAGTTCGATTCCAAATTTCAC

CCGGTGCCAAACACCCTAACCGGGCGCCCGGCCCAGATGATTCGAATCCC

CTCAATGTCCGCAAATCTGATGCGCCTAATAACCATAATAACCAATCATA

TTACCGTCCGCTACCAAGTATCCAGAATTGACCAATTAGAGAAGAGGGTG

AATTTAGCATCGGGCT

>rnd-5_family-1699#Unknown ( Recon Family Size = 18 Final Multiple Alignment Size = 17 )

TTGGGAGTATGGCTAGACGGTACACTGTCCTTCTCTCAGCACATATCAAA

GCTGCAGGCTA

>rnd-5_family-3173#Unknown ( Recon Family Size = 18 Final Multiple Alignment Size = 17 )

ATTCACTTCCATTTCTCCTCCTGTCTTCAGCTCACTCTGCTTACACTTTC

TACCATTCTTCTTTAACAAACCATCTCCCTTTTTTCCGCCATTTTTAACC

GACTCAAGCTCACCCTCTTCCTCCCTCTCTCTCTCTCTTAGACCTCCTCT

GCCTCTCTTTTTCCCTCCATTCCTCCTCCCGTGTTCCAAGTATACGTCTC

CTTATGATAATACTGCGCTTCTCCTGACATNCATCTTGTTCTTGCCTTCT

CAAGGGACGCCA

>rnd-5_family-7755#Unknown ( Recon Family Size = 20 Final Multiple Alignment Size = 17 )

TGTGTTTTTGTATATTCTCATATGGTTAAACGGCACCCAAAATACATTTG

CAATTATAGAAAATCTATGTAAAATATTTGATTTCATTTTAACAGGTAAT

AAATATTGTGTTTTTGTAGTTATACTTGAGGCAGGGATATCAGAACGTCT

ATCACAGAACTGGACTATTTGACATACTGTACAGTACATGTTACAGTGCA

CATACCACTGTAGACAGGTGGATTTGCTATTTTCCATTCATGTTCTCGCC

CAAGGAAAGAAAACAAAACAGAAGAAGAGTTTATGCCGCAGAGGATGAAC

GCCATAATCTCTCCTTCTGGCACCACTTGACTGAGAACAACACTAATGTC

TGTGTGCTTAGCCCATCACTGTCATTCCAGCAGTGCAGCCATGTTGTTTC

TGACGGGTCCTTCCCATAGAGGACTCATCTTTATGTATCTGTGCCATTAT

AGCGTCTGTGACAGCATGGGCAGCGCCATTGAGGCCATCTCCATGTTTTA

AGT

>rnd-5_family-618#Unknown ( Recon Family Size = 18 Final Multiple Alignment Size = 17 )

CTAGCTAGCTAACGTTTGCTCTGGTCCAACGTTAAACCAACTCGG

>rnd-5_family-4171#Unknown ( Recon Family Size = 17 Final Multiple Alignment Size = 17 )

AATACATCGCAGTCAATGGGAAAAGATGAGTGTCAACCAGTTGACGCTTA

TGTCAATACATTGCATTCAATGGGAAAAGATGAGTGTCACAGGGTTGACG

CTTATGTCAATACATTGCAGTCAATGGGAAAAGATGAGTGTCAACCAATT

GACGCTTATGTCAATACATTGCAGTCAATGGGAAAAGACGAAAAGATGAG

ACTCACAGACAGACAGGCAGACAGTATGNCTCTCTCTCTGTCTCTCCCGC

TCTCTCTCTATTCTCTANCTCCCCCTCTCTGTCATCCCCGTTACTNNTAT

TCTCTCTCCCCTCCTCTCATCTTTCGCTCTCNCT

>rnd-5_family-4504#LTR/Gypsy ( Recon Family Size = 19 Final Multiple Alignment Size = 17 )

TGCAGTATCTTAATGCACGTAAAGGGGGCCCACCACGACCTGCAGCACCC

GCTCCAAGGAGTCTCAGAGACACAAGGGACATCAGAAACGCCAGAGATGG

TGGAGGTAACTCTGGGGGTTATGTGTCTGGGAGGGAGGTAAGGGATCATG

CAGTTCGCTCTGATGGGAGGGGTCTGACCTGTTTTTACTGCCGGCAGCAG

GGGCACAAAGCTTCAATGTGTCCGCTACGTAAATCCAAGCTCTCAGGTTA

CTGTTATGTACCCAGAGAGGGGGATGGTGTTCAGAATAGACAGACTCGGG

AAGGGTCATGCTTGGTACCTGTAAAAGTGAATGGTAAAAGTCTTACTGCA

ATGATTGACACCGGCAGTTCCCTGTCGTTGATCAGAAAAGGTAATGTACC

TGTTAATGACATTGATTACGGTCATCAGACACTGATCCAATGTGTCCATG

GTGACCAGTCACAGCAGCCCACAGCTGAGCTCACAGTTGAGATTCAGGGT

CAGAAATACCTCCTCAAAGTTGGGGTAATGGAGAAGCTACCTTTTGAGAT

GATTTTGGGGAGGGATGTGCCTGTACTCTCTGATCTGTTGGGAAGTGTGG

GGG

>rnd-5_family-175#Unknown ( Recon Family Size = 19 Final Multiple Alignment Size = 17 )

GAGTTATTTCAGAAAATCACAGAAAATCACAGAAATCTCGCAGAGCTCCG

CAGCACACTTTAAAAAGATTCGTATGAACACCCTGCAACTGGATCTGTAA

CCGTTTAAAAAAAAAAACGCCTATTTTTGAACATCACCAATTTGACATTG

TACGATTTCTCTTAAACGACGATAGATAAATGNCTGGTTCTTTTTTTATT

GACACCGTAGGTTCCTTTACTTTGACGCGAAGCGGAAAAATTATTGCTCT

ATTTTCATTTTNGACCTTTAATCCCAGAAAAATGGCCATAACTCAAAAAC

CGTTGAGGCCTAGACGCCATCTTGTTCGGGGCCAACTGCCCATTATGCCC

GCTTTGAGGACAATACAGTGCCACTG

>rnd-5_family-892#Unknown ( Recon Family Size = 18 Final Multiple Alignment Size = 17 )

GCCCGGGGTTGATGATAATGAATCCCGTTCAGTGACGCCTAGAAGGCCGG

CGACGTAGACCTCCGGAGCTGGTGAACGGAATGAGCAGCAGTACCGGGGG

ATCCGTG

>rnd-5_family-3427#Unknown ( Recon Family Size = 18 Final Multiple Alignment Size = 17 )

TGCCGAGTCCCCCAACAACCGGATGTTCCGGTTTTCAGGGGAAATGGAAA

GAGAGCCTGTGACGCGACTTCCGCTTTTGGACGTTAACAAGGCGACACTC

CATCTTAACTCCTCCTCCACATTTACTGGATTGGTTGAACAGTGCAGAAG

AGAACCTCCCCCGACAGTTTTTTTCTTCTTCTCTCGTCAAGACCAGNATG

TAGGGGATCTAGTTTCAGGCGTTTCTTTTACGCCTGCTACGTTAGG

>rnd-5_family-1027#Unknown ( Recon Family Size = 18 Final Multiple Alignment Size = 17 )

AACCCGAAACCTGCCCCTCCGCCTGCCCGGAAGCTGGGCCCGCGGTTTGT

GGGGCCGTTCAAAGTCCTGAGGAGANTGAACGAGGTGTGTTATAGATTAC

AGCTCCCCCTGATTACCGTATTAACCCCTCGTTTCATGTGTCTCTCCTCA

GGCCGGTGGTGGCTGGTCCNCTNCAGGAAGNTGAGGTGCG

>rnd-5_family-368#Unknown ( Recon Family Size = 21 Final Multiple Alignment Size = 17 )

CTCCTTACCCCCTAGCAACAGATGTGGGATACACACACACACACNCACAC

ACACTCAACCCCTTTCCCCCACAAACAACCACAGGCTCAGATNNTCAACA

GTTGTNCCATCCCAGAGCCCAACTCAAGAAAGGNCTTGATTTGCGAATGC

ATATACAGTTGCAGCTGTTTGAGAAGGCATGTTTGATTAACCAATGTCAA

GTCCTAGNTGATGGAGGTCAGATGTACCCCTTTCCCCTGAAACACACACA

CGCCGGTCNCCCGTTGTGACCATTTTCCCAAGCATCTCCGTGCAGTCAGA

CCTTTGATTATTTTGTGCCACCAGGGCCACAATAATATGCCCCCTCTCTG

ANTGTCCGAGTGCCCCCCCCCATGGGTTACTGCAGCTNGTGTTGCCAGCA

CTGCCACTGCCTGGGTGGGGGCACCCCACCGGAATCCCCACCGGCTAGGT

ACAAGGTCGTCAAGGTTCTCATTAGCAGCTTTGAGAATTTCCTTGTATAT

TATGCTCTCCCATCGGGGGGCTCTGGCTTTGCAGGACCAACCCTGCCAGG

CAGGCTCAGAATCTTGAGGG

>rnd-5_family-917#DNA/Tc1 ( Recon Family Size = 18 Final Multiple Alignment Size = 17 )

GAAGCTTCTGATAGGCTAATTGACATCATTTGATG

>rnd-5_family-1418#Unknown ( Recon Family Size = 19 Final Multiple Alignment Size = 17 )

ACTCGGTTTAGANCGTAAAATGCGTTCCGGTAACCGGCTGGCACCCGGCG

CTGGACCGCACGCATTAAAAATAGACCCAGGGAAGAATCTGTTATCATGN

AGGTTACCCTACCGACCAGTCATGGGTACCGGCGACAAAGCGCCTCGTAA

CCTAGCCGGGAATGGGACAGACAGCTCTGAAAAACGGACCCCTTCTGTGG

TAACAGACAGGGGCGATTCGTAATCGAATCTAATTCCCAGGAATGAAAAG

CGCTGAGCTGGAGACAGACAGCTCATTTNGGGGTTCATATGAACCACAGA

GACTGTGTGTGGGATAATAGCATGGTCGTGTCCAACCCTGCTCGTTCCCT

CGACTCCGCTACCAACAACCAATCGCCTACAGAGCCCATAACCTGTATCA

CTAGACACCTCATAGGGACTAGTGAGCTACAGGCAGGAATCGGNAATGAA

TCCCAGTAATGAGCCACCTATGGGGGAGGGGCGCCCCCTTGTGGACAGAG

AGGAATCCCCAACTTATCACTCTCACCCTGGGGAGATTGATGTGCCCCTT

GAACCCCGTGAACACCGTGGAACGCGGGGTAACAGAGGGGGCATTAA

>rnd-5_family-1202#Unknown ( Recon Family Size = 30 Final Multiple Alignment Size = 17 )

CCTCCCTTCCCCATGGGCTAGGTCGGTCTTCTGTGCGCGGCTCTGCGGCC

CATCCCGTGCCCCCTGCCCTCGTGCCTTGAACCTCGTGCGCTTTCAGTGG

ATACAGCTGGAGAACTGCAAGGCAGTCCCATTGTGCGCCACTCGGGAGCC

ANGACCAATATATATTGTCCTGCTAATAACAGGGTTAATAATATATCTGT

GAGAATATCCAACGGGCTTTTATATAATTCTAAATNAATAATAATAACGA

TATTTTGGAGATGATTTAGTTTCAAATAACGTAAAGTGAGCGAGAAAAAG

GCCATTCTTGAACATGGGGTGAGACATTGTTACTAATTTGTAAATAAAGC

CAGTTTGTTATTTAGAATTATTTCTGTTTTTAGAGGGAGAAAAACCTACC

GTCGTCTCATGGGTC

>rnd-5_family-1917#Unknown ( Recon Family Size = 25 Final Multiple Alignment Size = 17 )

CAGGGCTCCAGACCGCGANCATTTAGTCACATTTTGCGACCCTTTGACTT

GGCTGTGCGGGATAAACGTATATTAGTCGCACCGGTGCGAGCTGCACATT

CTACATNGTCACCTCACTCAAAACGTCCTATTTTTTGGGAGGCTAAAACC

ATGATTTGGTCAAACAGTAAAGTGCATTATCCTCACGATTCCTGTAATGA

AAGTGTCTGCCCTGCCCCTGTGCCCGTTTCGCCGGGGCCTGCTGCTGTGA

TGAGCGAGCCGCTCTCTCTCTCCCGCGTGCGCACAAATGCGCTCGAGAGA

AAAAAGTGTTCTGATCGTATAACATTTCAAAATGCAATTGCGGGAAAAAC

ACAGCTATCCTGTTGTCACTGTCGAAGAGAGGAGGGTCTCAGCTTTCTGT

AGGTATACTTTTTATTTCTCAACTCTCAATATTCAGCTCAATAGCAACT

>rnd-5_family-1946#Unknown ( Recon Family Size = 18 Final Multiple Alignment Size = 17 )

TTTTACTTCCTGCTTTGCTCCTATGGGTACACTCGCAATGGCCGCCAGTC

CACCCATTATGCGATCATTGACTTGAACGGGGACGCCCGTTCTATTCATT

CTATTTCTATTGCAGCACATGCAGTCAGGAGCGGAAGAGAGAAGAAAATG

TGCACCTTTCGAACGGTTATTACGGTGACCGCGGTCATTTGGCTGGCCAA

TAACCGTCATCCAAAATTCCATGACCGTCACAGCC

>rnd-5_family-28#Unknown ( Recon Family Size = 19 Final Multiple Alignment Size = 17 )

TCCGGGTTAGAGTCCCGCTCCTTGAAAGCGGCAGCTCTACCCTTTAGCTC

AGTGCGGATGTTGCCTGTAATCCATGGCTTCTGGTTGGGGTATGTACGTA

CAGTCACTGTGGGGACGACGTCATCGATGCACTTTTGATGAAGCCAGTGA

CTGATGTGGNGTACTCCTCAATGCCATCGGAAGAATCCCGGAACATATTC

CAGTCTGTGCTAGCAAAACAGTCCTGTAGCTTAGCATCTGCTTCATCTGA

CCATTTTTTTTGACCGAGTCACTGCTGCTTCCAGCTTTAATTGTAAGCAG

GAATCAGGAGGATANAATTGTAGTCANATTTGCCAAATGGAGGGCCAGAG

CTTGGAGAATTGCAATACTTGGTCGCATCTTGGGGTCNCCAAATCCCTTA

ATTTACAATTNGACGCCACCTCCACGCCAATAGGCTCTTTGNAAGGGTTG

CCAGAATTTTTTAAAGCTTTATTGAGAGCGACCAACGAATGTNAACGCTT

GGAGTTTGCTAAACGGCATTT

>rnd-5_family-1590#Unknown ( Recon Family Size = 22 Final Multiple Alignment Size = 17 )

GCTTTGGCTTTAATACATTCTGAAAATACTTGAAAACCAAAAGAGAAAAG

AATAGTAGCCCTCCTCAGGAACAACAAAACCCTCACAGACCAACTTCTTC

TTCTATGATATAATGGAGGTCCTCAAACCAACGTTAAAGGTGCATGCCGC

CACCTACTGTGCTGGAGTGTGTTCAATCACGGTTTACACCACTCTAAATC

CTCCTACCTAACTCAGTACTTCTGAGAAAATAAAAGAGTCCTACTAACTT

CTAATAGACCCTCCCCCATCCCCCAAATCTCTTCCANNNNNNNNNNNNNN

NNNNNNNNNNNNNNNNNNNNNNNNNNNNNNNNNNNNNNNNNNNNNNNNNN

NNNNNNNNNNNNNNNNNNNNNNNNNNNNNNNNNNNNNNNNNNNNNNNNNN

GAATTNAATTGAGAGAGAGA

>rnd-5_family-444#Unknown ( Recon Family Size = 18 Final Multiple Alignment Size = 17 )

TTTTTTTTAAACGCCACTTTAAACGTGTACATGACACTGCAACAAAATTT

CCCCATGGGGACAATAAAGTCAGTA

>rnd-5_family-604#Unknown ( Recon Family Size = 23 Final Multiple Alignment Size = 17 )

ATTAAAGGGCAATTCCGCCACTTTTCAACCTCATATTCATTATCTCCAGC

ACCATACCAGTGTCTACATATGTGAAAACCGCGCGTTTCTATGATCTGTG

GTTAAAAAGAAAAGGTCCTAAAAAAAAATGCTTCTCTGTGACATCACAGG

GTAGGATTAAAAGTAAAAAACTGATTTTCAAAACCTGCAACGAGTTTCTA

GCCCGAGGGAGGGTATTTTCTTGCT

>rnd-5_family-5302#Unknown ( Recon Family Size = 19 Final Multiple Alignment Size = 17 )

GGAAAACATGCAACTATACGTAAATATCGGTACATTTNATTGCCCTTATG

CCTAAAACAAATGCAATATAGACAAAAACGAATAACAATGAAATGCATAT

AAATATCTATTCATGTTTATTTTCCCCCATTAAAAACACTCTTACATATC

TGTCTAGGTTGGAACTGTTGCTGTTAAAATACAATAAATAATTTTCATTC

TGAACTGGAATAAACTGATTGATCACATCACACAGACGNATAACAGAACG

CACACACAGGCTTTTTGATAGTGCAACCCTAAGTAACAGTACAGATGAAA

AATGATCAGGCCTACTGTACATTTTACTGTAGAAATTATTGTTGAAAAAA

AGTCTAGGTTGGAACTGTTGCTGTTAAAATACAATACATAATTTTTATTC

TGAACCGGAATAAACTGATTGATCACATCACACGGATGTAGACCAGAAAA

CACACACACACAGTCCTAATGAGAGGTGTGTGGCTGGTTGAAGTTTTCAA

CAAGCATGTCTATTCTGGGCTCAGTTTTTGACAGTGCAACACTGAGGTCA

TGCTCAGCATTGAGACTGTTTCTGTACTTGTTTTTTAAAGTATGTCAGAG

TTGAGAACCCTGACTCACA

>rnd-5_family-4273#Unknown ( Recon Family Size = 22 Final Multiple Alignment Size = 17 )

AACACGCATCCGATTATTCATTATGAATAGGATTTATTTCGTTTACATTC

TTCATTGTAACCACAATGTCACAAATAATTAACACACACACAGCAGATTA

ACTTGGTCAAAACAGTCAAAACATTTCTTTATTAAAGACTATCAGAAAGA

ACGTTGGTACTTATTTATTTTGATCCAAAGCCACGAATGAGTGAGTCACT

CAGCTTTATGCTGACAAATCCTCGCTGGACTCTCTTTCATTCAGTTTCTT

CTTCACATCAGCAAAGAAGGACTCTGTGTTTCAGAAACTCACTTTATATA

GAGGGGCTATCACTCTTTCACACTGTGGTAAATAAAGCCNGTTTGTTATT

TAAAACCAAAAGCCCACCGTGCCTATTTTTCGAAATTCGTCAGTCCACAT

GTCAAGTGTAANTGCGCCATTGTATTTACCCAAGTTCTCTCTCAACTCCA

GGACCACCGCCAGC

>rnd-5_family-614#LINE/L1 ( Recon Family Size = 19 Final Multiple Alignment Size = 17 )

GTTTTCTAAAGAAAGATAATCTATCTGAAGAAGAGGAGTCTGTGTTCAAG

TCTTTACAACTAGAATTAGAACAGCTTTACACGGATCTGGCAAAGGGCGC

CTTTGTAAGGTCAAGAGCAAAATGGATTGAAGAGGGGGAAAGAAACACTA

GTTACTTTTTTGCACTTGAAAAGAGAAACTACAAAAGAAAATCTATAACT

GCACTCAAAATTAACGATGTTTTATGCAAAGATCCCATTACAATATCATC

ATTTGTCAATTCCTTTTATGAAAACCTTTACAGCTCTCAATTTCAGGAAG

ATGGTTGTGAAAGCTACATTTGCCACATTCAGAATTATGTCCCTGTAATT

GAGGATGATTTCCACTCAGTTTGCGATTCACCTGTGTCAATTGAAGAAAT

TAGAGAGGCTCTGAATTCAATGAAAAAAGGGAAATCACCTGGCCCTGATG

GCCTGTCAGTTGAATTCTATAGACAGTTTTGGGAGTTACTAGAAGACCCG

ATTTTCAATATGTTTCAAGATTGCATTAAAAATGGGGAAATGGTCTCCAC

TATGAAACAGGGCCTTATTTCATTGATTCCGAAGCCCGATAAAGACCCTT

CTCTCATTGACAATTGGAGACCAATTACTTTATTAAATATTGATTACAAA

TTGATTGCTCCGTTTTTGCCGAAAGACTTAAAGAAANGAATAGNCANCAC

TATAAATGGGACT

>rnd-5_family-418#Unknown ( Recon Family Size = 18 Final Multiple Alignment Size = 17 )

TGCTGTTGACCCGGATCACTGGTTGCTGCGGAAAAGGAGGAGGTCAAAAG

GGGGGTGAG

>rnd-5_family-2400#Unknown ( Recon Family Size = 19 Final Multiple Alignment Size = 16 )

CAAATGCATCCAACAAGTTTGTAGAGTCACAAGCTTGATGTAGTCATTGC

GTGCTAGGAATATGGGACCAAATACTACACTTTTGAATACTTTAAAGGGT

CAAAAACTA

>rnd-5_family-1018#Unknown ( Recon Family Size = 33 Final Multiple Alignment Size = 16 )

TAAGGGATAATCAACGAGGGGCTATGCGTTCTATGGAAAATAATGAACGA

CGCGGAAGGTGTGCTCCGCGAGCGCTAGCGGGGTGGAACTGACCTTCCAC

GGAGTTGCATTATTTTCCAGAGAACGCATAGCCCCGAGTTGATTATCCCT

TTTATACCATGGCTATAATTTAACGCATTTGCCGGTAGAAATGTGTTCAT

TTGCCGGTAGAAATGTGTTCAACATCCACTGAAGTAGCTAGCAAGTTTAC

TAGATAGCGACAGTAGTTGCCGTGGTAACCAAACCATCAGTCCTGCTAGC

TAGCTATTATGAAAATCGAATTCAACAATGCCAATAATGTTTTCAATTCG

ACTTTTGCTTTCAAAAGCAGCTCAAACATAGAACATGTAAGCTGTGGCGA

T

>rnd-5_family-2463#Unknown ( Recon Family Size = 18 Final Multiple Alignment Size = 16 )

CAGCCATTTTGATCAGGCCACACCTACCTAATGGGATCAAACCATACCTT

ATGCTGCGTTCACGTCATGTCGGAAACTCAGAGATTTTCGACTTGCTTAC

TCGTTGTAGAAAGATGATACCCGAGTTTCCCAATTGGGAAGTATCAGAAT

CAACCAATGGGAAGCTCGACGCAAATAATTTGCATTCATTTTAACTCAGT

TTCCGACAAGATGTGAACGCAGCATTAATCTCGGTTAACCCAGAACTGAA

GTCTTGCGTAATTGGTCAGATGCTCGATNAAGTTCTGGGTCCATACGTGT

TAAGAGAAGATTGAGAACGAGGATCGGAACCGGAACGAAGAGCTCCACCC

TCTCTATAGGCCAAATGTCCCATCCCCTTCCGAGATTCGGAAGATGCTAA

AACCTGCGAAATCGTGATTTGTCCAAAGCACNGGAACTAATGCTGCTCGT

CGTCTCACGCATCCCGTTGTATCGTGACAGATCTACGGTACCGTTTTANG

TAGTCTGTCCACGAGAGATTAACCACGCCTTAAAGACGGTTGAAGAACCG

TTTAGGG

>rnd-5_family-2377#Unknown ( Recon Family Size = 18 Final Multiple Alignment Size = 16 )

AGTTTTTTCCAATTGCTTTACACACTAAAATCTTTTCATGNCACACGATT

ATCGAAACCTCNCACTCAAAGNGCAAAACCACACACCAAATCTCCAAAAC

CGTAAGCACATTTCTCTGCTTTACACTCAGTTTGCAATTGCATAAAACAC

TTTTTTTCAAAACACTACACACAATTCTCTACATAAGACACAAAAATCTA

ACAGGAAGTGACTTGCTTTCCTTTTCNAAACACAACCAATCAAAATGCTA

CACTTATTCACCAGGTCACACACGCGCTCCTCACTCGCATGTGCAAACAC

TAATAGCTTAACTGATCACTAACCAATCACTGCTTTACTGTAGTATAGGC

CTATAAATAGGTCAAAGGTCAGATTACCTGTTTTGAACAATGGACGCCAA

CAATGGACGGAGAGCAAGAGGAGGAGGAGGGAGAAGAAGAGGACGAGGGC

AAAGAACCTGAGGAGGTNAGGGCAACACTTGTTGATCATGTGATCAACCA

CGGTTTGACCATGAGAGAGGCTGGACTGAGAGTCCAGCCCAACTTGAGTC

GATTTACAGTGGCGT

>rnd-5_family-6337#Unknown ( Recon Family Size = 19 Final Multiple Alignment Size = 16 )

AGTTGTCTCATTAAATGCTTTCAANATTTGTATATGAATTTCTACAATTT

ATAGTTGGTTTNAAGNTTTTTAAGTCATTGAAATTTGGAGCTATTGNCCT

TTTAANATATTGTCCCATTTACATTGTGTAATTTACCGATGGTCCCTAAC

TAGCCCCATAGGGATATCCAAAGTCGAACCAAATTTACCACGGGCATTAT

GATCGGGTCGCGTGCAGCTGCACTCCGAATTGATGATTACTTGTGTGCTG

CCAGCTGTGGGCAGGGTTGAAAATAAACCCCGGGAAAACAGTTGCCGTAC

CCTTGATTCATTCATAACGTGACATACCATTNTTTCCTANCATCTCACAA

ATCTCCGTTTTGGACGAGACTGACTTTATGACCAAAATTATCCTATTTAC

ACTTTGTAGTACATTTTNACACTAGAATAAATGTTTCTGACTCATATCGA

TGCCACATAGGCCGTTTTCAAAATGAGAAGTCGCTTTTTTTAGGGGCAGT

CGCTCTTTAA

>rnd-5_family-565#Unknown ( Recon Family Size = 21 Final Multiple Alignment Size = 16 )

AACCGCAGTCCCGCAATGTTATTTTAGGCGTATGTGACGCAGCTCACTTG

CCAGCCATGGTCCCAGCAATTTTGCGCCCTATAGGCAATATCAAATGCGC

AAAAAATAACTTAAGAAATAGGTTAAATTACCAGAGATNCTCGCGTGGCC

CATTTATATTAGTGTCAGTATTACTGGGCTATATCAGCCAATATGCTAGA

TGTAGTTTGAGGAGAGCAGAGTTGGAGAGACTTGCACTTTCTCTTGAGCT

ATTTTTATAGCCTACCTTTTAGGAGCGGGAAGATTTTCAGACGGAGAAAT

ATGGGTGATCAATATTTAGGCCTATTTCTAGGCAATGTAGTAAACTATAG

CCTAATCATAGGCCTATTTAGGAAGTACATTTCCTTGGAAAGATAACTGT

CCCGTGCTCTATTTAATTGAGAGCAC

>rnd-5_family-37#DNA/TcMar-Tc2 ( Recon Family Size = 16 Final Multiple Alignment Size = 16 )

CAGAGGGAAGAAAAGCAGATGGCCCAAAGTATTTGCAGCCACTCGACATC

AGTGTAAATCGTGCATTTAAGGTGGCGCTCCNTGTTCAGTGGGAGGCTTG

GATGACAAGTGGGGAGAAATCCTTCACTAAAACGGGCCGCATGCGAAGAG

CAACTTATGGTCAAGTCTGCCAGTGGGTCCTGACAGCGTGGAGCATTGTC

AAAAAATCCACTATCATCAACGGGTTTCGAAAGGCTGGACTGCTGCGTGT

TGAAGGGGCAGCATGAGCTCAGCGGGGTATTTGCCTCCGGATGAAAGTGA

CGAGAGCGACAATGAAAACGATCCAACATCGGATGAAGCAATTCTGAGGC

TATTCAACTCCGACACCGAAGGAGATGACTTCAGTGGTTTCAGTGCACAG

GAGGAGGAAGATAGTGACCAAGTTAATTTGTTTCAACGTACCGGTAGGCA

CCTGCGGCTTATAGACATGTTTCAACGTACCGGTAGGCACCTGCGGCTTA

TAGACATGTGCGGCTTATTTATGTACAAAATACATATTTTTTAATAATTC

AGTGGGTGCGGTTTATATTCAGGTGCGCTTAATA

>rnd-5_family-1396#Unknown ( Recon Family Size = 23 Final Multiple Alignment Size = 16 )

ATATANTTTGCCATATTAAATTATCATTGTTATAGCACACACGTTGATGT

CAGACATGCACCTACCCCAATGCTCTGTTGCTGATGACTGGGATGAATGC

AGGAGCAGATTTCAGGAGCAGGACAAGACACCCTCTTTTTGACTGATGAC

TGACATAAGGGCATGTACGTGATAGGCCTATCTGGCTTATATGATTATGA

TGGTCATAATGCTTCTTGACAGTGTCATAAAGTGTATTTTCTTAGTCCAA

GTAAAGTGACACAGGACGGTTTAAGAAACAAACTTTCAAACGAAAGGAAA

CTTCTTGGCAGGGAAAAAACNCNTTTGAATAAATGTGGNTTTTGACACTC

TTATGTAGGTGTCATAACCAGCCATAGAATAACACAACATGTGTAANCGT

ATGGGT

>rnd-5_family-422#Unknown ( Recon Family Size = 25 Final Multiple Alignment Size = 16 )

TGTTGGCCAATCATAAGTCATCAAAGCGACAATNGGCTACAGTCATAGAG

CCTCCGTTCGGGACGAAGGTTAGGCGATATCTAATCAATGGAAGATGGAA

TTAAAATTACGGATTTAAAAATTAAAGGAGAAGGATATGCTACAACGACC

AAGAGCCAACAGGTAGGCTGCTACTTNATANTCTGAATGGAGTTGTTGCT

GTTTGTAACTGTGGGACGAGAAAGAGCAATGCAGCNCCAATTAGCCTAGC

TAGCTAGCTAGCTTAACTAGCTCCTCCCGGTTTGATGCAGTCAAGACAGG

TACTACGTAATCATATTGGATGATAAATAACCTAGCTATGGCAGCTATTA

GTCTACTATAGCGCGAGTCGGCTTGGTTGGTTGCCGTCTTTTCTCCTCTC

CCTCCCTCCTCCCTGTTCCCGTGTCACTCACACACACTCACAGTAGCCTA

CACACAGCACGGCCCCTGCTAGAGCGTCACGTCTCTCTACCTCCTCTCCT

TCACGCTTTATCAGCTCCGGTTAATAAAGTAGCTTAAAAATNGACTTTTC

CGCTGCTTCTCTCACTCGGTCGGACCGGGTCTGGATCCGACCAGGTCTAT

ACGGAACGGGTCTAGTTGTCCTCGGGTCCGTT

>rnd-5_family-2017#Unknown ( Recon Family Size = 19 Final Multiple Alignment Size = 16 )

GTGTAGCTAGCCACTGGATGGCTCTGAATGCACTGTCAGCATGCAGTCAA

ACTACTAAGCGCTTTTGGAGCTAACTAGTGCTCTCAAATCGTNTCCTGAT

GTCGACGGCAGATTACGTATATTCATGANATAGNCAGGTTATATTAAGTG

GCTAGCTAGCGTTAGCAACGTTTGGTGGTCAACGAGACTGAGAGCTGAGC

TAGCAAACAATGTAACAAAAGTATAATTTCGGATTTNAAAAAATACNCCA

ACAGGCTCTGCGTTTCAGGAATCACAGTAGCAAGTACTCCTGGTACACTG

TTAAATTATTTAGCCATTTATAGCCATTCACTGGCTTTCATGCGATTTAA

ACGTGAGCTTGTCCGAGGTGTCGGACTCGACGACAGTTGCTATCCATTGG

AGCGCTGCGATTGGTTGCCCAAAATTCTGGGGCGGGGCTTAGCGAAGGGT

CAATTG

>rnd-5_family-686#Unknown ( Recon Family Size = 19 Final Multiple Alignment Size = 16 )

TTACACTGGTCACTCAAAACATTTATAAAGTATTTAATAAAATAGCCCAC

ACTTTAGATGAGGCCACGCAAAAATACGTAACTAATGATTAGTAAATGGT

TTATAAGGACCTATATTAAACGTCTCATGAATCATTATTAAATACTTTAA

AAGTTGTTTATAAATGTGTGAATAACTAGCTTACTAACATTTACAAATGT

GGGAGTAATGATTAATAAATGGTGAGCAAACTGTTTACTAATCCNTTACA

NATGCTTTATTACTGAAGTTATTATAAAGTGTTACCGAAA

>rnd-5_family-659#Simple_repeat ( Recon Family Size = 19 Final Multiple Alignment Size = 16 )

TTAGTAGACCGGGGCGANCCAGACAGTTAGCAGGCCGATGATAACAGGCT

AGCAGTTAGCAGACCGGGCAGGCAAGCTAGCAGTTAGCAGACCGGGTTAG

CAAGCGAGCAGTTAGCAGGGGCTAGCAGTTAGCAGACCGGGGCAGCAAGC

AAGCTAGCAGTTAGCAGACCGGGCAGGCAAGCTAGCAGTTAGCAGACCGG

GTTAGCAAGCAAGCAGATAGCAGGGGCTAGCAAGTTAGCCTTTGGGGGAC

GTCGCGATGGGGGTAAGTCTGTTTTTGCCTCTTCGTGCGGTGACGTCGAT

AGACCAGTCGTGGAATTAGTAGGGTTCCAAGTAGCTCTAGGTAGCTAGCA

GGCCGCGGTTAGCAGAATGGGCCTTCAGGCGGACGTCGCGCCTGAGGGGC

CTGTTGGAATCCTCGGGCAGATTACGTCGGTANTCCNGTCGTAGAGGATC

GGCGGGGTTCCGTGCCCCGTACCGGCAGTAGAAGGGGTCCGGATATTGTA

GCCCAGGAGTGGGCTTCGGTGGTAGCACAG

>rnd-5_family-1246#Unknown ( Recon Family Size = 25 Final Multiple Alignment Size = 16 )

GCAGAAATAATTAGACAGGACCCAAACGTAACAGGTATTGGTAAACATGA

ATATAAACTAAACCTATTTGCAGACGATCTCCTGATATACCTGACCAATA

TTGAAAACTCAATGCCCCCCTTGTTAAAAATATTTTCAGAATACTCTAAA

ATTAACGTGGAAAAAACTTAAATAATGGCAATAGGAAAAATAAAATAACT

CATGATCTACAGCAATCCTTTAAGTGGACCACAAAAAATACTAAATANTA

GGATGCTTAATAAGTGACAACAAANAAAGCAGATNTAATTAAATNGATAA

TCTCCCCATAAATCTTACAGGAGAATAAAACTCTTCGGAATGGCATNGCT

TCCAAAGTTTTTGTATTTATTTTCGGTAATACCAATTACCCCACCGAAGA

CATTCTTTAAAAAAGTATACTCGGCCATAACAGACTTCATATGGGCAAAT

AAAATTCATAGAATAAAAAGGAAAGTTTTACATCTCCCTAAGTCTGAGGG

TGGTTTTAACCTTCCAGACTTGGAATTGTATCAACTCGCCACCCATCAAC

TCGCCACCCAAGGCTTTTACTTGCGACATATTGTTAAACGCACTAAAGAG

GAACAATGGGTACATATTGA

>rnd-5_family-1355#Unknown ( Recon Family Size = 21 Final Multiple Alignment Size = 16 )

ATAGGCTGCCGCTGTTACAGTATGCTGCCAATCAGCAAGTGATGTGCATG

TTGTCAACAGAAGAGTCAGTGAAGGCACAGTAGGTTACTGGCAACATTTT

GCCACTAAGTTATTGTGACTTTTACAATAACTTACTGACAAGTAGTTTCC

NGTTAAGAAACGACAAATTCACAGCAACAAGTCGCCANTAAGTTACTGGA

AAATGCACAATAACCTCTTCACAGTGCAGCTCATTACCGACGCATTCTCT

TACAAAGTTTGCAGAACAGACAGTGTCGAAAGAAGCGCTCNACTTGCATC

GGCATAANCATCAAACGCTTAAACTGCTACAGCACTTCCACTGACGNTTG

GCACAAATACACAACTATTCGACCACGCTAATGAGCCCCACCGGTACATT

GCCCCCACCTATCAAAGCGCAGTGATGATCGTACCTTATATTCGAAATAT

TGGGTAGNAAAAAGTCCCATTATACCTACAAGGTACCGNACTGTACC

>rnd-5_family-9#Unknown ( Recon Family Size = 26 Final Multiple Alignment Size = 16 )

AAATATGCTATAGGACGGGTCCAATCGGGTCCAGTCGGTAAATCAATTNT

AATTGCACATACCCGAGACCCGTGGCAATCATATCAGACCCGACCCAGAC

CCGAGACAAAGGTTNGAATTTCGGACCCGGACCCGCTCGGGTCCCGGGTC

GGGTCTCGGAATTTCGGGTCTGGTGGACCCGTGAAGACT

>rnd-5_family-1334#Unknown ( Recon Family Size = 19 Final Multiple Alignment Size = 16 )

GAAAACGGAGCAAAGTAGNGTACAGTGATGAGAATGATCCTTCGTATCTT

GTAGGAGTACGGTTTGTTAATGAGGAGCGGCTGATCGGATTACCGATCTT

GAGGAATCCATTTGAGATATCCAAACTGGTGGANAGAGTGCTGGGGTAGA

GTGAAGGNTGTGAGGATCACGAGAGGCGGGCTTGTTTAGATTTTTATTTT

TGCTTCTGAGGATCAGAAGGAACGTGCGTTGCGTCTCACCCGATTTGATA

AGTTTGAAGTGTCGTGTGTGTCTCCTAGGAGCAGGGCACCCCTCAAGGGG

GTTATATCCGGCGTCTCGTGGGAAGTNGATGTTGAAGAGATGAAAAATAT

TCCTGGAGTGATTGATGCACGTCGGATGAATCGTGTGGTGAATGAAGAAA

AAGTGAAGAGTATCTTCCGTTCTTTGTTGTTTTTTGATGTGGAGTCTCTC

CCTACTCAAGTGCAGTTGGGNTATATAAACTACAGNGTCAGAGCGTTTGT

TCCAAGACCGATGCANTGTGATCACTG

>rnd-5_family-530#Unknown ( Recon Family Size = 17 Final Multiple Alignment Size = 16 )

TCACTCTCAAAATTACATTTTTTTTTCATNNNNNNNNNNNNNNNNNNNNN

NNNNNNNNNNNNNNNNNNNNNNNNNNNNNNNNNNNNNNNNNNNNNNNNNN

NNNNNNNNNNNNNNNNNNNNNNATGCAAACAGCGCATGATGACTGAATTG

CGCATCACAAATAGCCTACTAACCAACACTTCGGACTAAACTTTTTCAAT

ACCTGGTTTGCATACCCATTGTTGCCTTAGTTAGCATTTACTGGTAGATA

TCATAAGTCGAAACGTCTTTCCTACCCTACCGGCTACCGATGTCATTCTT

CTGTGAGCTGCTCCATGCCAAAACCAGTCGAGAGCTGCGCACTCTTGCTG

CCTGCAGATGATATTCCGCTGAAACTAGGCTATGTGTGCGGCGCGCATGT

GAATATATTTCACGTGCTTTGCGATTGTGTAGGCTGCTTTGTGCATGATN

TCTCTATTGTACTACGTATTGGATAAGTCGTATACCTCCACTACACTACT

TTGATACGCATCAGTGGGGATTAAGAAGTGAGTATACGCAA

>rnd-5_family-171#DNA/hAT-Tip100 ( Recon Family Size = 21 Final Multiple Alignment Size = 16 )

TCATCCACGAAAGCTTTTTACACTTTCAGTCAGCTGAAAGCTTAGATGCA

GCAGGTCTCACAAAAATGATAATTGATTGCCTTGAAAAACATGGTCTGGA

CTACAGAAATAATCTTGTGGGGCAAGGCTATGACGGTGCATCCGTCATGA

GCGGAAAGCATTCTGGTGTGTCTGCACGGATTAAAAACAGTGCAAGATTN

GCATTTTATGTGCACTGTAATGCACATNGTTTGNATTTGGTTCTTGTCGA

CGCTGTAAAATCAGTGCCTGAGGCAGTTAACTGTTTTGCTCTCCTGCAGA

AGCTTTATAACTTTGTATCTGGCTCGTACGTTCATCTCANGTGGCTTGCA

GTTCAGAAAGAGCTGTATCCACAGCAGCAGCCCGGGGAACTACAGAGACT

TACGGGAAGGTGGGCATGCAGATACATGGCATGCCGTAATCTGAGGGACA

GNCTTCCAGCAGTTCTGAGAGCGCTACAGGATATCACACTTGAANATAGT

GGTGGTAGATCAGTGGAGGCAAGGGGCCTTCTTTCTCAGATAGATTTACA

TTTCATAGGGCTTTTGGTTACCTTTTGTAAAGTGCTTGGTGATGCCAAAT

GTCTTTCTGACATGCTCCAATCAAGCTCTCTTGACCTAGCAAGGGCTG

>rnd-5_family-513#Unknown ( Recon Family Size = 20 Final Multiple Alignment Size = 16 )

TACCGCAGGATTTTGTTCCAACTAGGCACCACACGTGACCAACTGAGCCA

ATTGATCAGTTCAGGGATAAATTCGCCGAACTGATCGATAAATTCAACAC

ACCTGGTCTTCCAGGTCGGTTAAATCAAAAACATGAAGTGCCTGCGGCAC

TCCAGGACCAGGGTTGCCTACCCCTGCTCTAGAGAGTCTGTTGATAAATT

CGAATGAAAGGTACACTAAATTTAAAGGGATGGCTTTAGATAAATGTACG

GAAAACCCTAGCGAATGAAAACTCCATGAGAANATCTGTTGGCCAGCCAG

TGGGAGTGTTTTCAGTTCCATTTCTGTGTTTTTTTTATT

>rnd-5_family-2494#Unknown ( Recon Family Size = 17 Final Multiple Alignment Size = 16 )

TCTCTCAAATTTTGTGCACAAATTTGTTTACATCCCTGTTAGTGAGCATT

>rnd-5_family-1704#Unknown ( Recon Family Size = 18 Final Multiple Alignment Size = 16 )

AAAATGAAGTGCTTTGTAACACCAAAACTAGTGGCAACTGAGCNGCCACC

AACTCGAAGGAGACGAAACCTTAACTTTGCTGGAGTATTGAAACACATCA

TCCTGAGATGTTTTGAAACATGACATGCGGTGTGTTGTAACACCACTTAA

TCAAGTGTTGTGCAACACCTTGCCAGGGACCGGGAGCACTACCGGAAAAG

GTTGAAAACACTATNTTGGTGTTTCGAGTCCTGTTAAGTGCAGAATNAGA

TACCTTCTCTTTTGGTGCCGTTTCCTCTCTCTANNGCTTCTAAACACCAT

TATAATGTTTCGAATCCTGTCTAGTGCAGAATTAGATCCCTTCTTCTGAA

GCGTCTTAATGTTTAACGTTGTTTTATGTATTTGATCATTTTGGCATGCA

TTGTCAAGCACAGTGTTCAAACCACCAGCACTANCTGGCGGTTGANCTGC

AANAATTGAGGATTCTGCACTTGCCTCTTACATTGTGAAANCTGGGAACG

GGCGTGGCCACATTGAACGTTAATTTGCCTGACGGTTTCGATATCCGTTC

ATCGTGATCCATTACACCTCATGGCA

>rnd-5_family-3063#Unknown ( Recon Family Size = 17 Final Multiple Alignment Size = 16 )

TGATTTACATAAATTCACTGAAAACCCACACTAACACACGGTTATATTAA

CAGTATTGCACTTTTCATGTAGCCTACTTTTGGCCAGCTAATAGCCTAAC

CACCGATCAAGCAACATTATGGACTAAACGTTNAAATCCTGTCGCTGCAG

GATTATTTTGCTGTGACAATA

>rnd-5_family-342#Unknown ( Recon Family Size = 20 Final Multiple Alignment Size = 16 )

GTCTCGGTGAGAGGTGTAGGAGTCAGGCGCAGGAGAGCAGGGATGTCCGA

GAAGCGCGTTTAATAATATAGTCCACCAATACAAAATTGGCACAGACGAA

AATCCCCAAACTACGCTCTCGAANAAACAGAAACTCGTGCAAACGCACGG

AGGAACAAACACAGTTCCGACACTTTACATACA

>rnd-5_family-3667#Unknown ( Recon Family Size = 18 Final Multiple Alignment Size = 16 )

TACAGTGGTTCCTCCTTTAAAAGTTGCGAGCTTACACCGCGGGACTTAGA

GGTACCCAGC

>rnd-5_family-516#Unknown ( Recon Family Size = 17 Final Multiple Alignment Size = 16 )

TGACTATACTATTTCCCAGTCACCCTCACCCAATGAAGACTTGAGCGTGA

GTACTCAATATTCTCAACTGAGCTCAGAAGAGAGTGTCAGAATCACAGAG

TCCTCAACTAGTCTGATTCAGAGCATTAAGAAGCTCTCTAGCAATGACTT

TCAGACTCAGGCAGAAGAGGCAGTGAGTAAAGTGCTGATGAGATCCAGTC

ATTCCTTTATCACACAGATCAGCCATACTGGTCTTCAGAAAAGTCTGCAG

GCTGGCTTATCATCTAGCTCACCATCAGAGATCCATGTCCTTTCAGAATC

CATGTCCTCCATGTCCTCTGAGAATACAGCCTCTGGATTAGTGGAAACCT

TTGTCAAAGGAATGGCGACTATTTTCCAGAAAAATGAGTCCACTGACACT

GTGCTACTGGAAAGAAGTGGGAGAGTTTCACAGTGCTCCCACGGGGGCTC

CCAACTGGATGATACTGAATTGAGTGTTAAAATATCAGAGGAGAAGCTTT

GGTCAACAGCTAAGAACATCTGCGTCAGTATGAAGAACACACTTAAGGAT

TTCTTCACAGGGCTGAAGCCATCCGGATCCGAAAGGACAGAAATGGCTTC

TTCCAAAGAGACCCTTGGGGAAATCCTGGTCGCTATCCAGAGTGAAATCT

CAAA

>rnd-5_family-2902#Unknown ( Recon Family Size = 17 Final Multiple Alignment Size = 16 )

AACATGGAAACGTACATTCAAACTACTTTTTGGGGTACCTCGTCAACATT

ACATGAAATCCATGTCTTAAACGTTGCTACGTTTCGGGAAAACGTGTATT

CTGCTTGACACATTAAAGTGACTTACCTTACAGATCAAAGTAAGCTGATG

TCCACTCCATTCAGATGTAAATCCTTTTGATTCAGTCACTGGCTTGTCTG

GCTGTCATGTTTTTCTTTTAACCTGCCCTCATCTACACTGAAATAATATC

CTTTCAGTAGGCCTCTATTATAATTCACCCCCCCCCCCCCCCTTCATTAA

TACACCCTTGTGGTTGAATATATATATAGTTTTATGTACGTATGTGAACT

TGTTAGATTTTACTGCATGGTTAGAACTAGAAGCACAAGCATTTCGCTAC

ACCCGCATTAGCATCTGCTAACCATGTGTACGTGACAAATAAAAT

>rnd-5_family-843#Satellite ( Recon Family Size = 16 Final Multiple Alignment Size = 16 )

TGGACAGTTTTCAGCAAGTTGCAACANAACAGAAATAACCACTTTTCCAT

AAACAAGGTCTAAATCATGTTTGATGCACTCTGGATGTCCAATTTTTGCT

GTGCTGTCTATTTGAGATGAAATGAACACTGTGGACAGTTTTCAGCAAGT

TGCAACATTGAAATGCAGAAATAACTTCTTTTTTTCCATAAACAAGCTCT

AAATCATGTTTGATGCACTCTGGATGTCCANGTTTTGCTGTGCTGTCTAT

TTGAGATGAAATGAACATTGTGGACAGTTTTCAGCAAGTTGCAACATTGA

AATGAAGAAATTAACTTCTTTTTTTCCATAAACAAGCTCTAAATCATGGT

TGATGCACTCTGGATGTCCAATTTTTCTGTGCTGTCTATTTGAGATGAAA

TGAACATTGTGGACAGTTTTCAGCAAGTTGCAACATTGAAATGAAGAAAT

TAACCTCTTTTTTTACCATAAACAAGCTCTAAATCATGTTTGATGCACTC

TGGATGTCCAATTTTTGTTGTGCTGTCTATTCGAGACGANATGAACACTG

TGGACAGTTTTCAGCAAGTTGCAACACACAGAAATAACCACTTTTCCATA

AAC

>rnd-5_family-411#Unknown ( Recon Family Size = 18 Final Multiple Alignment Size = 16 )

CCACTCTTTGCGTCCGTGGACTACTCCTCTCCATTCTTCGTGGCCTTTCT

CCGCTGGAGATCTGTTGGCGGATTGGATTGTCTGACTGACCGACTGACTA

CCACCTTTTTTGTATACGGTATTTCATTTGTTTTGATATGATGTATACGG

TGATGATTTATGTAGTTAGATGTAGTTGAGGAATTAGTAAGAGCTGATAC

GCTTTAAAGTTCTGTGGTAAAATTGTTATATTGATTGTATGATTTAATAC

TGGGTTTACGCTACACGGAATGAACGGACAAACATTGCGTGACNAAGGTC

ACTTGAGTTCCCTGAACTCCTTTACCGGGCTGGACTCTTTTTAGGCCCGA

GGTACAGGACATTTCTGGAGGAACCAGAACTCATTGTGTTATATTATTAT

ATGTGTGTGTGTAATCATTTATGTTGGTAATACAACCCACGCAAAAGAAT

AGTTGTTTTTGAAGTTCTTTCCAATGTTTTAAGGGTTTATGAAAAGTTTA

TTTCCNTCCTGACTTTNACATGAGTCCTTTGTATTGGTGTAGACTTGACG

GACCAGATGGGACTCATT

>rnd-5_family-3548#Unknown ( Recon Family Size = 20 Final Multiple Alignment Size = 16 )

GACAATTGCCAGGGATCCTCACGATAATATCACGATACTATATGCATTGC

GATTCTCACGATACTATACGTNTTGCGATTCGATACTGTGATTTTATTGC

GATTCGATGTTCAAACATATTGCTCACTATATATGTCTGCTGCAGAGAGA

CGAGAGAGCATGAGAAAACGAGTTTTGATCAGTCATGGAAATAAAAGTGC

TGAAAACACGTTGGCTCACTATTTAAAAAGAAGATGGAGAACAAGCTATA

GGACGAAAAATACCGGAGTTTTGGCGCAGGTACAGCCGACTAGCGCTAGC

TAACGCTACCANTAAAAAAAAGGTACCTACAGCATGTTCTCCACCTTGGA

GTCAAAGTATCGATATAATATCGTCCAAAATAATATTGCGATATGTAACT

GTATCGATTCCCACCCCATCACTACTAAAACGCACTCTAACACTCACCCT

CTCACTCACACACAACCGGTTGTATCAGCGTAGTGTGAAAGGCGAGCAAT

AATCCCCTTCCCTGGTGTCTCGGTAGCAGAGAGAGCGGAATGACAGGATC

AAAGGCTTTCTGGAAGAGCTGTCAGAAATCAATGGAGTGAA

>rnd-5_family-1155#Unknown ( Recon Family Size = 16 Final Multiple Alignment Size = 15 )

GGGCTCTACAGTGCGACCATTTTGNTCGCATATGCGCCTAAATATTTTGC

TGTGCGACCTGGAATTTTNATTTAGGAGCACCAGTGCGCCTAGAAAAATT

AAGGATTCTAGCTTTANTACCTCAAATATTTTTCATTGTGCTCCTACATT

TCTTTGTGTGCGCCTACATTTTTCAACTTAGGCGCACGCGTGCTCCTTGT

AAAAAAGGTCA

>rnd-5_family-1313#Unknown ( Recon Family Size = 17 Final Multiple Alignment Size = 15 )

AATTAAAAAAAAAAAAAAAAAAGTTTGCTCCATGAAGTAATCCAACAGCT

GTGTACGCCGCCATCGTGTCTATTTCAACTCTTCTCTCATTGATTGAGAC

AGGTGGGTCCACCCCGAAGTGCNTCATGTCATGATGACACTCGCCTGTCT

CGATCAATGAGAGAATATTTGAATTAGACAAGATGGCGGCGTACACATCG

TTGGATTACTTCATGGAGCAAAGAATTGATTTANTTTAATAACGGAGCCT

CTTCTAAATTCAAACGAACCCACTGCAACTAGACACGGACGTTTAGAAGA

CGTGTTGTCTTCCAAGTCGGGAATTNAGGAAGTATTCGCCGNGTAAAGGT

TGGTCAAAATTCTCTGTGCTACGCCNAACAGTACCTACCCTATAACGGCT

GTATGGAAACGTTCACATATCGCCGCCTTTTCAAAGTGTAACTATAGAAG

AGCCACACTCATTTGAGCAGAGATTAGGCTTCCCCTTGAATGGGAGAAGG

GATGACGGACAAGGAAAAGATTGTCAGTACTTGTAGTAT

>rnd-5_family-4849#Unknown ( Recon Family Size = 16 Final Multiple Alignment Size = 15 )

ATTTTATTAAAATGTCTAATATCATGGTATATCCTTGTATGTAACACGGA

TANTTTCATTTTACGCAAAGCNTTTTCANTGTTAAAATAGGCCTATACAT

TTTTTTTCTCAAAAATGAATACTCACGCAAGTAATCGAATACTAACGTCC

GTTCGGATATTCGAATAACCGTGCCCATCCC

>rnd-5_family-2543#LINE/L2 ( Recon Family Size = 16 Final Multiple Alignment Size = 15 )

CAGTATGTTACCCCTTGGCAGCATTATCAGAAAGCACAGCATTGATTTTC

ACTGCTTCGCATTTTTGTCTCACCAGAGGATTTTAGCTCCACGGATAAAT

TATTAGACTGTATTAGTGATTTAAATACTTGGATGGCTCACAACTTCCTC

CAGCTAAATCAAGACAAGACCGAGGTACTTATTGTTGGAGCCAAAGCACA

GAGAGAGAATCTAGCCGCACATTTTAATTCACGGGCAATAAAGATAAAAC

ACCAGGTAAAAAACCTAGGTGTTATTTTAGATTCTGAACTCAATTTCGAA

TCACACATTAGGAACGTGACCAAAATAGCTTTTTACCACCT

>rnd-5_family-1071#Unknown ( Recon Family Size = 20 Final Multiple Alignment Size = 15 )

CTGGAAGTTTCACTATATTTCACAATAAGGTACNCATATTAAGTAAGACT

TACAAAAGCTTTAAAATGAGGGCCAGGTTGTCAGGATGGGTAATGTACTG

TATAAGTACACATTCATTACAAGTAAGTACCCCTCAAGATACACNTAATT

ATAACGTAGCTATGCGGATATTACATGTACTNTGTGTGTTTATCCTCTCA

CTTGGACGGCGCTTCCAGAAGCTTTAAAACGAGGGCCAGGTCGTCAGGAT

GGGTAACGTACTTTGTANGTACACGTTAATTACAAGTAAGTACCCCTCAA

GATACACTTNATTTTAACTAGCTAAATCCTGCGTAACACCTAGTAATTAC

ANTGTACTTANGCAAACATNTAATGTACTATGCTTTGAANTAGTGTGTNT

TTTCCTCATCTGGGATGAGCAAGTTAACCCAGATGGTACACTTTATTTTG

GTTGCAAGTGCTAGCAATAACGTTGAGTGGAAATATATTTTAAACAGTGC

GCATTCACGGGTAAGTAATTAGAGCCTATTGAGCATAATCTCGCGGACAC

CCAGCGCATCCACGAGGGGTCNCAANCTTCGTGACGGTTGTTACTGAATC

GGATGCAGCTGAGAAGAAACGAAANACTAAGTTGGTGAATTTAGTGGAAA

CCT

>rnd-5_family-3420#Unknown ( Recon Family Size = 16 Final Multiple Alignment Size = 15 )

ACTGTGTGAGAGCGAAGTCTCGCATCTCGCTCATCTCAATATCTGCGGTG

CTGCTCGTGGCAACGTCGTTTCGCTGAGTCTACCTTT

>rnd-5_family-1615#Unknown ( Recon Family Size = 16 Final Multiple Alignment Size = 15 )

GAGTACGGTTACATGCGCACAATAATACGATTATTGTGGATAGTCAGATT

AATATAATAGTTCGATTAAAACGTTTACGTGCT

>rnd-5_family-784#Unknown ( Recon Family Size = 16 Final Multiple Alignment Size = 15 )

GTCAGCTGTTANAAAGTTGCCTTGATGTTGCTATGCTTCTCACTGAAACT

GGCATTCTATATATTTTTCAGCAAGGAAACAATGTTCTGCCTTGTGTAAA

TGCTATATAAACAAACAATGTAGCTAGCATAAACTGGGACTTAGGTCTAC

TAAATCTACCTTTAAATGTGTGGCCAACTGTTCCTAGAGAGCCGCAGTAT

ACGAGATTTTTCTCCAGCCAAGCCTCAACACAATTGATTCAAACTAATCA

ACTAATTATGATCTTCAATCTAGACTGGGGACTTCATCATTAAGAGACAG

TTGTCTACTAGTTGTCTCTTCTTCCTGACTCNGGTGTATAAGGCTTGGGT

GGAAGAAAAGCAACATAAAATTGAGTTATGCCTAATATGGCAATTCATAT

TATTTTTCCAAAATGAATTATTGTTAACTTACATCTCAAAGCCACGCATG

CAAGAAGCCTTGGCCTGGTGCACATTTCTCTGTCCTCGGTTTGATTGAAG

GCCTCAATCTGTCTTTTTGCCTTGTAACTGATTGCTTCTAGAAAGAGAAC

AACACATGATCAAGATGAGCTAGCTACTAGTTTAAAGCAATGAAATATCT

AATTGTTACAAAGAATGAATGTATGGGCTACATTTATGAGAATAATGCAT

GCATTTGCTTAC

>rnd-5_family-264#Unknown ( Recon Family Size = 26 Final Multiple Alignment Size = 15 )

AAAACGATGTTTTAAGTGAGAAGTAGGCATTGGTCTGAAGCCGAAAAAGA

AAGGAAATTCACATGAGCACCACAATGCCTACTCCACCCATGCTCTACCA

AGGTTTTCCAGCACACAGCTTTGACCTACTACAGTAGCTATGTTGGTATT

GTACTTCAGGTTGCTTAGGATTCAAACCTTCTCAAACAGCCTAATTCATA

CTCTTAGAGGAGGTGCTCCCACAATAATGTGATTTGTACTGCATACAAGG

TAAAGTATTGGATTCTTCACATACCACACTAAGACATTACATCCTACCTT

TTCAATATTTGTTTATGAAA

>rnd-5_family-285#Simple_repeat ( Recon Family Size = 19 Final Multiple Alignment Size = 15 )

ACTCCTCCCCTCCGGCGTTCGACGTCGCCGGTNTACTAACCACCGGTCCT

GG

>rnd-5_family-1011#Unknown ( Recon Family Size = 21 Final Multiple Alignment Size = 15 )

AAAAAAATCGGGGTGAAAAAAATATTATTTTTCCCTCACAGAAGTAGTGC

ACTGGGCCTTTACTAGTCCTGTATTAGCGGACCGATATAGACCGTAGCGC

GGGCAAAAACAGCAAAACATTCAGCATCTCCGCTTTGGCAAAAAAGGTAT

CTGTTGTATAATTAAGAACAGTATCTTGCAGGATTCAATAGTTGGAATTG

TAAGCGTTGTTGCCCCGTCCCTTTCTCTGCGATCGTCATTCTAATGGAAT

CCAGAAAGAACNANACCCTGTCCTCAAACATTTACATCAAAAGGTGCAAA

GTTATGGGCAAAGACCCAACACATCCACATCAAATGTAATATTTTTCTTG

ATCAAGTAACATGGAAAACAACCACTTTTTGTGCAGTATTAATTTACCAT

CCTTACAA

>rnd-5_family-1470#Unknown ( Recon Family Size = 16 Final Multiple Alignment Size = 15 )

GATATTTACAATATNAAAATAATAAGAATCAAAATTGTCAACGGGACAAC

AGTAACAACAATAACCAAGGGTCAAAATAACCATACATTGAACAATAACA

ATAAGCATACAGTAGAGGACATGTGCAGGTTGATTGGTCTGTCAGACACT

GTCCCTCATCTTATGGCAGGCAGCAATGTAGTGCGCTGCCAACCCACAGC

TCTCTGCGTCCTCCCCCAACAGGACGGGTAGCCTATCCTCATCAGAGAGG

TCTTTGAACAACGGTTTCAAATTTGGGGAAATGACGTTTTATATTTTTGA

CATCTTTNNNNNNNNNNNNNNNNNNNNNNNNNNNNNNNNNNNNNNNNNNN

NNNNNNNNNNNNNNNNNNNNNNNNNNNNNNNNNNNNNNNNNNNNNNNNNN

NNNNNNNNNNNNNNNNNNNNNNNNNNNNNNNNNNNNNNNNNNNAGGTTTT

TCTAAGGTTTTGATCAGTAACCATGGTCAGAT

>rnd-5_family-1278#LTR/DIRS1 ( Recon Family Size = 23 Final Multiple Alignment Size = 15 )

ATGGCGACCTTTAGAGGCGCTGGAAAGCGGGGAGTTAATATTTCTCTAAC

CCGCTTTACTTGATTGCCCTGACGCCTGCAAAGCGAGTGAGTGGGCTGCA

CGCGCTGTCAGCCCACCGNTCGCGCCTNCAGTTTGCCCCGGGGTCCTCCN

GGGTGACCTTNGTACACNAACCCCACCTTTATGCCTAGGGTCGGCGGCCT

CTATGTTGTCGCGCCCTGGAGTTTGTGGTTACACTCCACCGTTTGTGTCC

AGCATGAGCCTTGCGCATTTTCTTGGATGAAAGCGAGAGTATTGCGCTTC

AGCGGCAACGACCCCTGTATTTTTTGGGGAGGCCCCCTACCTTGTTAACG

GCTATCTTTGTAAAGGGAGGCCCCCTACCTCGTCAACGGCTATCTCGTTG

GATAGTGGAGGCTATTATATTGGAAAATATAATAGCAAGGGGTTACAGCC

CTGAAGGGCTCGTTCCACCAGAGGTATGGCTACCTCTTGGGCACTGTTCA

AAGGCATCTCCTTACGGGAGATTTGCGNTGCGGCNTGTTGGGCCACCCCT

CATACTTTTATGAGGTCTTTACCGACTGGACGTCACTGCACCTTCGTTGG

NGNATACAGTNCTTAGCGTCGGANCCTCTGATGGNTGATGCTGTTGGGAG

TACCCNGGCTTCG

>rnd-5_family-393#Unknown ( Recon Family Size = 16 Final Multiple Alignment Size = 15 )

GAATGCACTGCATGCCAGTGGTACACGACCCTGGGGTGCGTTCAGTTCGC

TTCAACGTTTGCTACATTACGNGGCGGTTTTTGCTGAACGACACGTTTCC

CCAAAACGGTGCGCACCTTTCTTCAACAGCCTTTGAGGTACGTTTGCGTC

CCGTGTGGCGGGTGTGGCCTGATCAATATGGGTGTGACCATTTTGAAAAT

CGCAAAACACGTGCAGCACACCATACGGTCACTTCCCTCTGGGCCACCAT

AATCACAACGTTTTTCAACTGGTCGTTCAGTACCGTTTCCGTTGAATTCG

ACGTTGCACACCGTTCTGTCGTACTGAACGCGACCCTGCTGCTTTTGTCT

CTATAGAACTGAATGCTAAAATCACATGGT

>rnd-5_family-53#Unknown ( Recon Family Size = 18 Final Multiple Alignment Size = 15 )

TGAGGATTTGGCCAGGGTTGTTCCGGTTTTGGTCACTAGATGCCCCCATT

GTGCTTTTTGACCCTTTTGTTTTCCCTTGTTTCCAGTTATTATTTGCACC

TGTGCCTCGTTTCCCTTGAATGTATTTAAACCCTTAGTTTTCCTCAGTTC

TTTGCTCTGTGTTTGAATGTTAGCACCCAGCCCCAGCGATGCTGTGAACA

TTTGTTGCTCCAGTTGGACTCTCTTGTGGTACTCTGTTTTTGTTCTCGTT

TATTTATTTTTTATTATCTTTTGAGGCTTTTTTTTCCTGCTGTACCTACC

GCCTTGTGGATTTACCTTTTTGACTTGGAGGATTACCTTTGTTCTCTTGG

AATTACTTTTGACGTTGTGGATTTATATTTTTGCCTGAAGAACTTTCCTT

TTTACTTTATTAAATACACCGTCTCAAGTACTGCTGTGTCTGCCTCATCT

TCTGGGTTCTGCCGACTAT

>rnd-5_family-404#Unknown ( Recon Family Size = 29 Final Multiple Alignment Size = 15 )

AGTTAGCTAACCTGGCTAGCACGCTACGCGGCGCTTGTTTAGCTAGCCCG

GTCTCGAAAGTCTACGTAGGACCGGATCACCGAGGTGGGAACGGACCCTT

CATCGACAAGTCTGGGAAGAGAGGCAAGTTGTGCTTGACCGAGGCAGACT

CTCGGCGGGGGTTACCCACCGAACCTGGCCGCTCTCGCTGTTCGAAAAGC

CTCCCGTAACCGGCGACAGAGCACACATTGCCAGGGTTGGGCGCAGGCAG

CCACACCATGCACCACGCGCAGGCACACAAGACATTTGCGCTGACCACCC

TATCTCTGGTCCCTGGAAAGGAAAGCCGAAGAATTCTACAAGTCTTTCCA

GGGAGTTTGTATACAGTACGTGGCAATGTGCACACGGACCGGTTTCGAGC

GATAGCCGCCCGAGCGTCCTCCAAGCAGAGAAACTACATAGAGAGCGAGT

ATCTTTCAGAAAAATGCTATAACCCTGACAGGGGGCATGACCCAGAACCC

AGCTTAGCCATCGTTGTCTAGGTCGTTCTCTTAGCCATCTTACTCATTGC

GCTAATAAACTGAATAAAAACTAAAAAAAAAAAAAGAGACATTAGAAAAC

AAGT

>rnd-5_family-700#Unknown ( Recon Family Size = 19 Final Multiple Alignment Size = 15 )

TAGGGTAGGGGTTTAGCGTAGGGACGTCCCAAGGATCCCGGATAGCACTA

ACCCTCAGCGAATGGCGTCAAGTACTCTCACAGCTAGTTAGCTGGGGGTG

GCGAAACGTGAATNTGGACCGGTCCCTGACAAGTGACAACCCATACATNA

AAACGTAGCTACGAAGCGCTACTTTGTAACCTGCTATGTTTGTTTTTGTG

TCTCTGAAAACGTTTGAATGACAGAGCTTGAGGAATACCCTTCGCAAAGA

GCAAATATAACACCAGATTTCGCAGGCTTCACTAAAGGGCTGTAACGTTA

ACGTTACCACAGAGTTGAACGTTCTTAGCACGGCACTTGCTTTACGTTCG

GGCATAGCATCCATTGTTTGTCACGACACCCAACACTTCACGTAACGGTA

TATGGCCAGTGCGCATTTTGGCACTTTTATTGAGCTG

>rnd-5_family-563#Simple_repeat ( Recon Family Size = 21 Final Multiple Alignment Size = 15 )

GTTACTCATTGTGTATCTATTATTNATTGTATTATTACGTGTTTTACTTT

TCTATTATTTCTTTTTTCTTTANCTCTGCATTGTTGGGAAGGGCCCGTAA

GTAAGCATTTCACTGTNAGGTCTACACCTG

>rnd-5_family-4377#Unknown ( Recon Family Size = 17 Final Multiple Alignment Size = 15 )

TTTGGCTACTAATTNTTGTGAANTTGTGTATCAGTGAACTGTATTCTGTA

ATTAGTTACAAAACANTAGAAATGCAATTGTGTATTGCTGCTGTTTGTCT

GATATCCAACAGTCCATGATCAGCTCTGTTGACCATGAGAGCATTTTGCA

GAGAAAATGGGTTGAGGCATACTATTTAGGCTCGGAATGACACGTGGCCC

ATTGTTTACCTTGTTACCTGGCAACGACCACAAGGGCGTTTCAAAGAGCT

GTCAGTCAAGGCGAGCTCATGAATATAAGCTCCCCGCCCACTCAGCCTGT

TCTTTCAGGCTTCCTGATAGTTAGAGATGAGAGAAAAGGTACAATTTCTT

CAATTCTGAGCATTTTAATAGCGTAAAAATATTATGGGTGATGTTTTGGT

CATTCAAAGGCNATTTTTACTTGGGAAATAGGATGACTGTGCGGGACCTT

TT

>rnd-5_family-6640#Unknown ( Recon Family Size = 16 Final Multiple Alignment Size = 15 )

CCTTAAGGGAACATTTCGGCATTTCGTGTATGATCGGTGAGAAAGTCCAT

ATTGCAAATGTACGGTCCCTTACCAGACGTCCGAAANCGTTTGTAAAATT

CGCGGTCGGCGTCGGGCCGTGCGGCAGGGCCGGACCTNCCGGGAAGTCAT

CGAATGAACTCTCTCGGACATTCGGTTTCCGTTTTATAAAATAGTAAAAT

NTTTGTAATTTTTCCGCTGTCCCGGTAAATCCCACAAGATGGCGAATGGA

ATCATATTGCAAATGTACAAAACATCACCAATCACGACGTGGCCNTNTCT

CCTAAACGGAAAAGACTTCGAAGNCGAAACTTGGTGAGCATAGGTTTGGC

A

>rnd-5_family-3395#SINE? ( Recon Family Size = 16 Final Multiple Alignment Size = 15 )

TACCAGCTCACACTGGGAACCGATTTTCACTCTAGTACCCTTCCCTGGTG

GTCTAGTGGTTAGGATTCGGCGCTCTCACCGCCGCGGCCCGGGTTCGATT

CCCGGTCAGGGAACTACTCTTTTGCTACATGTTTACTTGTGCAACCTGTC

ACTATGAATATGGAAGGTTATCGCACATATGTACCAGCTCACACTGGGAA

CCGATTTGCACTCTAGTACCCTTCCCTGGTGGTCTAGTGGTTAGGATTCG

GCGCTCTCACCGCCGCGGCCCGGGTTCGATTCCCGGTCAGGGAAATAGTC

TATGCTCCTTGTTTACTTGTGCAACCTGTCACTATGAATTTGGTAGGTTG

TCG

>rnd-5_family-582#Unknown ( Recon Family Size = 17 Final Multiple Alignment Size = 15 )

ACTGGCAGCACAGTGTGTGTTTGTCTTTCATTATGATTAAACCATGCTGT

TACGGCAAAATACAATTTGCTCTTAACGACTTTCCTATACAGATTAAATC

GCTTACCCGCTCCACAGCAAGC

>rnd-5_family-4633#Unknown ( Recon Family Size = 16 Final Multiple Alignment Size = 15 )

ATACATGTCAAGCAGTGAAGTTTCAGCTCTGTCTGTCCGTGGCCTCTCTT

CCTCGGTGCGCACTGTCACTGTGTCCGTTTCCATCTTGTCCAGCTGTGTC

TGTAACATTTCACGTAAACCCTGTTTCTTGTCTGCATCGAAGTAGCGGTC

CTTGTACCTAGCATCGAGCATGGTGGCGACACAATAAAGNGGCTCAGAGA

GAATGCCACGGAATCGCTAGNTCACAGCCTCTTGTAGAGTACTTTTGCAA

GTTAACCCCACGGTCTGTGTCGGCAGTTTTGTTGAGCAGGCGTTTCAATG

CCGTGACGGAGGGTATCACGTCTGCTGCAGACGCAGTTGATGAGCTTATT

TCCTCGAGTCAGTTGNTCGAATGGAGC

>rnd-5_family-1692#DNA/hAT ( Recon Family Size = 16 Final Multiple Alignment Size = 15 )

AGGGATGCANATTTCGGTCAATTTTGCTGCCGACTAACTGACCCTCATTA

ACCGGTCAACAAAATGGTTAAATTTTTTCCAAACAGTAAAATGACGTGAC

CAACAGAAA

>rnd-5_family-5058#Unknown ( Recon Family Size = 16 Final Multiple Alignment Size = 15 )

TTCACTCCATGGTGCTGAAAAGAAAGCTCTGCTGTTGG

>rnd-5_family-1483#Unknown ( Recon Family Size = 23 Final Multiple Alignment Size = 15 )

GTTAGGGTTAGTTGGTAGGGCAATTTTTCCACAAAGCGTAATGAATTCAT

ACTACAGAATAGTAGGCTGATACACAGCCGGCACAGTAGGTGGCGGCATG

CACCTATAACGTTTGTTTGCGGACCGCCATGATACCAAAGAAGAAG

>rnd-5_family-3979#Unknown ( Recon Family Size = 18 Final Multiple Alignment Size = 15 )

CTACTGGCTGTAAGCGAACGAGTGATGCACGTTTGGAGCAATACTGGCCG

TATCCAAGGCTCAGCCAATCGTTCACATAACAGAATGCAGCACGCGACTG

AAGAGAGAAAGACAGCCAACTTGCTAGTTACAGCATCAGCGGCGCTCTGG

AAAGACGGGAGAGTGGAAAGGCAGTTTGCCAGTTACAGCAGCGCGTCCCC

TGAACGATAACGAAGAGGTAGGTGAGAAGCCTGACCACGGAGACGGGGTG

AGAAGGTGATGAAGCGTACTTCGTGAGCTGTAACCGAAAAACAACTGGCA

TTTGGAAAGTTTGAGGTGAGGGAGAAAGTGTGGTGGAACAAGTATTGTTA

GCATTGTTAAGTATATCTTGCTAGCTGGATCGAATTCTCCGTTAGCTAGC

CAGAGAAATGTTGAGCAACATTAGCCAACTTAACTGATCAATTAACTGAT

CAAATAATTGAGTTTATGGCGTGAAAATTAGCTTGCTAATAAAGTCAGAC

AGCTAGCTTTAGCTAGCT
